# Supplementary figures and images for: trim-21 promotes proteasomal degradation of CED-1 for apoptotic cell clearance in C. elegans (part 1 of 2)
Source: eLife. 2022 Aug 5;11:e76436. doi: 10.7554/eLife.76436 (PMC9388098; doi:10.7554/eLife.76436)

Figure 1A


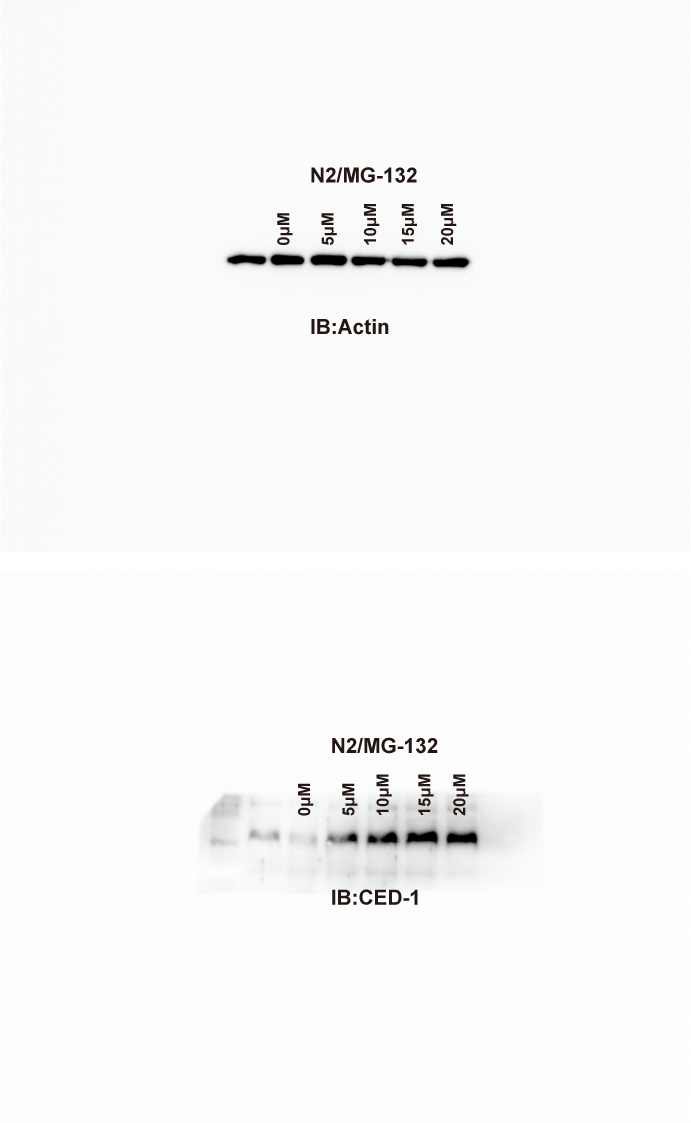


Figure 1B


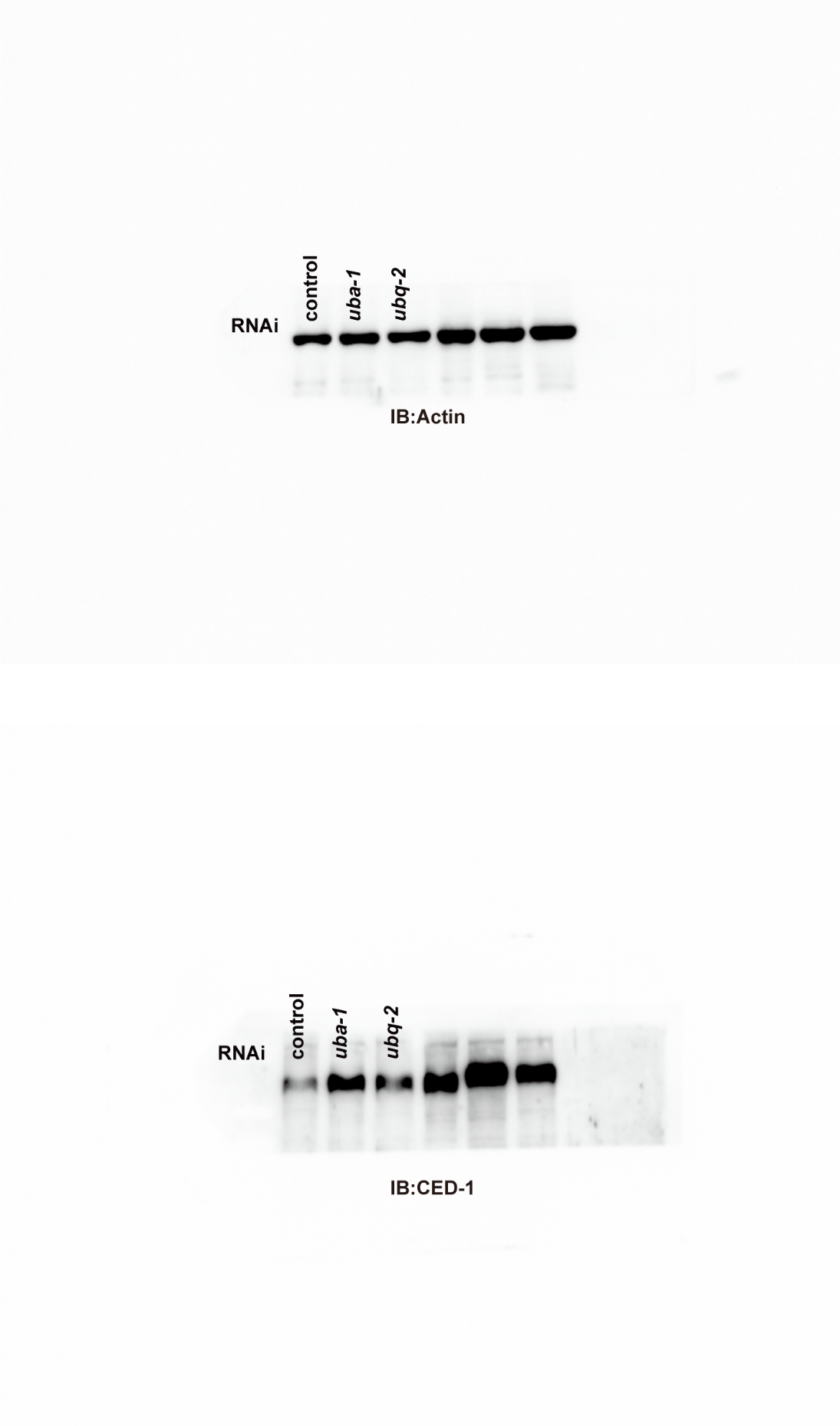


Figure 1C


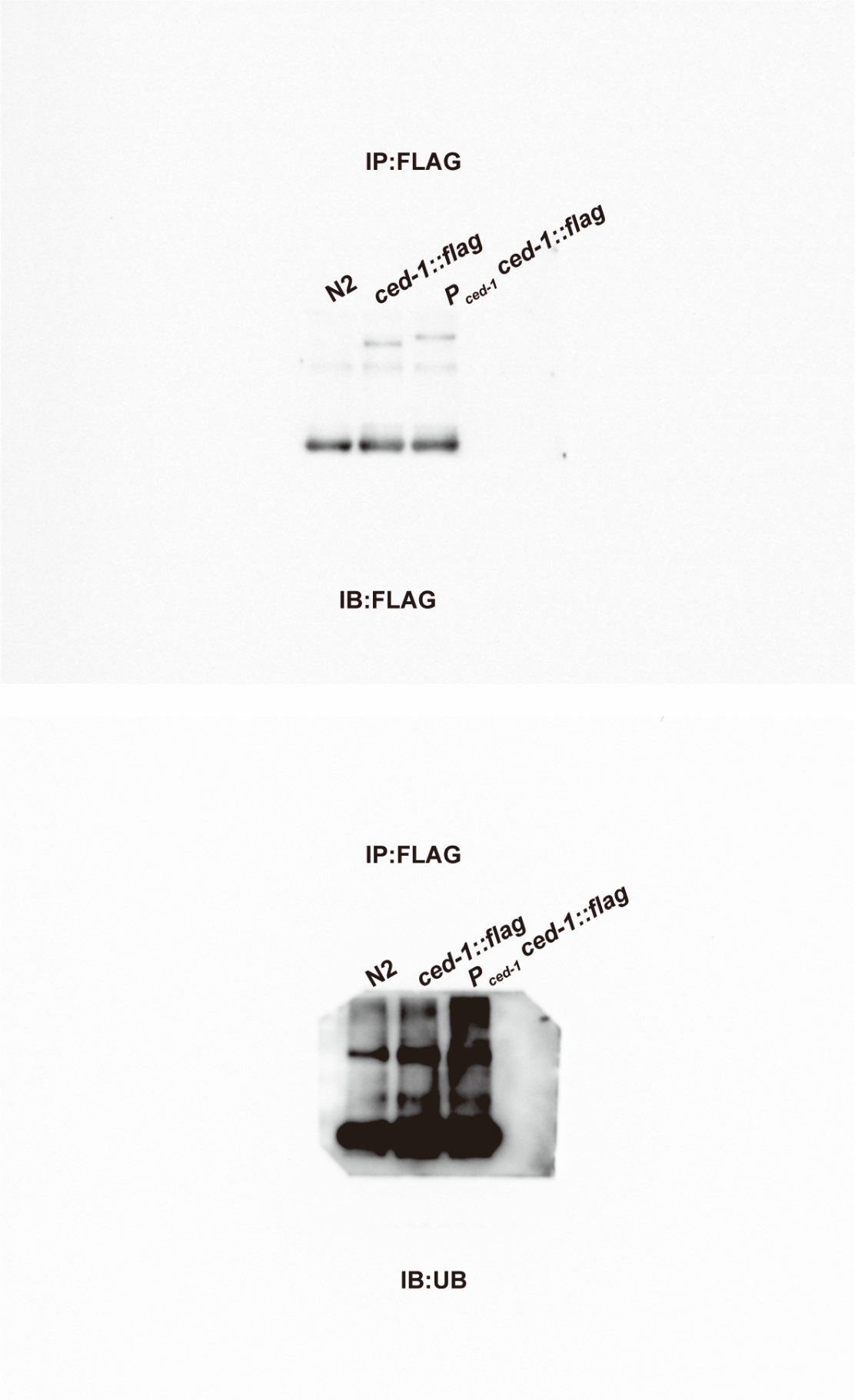


Figure 1D


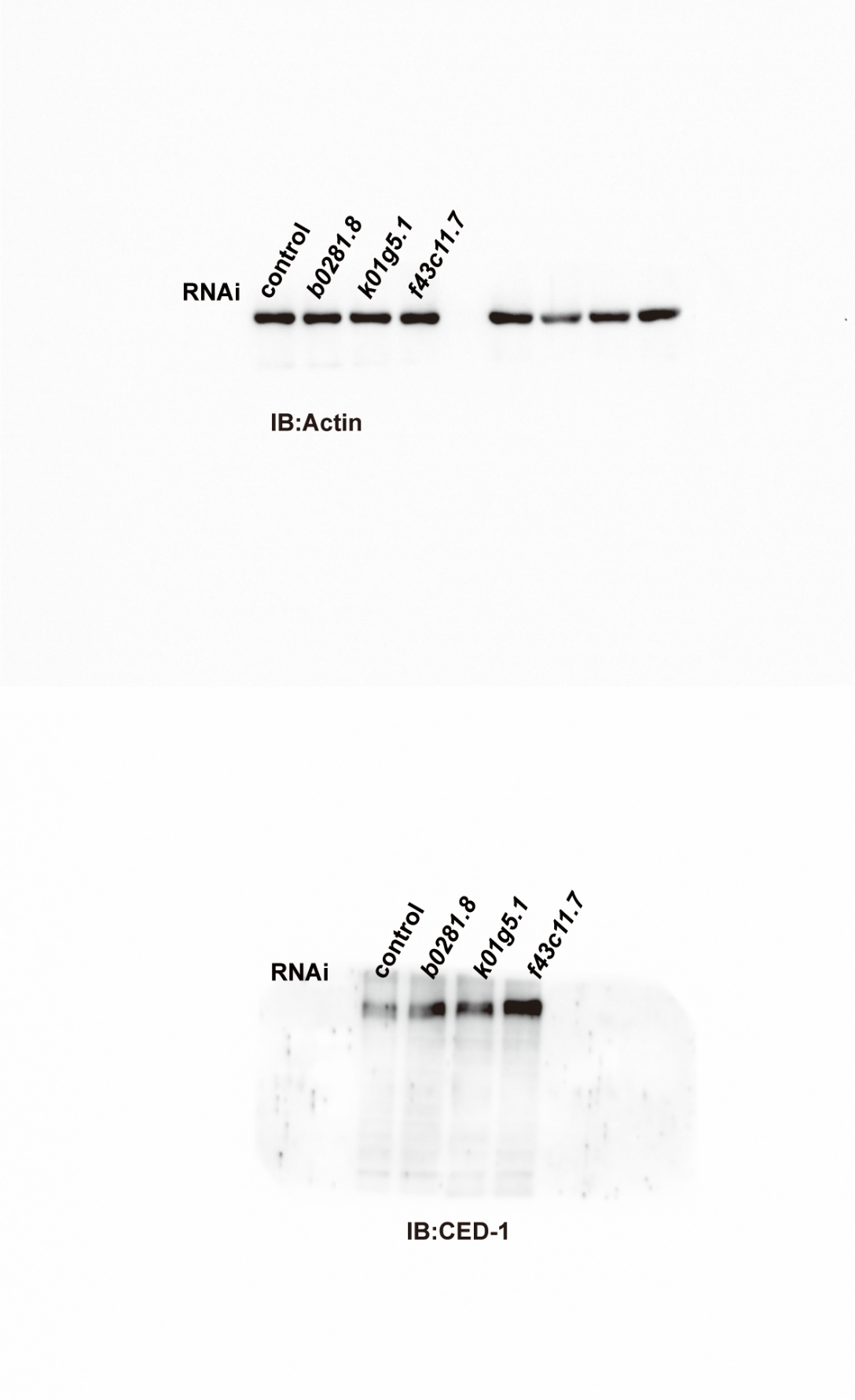


Figure 1F


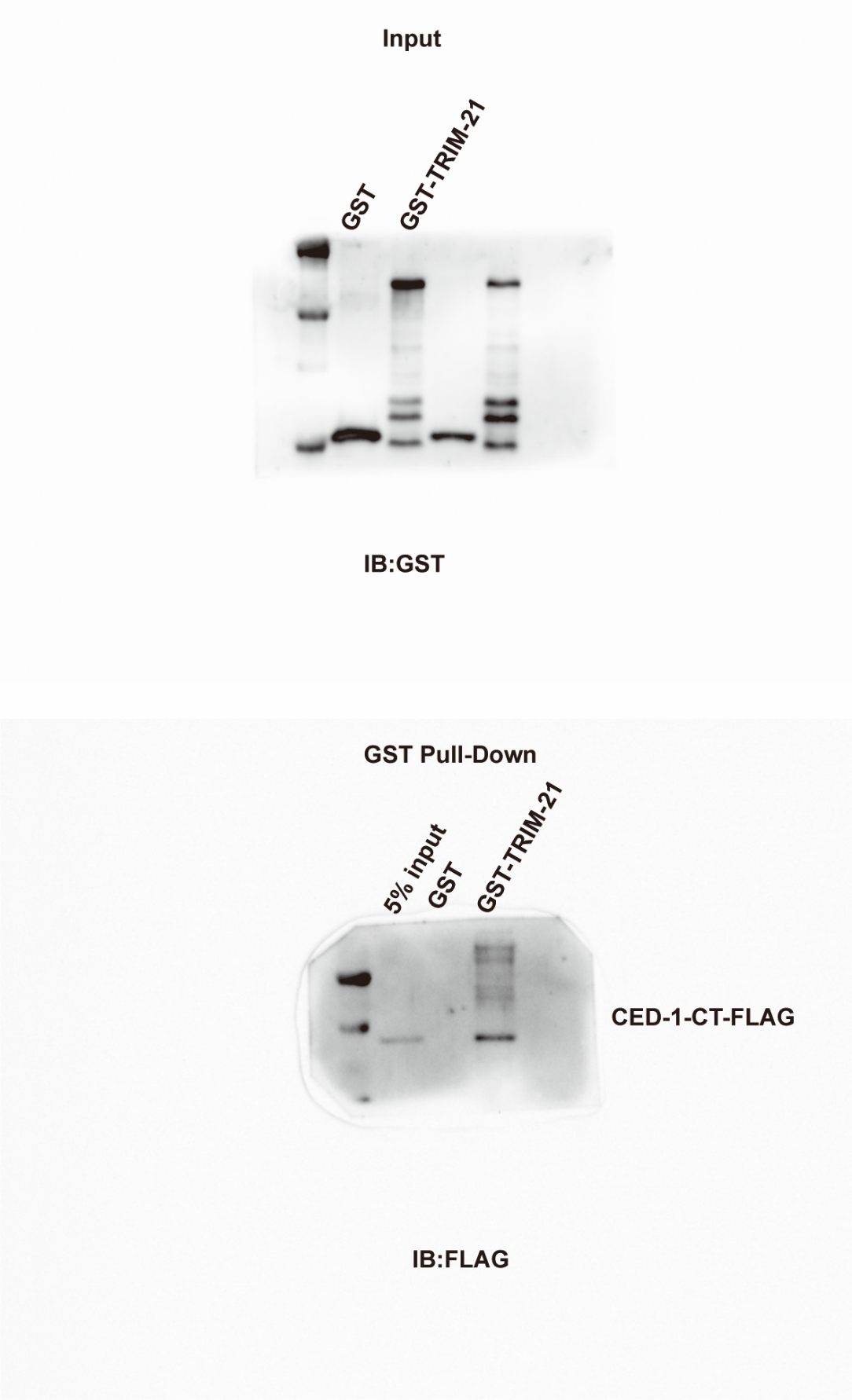


Figure 1G


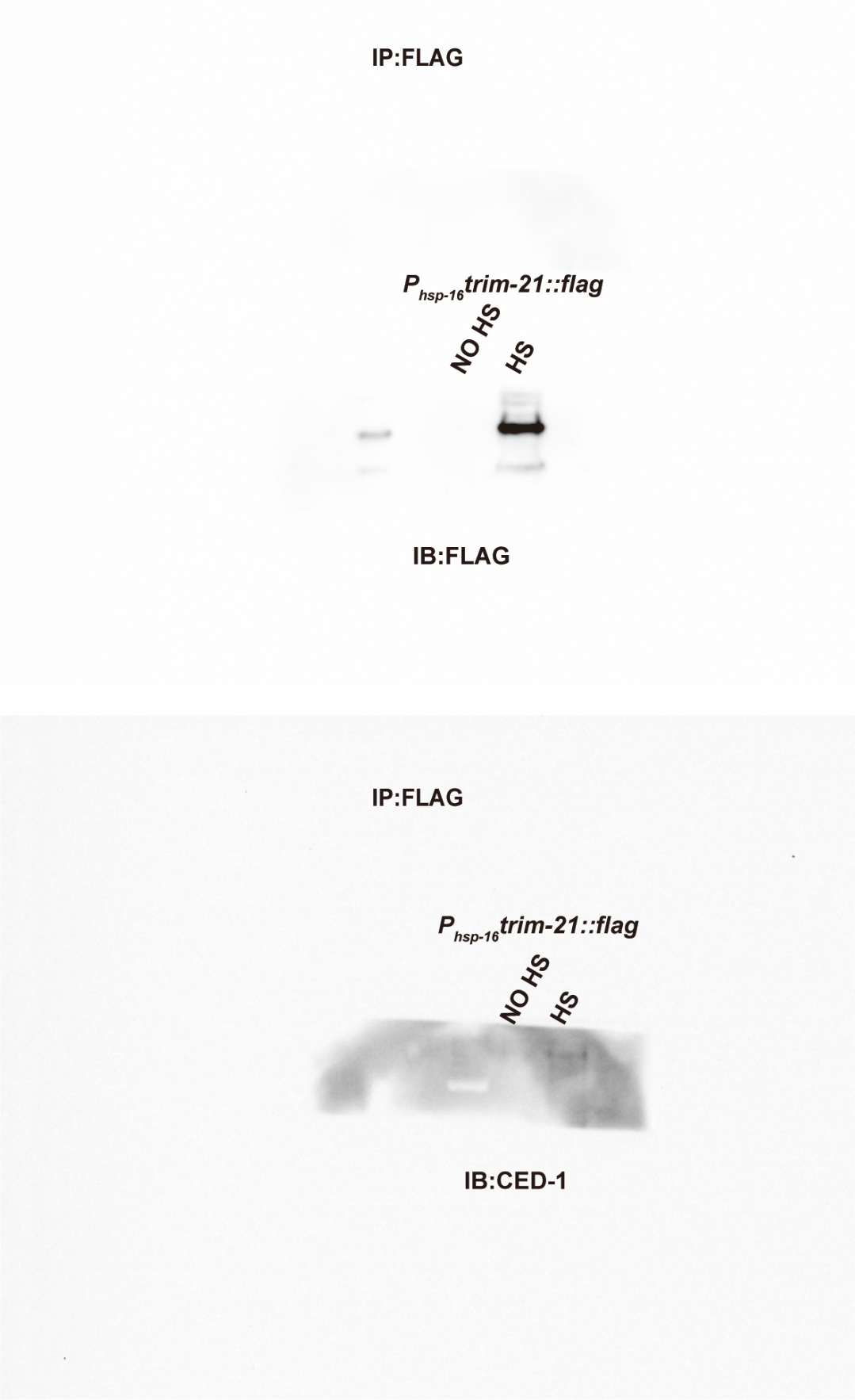


Figure 1H


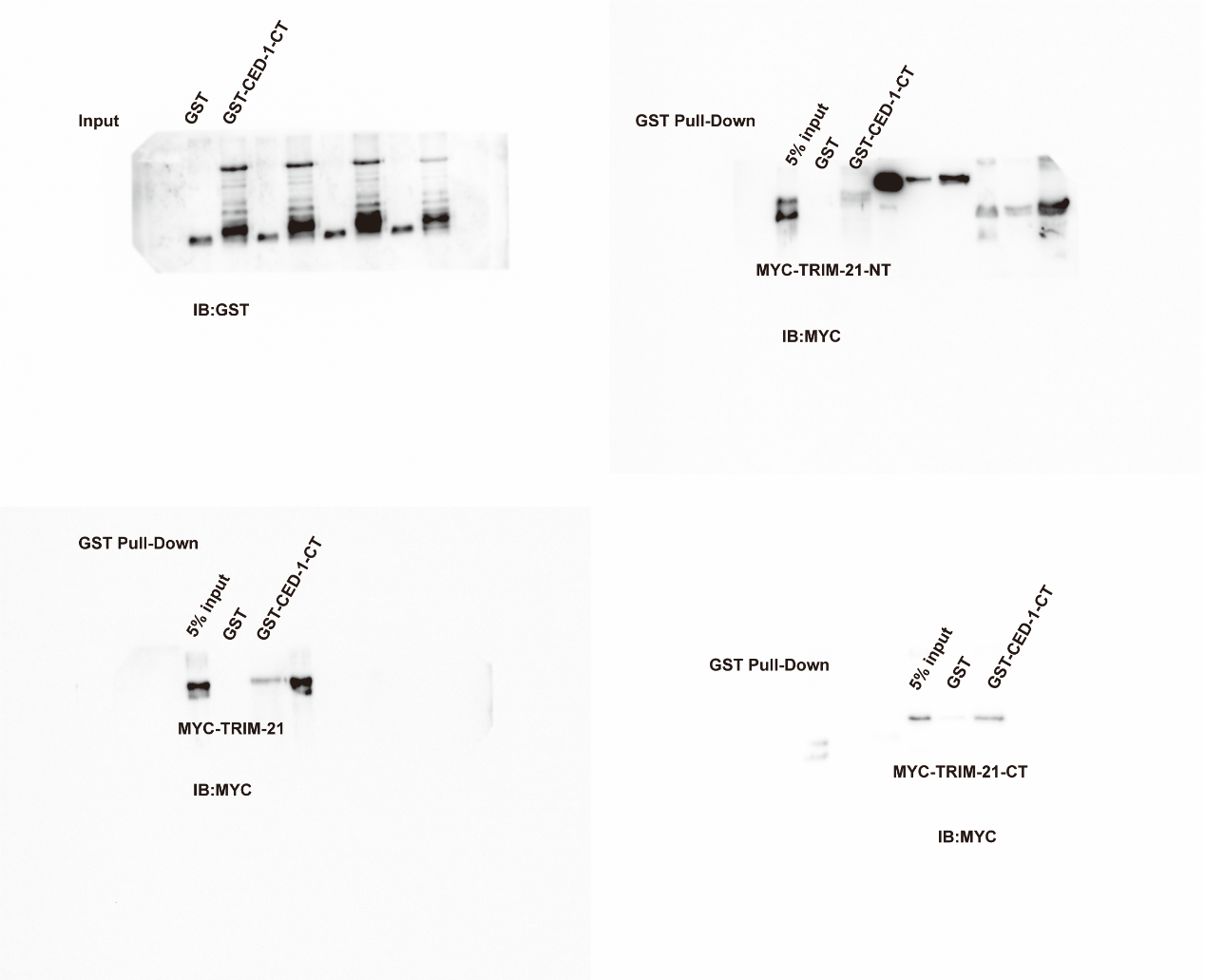


Figure 1I


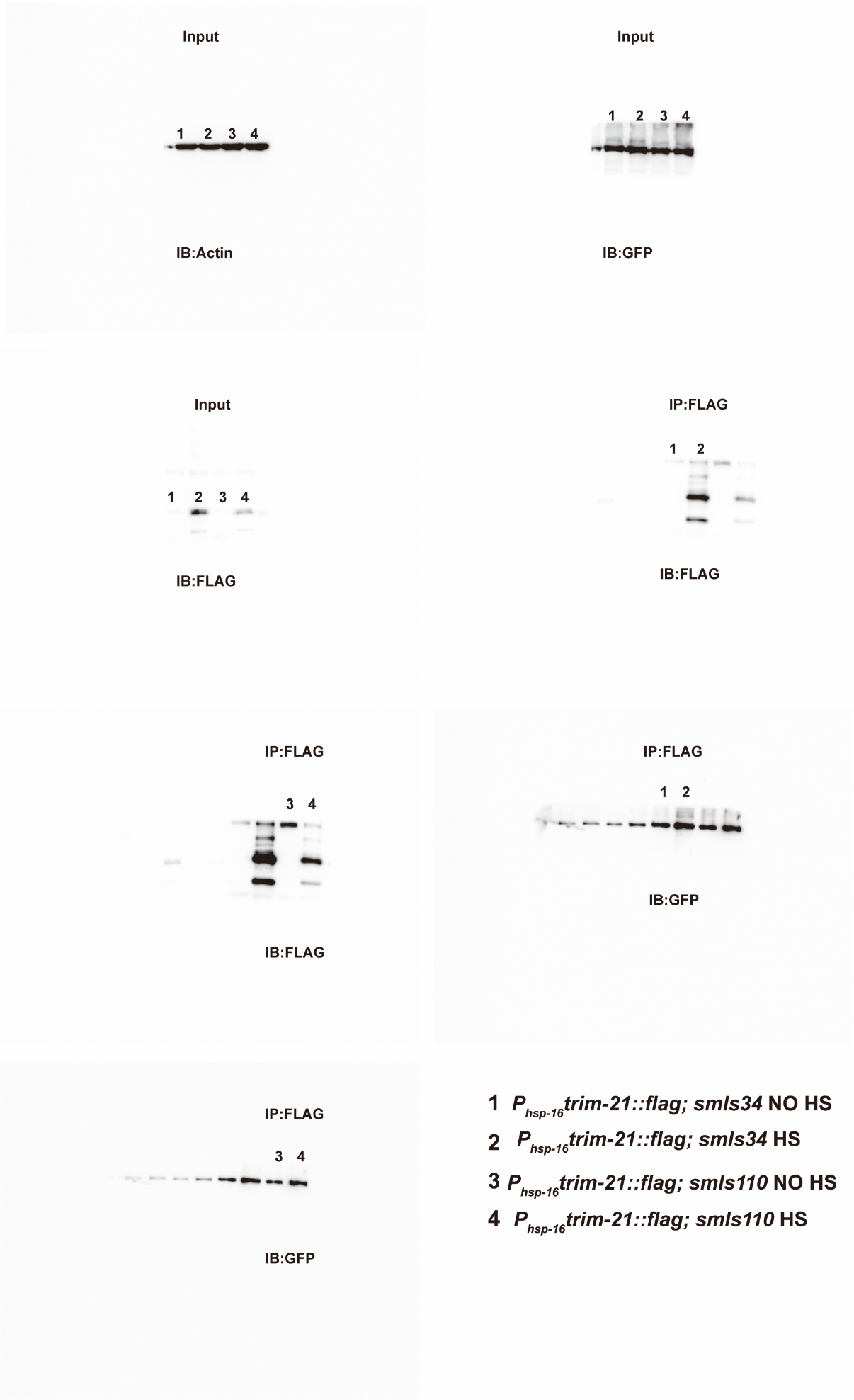


Figure 1J


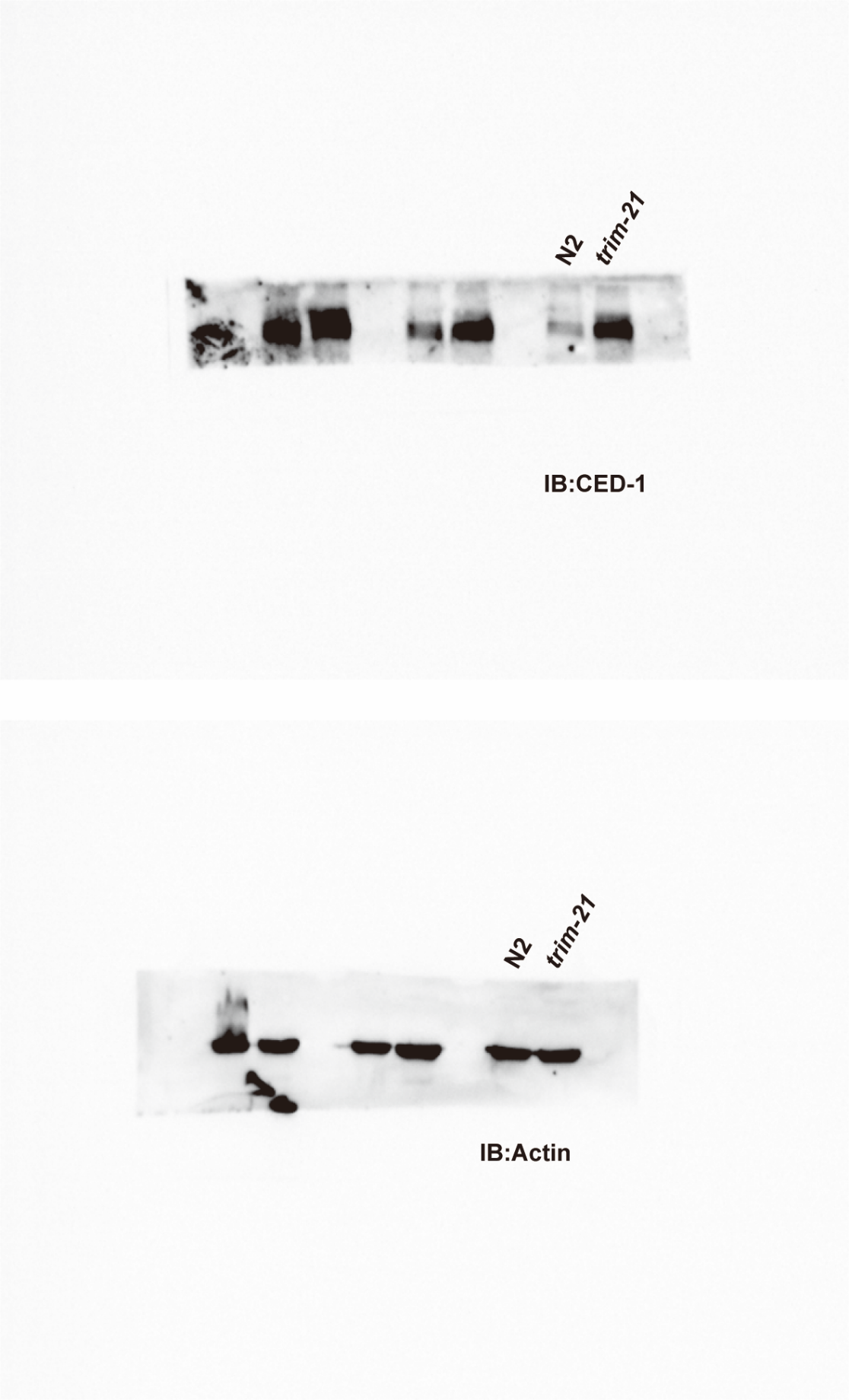


Figure 1L


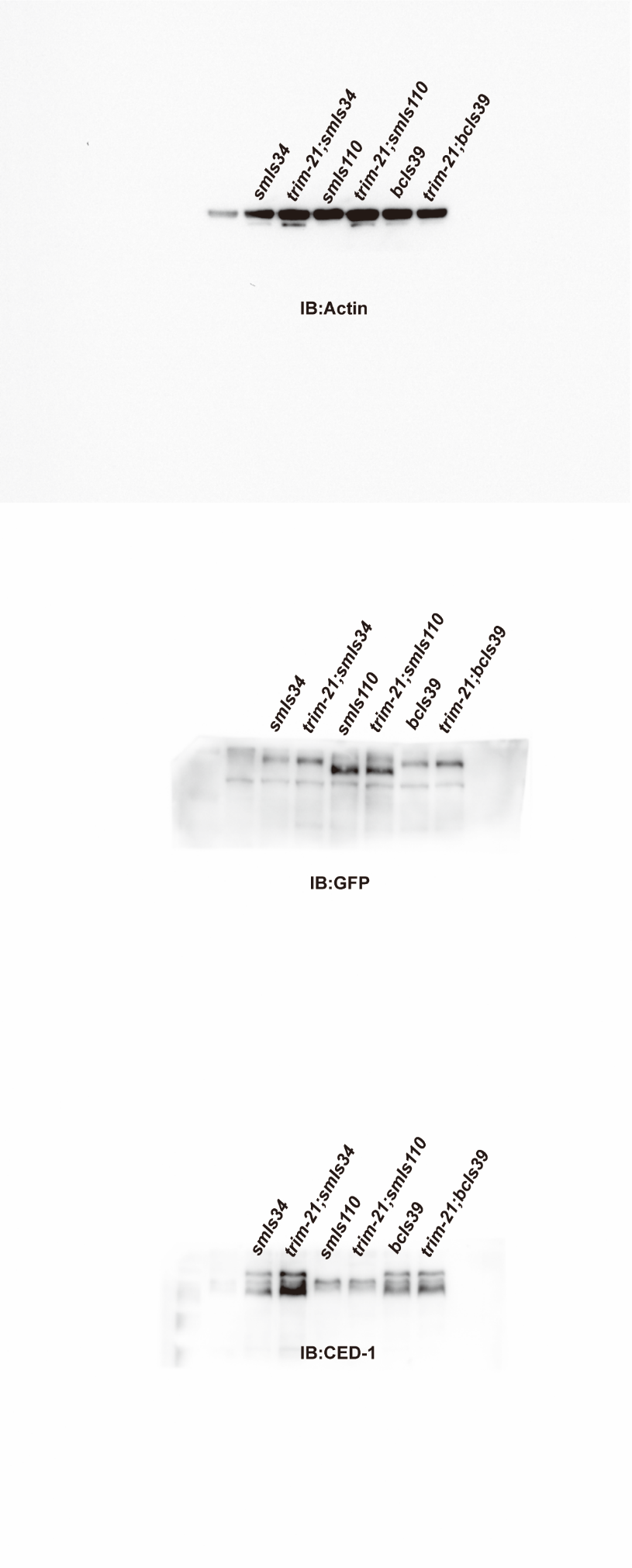


Figure 1M


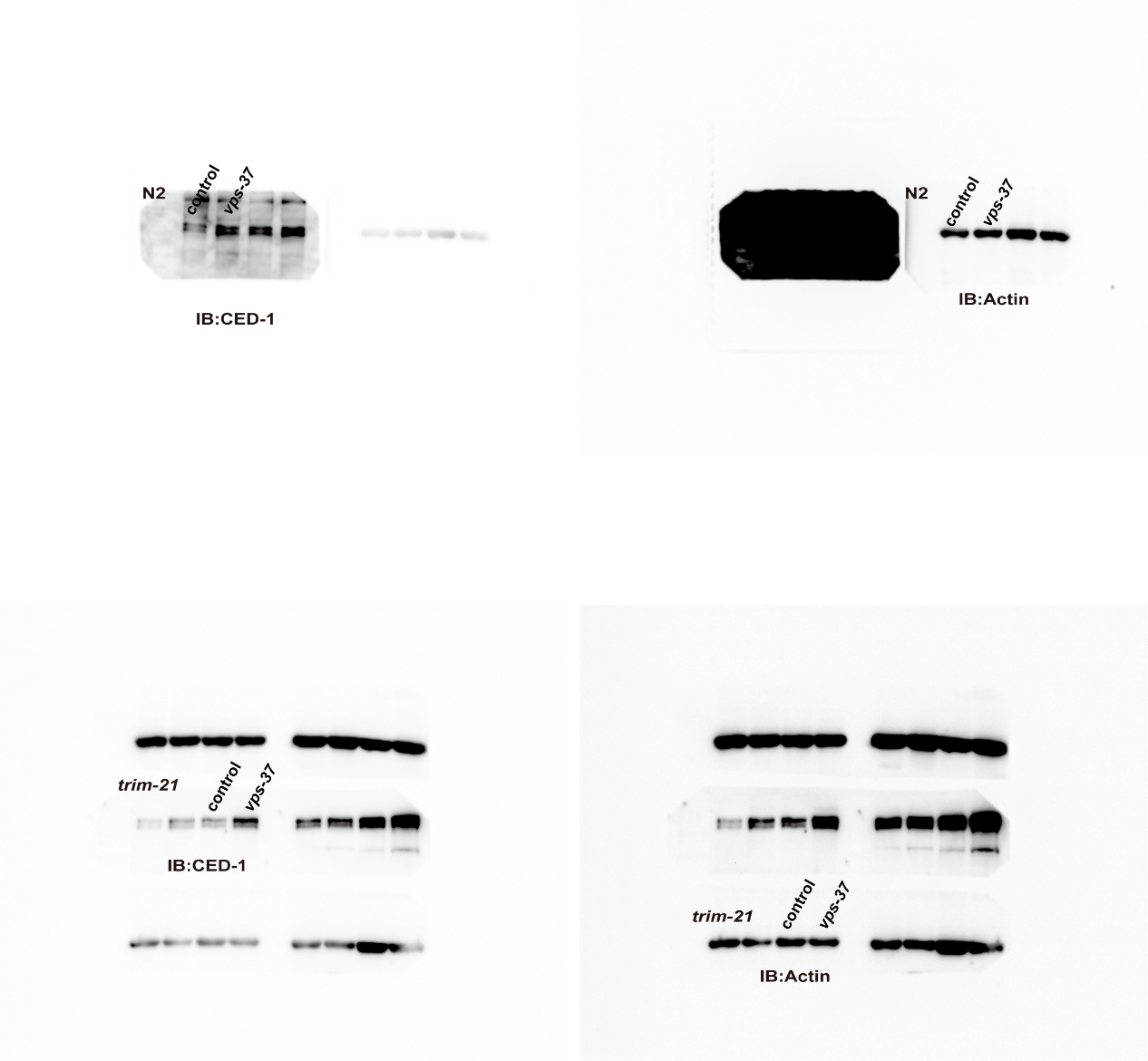

Supplement: Figure 1—source data 1. — Including uncropped Western blot images and raw statistics. [file elife-76436-fig1-data1.zip › Figure 1-Source Data 1/Figure 1 uncroppped blot with relevant bands.docx]

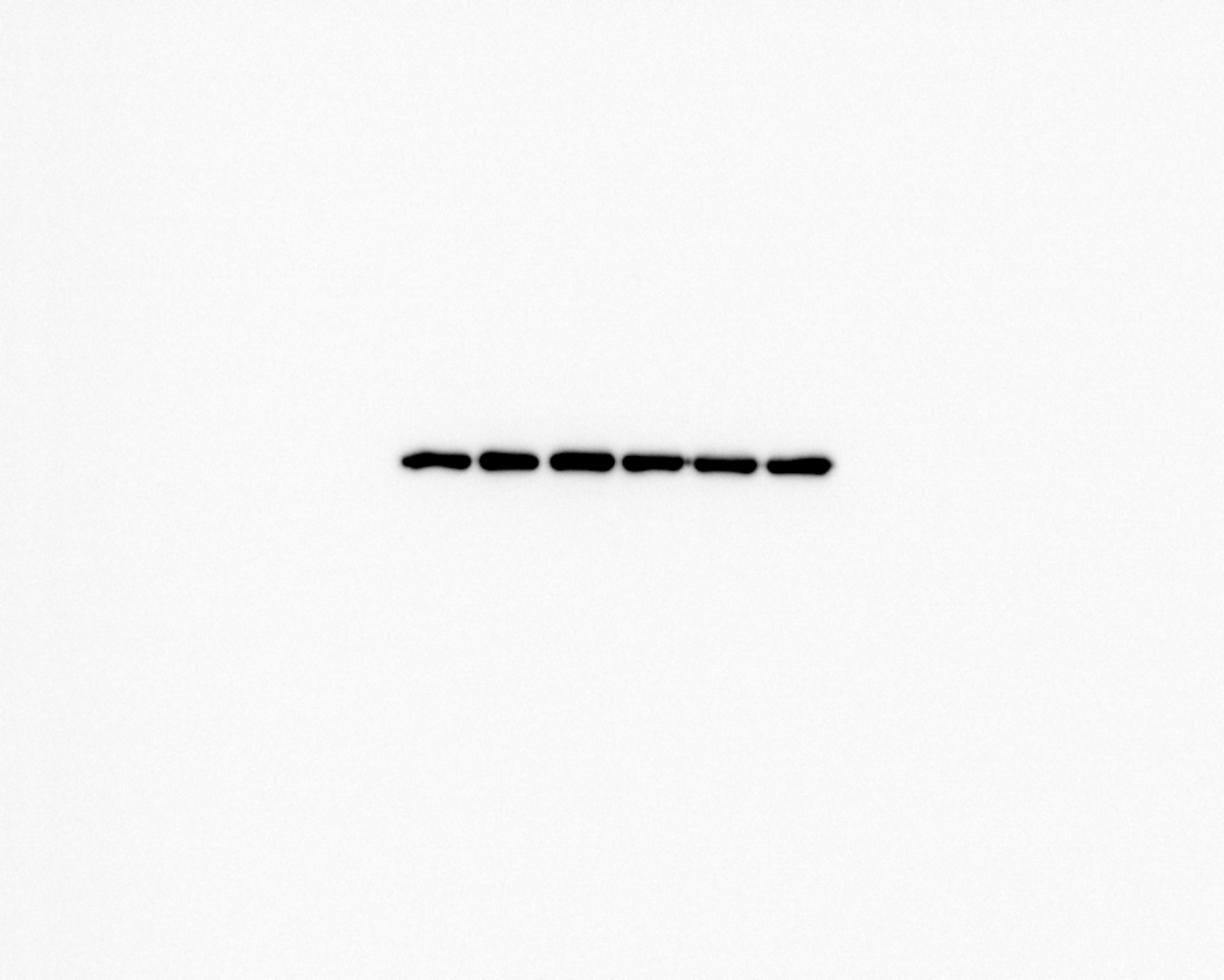

Supplement: Figure 1—source data 1. — Including uncropped Western blot images and raw statistics. [file elife-76436-fig1-data1.zip › Figure 1-Source Data 1/Figure 1A full raw unedited/IB-Actin.tif]

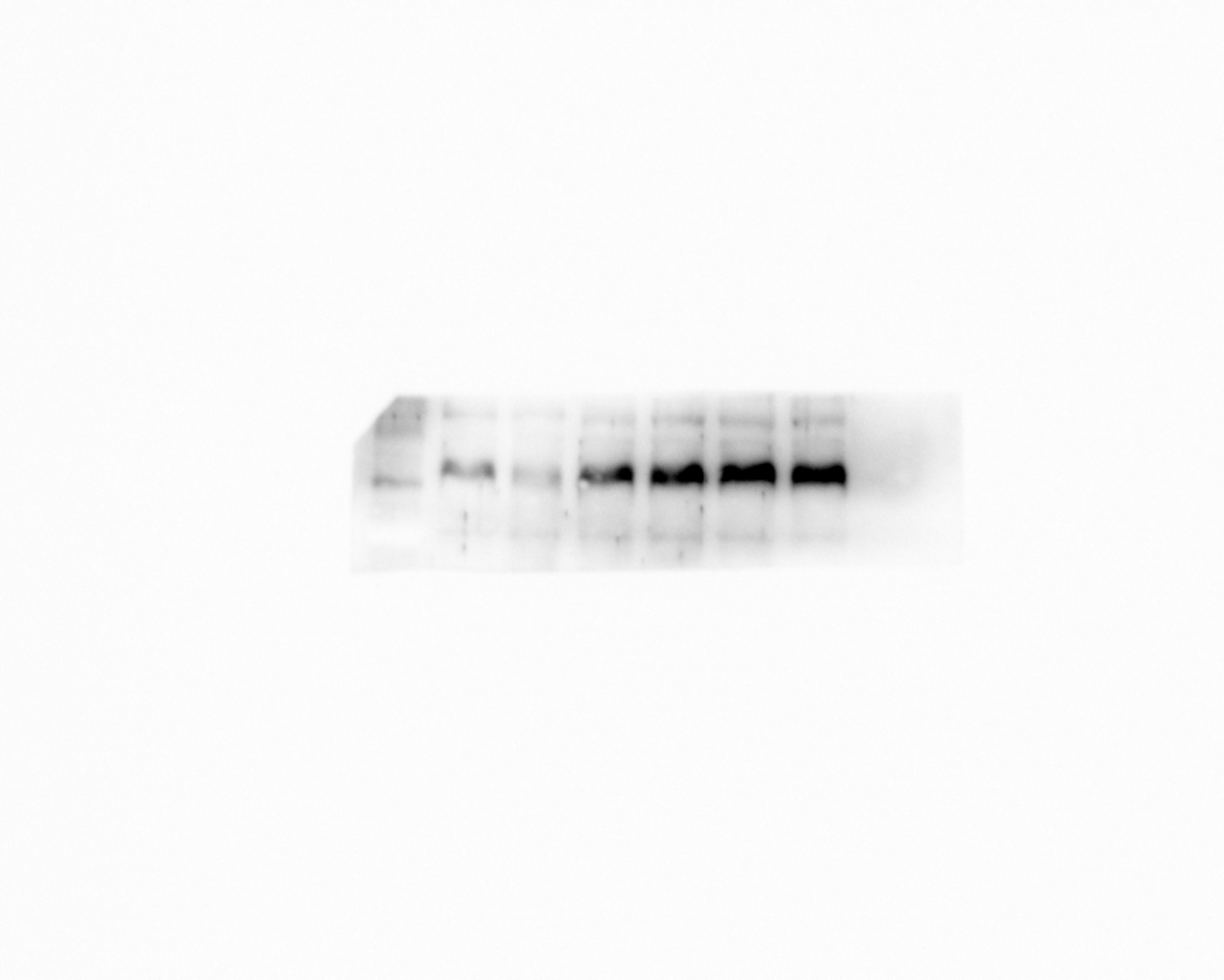

Supplement: Figure 1—source data 1. — Including uncropped Western blot images and raw statistics. [file elife-76436-fig1-data1.zip › Figure 1-Source Data 1/Figure 1A full raw unedited/IB-CED-1.tif]

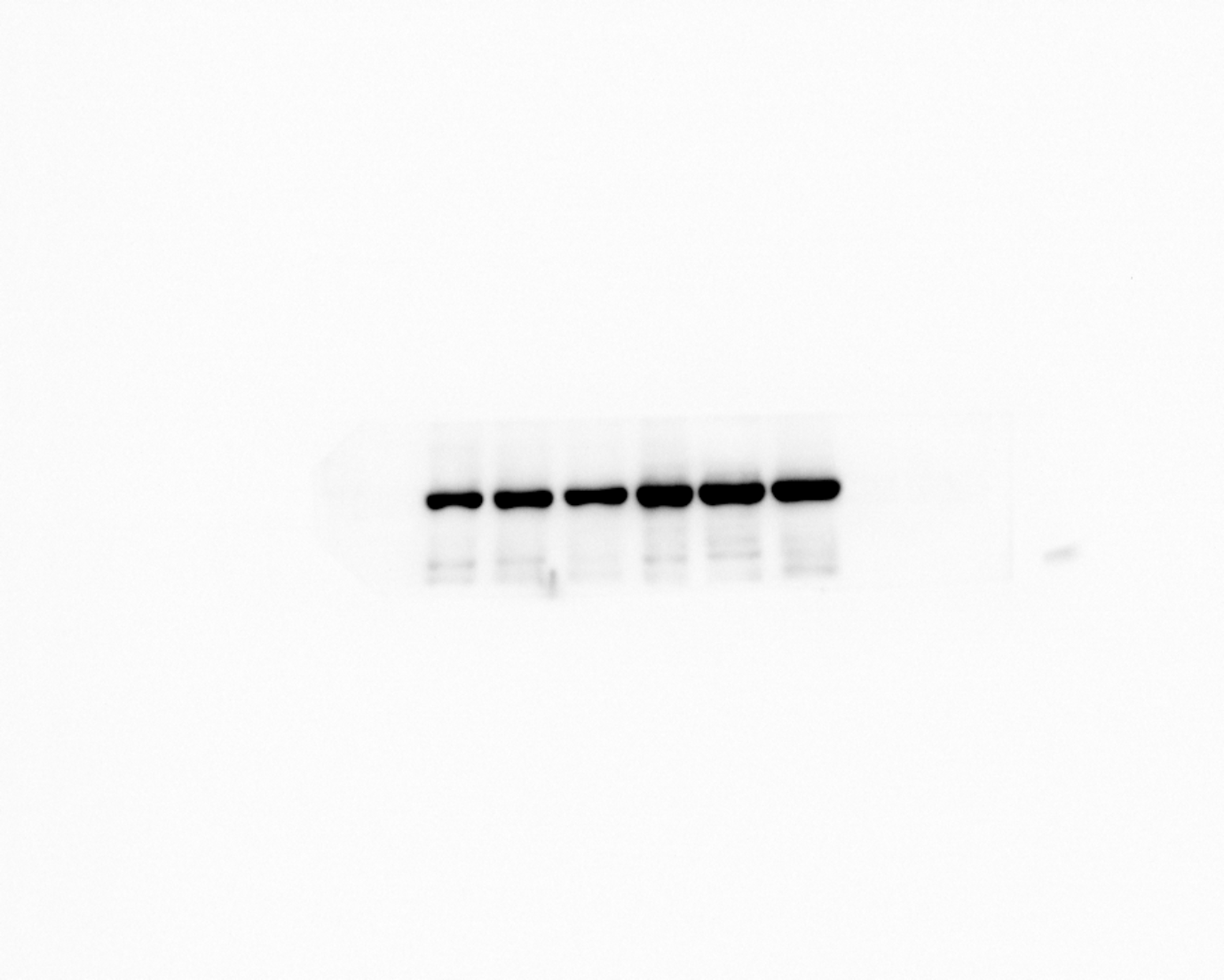

Supplement: Figure 1—source data 1. — Including uncropped Western blot images and raw statistics. [file elife-76436-fig1-data1.zip › Figure 1-Source Data 1/Figure 1B full raw unedited/IB-Actin.tif]

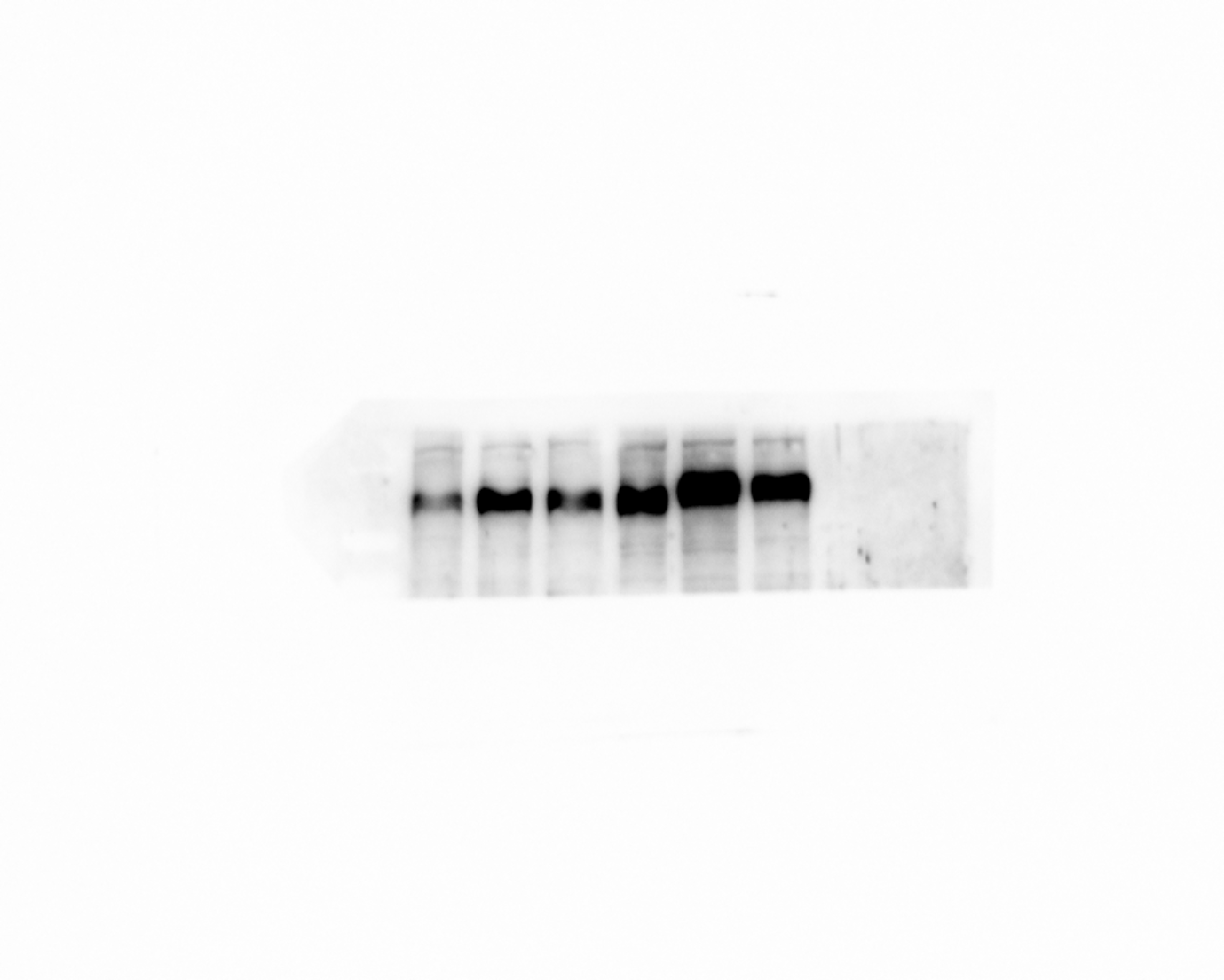

Supplement: Figure 1—source data 1. — Including uncropped Western blot images and raw statistics. [file elife-76436-fig1-data1.zip › Figure 1-Source Data 1/Figure 1B full raw unedited/IB-CED-1.tif]

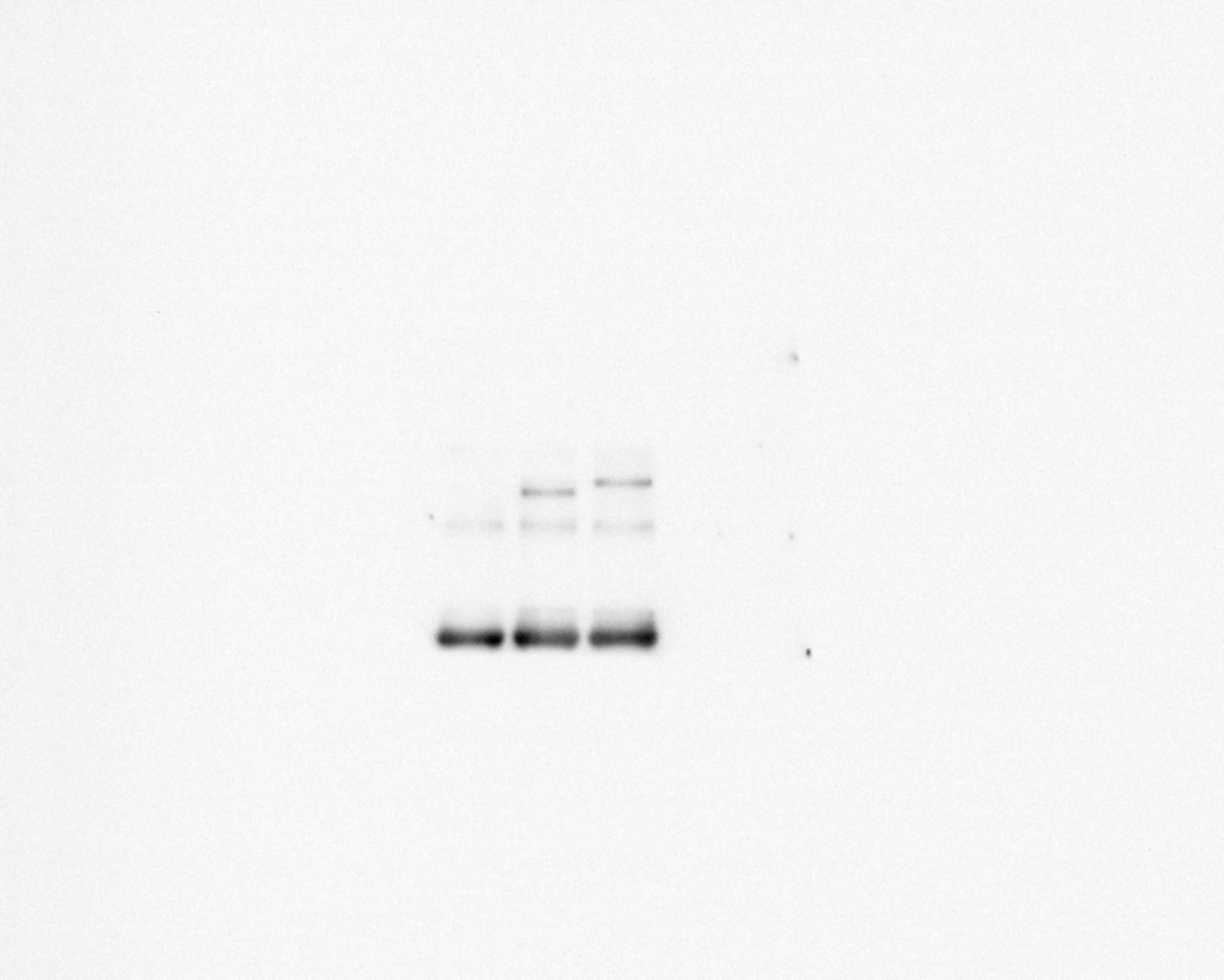

Supplement: Figure 1—source data 1. — Including uncropped Western blot images and raw statistics. [file elife-76436-fig1-data1.zip › Figure 1-Source Data 1/Figure 1C full raw unedited/IB-FLAG.tif]

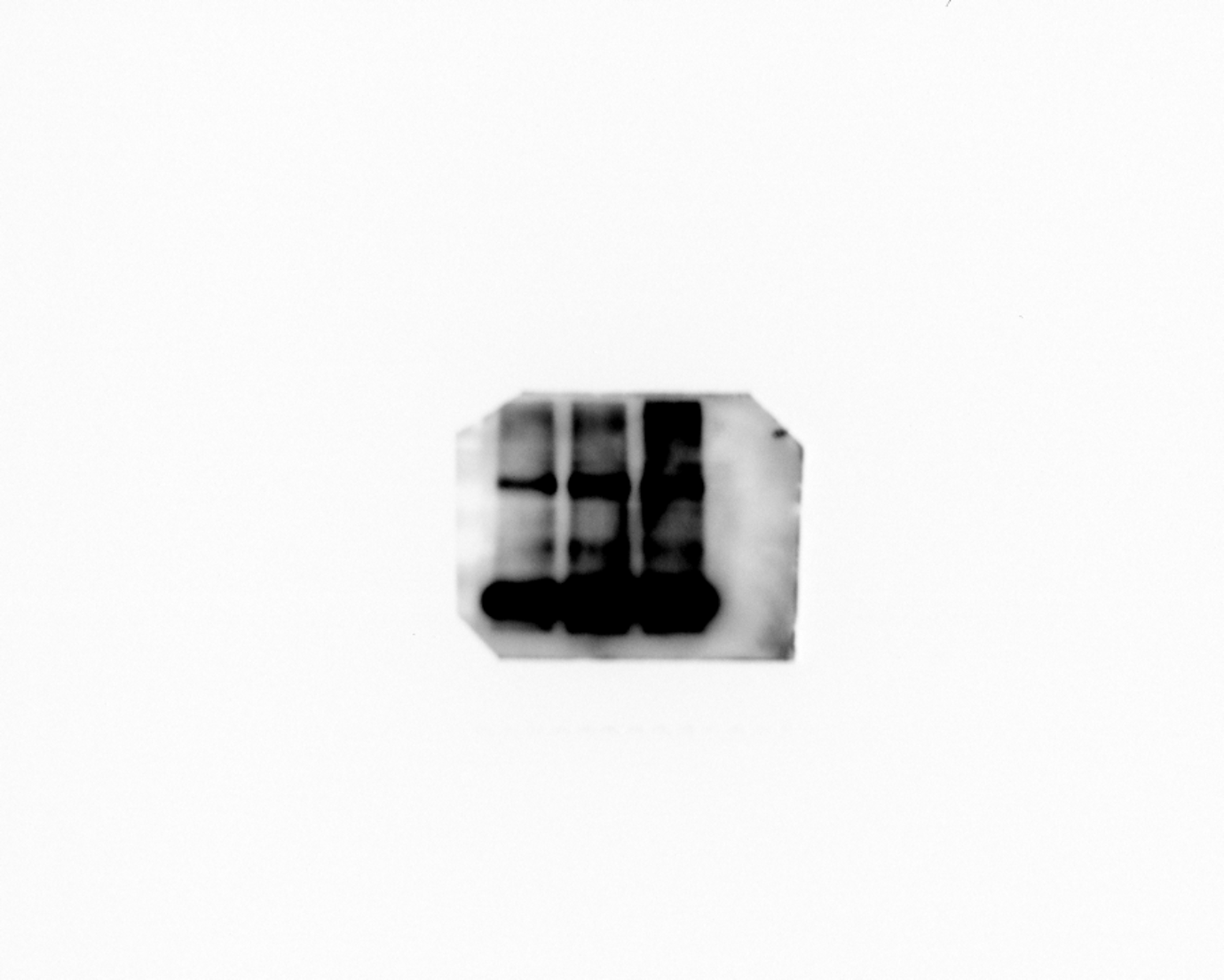

Supplement: Figure 1—source data 1. — Including uncropped Western blot images and raw statistics. [file elife-76436-fig1-data1.zip › Figure 1-Source Data 1/Figure 1C full raw unedited/IB-UB.tif]

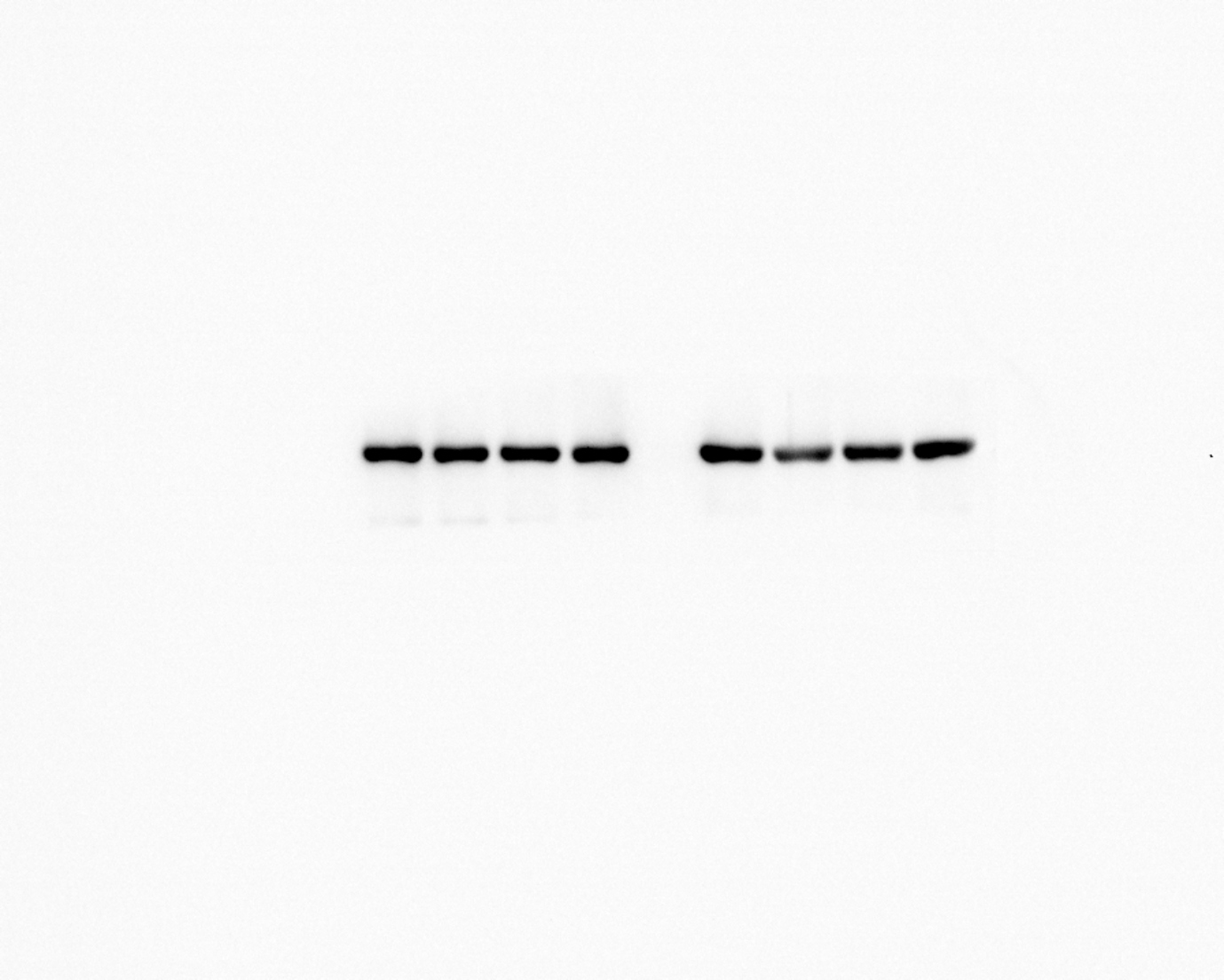

Supplement: Figure 1—source data 1. — Including uncropped Western blot images and raw statistics. [file elife-76436-fig1-data1.zip › Figure 1-Source Data 1/Figure 1D full raw unedited/IB-Actin.tif]

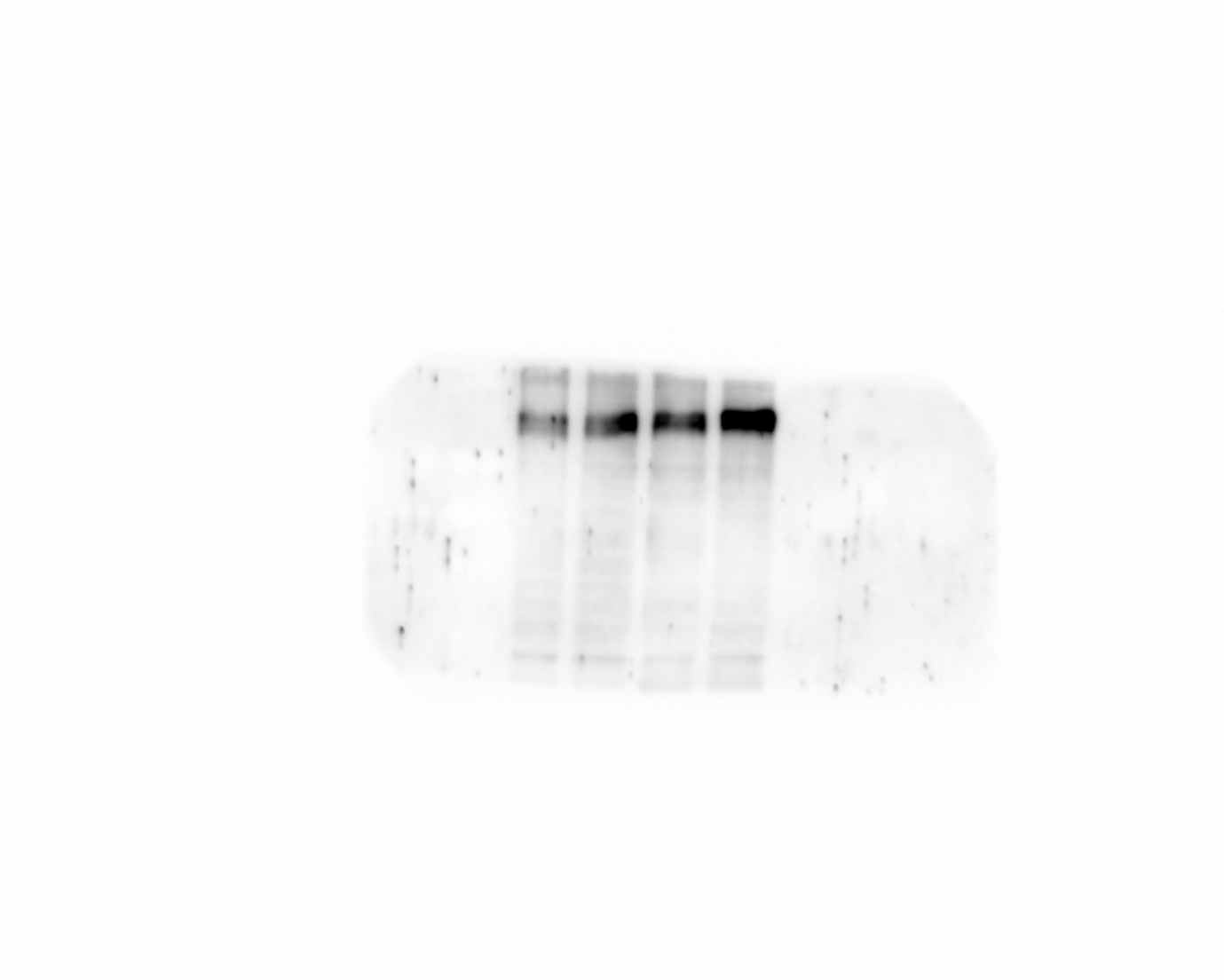

Supplement: Figure 1—source data 1. — Including uncropped Western blot images and raw statistics. [file elife-76436-fig1-data1.zip › Figure 1-Source Data 1/Figure 1D full raw unedited/IB-CED-1.tif]

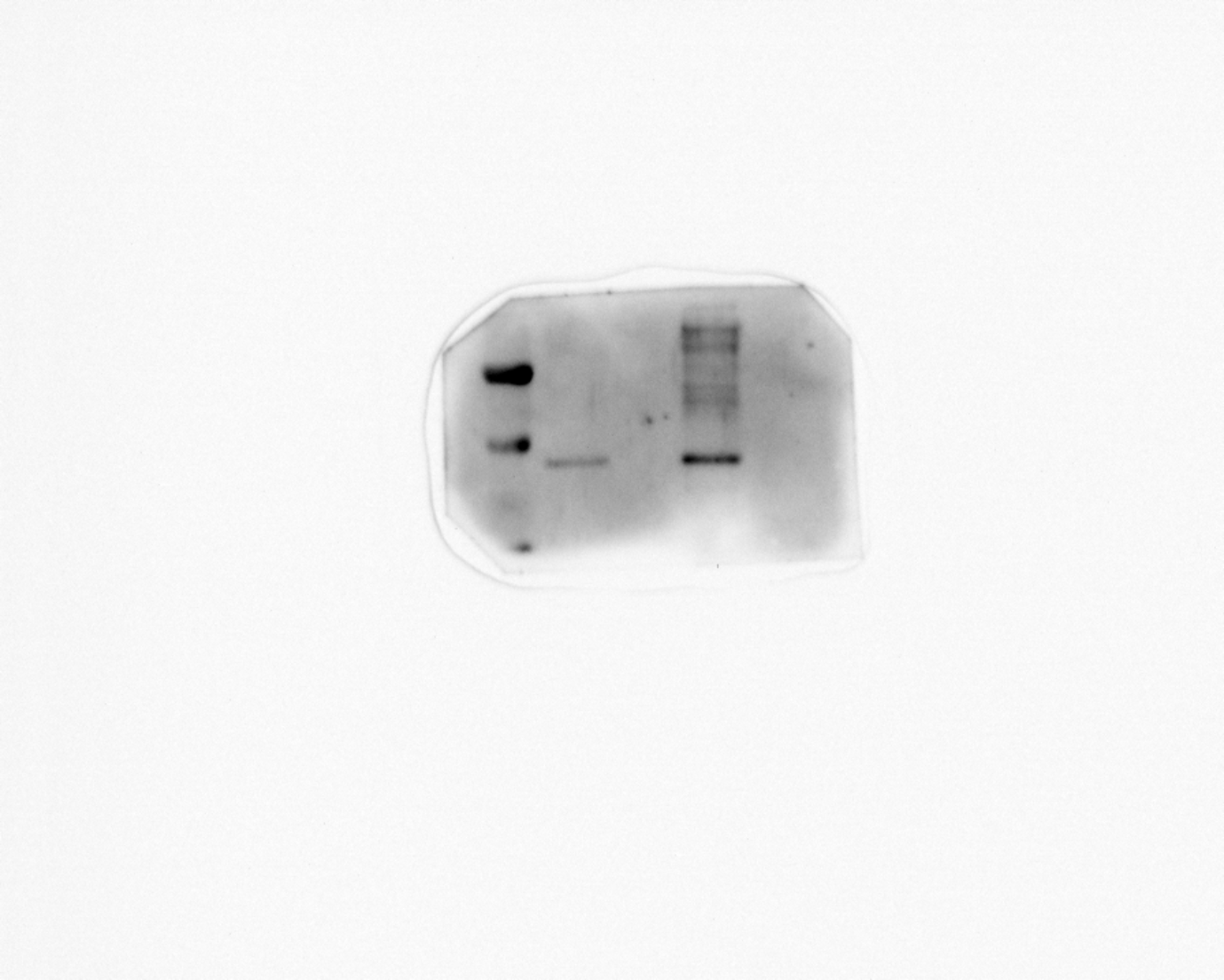

Supplement: Figure 1—source data 1. — Including uncropped Western blot images and raw statistics. [file elife-76436-fig1-data1.zip › Figure 1-Source Data 1/Figure 1F full raw unedited/IB-FLAG.tif]

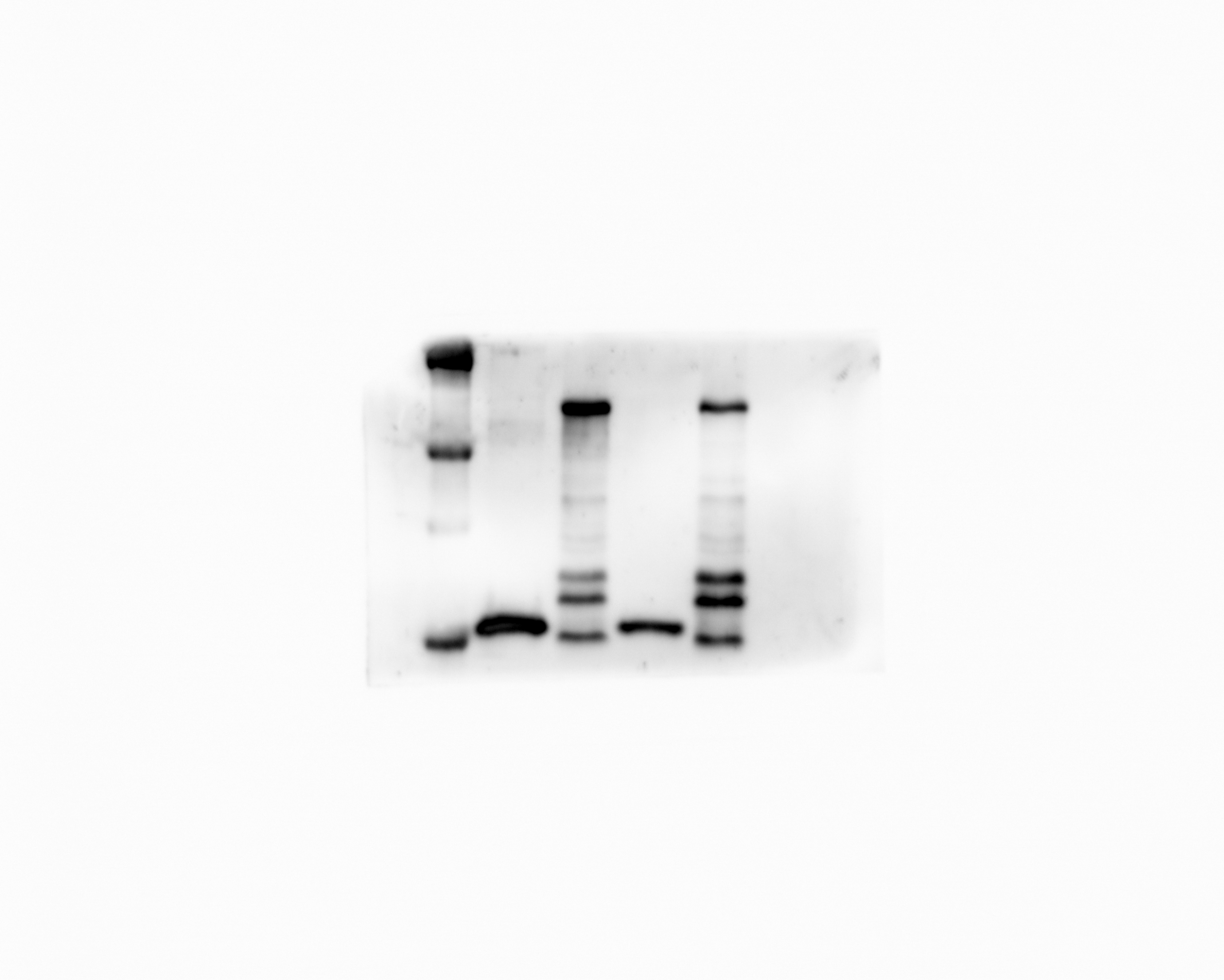

Supplement: Figure 1—source data 1. — Including uncropped Western blot images and raw statistics. [file elife-76436-fig1-data1.zip › Figure 1-Source Data 1/Figure 1F full raw unedited/IB-GST.tif]

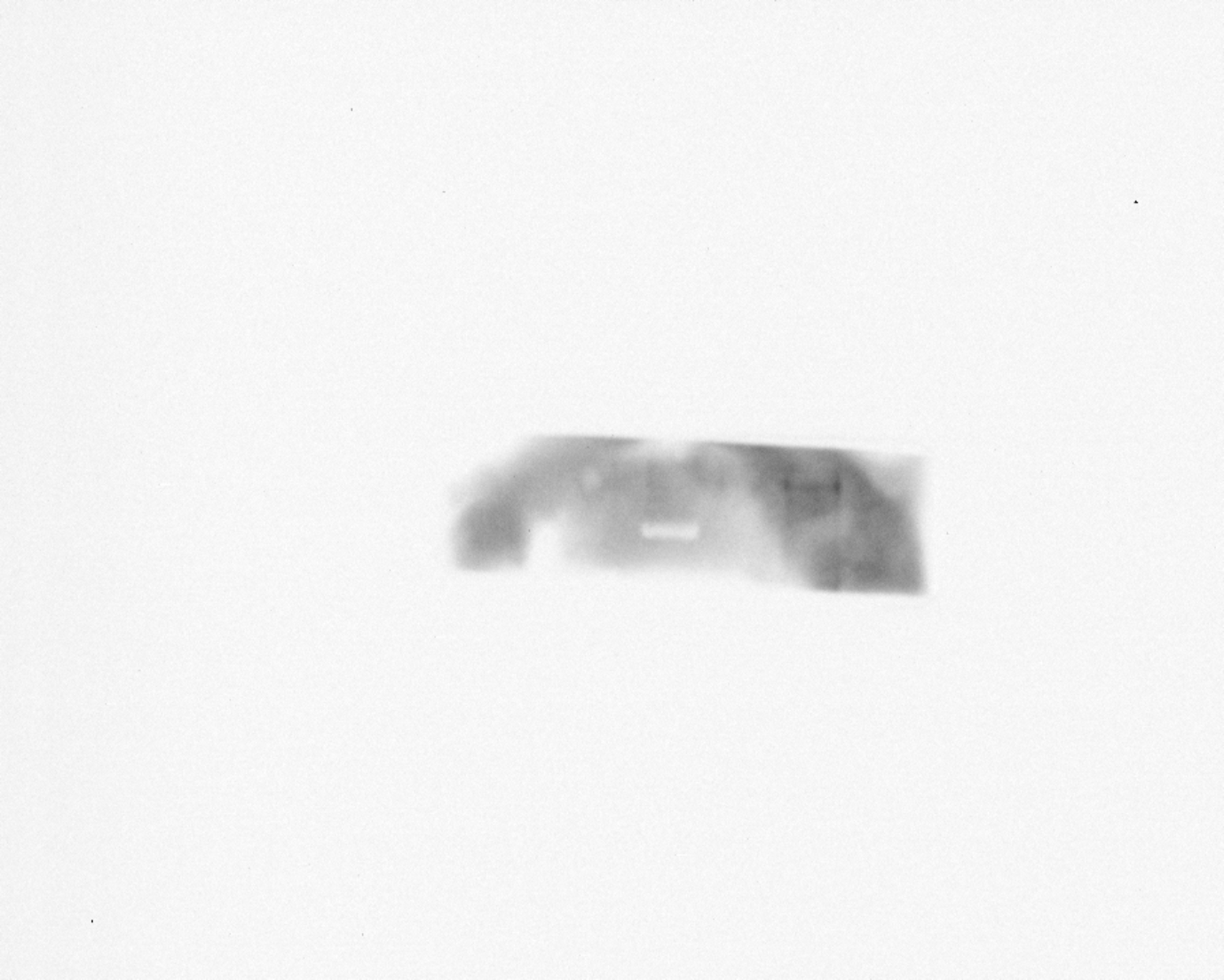

Supplement: Figure 1—source data 1. — Including uncropped Western blot images and raw statistics. [file elife-76436-fig1-data1.zip › Figure 1-Source Data 1/Figure 1G full raw unedited/IB-CED-1.tif]

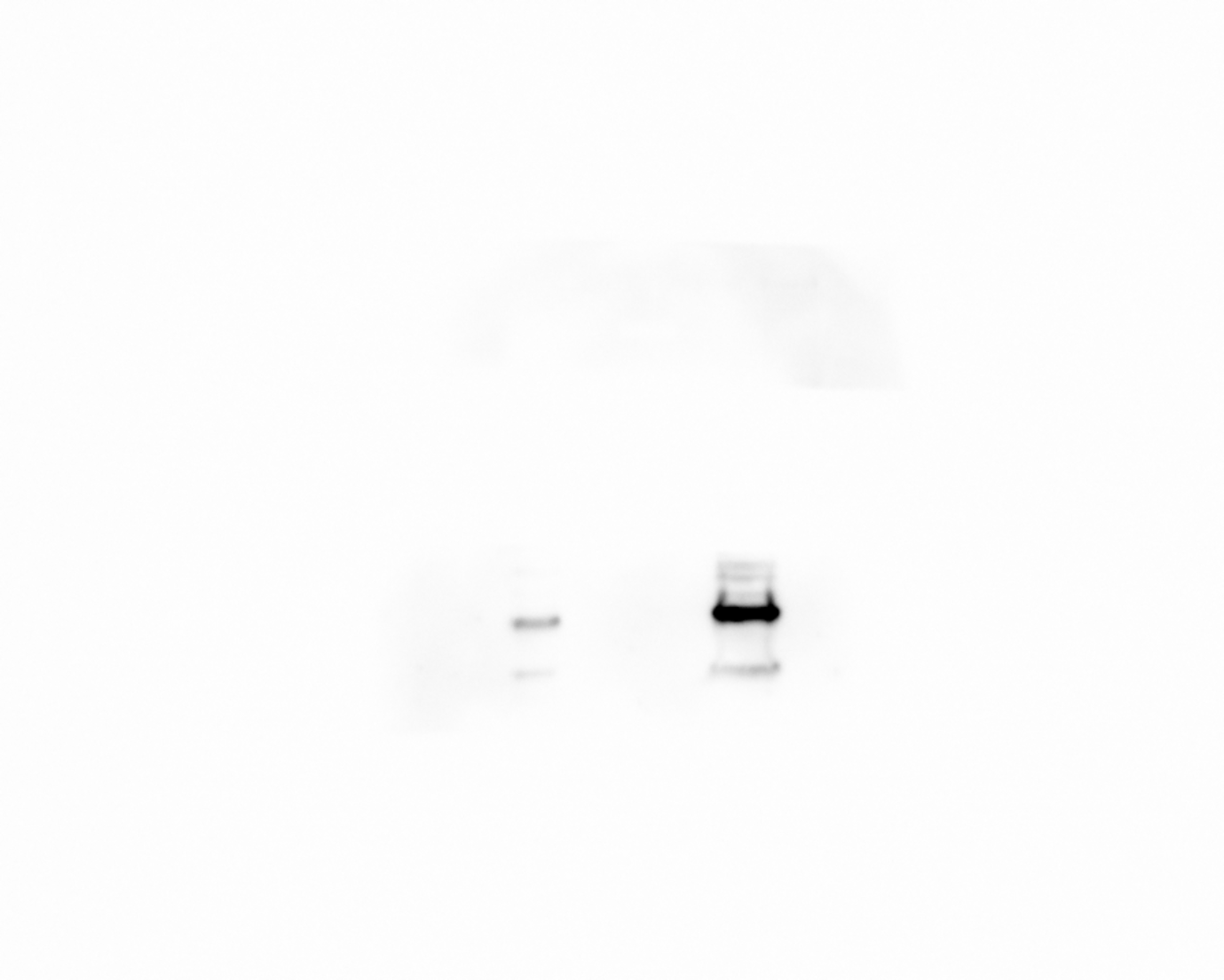

Supplement: Figure 1—source data 1. — Including uncropped Western blot images and raw statistics. [file elife-76436-fig1-data1.zip › Figure 1-Source Data 1/Figure 1G full raw unedited/IB-FLAG.tif]

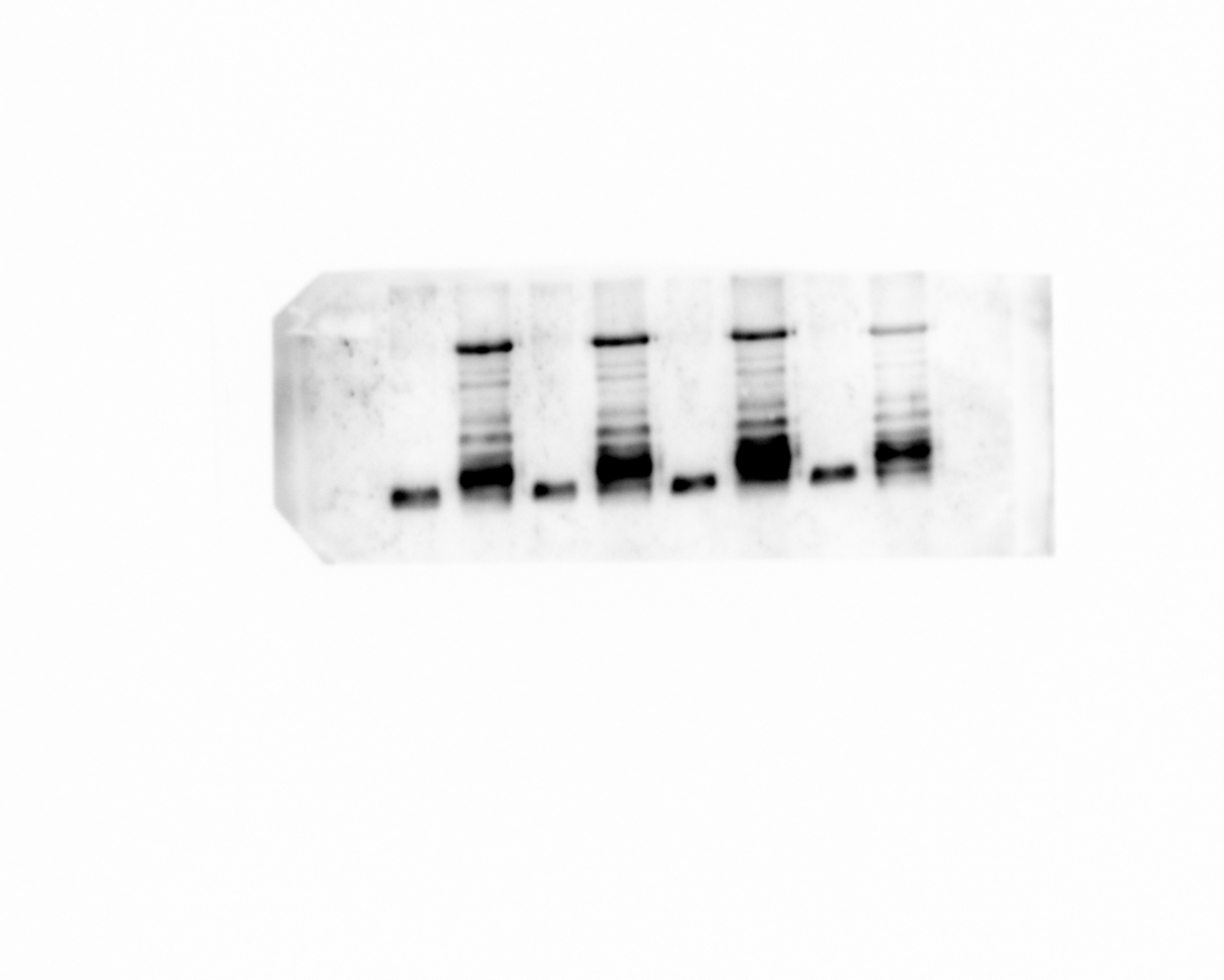

Supplement: Figure 1—source data 1. — Including uncropped Western blot images and raw statistics. [file elife-76436-fig1-data1.zip › Figure 1-Source Data 1/Figure 1H full raw unedited/IB-GST.tif]

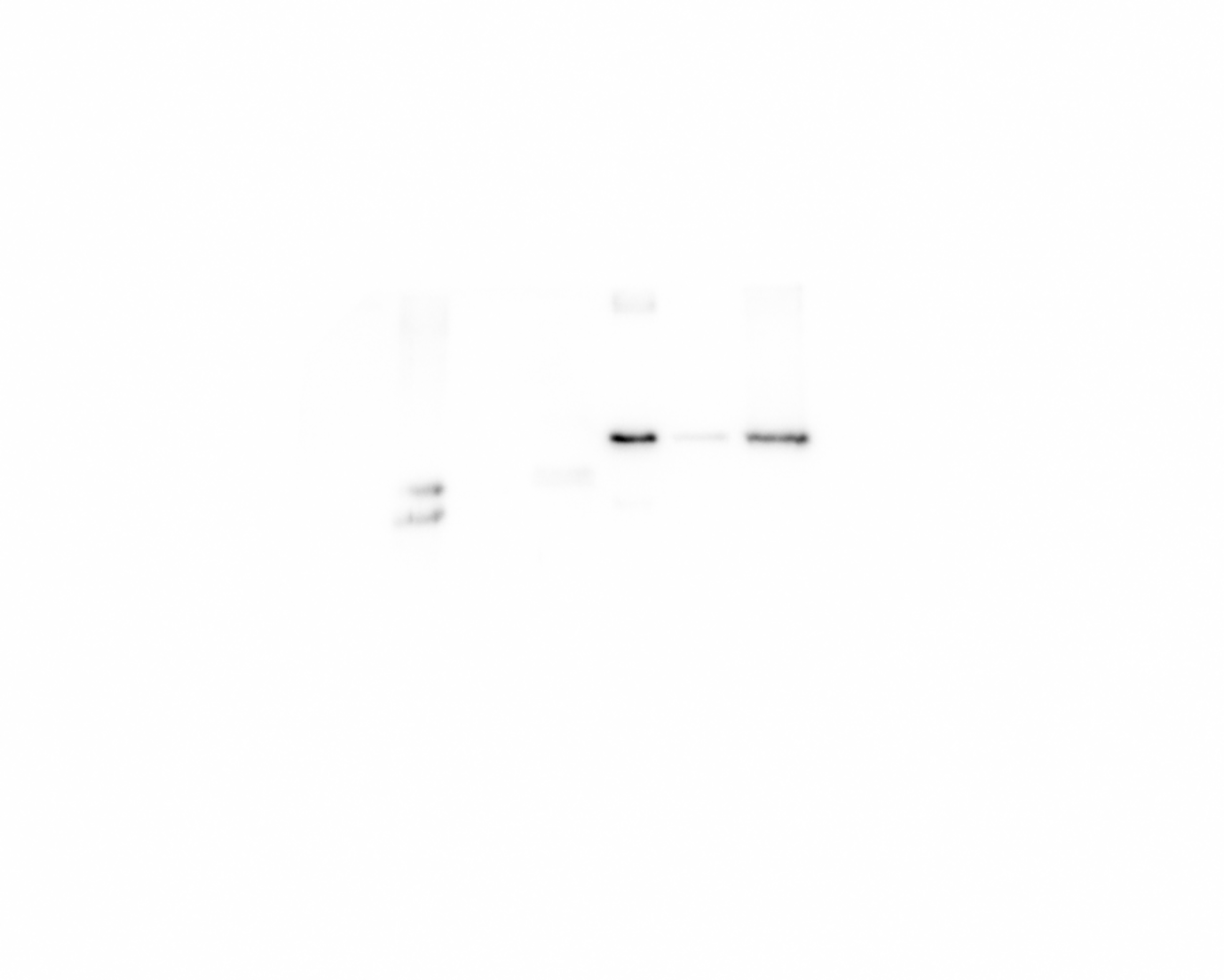

Supplement: Figure 1—source data 1. — Including uncropped Western blot images and raw statistics. [file elife-76436-fig1-data1.zip › Figure 1-Source Data 1/Figure 1H full raw unedited/IB-MYC-TRIM-21-CT.tif]

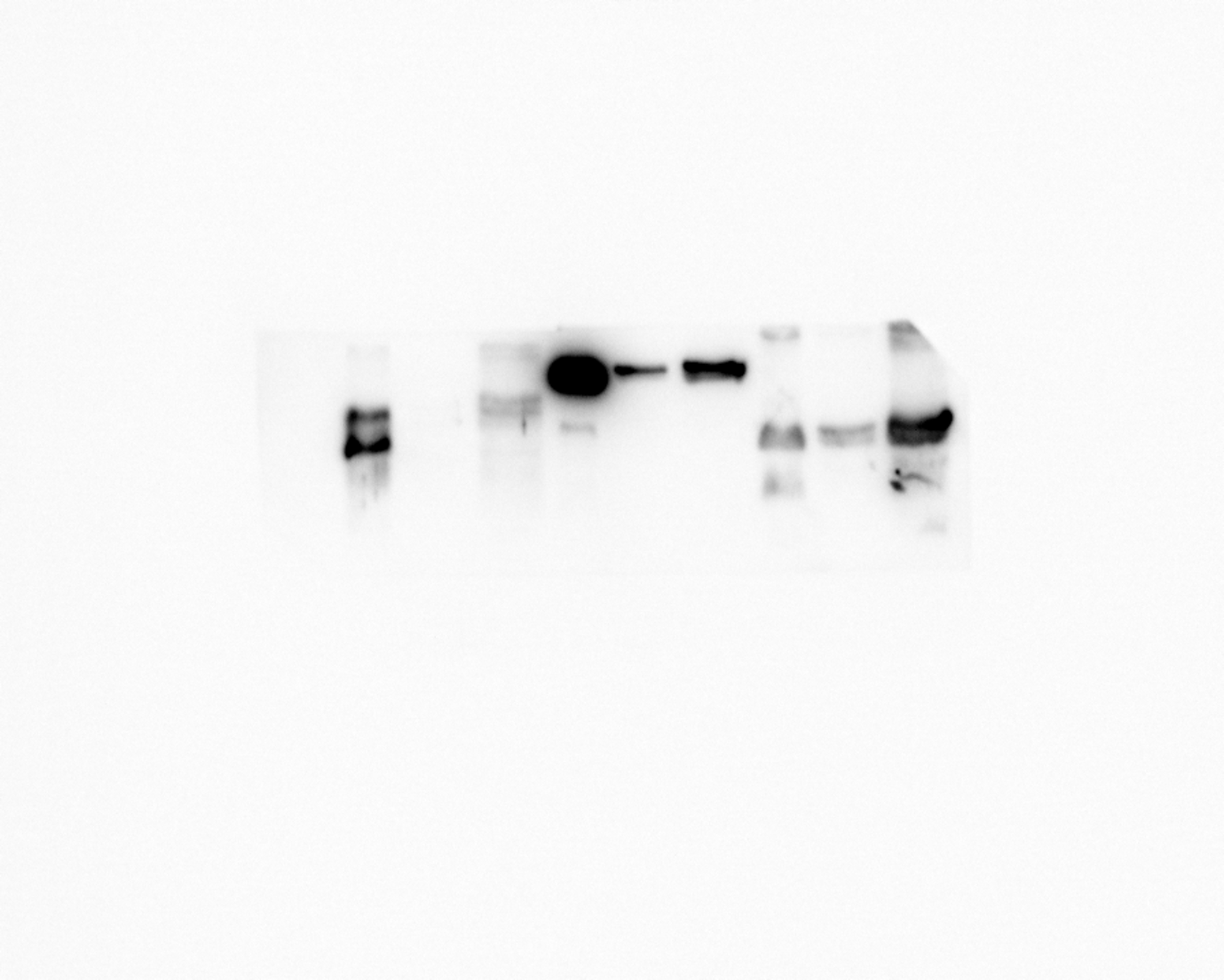

Supplement: Figure 1—source data 1. — Including uncropped Western blot images and raw statistics. [file elife-76436-fig1-data1.zip › Figure 1-Source Data 1/Figure 1H full raw unedited/IB-MYC-TRIM-21-NT.tif]

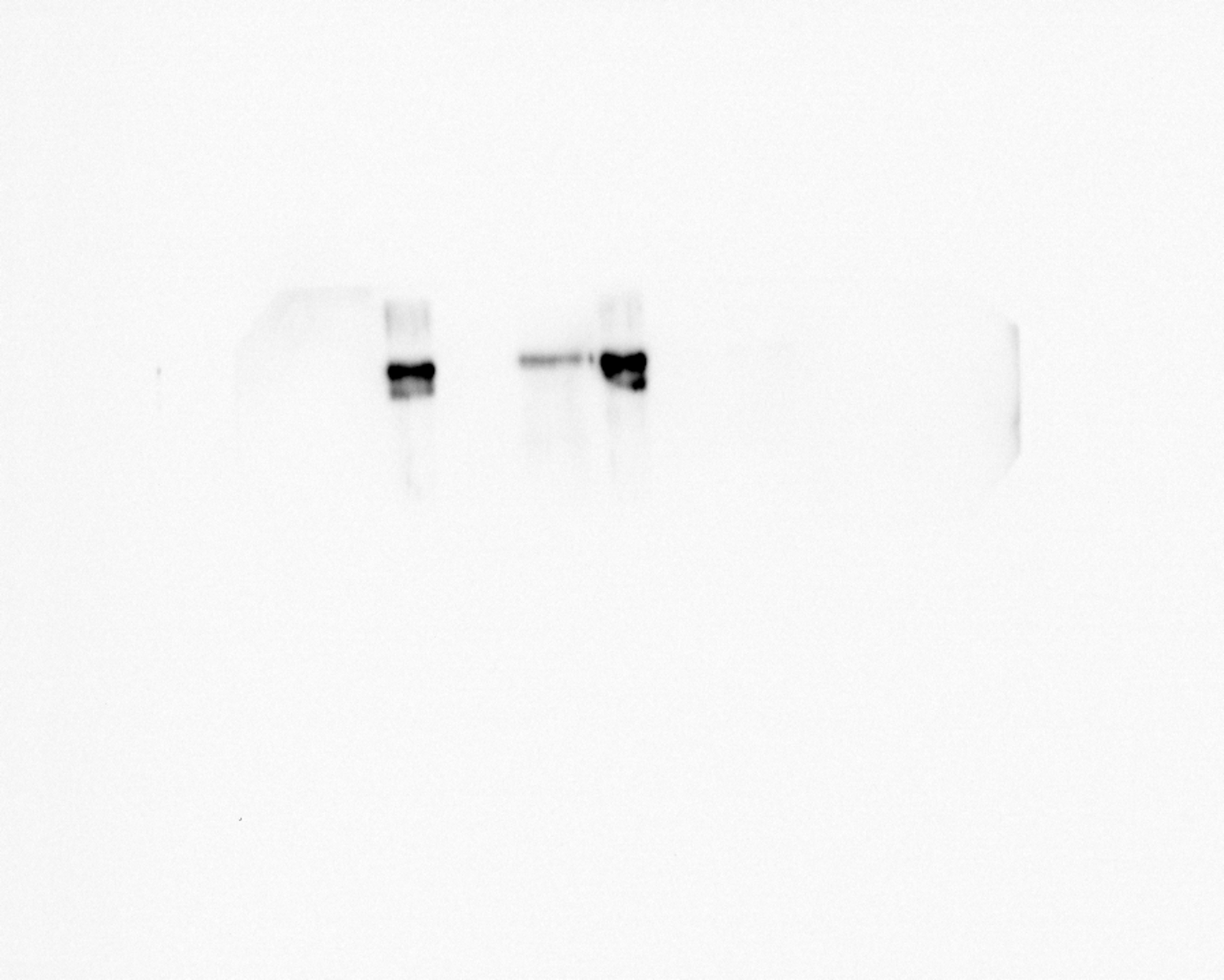

Supplement: Figure 1—source data 1. — Including uncropped Western blot images and raw statistics. [file elife-76436-fig1-data1.zip › Figure 1-Source Data 1/Figure 1H full raw unedited/IB-MYC-TRIM-21.tif]

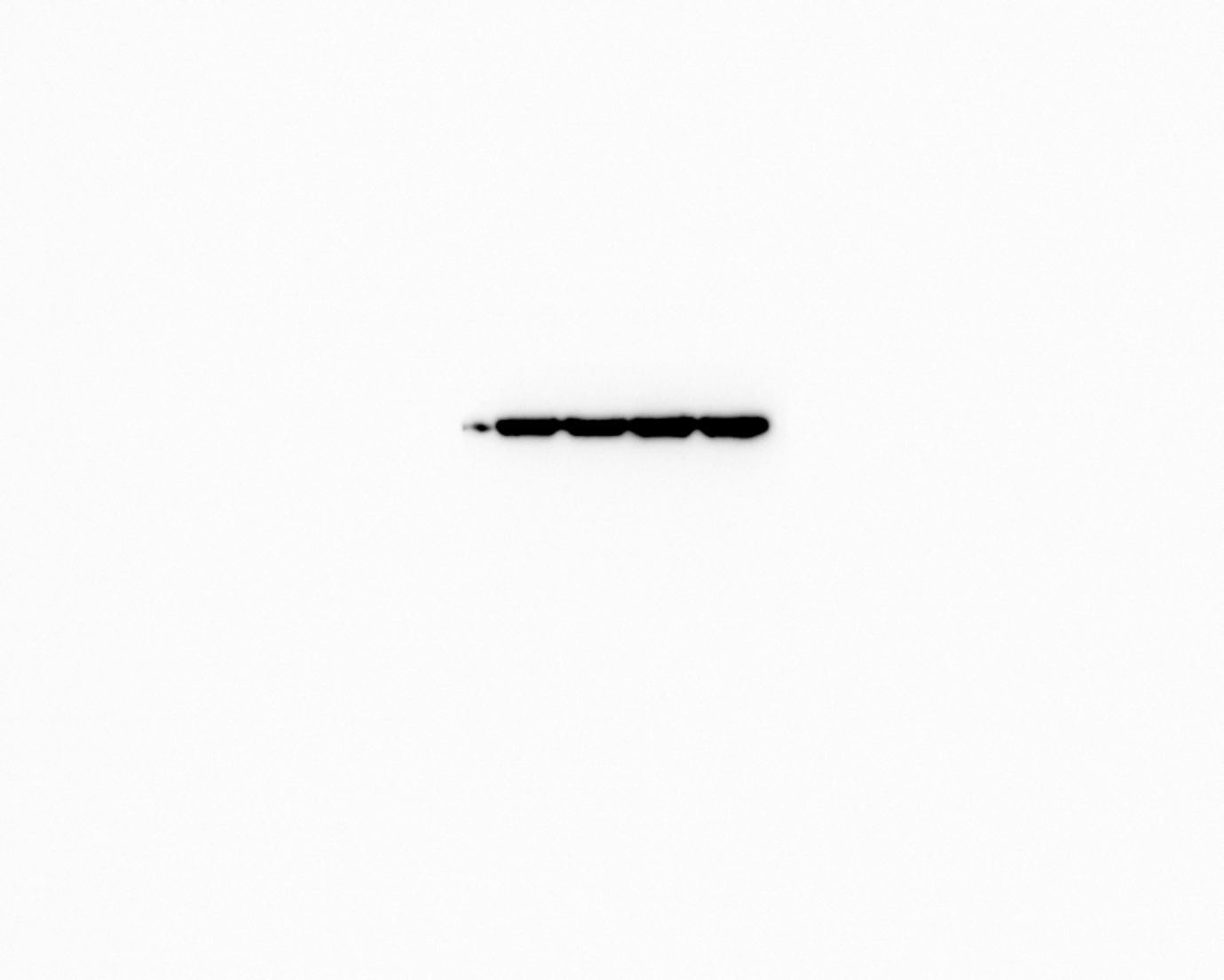

Supplement: Figure 1—source data 1. — Including uncropped Western blot images and raw statistics. [file elife-76436-fig1-data1.zip › Figure 1-Source Data 1/Figure 1I full raw unedited/Input-IB-Actin.tif]

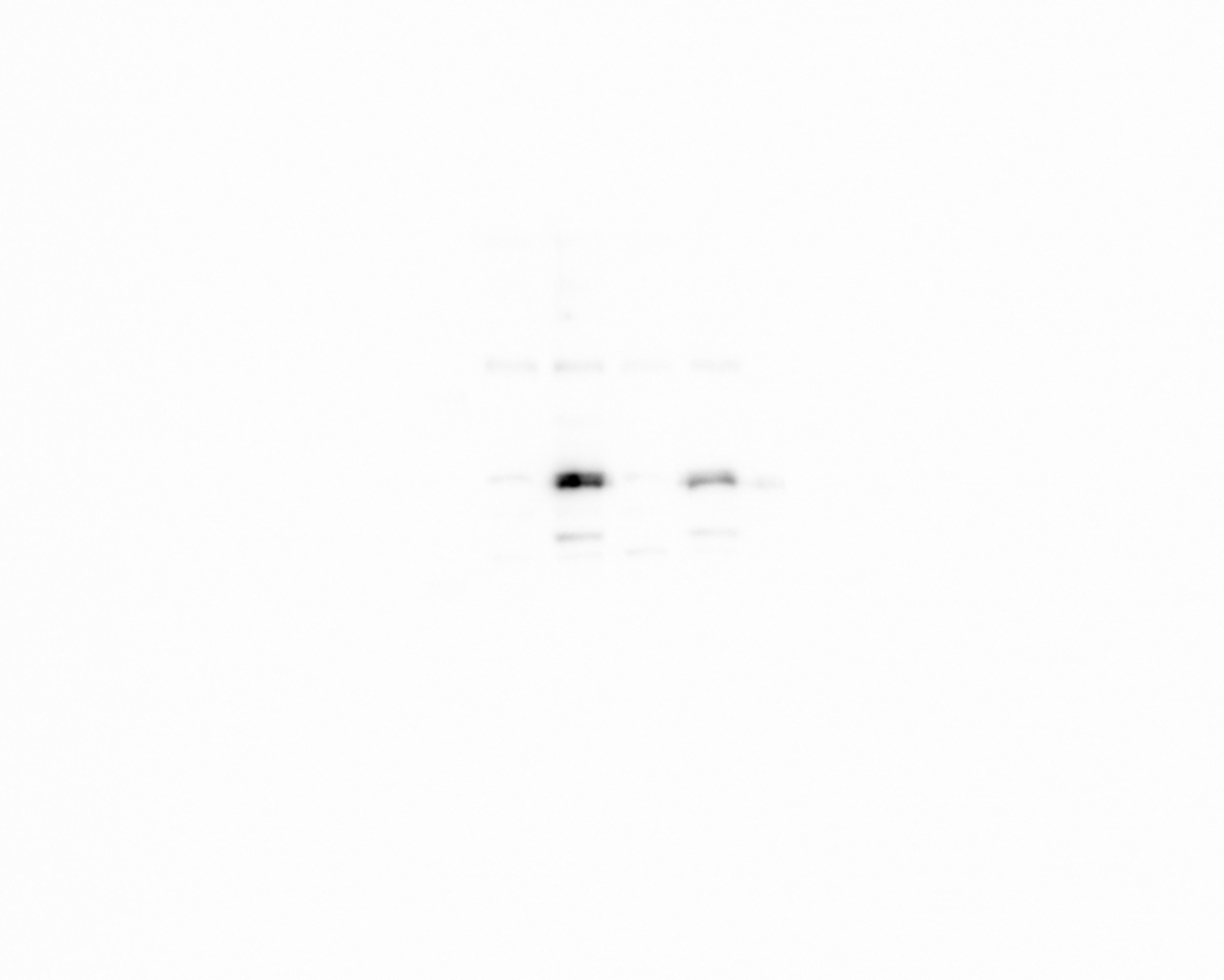

Supplement: Figure 1—source data 1. — Including uncropped Western blot images and raw statistics. [file elife-76436-fig1-data1.zip › Figure 1-Source Data 1/Figure 1I full raw unedited/Input-IB-FLAG.tif]

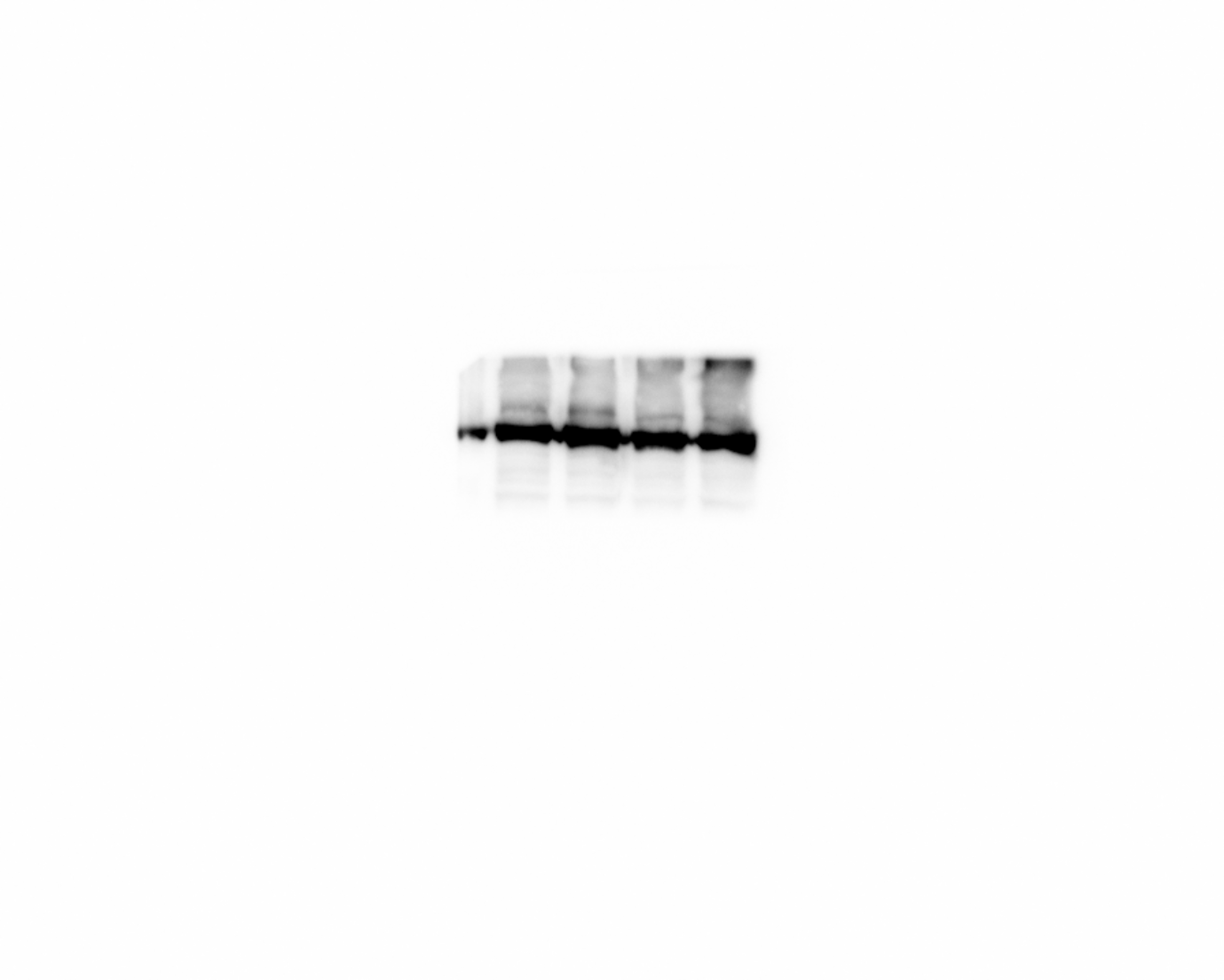

Supplement: Figure 1—source data 1. — Including uncropped Western blot images and raw statistics. [file elife-76436-fig1-data1.zip › Figure 1-Source Data 1/Figure 1I full raw unedited/Input-IB-GFP.tif]

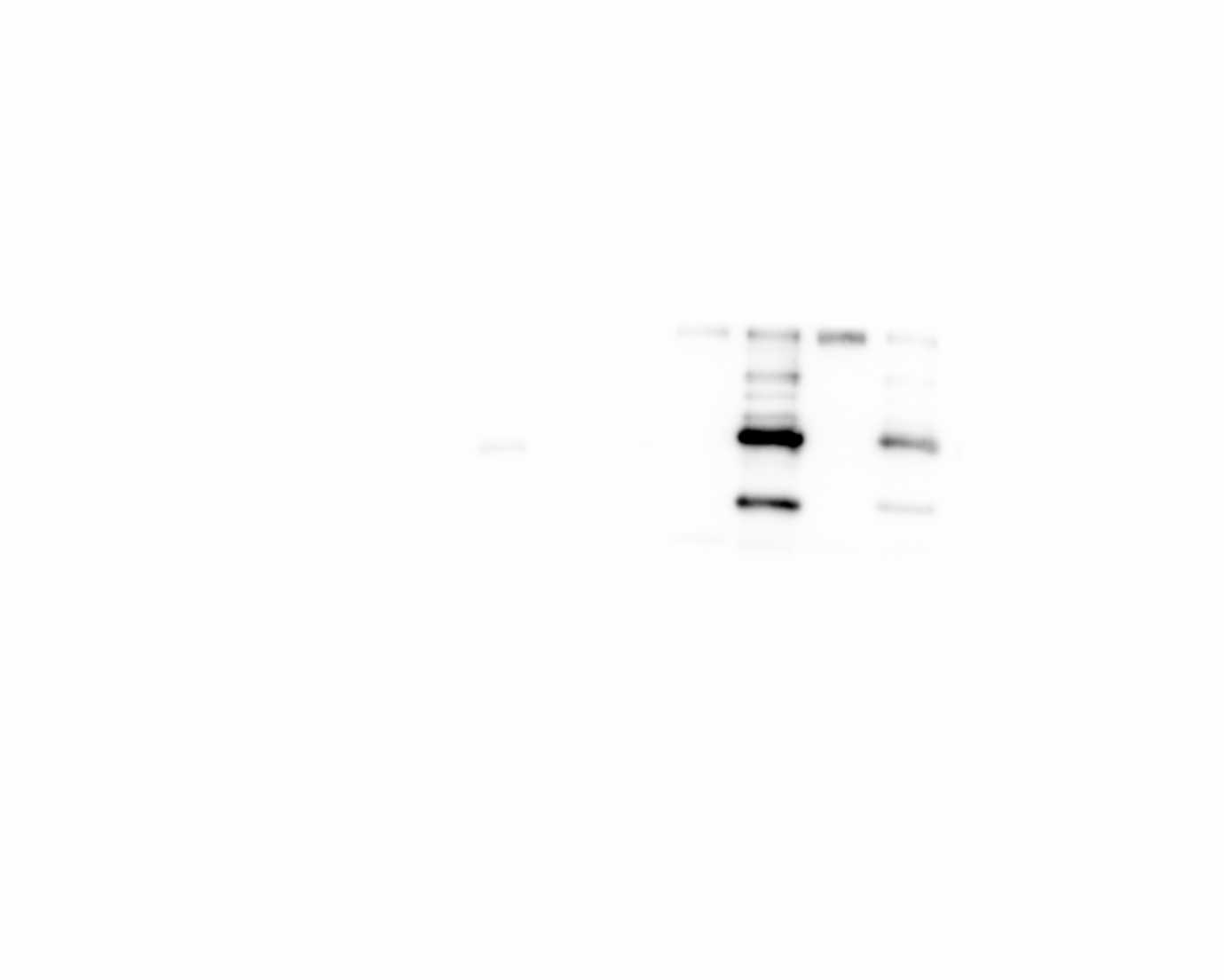

Supplement: Figure 1—source data 1. — Including uncropped Western blot images and raw statistics. [file elife-76436-fig1-data1.zip › Figure 1-Source Data 1/Figure 1I full raw unedited/IP-IB-FLAG-Left.tif]

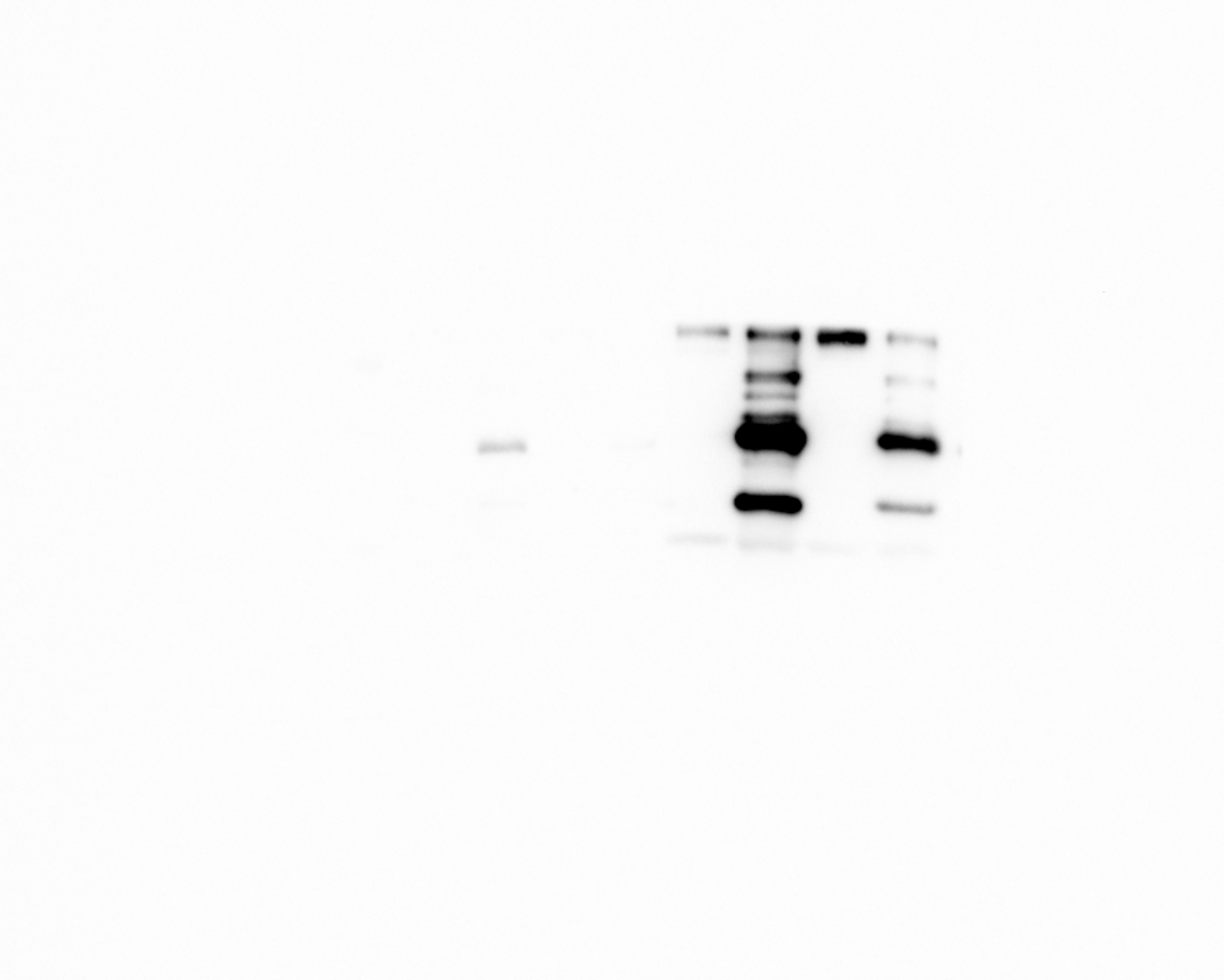

Supplement: Figure 1—source data 1. — Including uncropped Western blot images and raw statistics. [file elife-76436-fig1-data1.zip › Figure 1-Source Data 1/Figure 1I full raw unedited/IP-IB-FLAG-Right.tif]

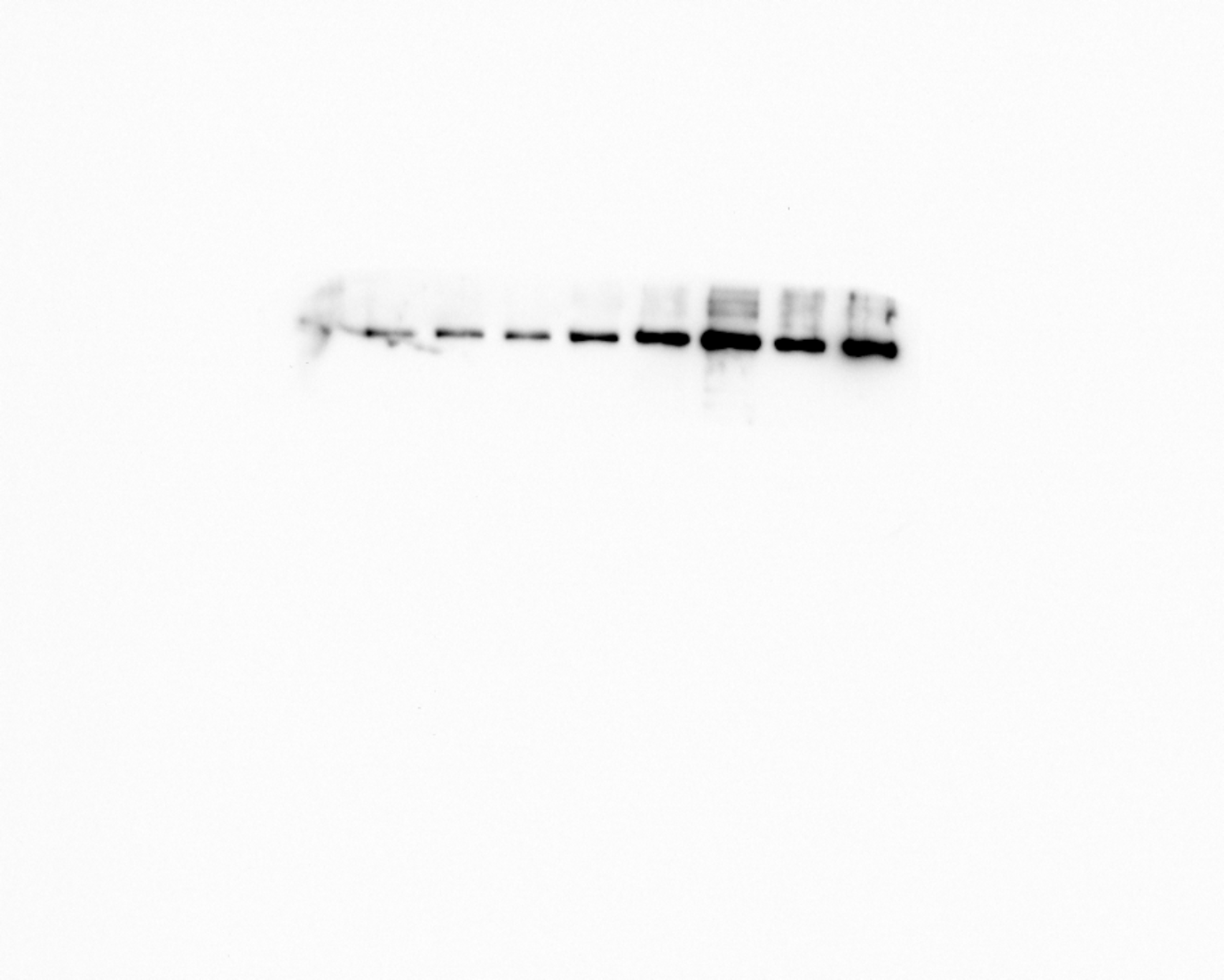

Supplement: Figure 1—source data 1. — Including uncropped Western blot images and raw statistics. [file elife-76436-fig1-data1.zip › Figure 1-Source Data 1/Figure 1I full raw unedited/IP-IB-GFP-Left.tif]

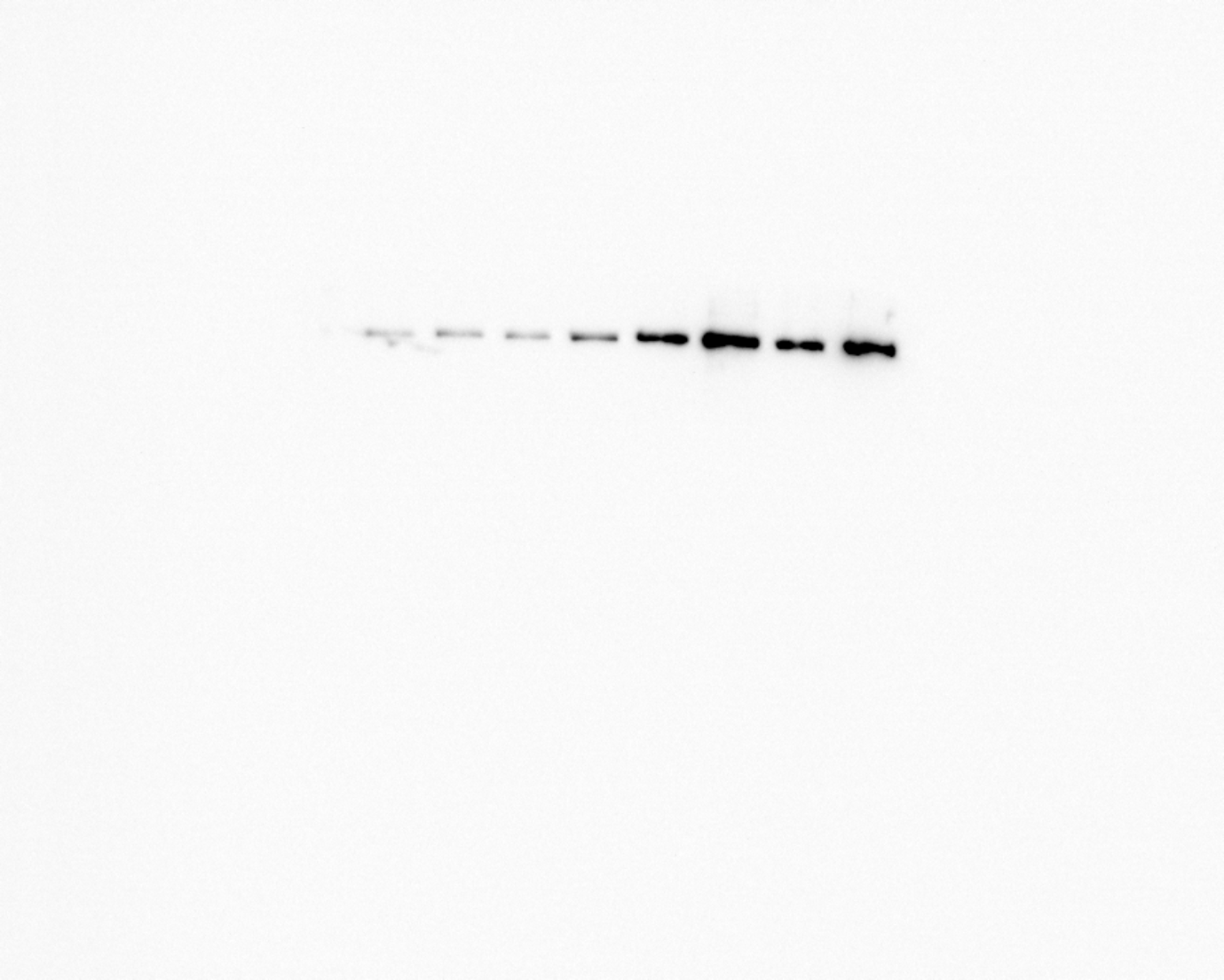

Supplement: Figure 1—source data 1. — Including uncropped Western blot images and raw statistics. [file elife-76436-fig1-data1.zip › Figure 1-Source Data 1/Figure 1I full raw unedited/IP-IB-GFP-Right.tif]

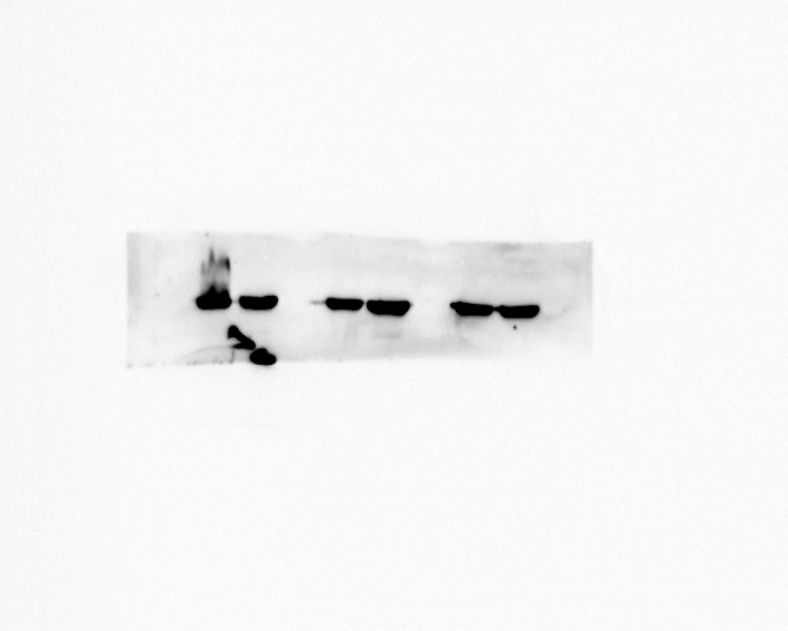

Supplement: Figure 1—source data 1. — Including uncropped Western blot images and raw statistics. [file elife-76436-fig1-data1.zip › Figure 1-Source Data 1/Figure 1J full raw unedited/IB-Actin.tif]

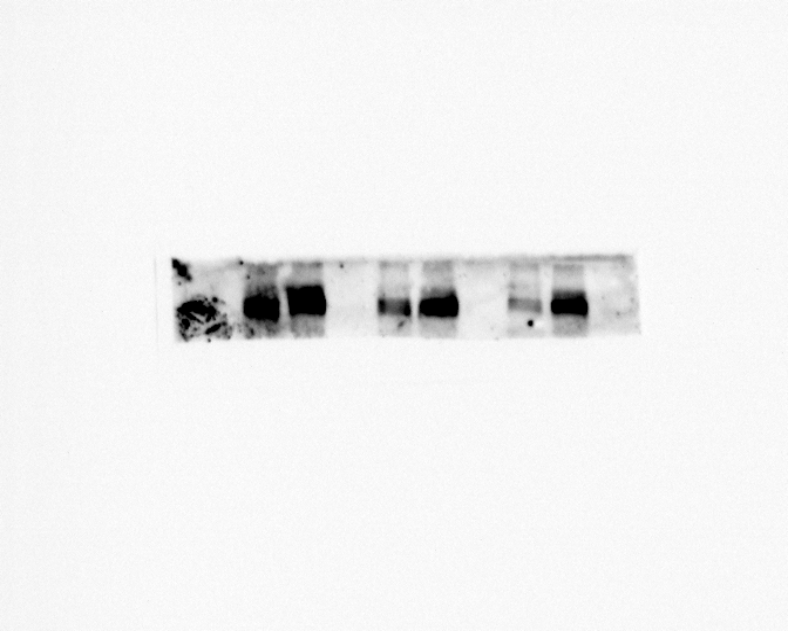

Supplement: Figure 1—source data 1. — Including uncropped Western blot images and raw statistics. [file elife-76436-fig1-data1.zip › Figure 1-Source Data 1/Figure 1J full raw unedited/IB-CED-1.tif]

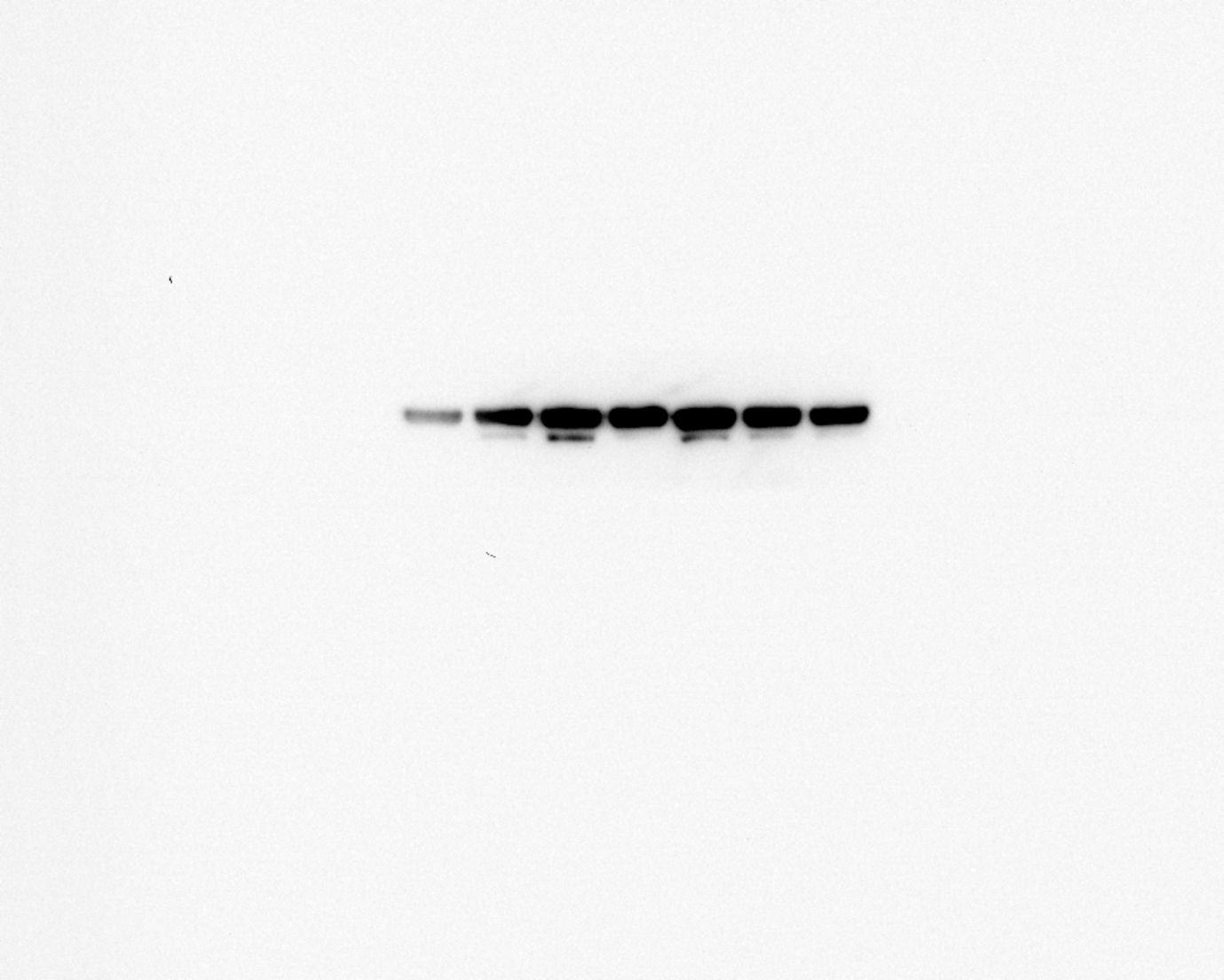

Supplement: Figure 1—source data 1. — Including uncropped Western blot images and raw statistics. [file elife-76436-fig1-data1.zip › Figure 1-Source Data 1/Figure 1L full raw unedited/IB-Actin.tif]

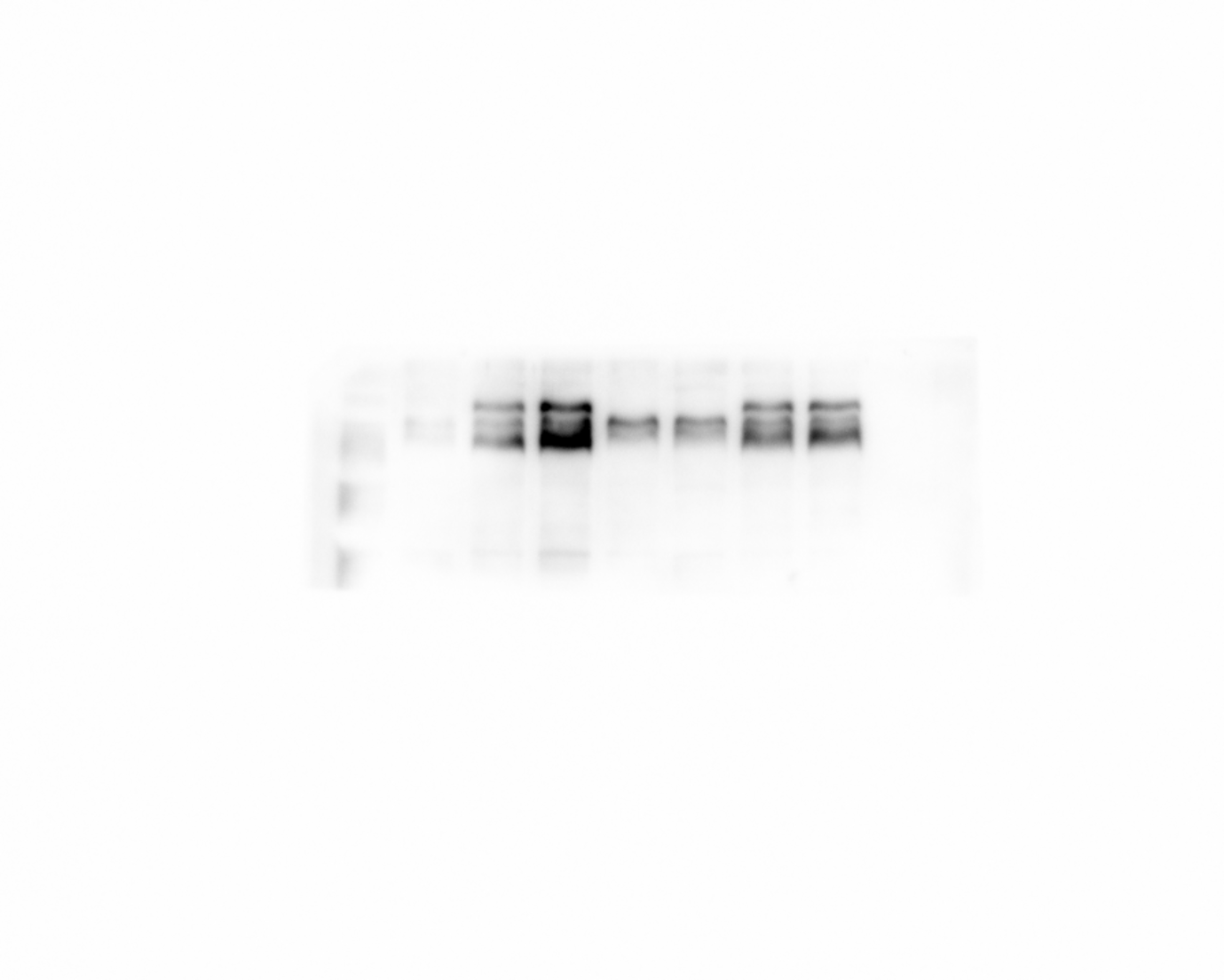

Supplement: Figure 1—source data 1. — Including uncropped Western blot images and raw statistics. [file elife-76436-fig1-data1.zip › Figure 1-Source Data 1/Figure 1L full raw unedited/IB-CED-1.tif]

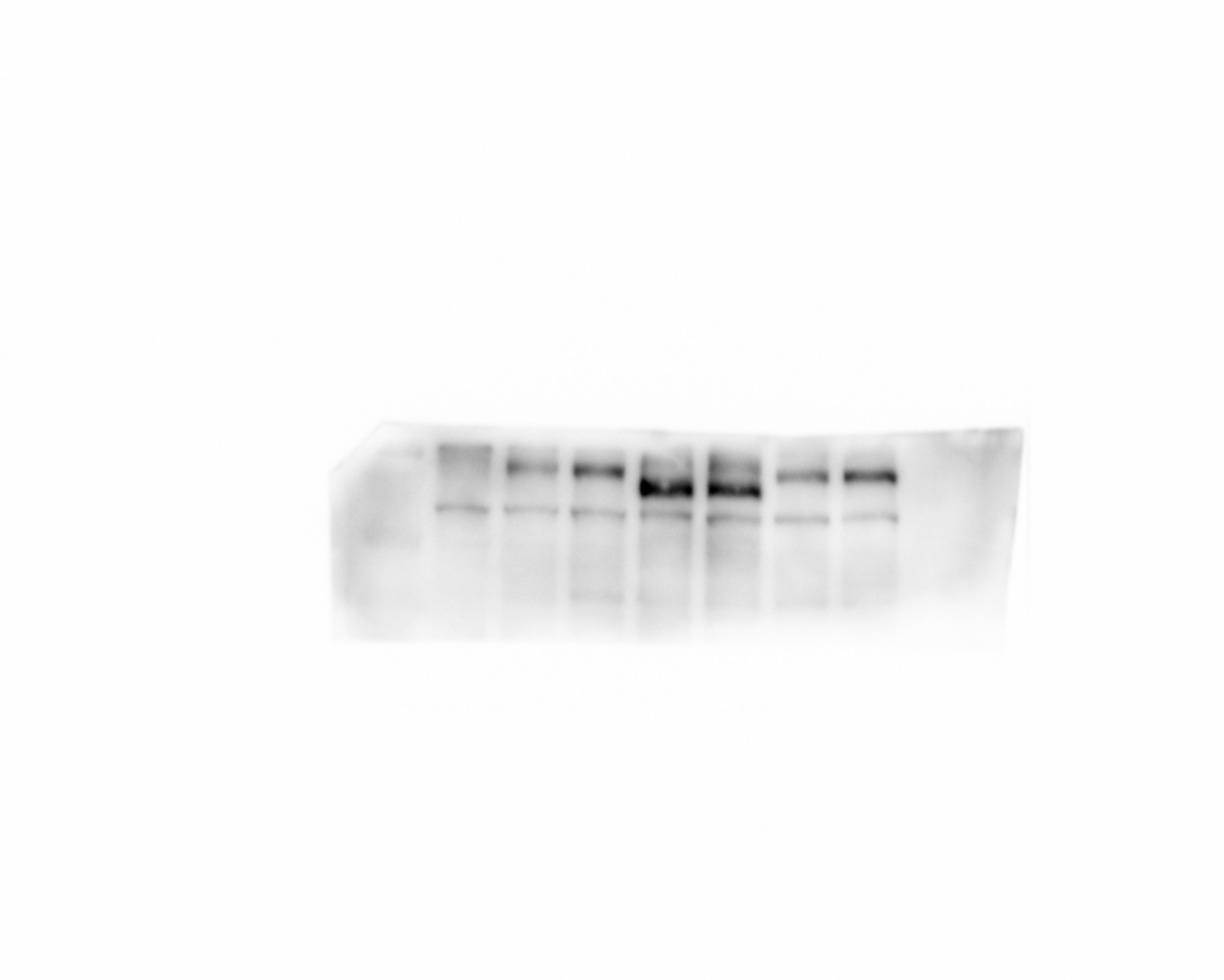

Supplement: Figure 1—source data 1. — Including uncropped Western blot images and raw statistics. [file elife-76436-fig1-data1.zip › Figure 1-Source Data 1/Figure 1L full raw unedited/IB-GFP.tif]

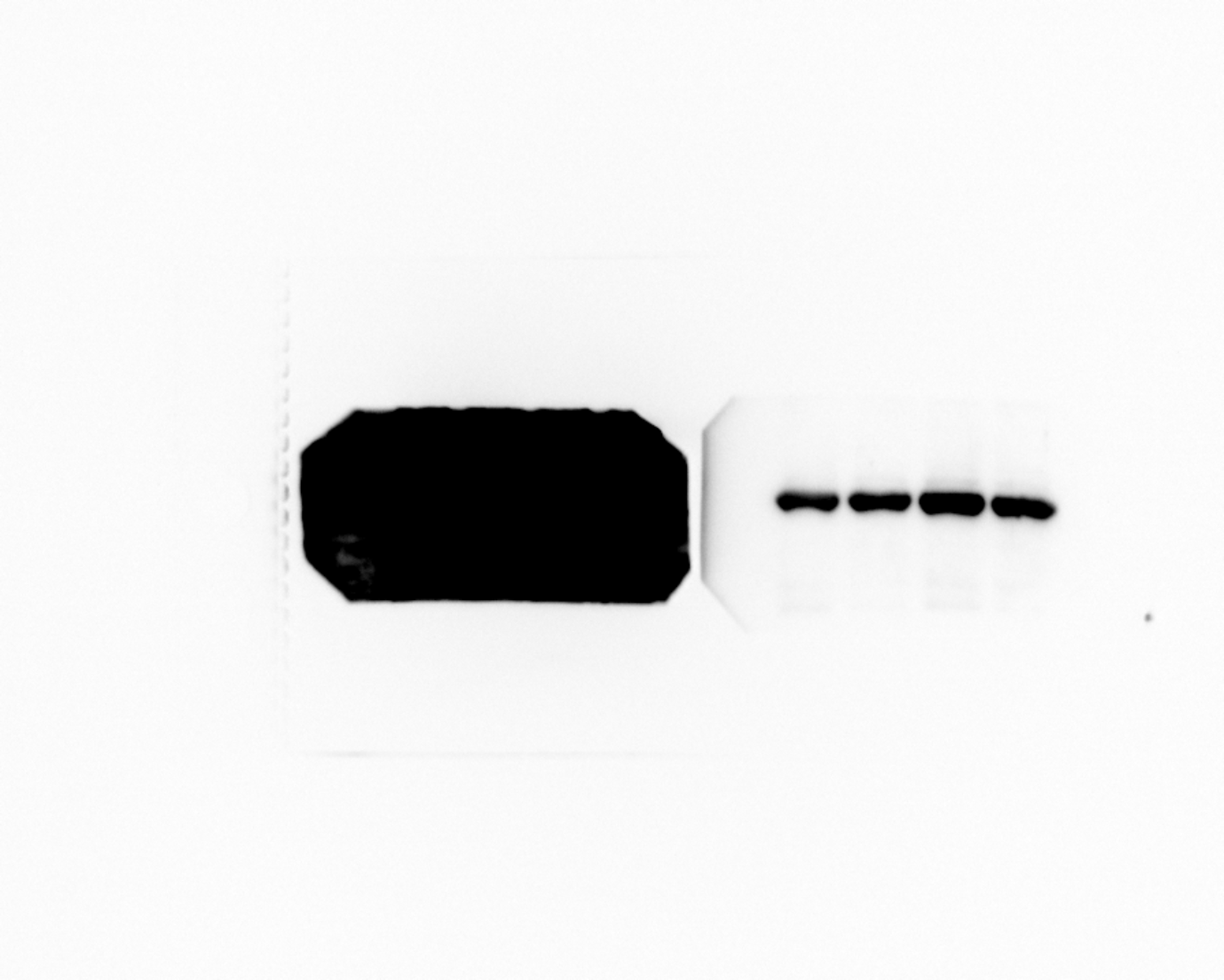

Supplement: Figure 1—source data 1. — Including uncropped Western blot images and raw statistics. [file elife-76436-fig1-data1.zip › Figure 1-Source Data 1/Figure 1M full raw unedited/N2-IB-Actin.tif]

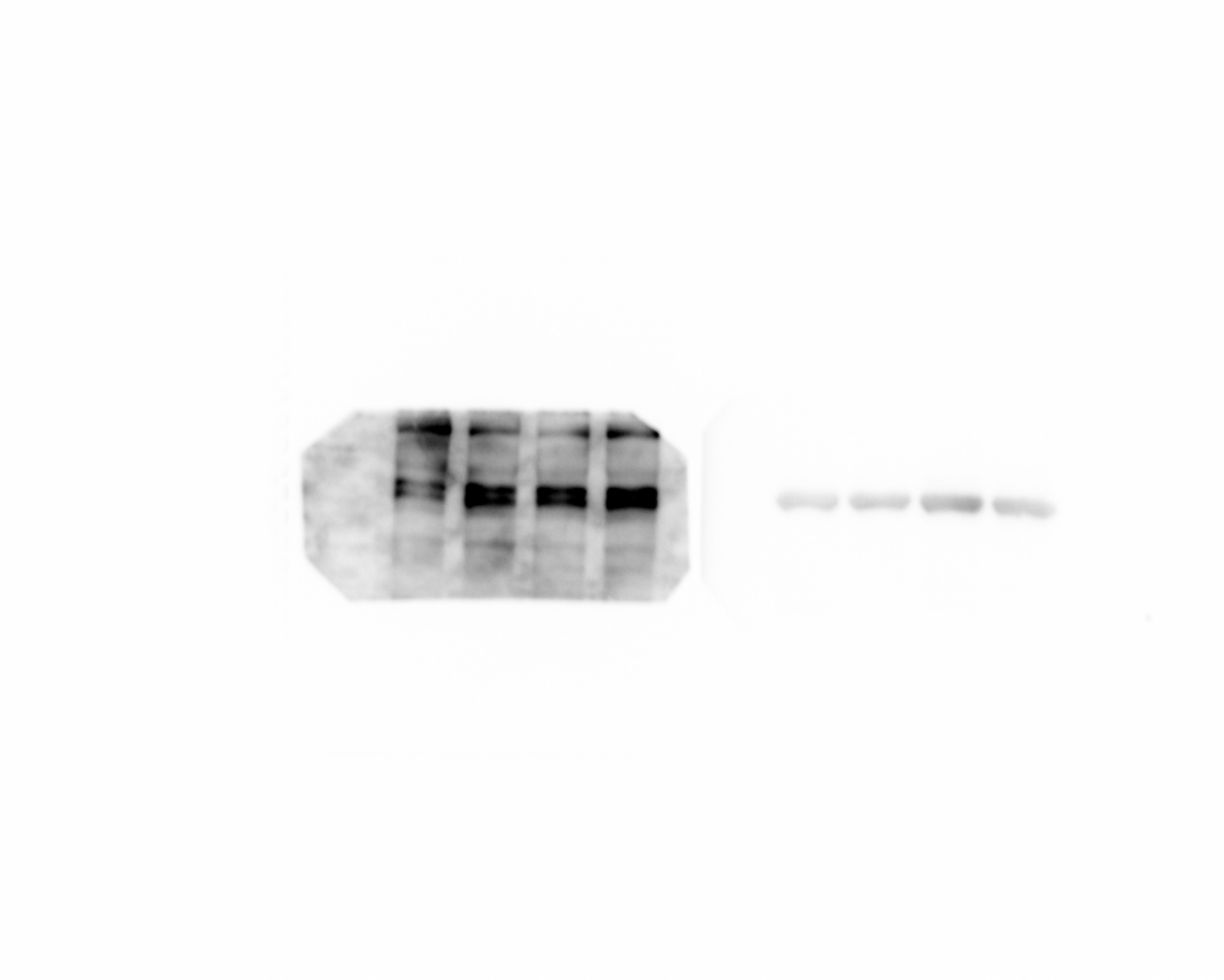

Supplement: Figure 1—source data 1. — Including uncropped Western blot images and raw statistics. [file elife-76436-fig1-data1.zip › Figure 1-Source Data 1/Figure 1M full raw unedited/N2-IB-CED-1.tif]

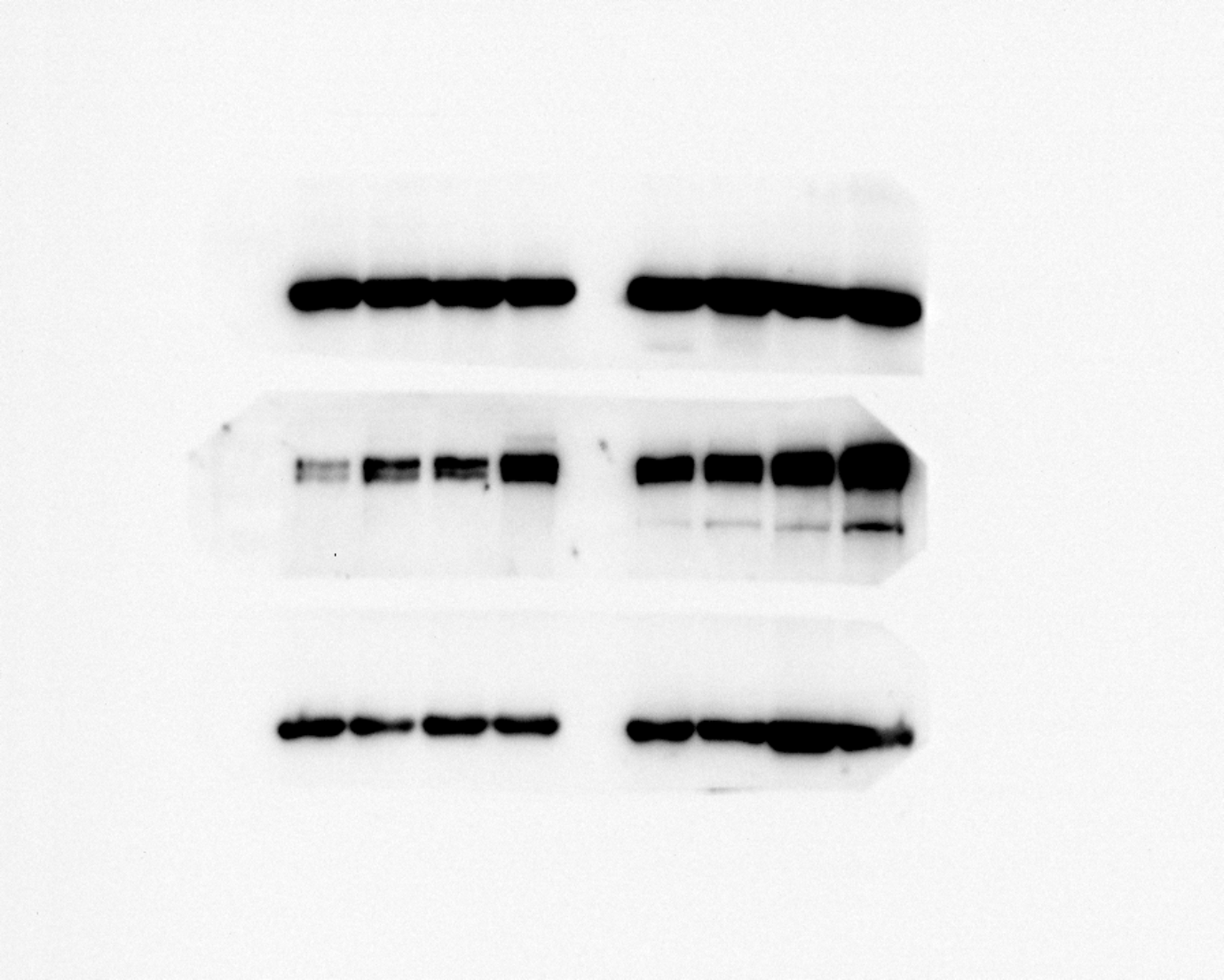

Supplement: Figure 1—source data 1. — Including uncropped Western blot images and raw statistics. [file elife-76436-fig1-data1.zip › Figure 1-Source Data 1/Figure 1M full raw unedited/trim-21-IB-Actin.tif]

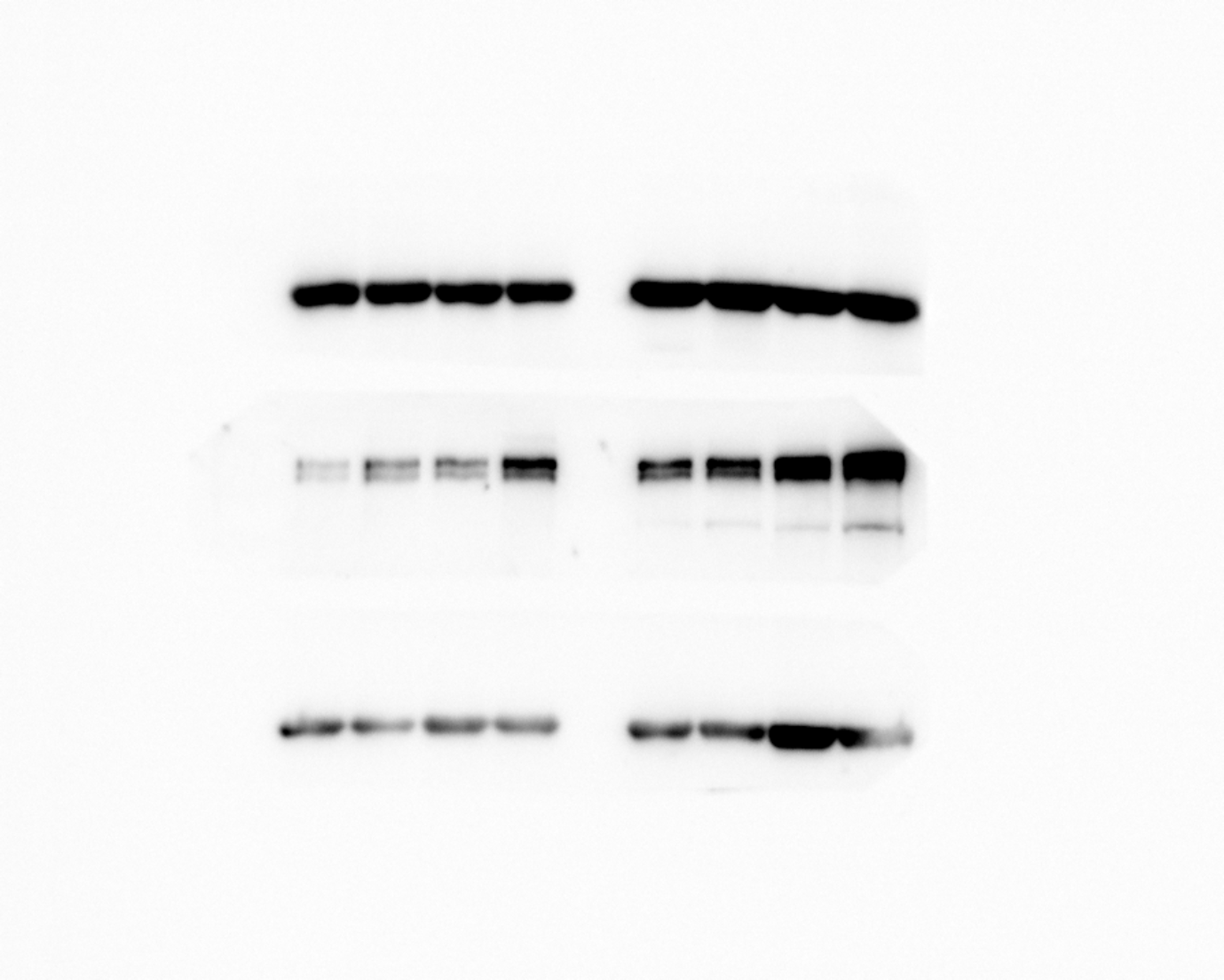

Supplement: Figure 1—source data 1. — Including uncropped Western blot images and raw statistics. [file elife-76436-fig1-data1.zip › Figure 1-Source Data 1/Figure 1M full raw unedited/trim-21-IB-CED-1.tif]

Figure 1-figure supplement 1A


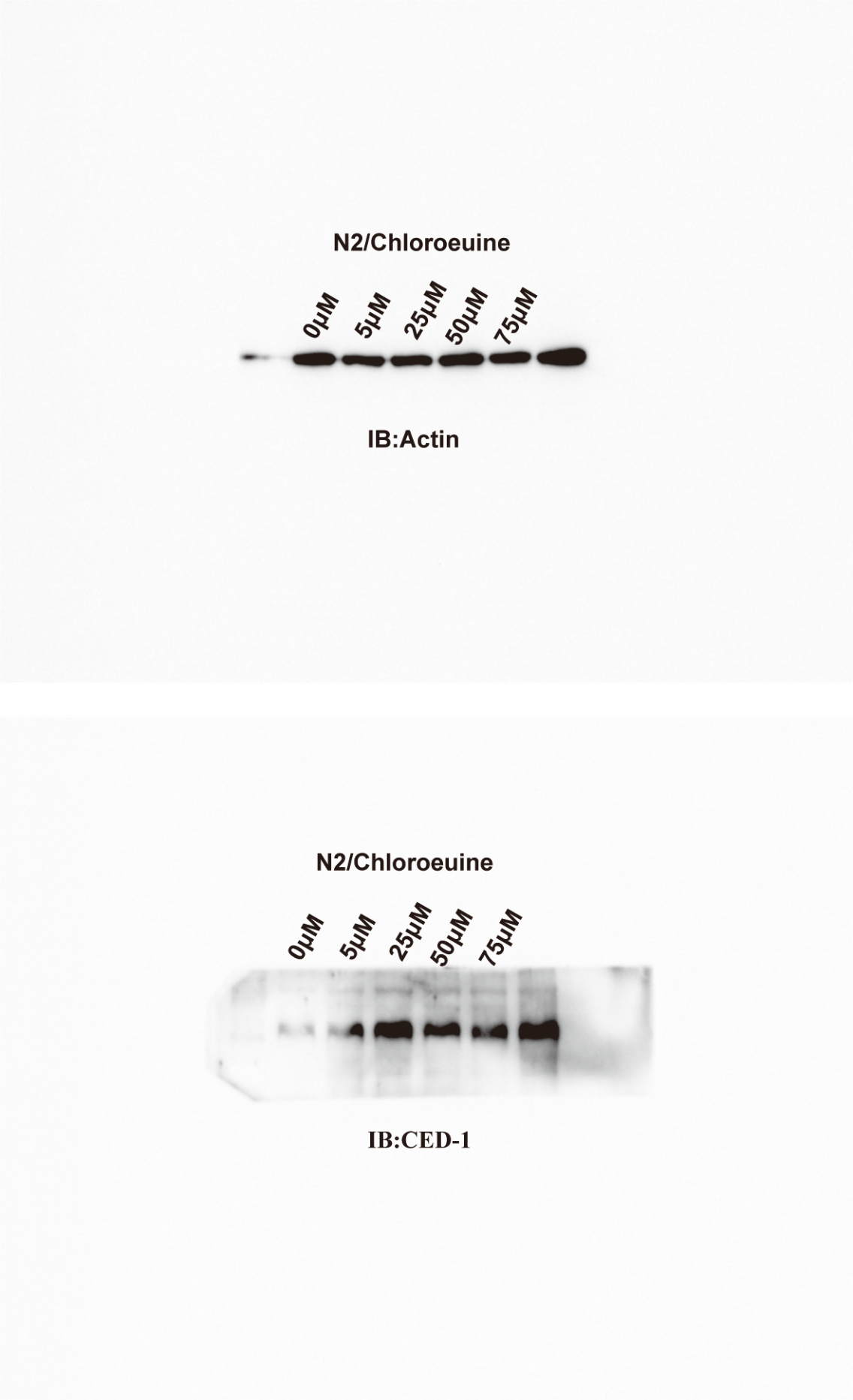


Figure 1-figure supplement 1B


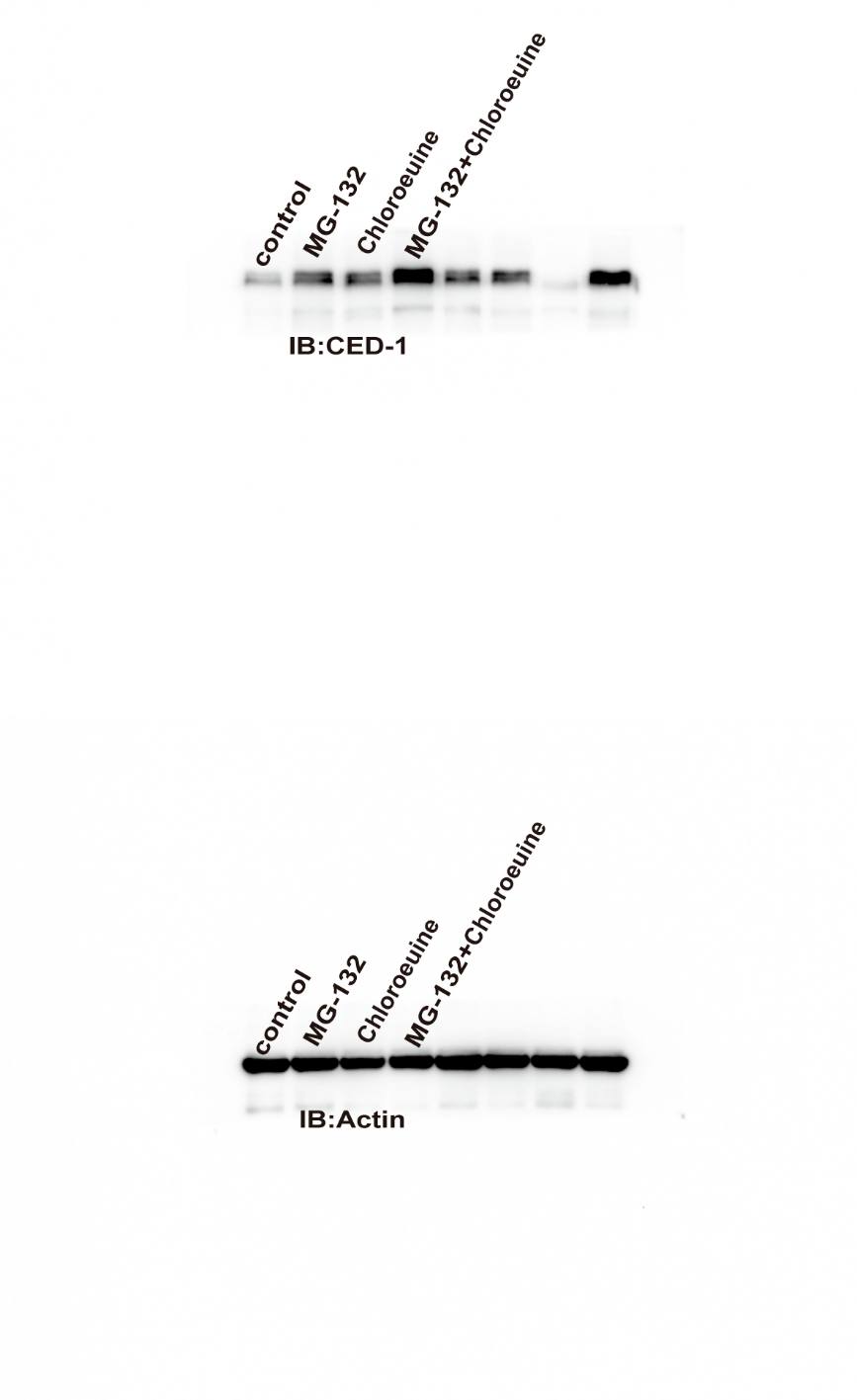


Figure 1-figure supplement 1I


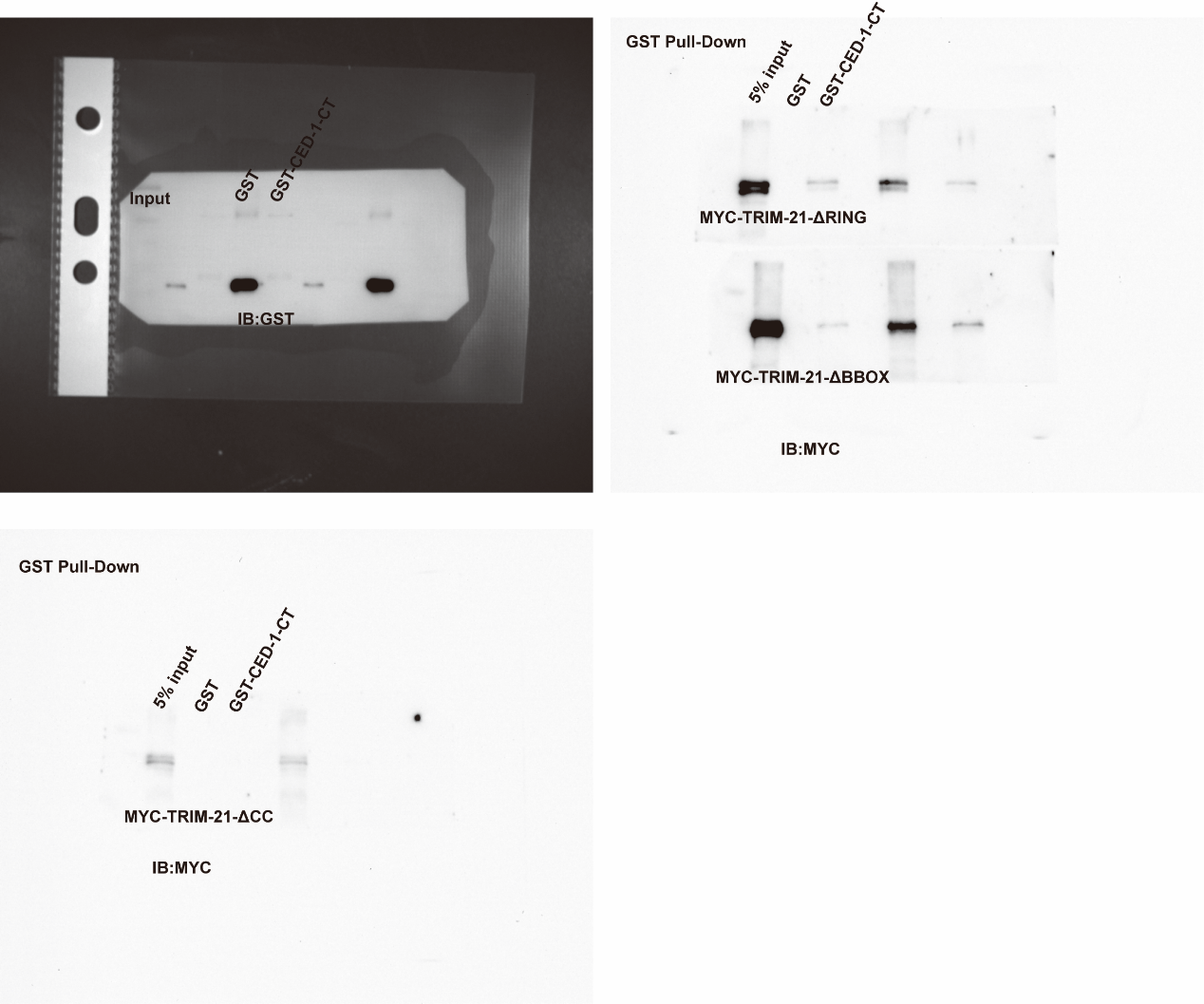

Supplement: Figure 1—figure supplement 1—source data 1. — Including uncropped Western blot images and raw statistics. [file elife-76436-fig1-figsupp1-data1.zip › Figure 1-figure supplement 1-Source Data 1/Figure 1-figure supplement 1 uncroppped blot with relevant bands.docx]

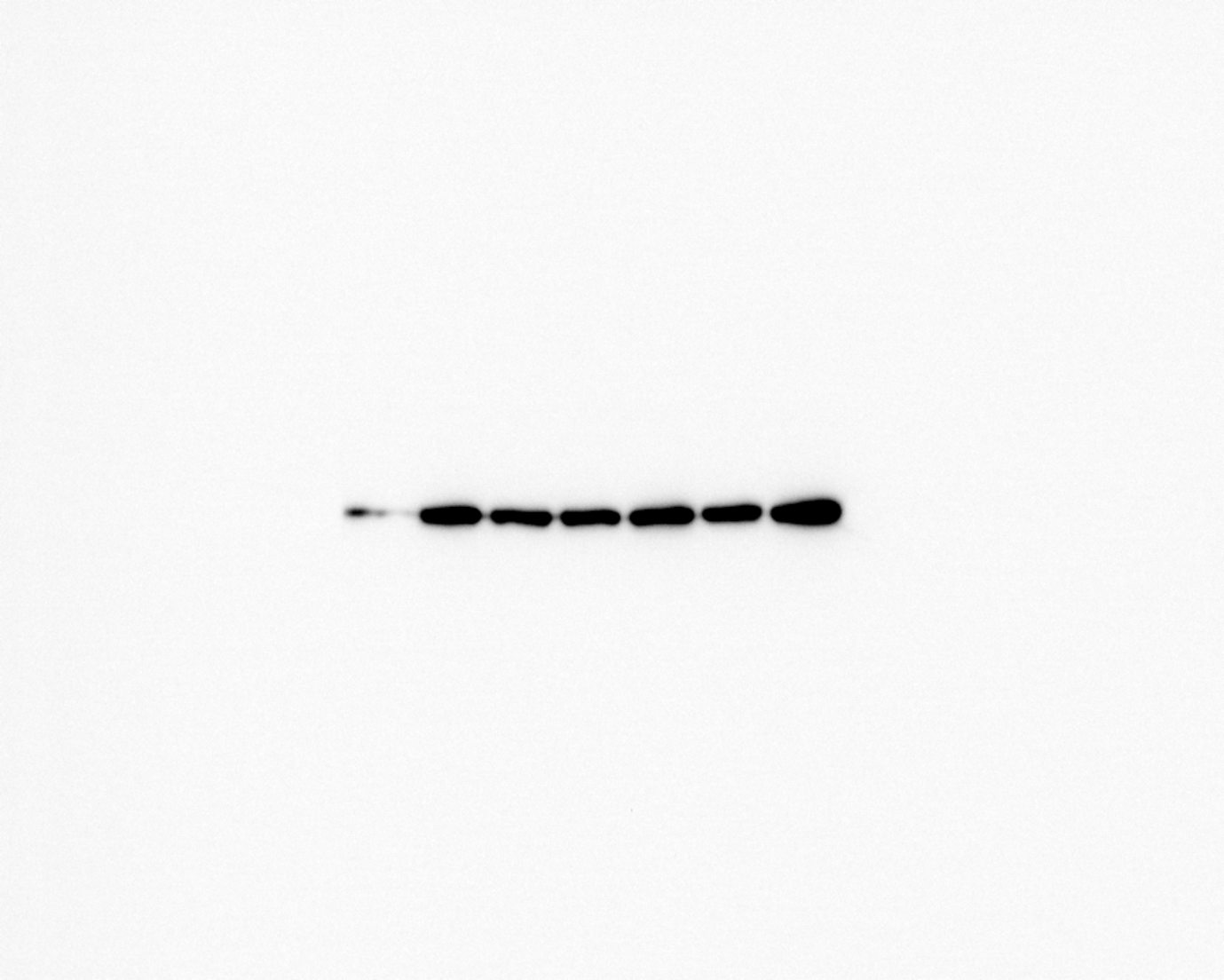

Supplement: Figure 1—figure supplement 1—source data 1. — Including uncropped Western blot images and raw statistics. [file elife-76436-fig1-figsupp1-data1.zip › Figure 1-figure supplement 1-Source Data 1/Figure 1-figure supplement 1A full raw unedited/IB-Actin.tif]

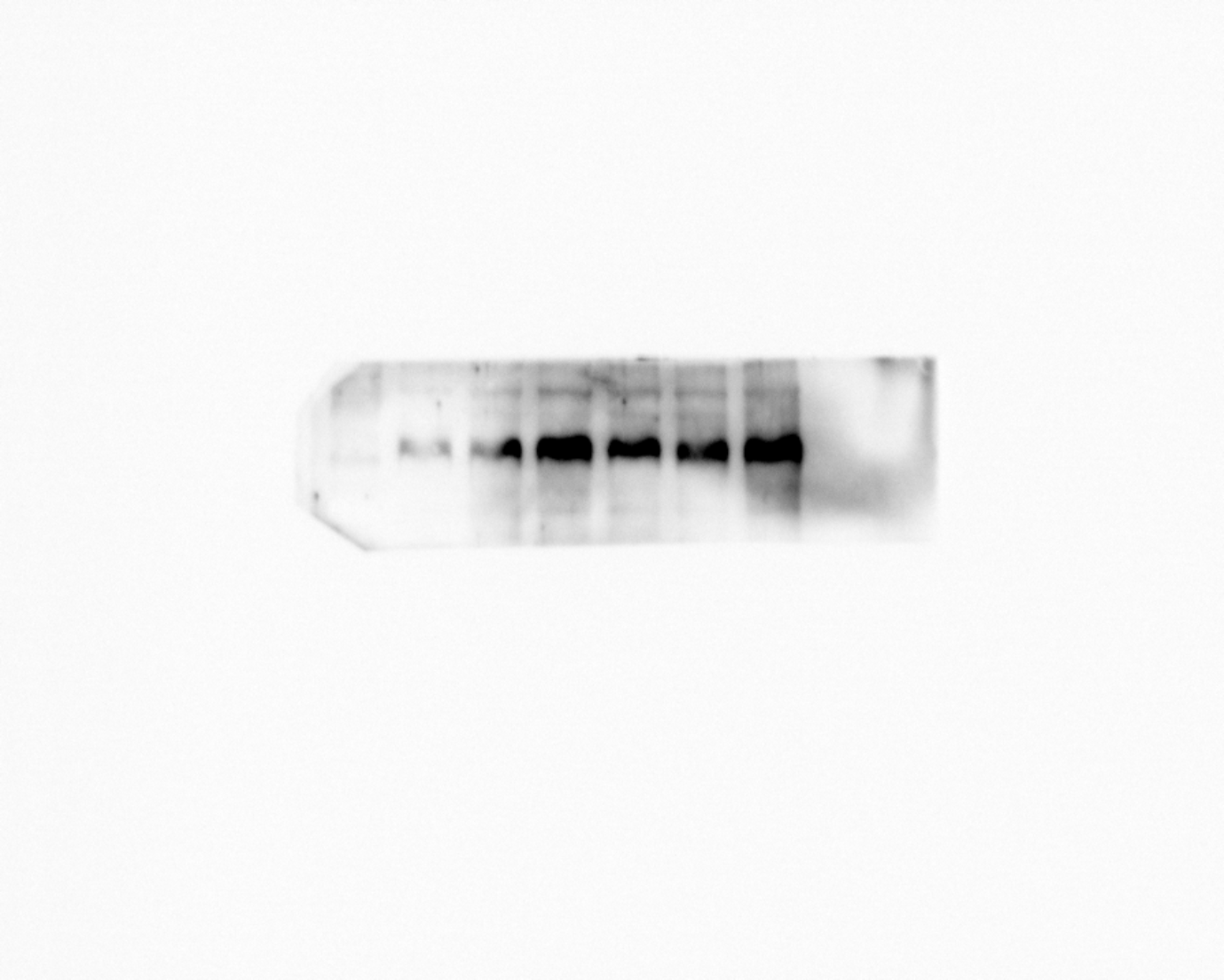

Supplement: Figure 1—figure supplement 1—source data 1. — Including uncropped Western blot images and raw statistics. [file elife-76436-fig1-figsupp1-data1.zip › Figure 1-figure supplement 1-Source Data 1/Figure 1-figure supplement 1A full raw unedited/IB-CED-1.tif]

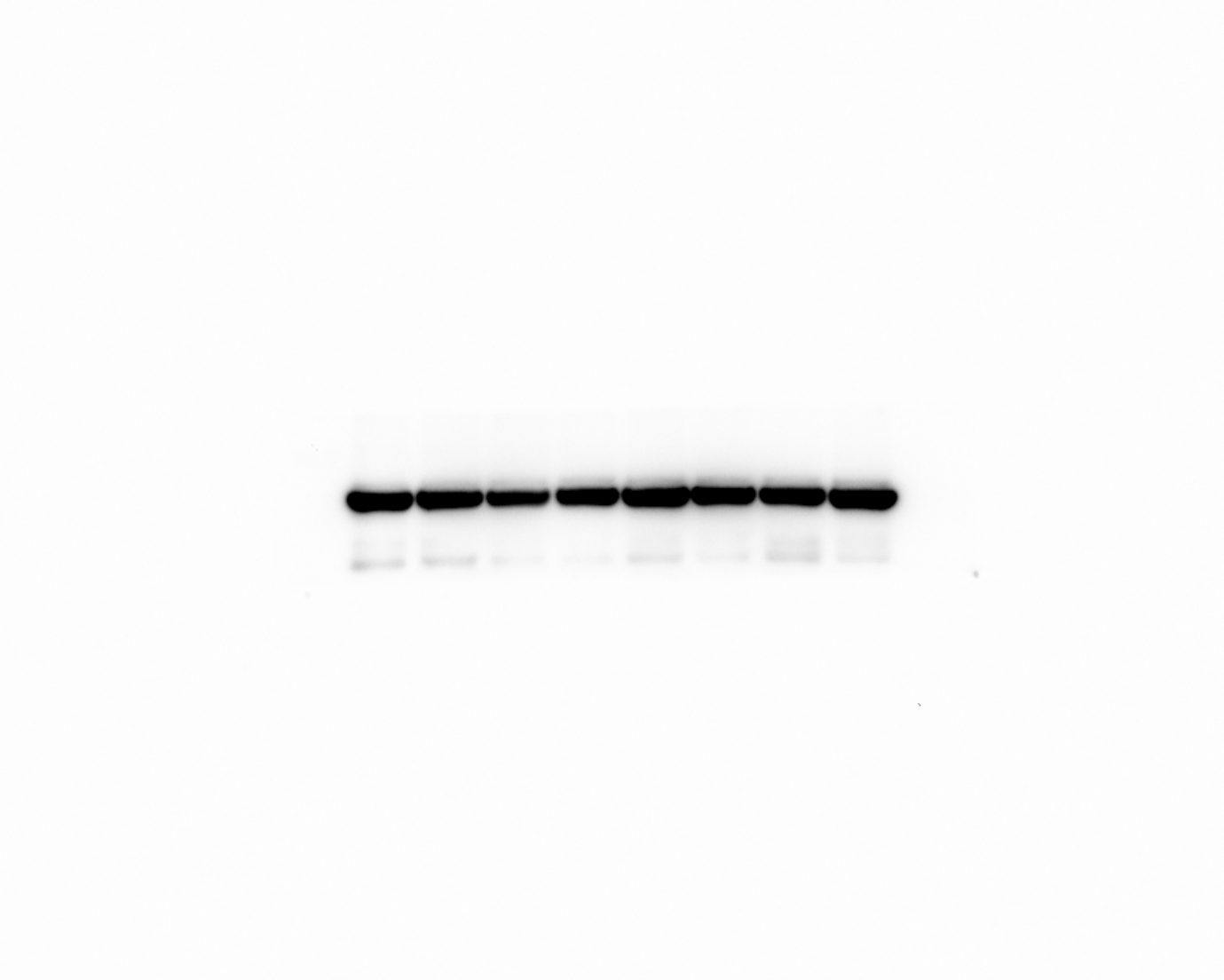

Supplement: Figure 1—figure supplement 1—source data 1. — Including uncropped Western blot images and raw statistics. [file elife-76436-fig1-figsupp1-data1.zip › Figure 1-figure supplement 1-Source Data 1/Figure 1-figure supplement 1B full raw unedited/IB-Actin.tif]

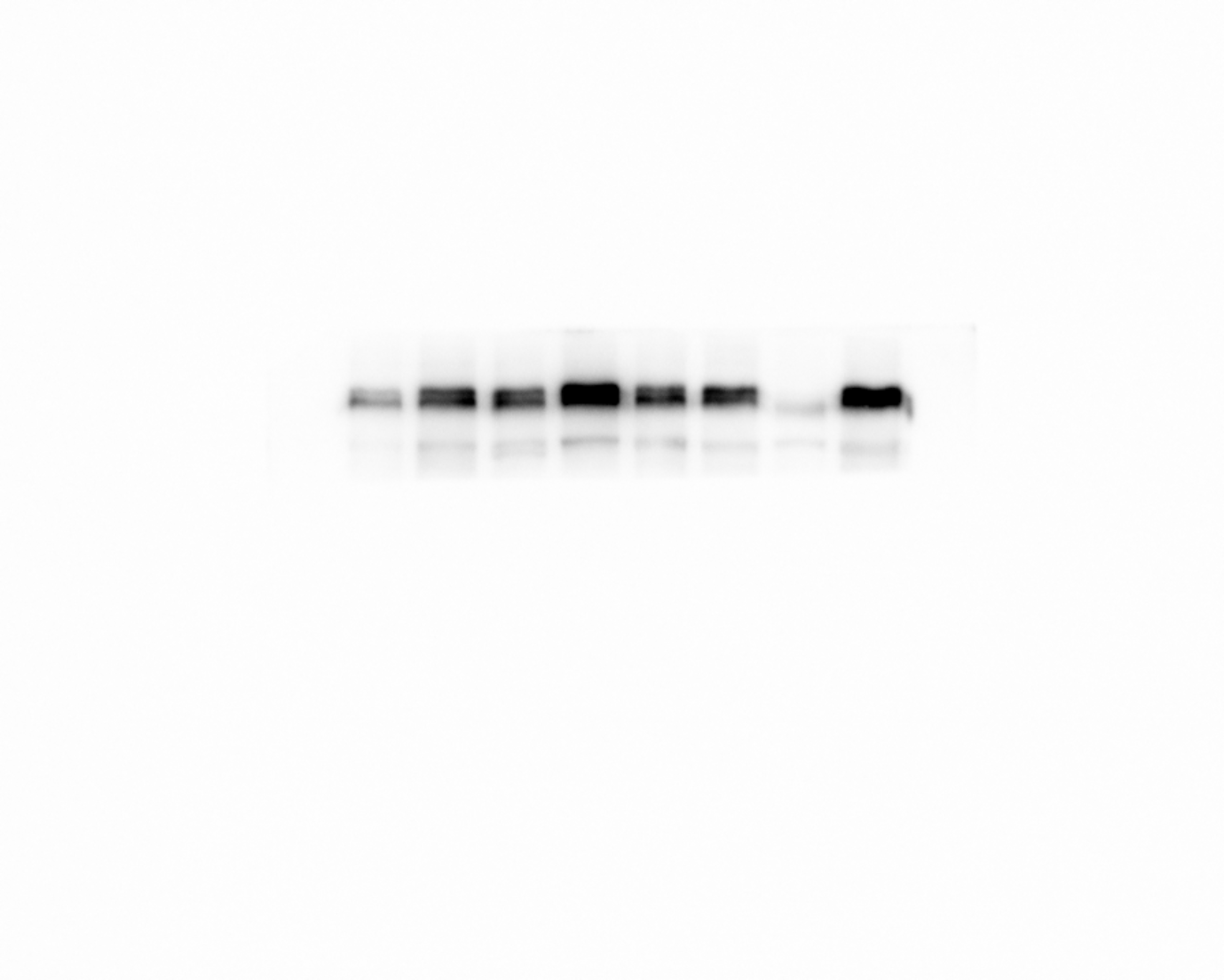

Supplement: Figure 1—figure supplement 1—source data 1. — Including uncropped Western blot images and raw statistics. [file elife-76436-fig1-figsupp1-data1.zip › Figure 1-figure supplement 1-Source Data 1/Figure 1-figure supplement 1B full raw unedited/IB-CED-1.tif]

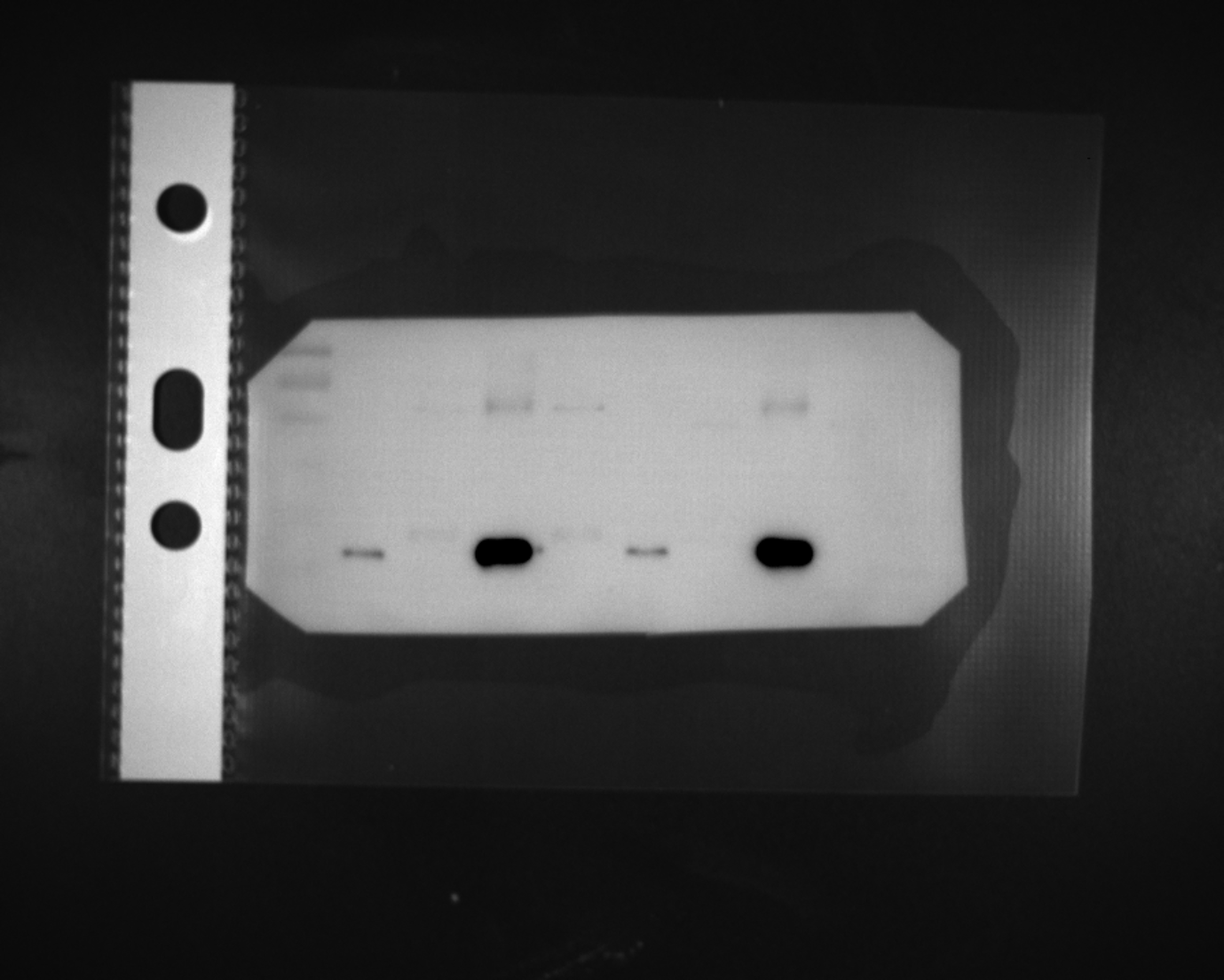

Supplement: Figure 1—figure supplement 1—source data 1. — Including uncropped Western blot images and raw statistics. [file elife-76436-fig1-figsupp1-data1.zip › Figure 1-figure supplement 1-Source Data 1/Figure 1-figure supplement 1I full raw unedited/IB-GST.tif]

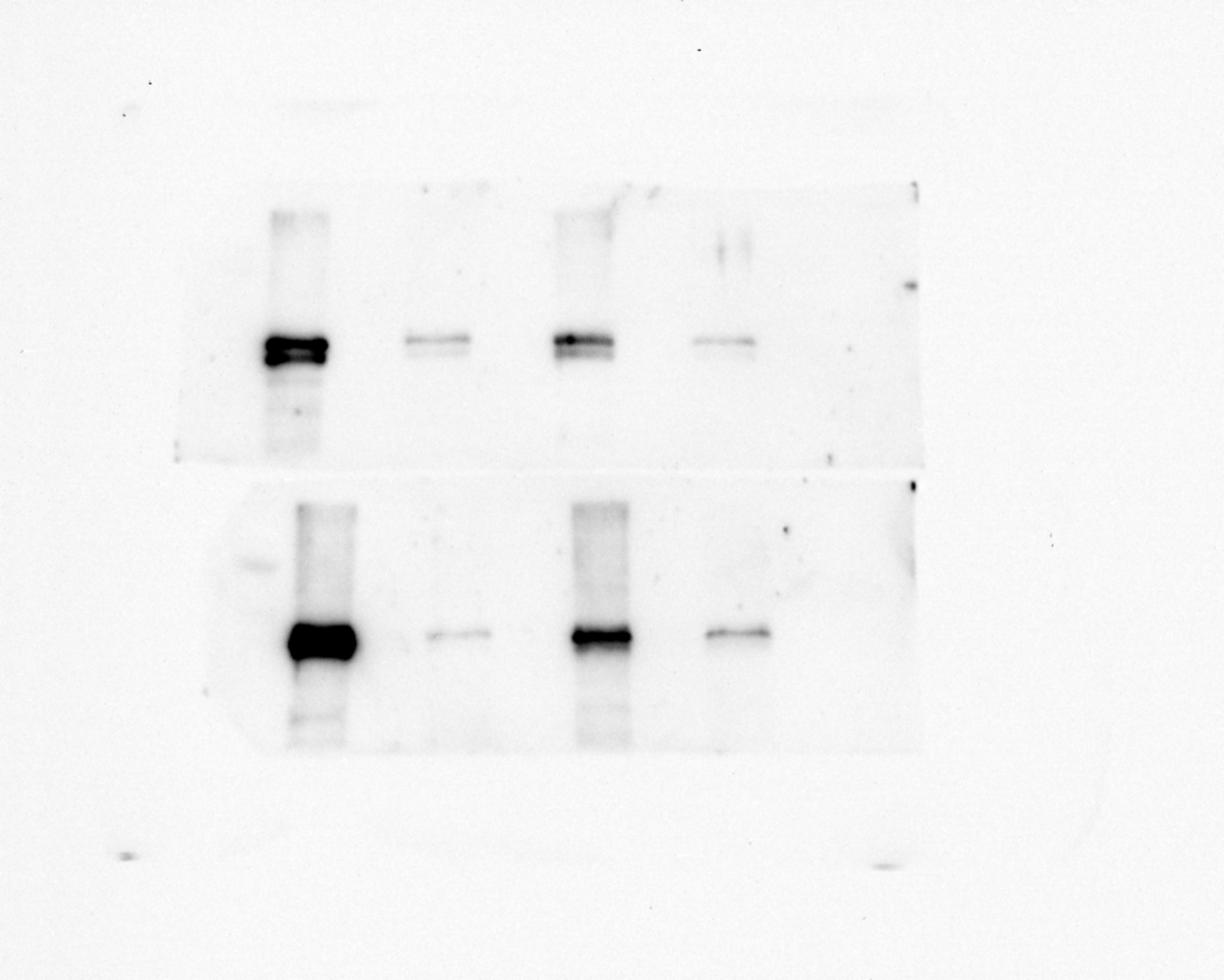

Supplement: Figure 1—figure supplement 1—source data 1. — Including uncropped Western blot images and raw statistics. [file elife-76436-fig1-figsupp1-data1.zip › Figure 1-figure supplement 1-Source Data 1/Figure 1-figure supplement 1I full raw unedited/IB-MYC-TRIM-21-ΔBBOX.tif]

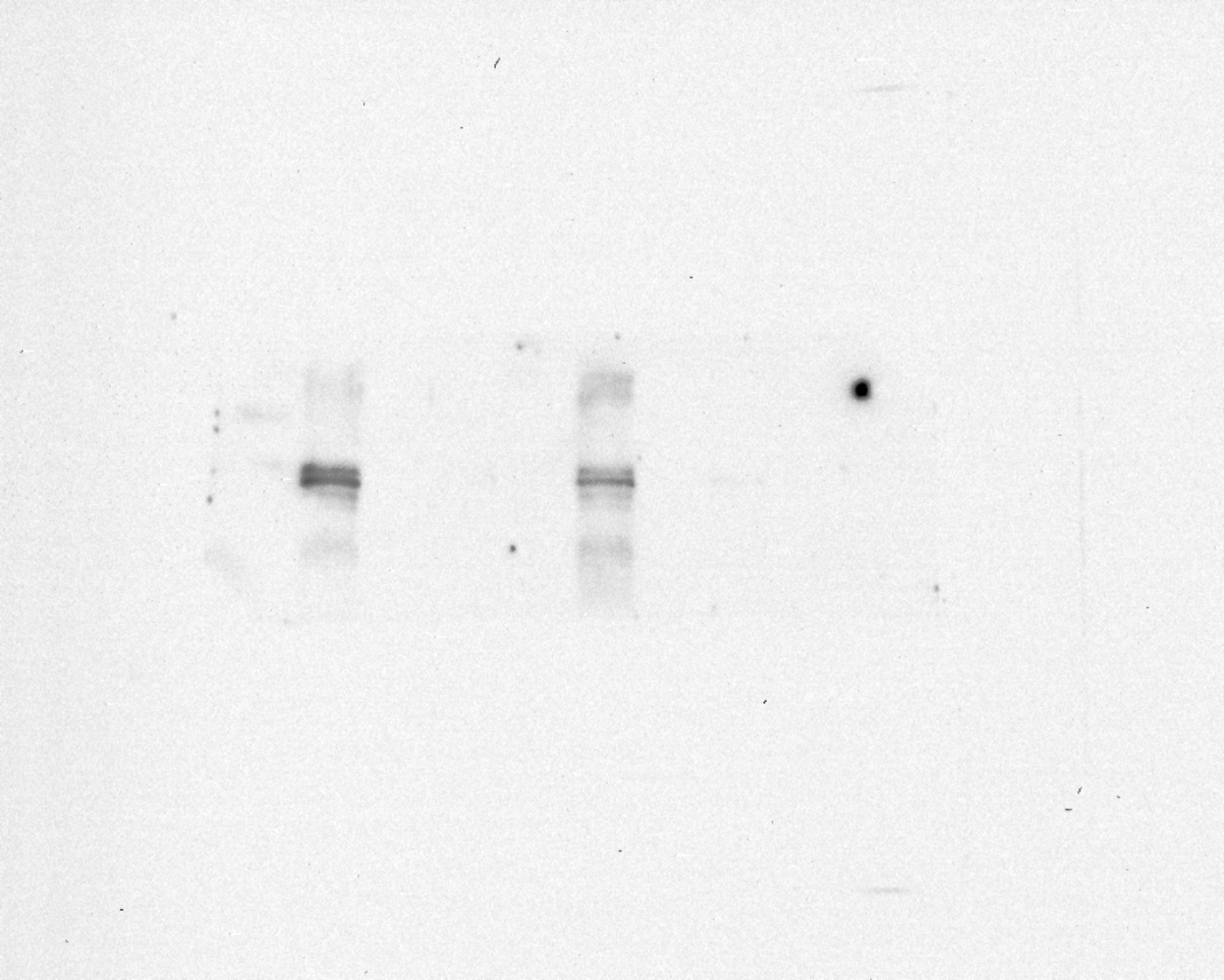

Supplement: Figure 1—figure supplement 1—source data 1. — Including uncropped Western blot images and raw statistics. [file elife-76436-fig1-figsupp1-data1.zip › Figure 1-figure supplement 1-Source Data 1/Figure 1-figure supplement 1I full raw unedited/IB-MYC-TRIM-21-ΔCC.tif]

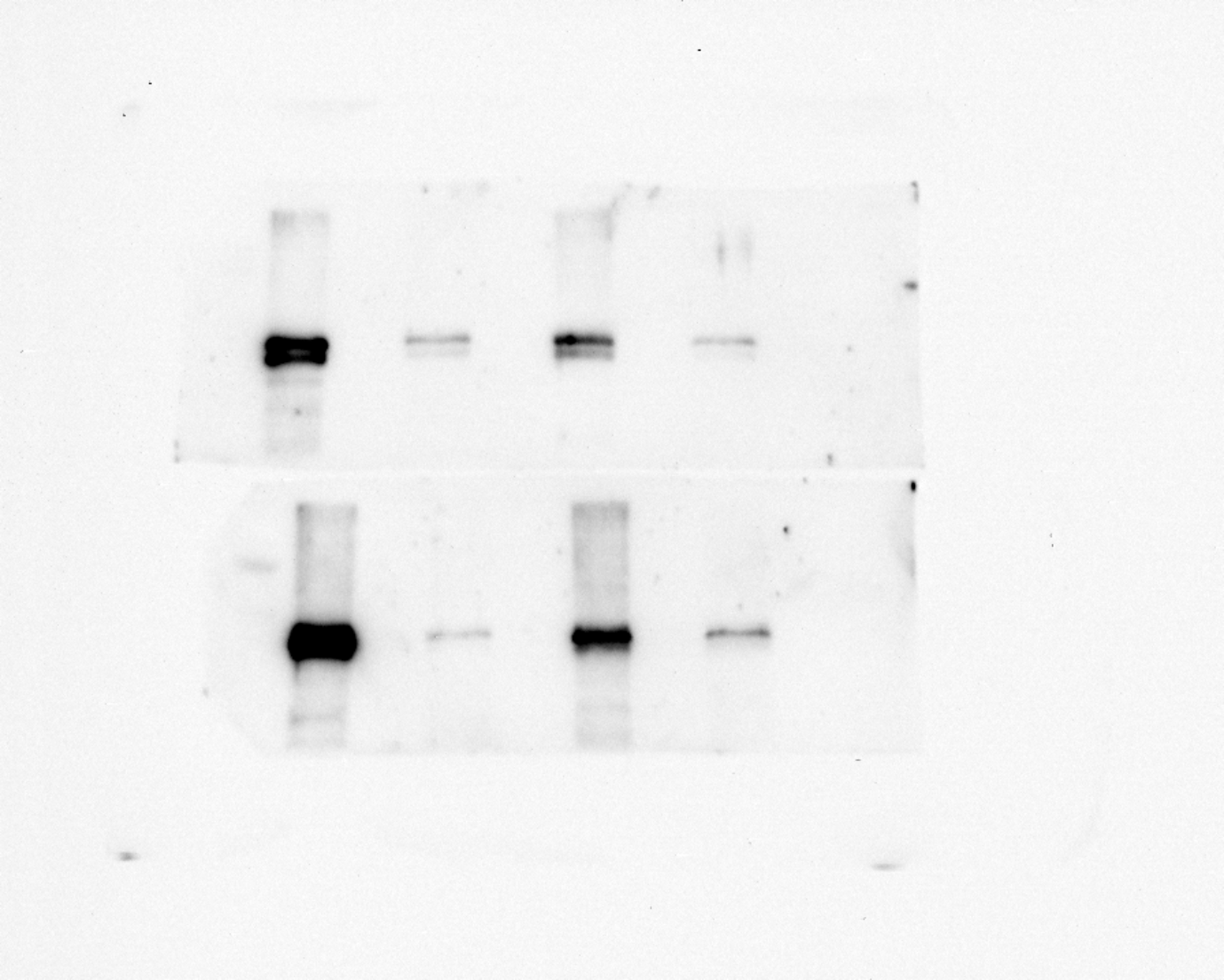

Supplement: Figure 1—figure supplement 1—source data 1. — Including uncropped Western blot images and raw statistics. [file elife-76436-fig1-figsupp1-data1.zip › Figure 1-figure supplement 1-Source Data 1/Figure 1-figure supplement 1I full raw unedited/IB-MYC-TRIM-21-ΔRING.tif]

Figure 1-figure supplement 2D


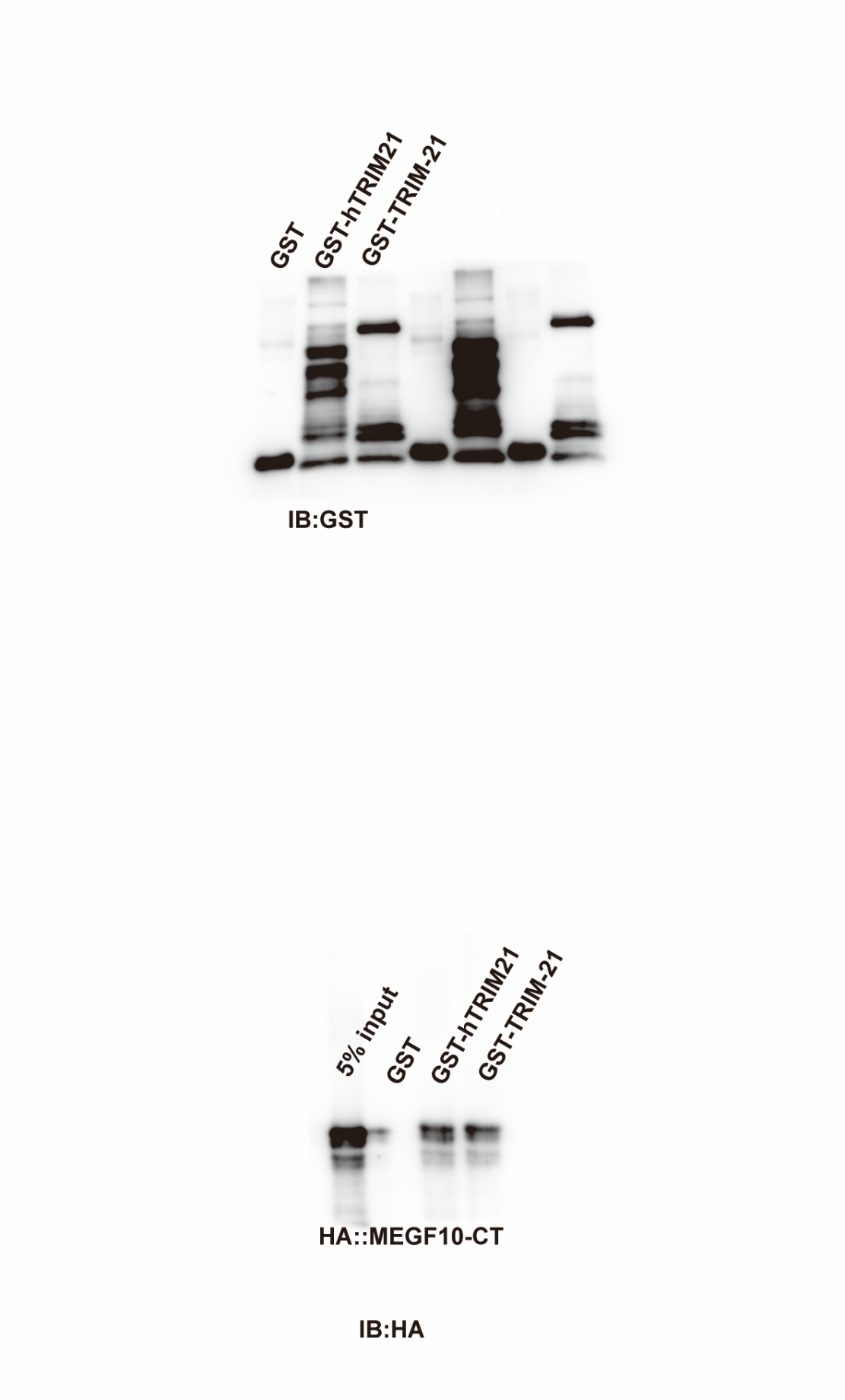


Figure 1-figure supplement 2E


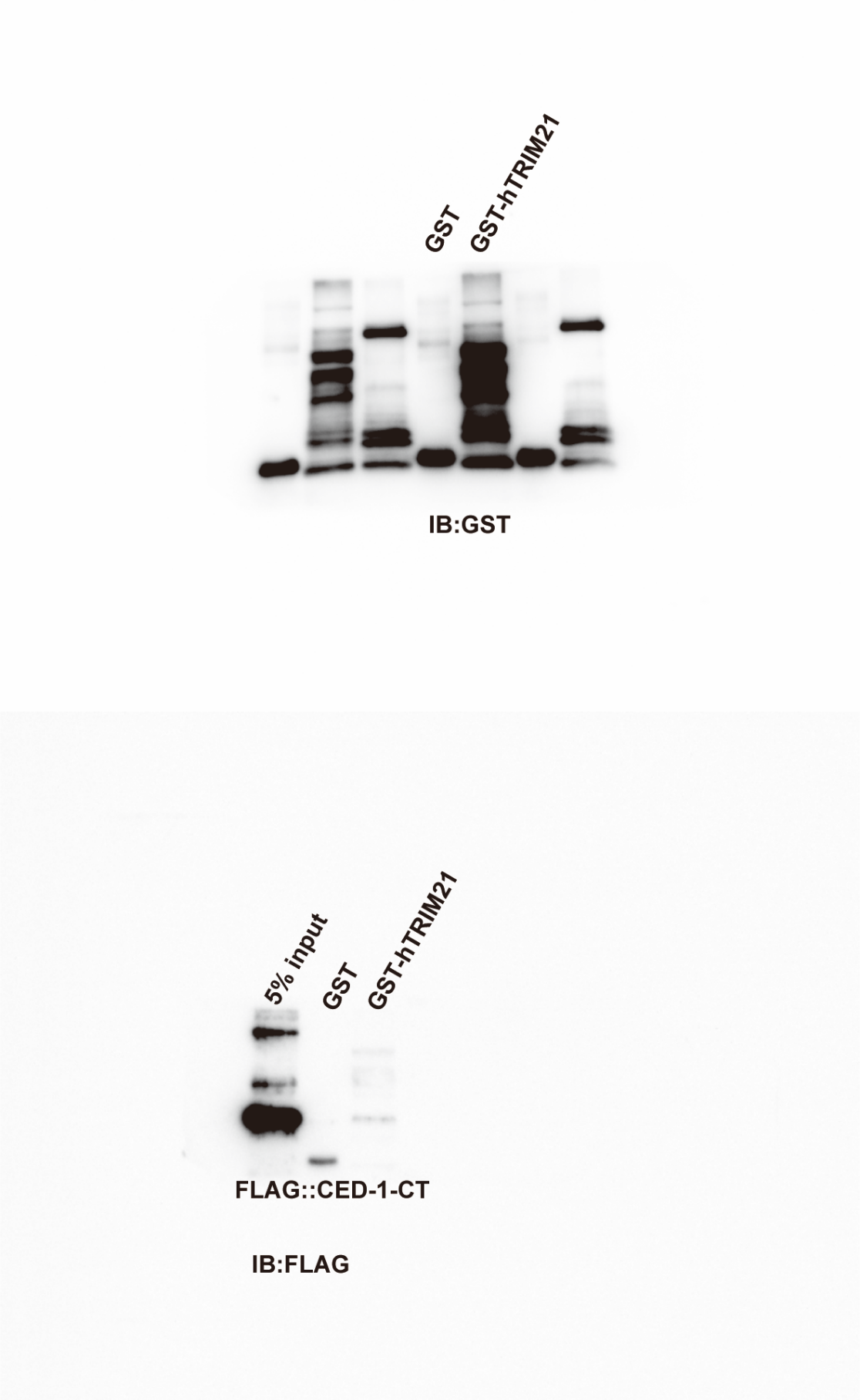

Supplement: Figure 1—figure supplement 2—source data 1. — Including uncropped Western blot images. [file elife-76436-fig1-figsupp2-data1.zip › Figure 1-figure supplement 2-Source Data 1/Figure 1-figure supplement 2 uncroppped blot with relevant bands.docx]

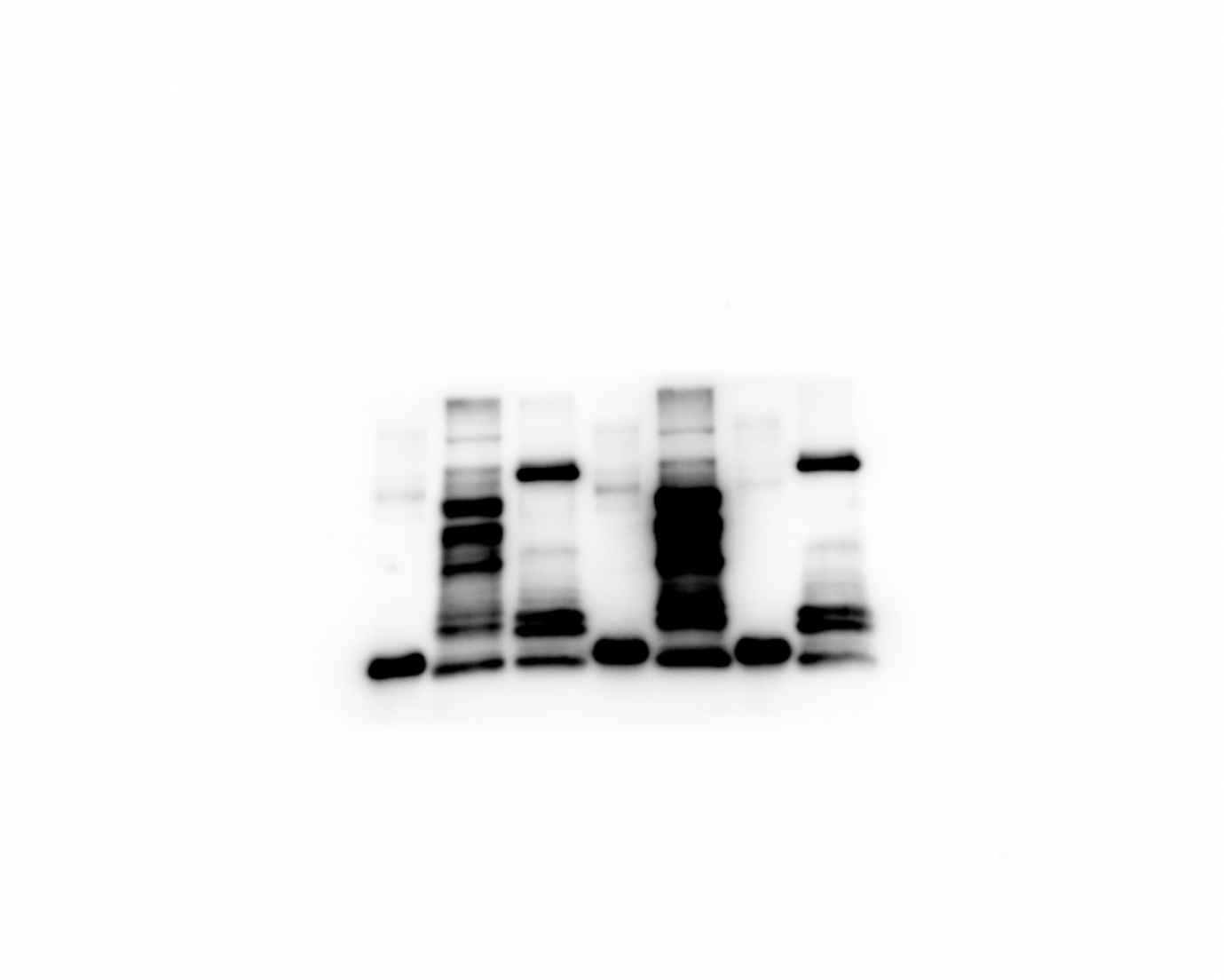

Supplement: Figure 1—figure supplement 2—source data 1. — Including uncropped Western blot images. [file elife-76436-fig1-figsupp2-data1.zip › Figure 1-figure supplement 2-Source Data 1/Figure 1-figure supplement 2D full raw unedited/IB-GST.tif]

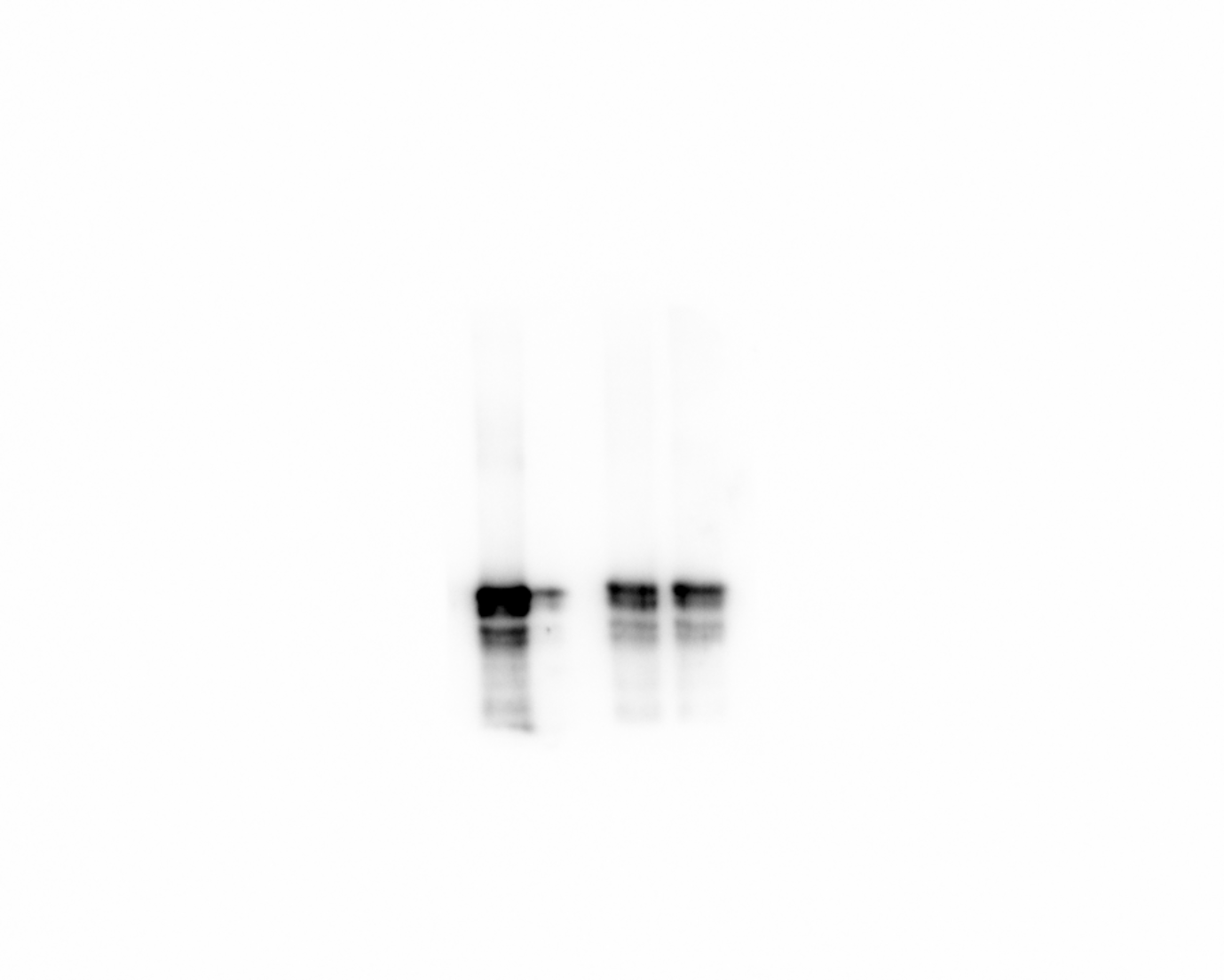

Supplement: Figure 1—figure supplement 2—source data 1. — Including uncropped Western blot images. [file elife-76436-fig1-figsupp2-data1.zip › Figure 1-figure supplement 2-Source Data 1/Figure 1-figure supplement 2D full raw unedited/IB-HA.tif]

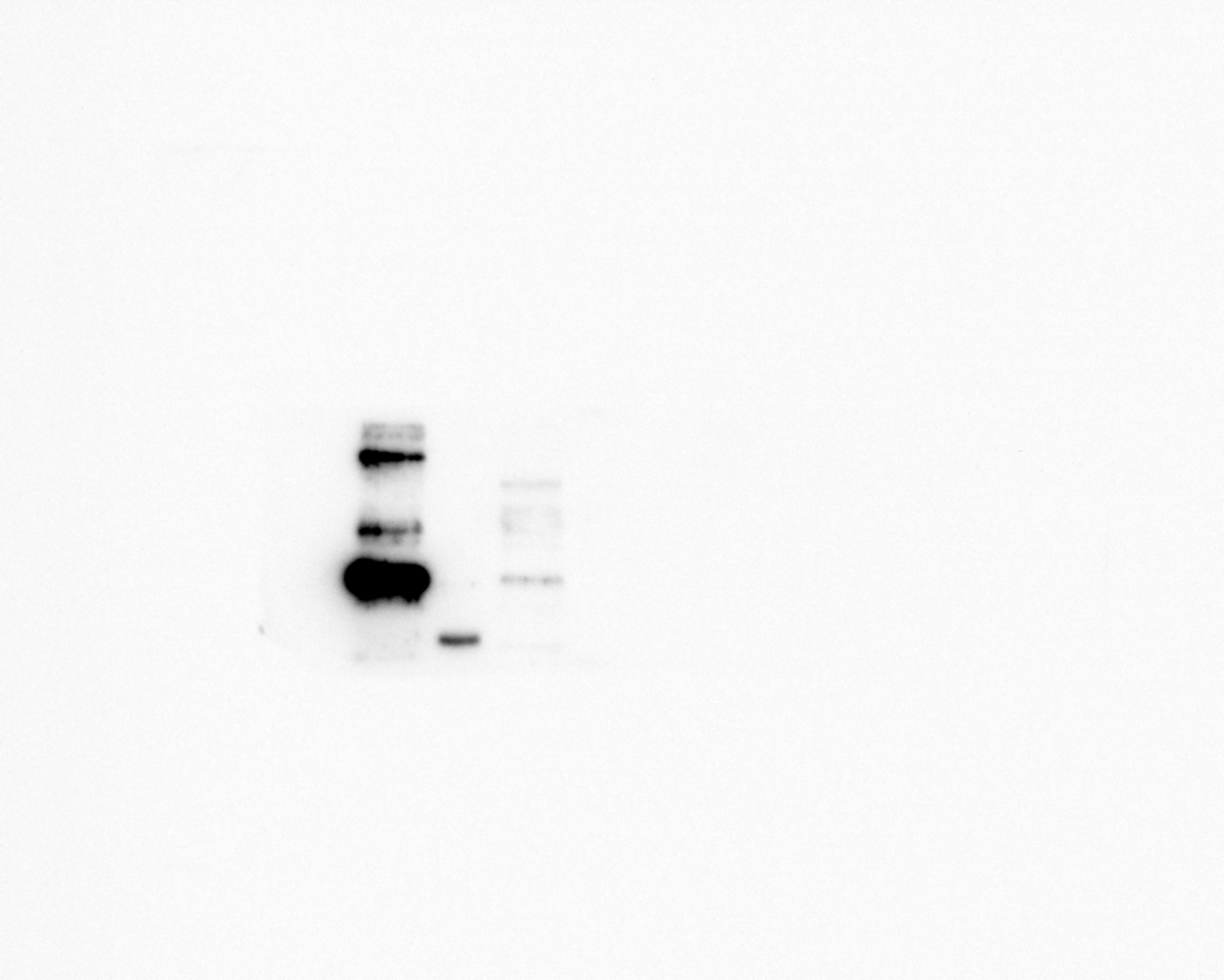

Supplement: Figure 1—figure supplement 2—source data 1. — Including uncropped Western blot images. [file elife-76436-fig1-figsupp2-data1.zip › Figure 1-figure supplement 2-Source Data 1/Figure 1-figure supplement 2E full raw unedited/IB-FLAG.tif]

Figure 2B


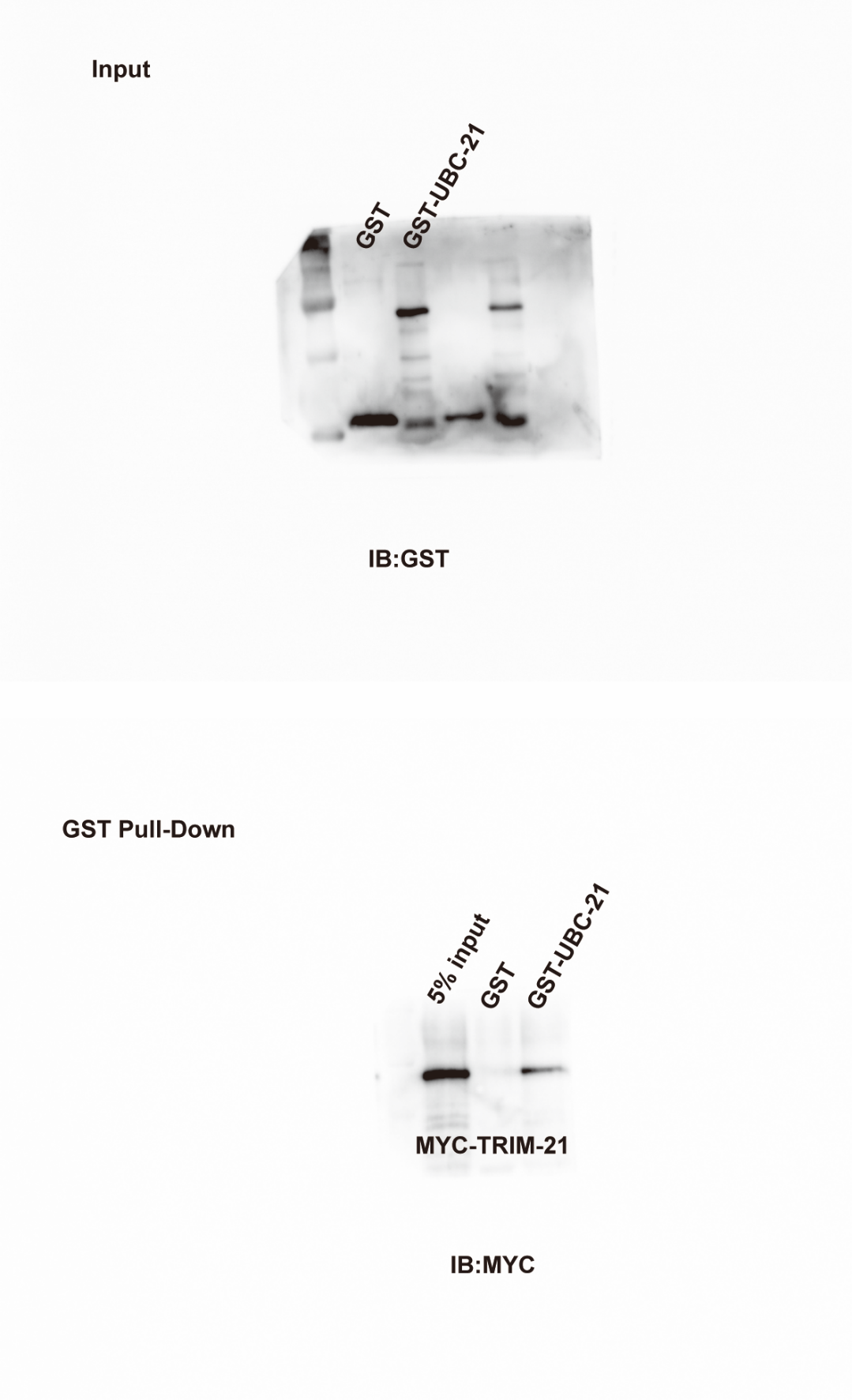


Figure 2E


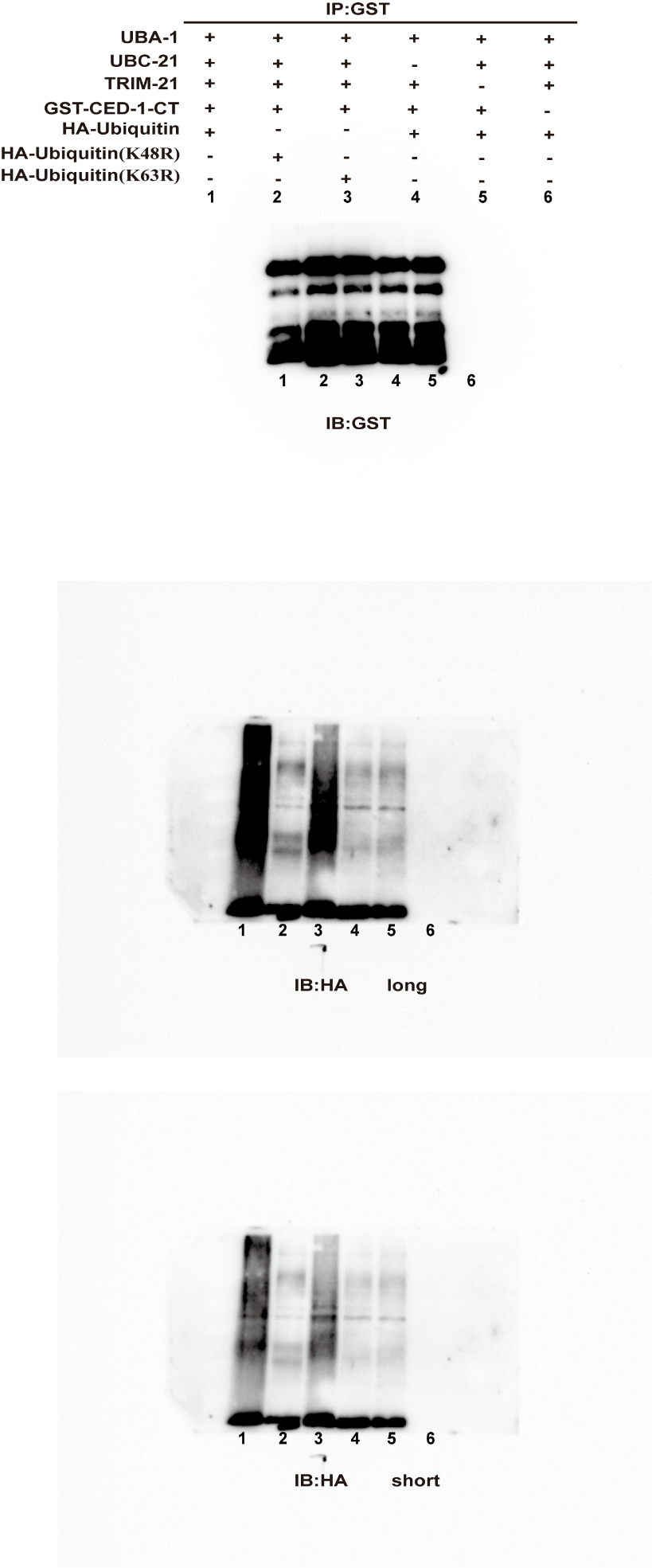


Figure 2F


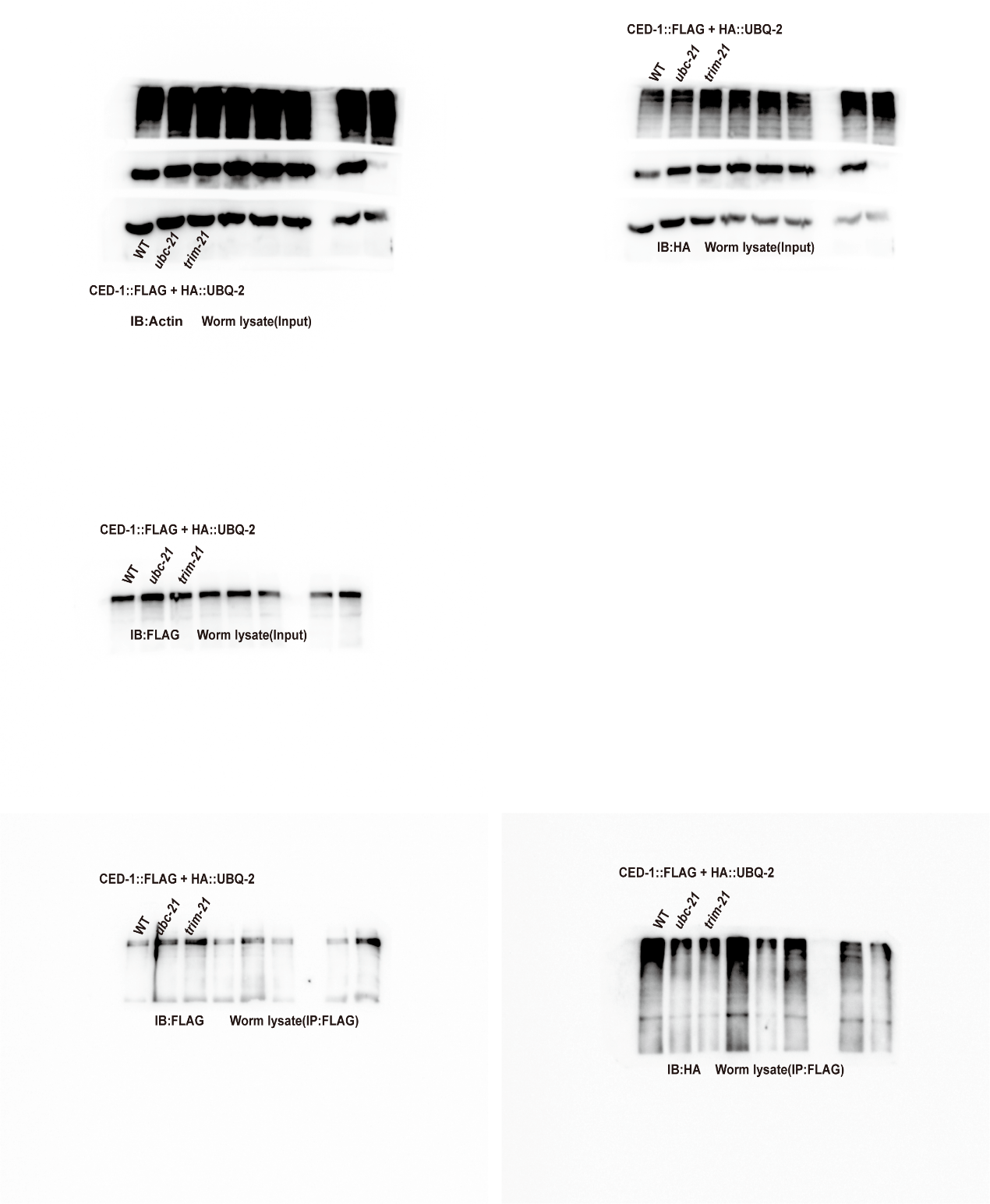


Figure 2G


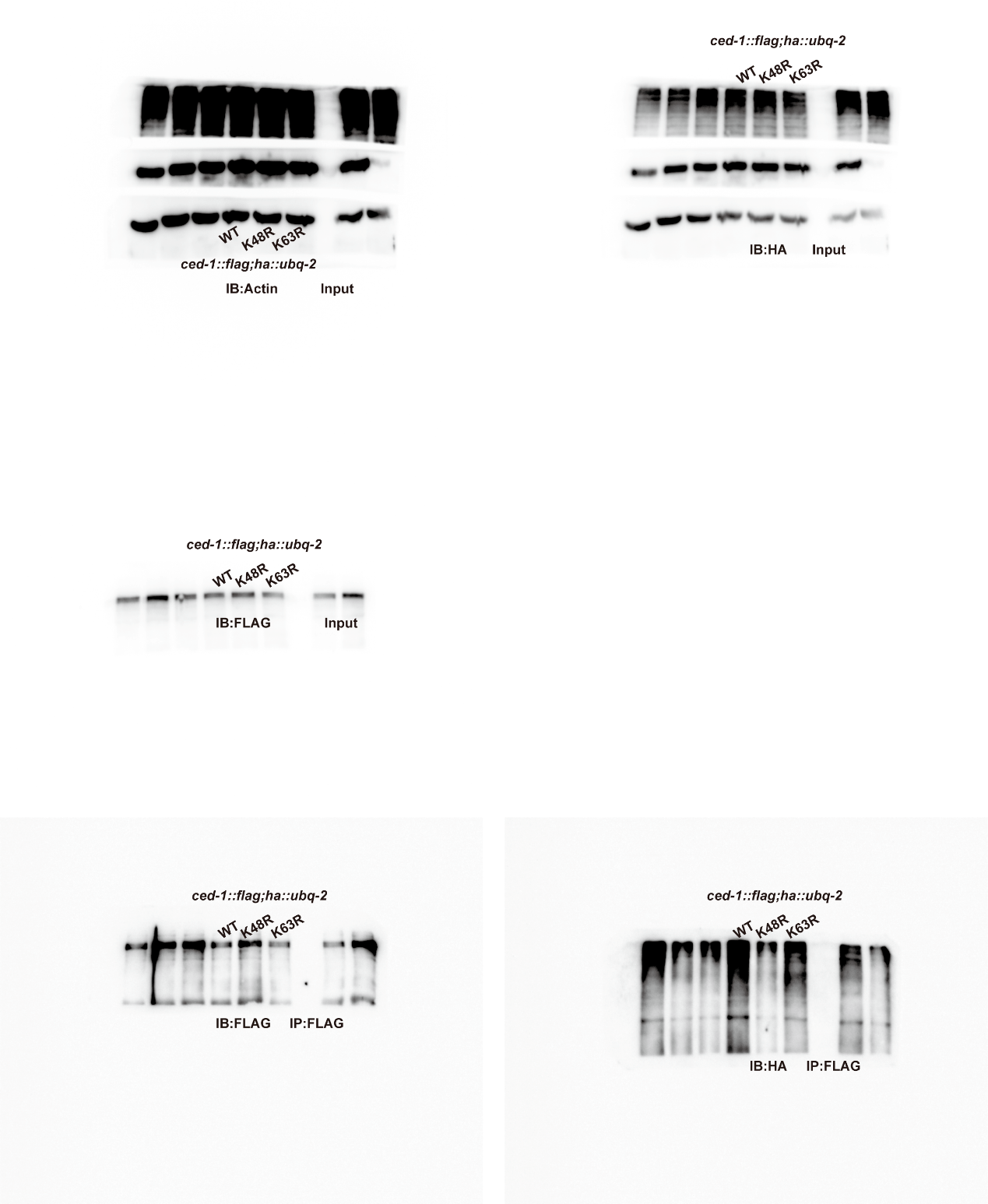


Figure 2I


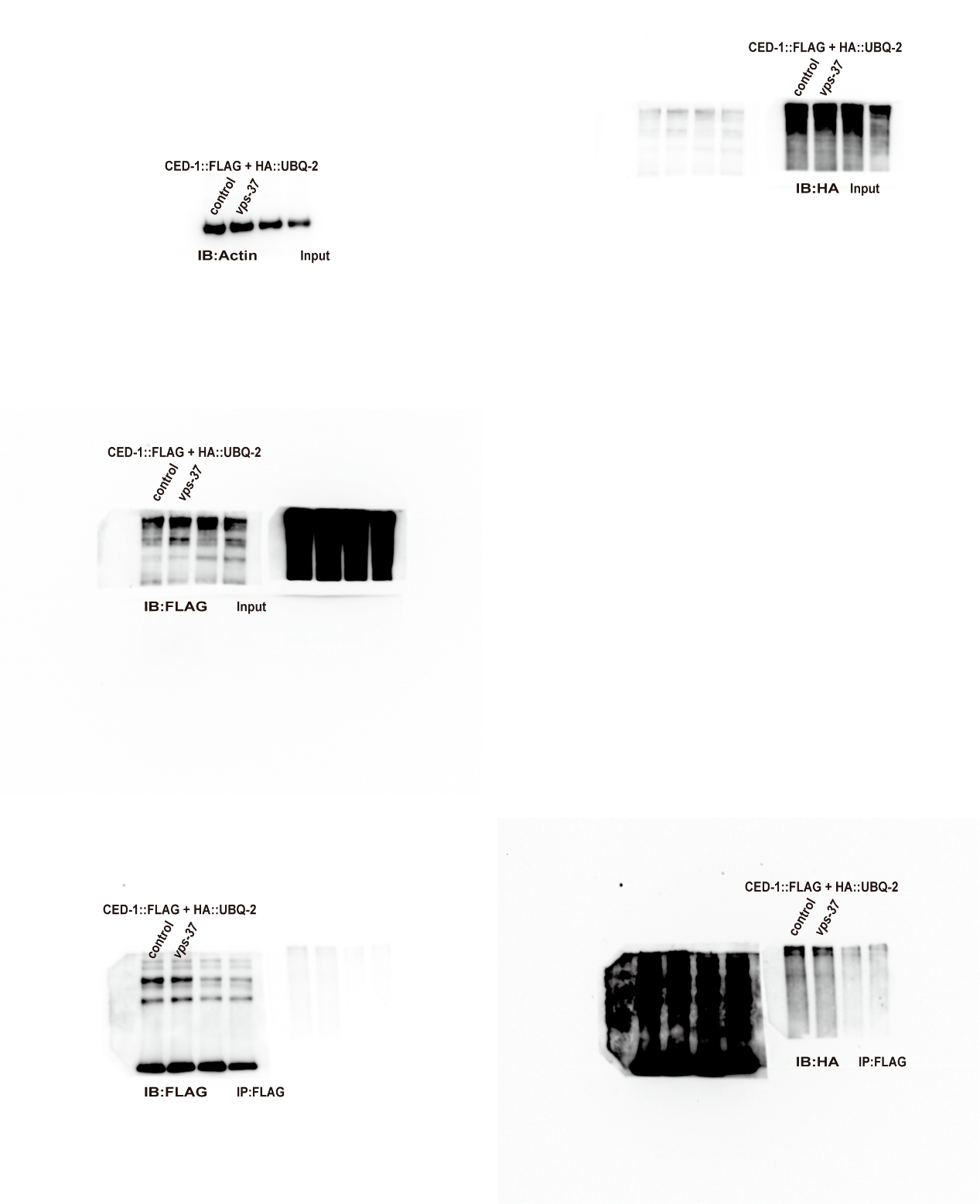

Supplement: Figure 2—source data 1. — Including uncropped Western blot images and raw statistics. [file elife-76436-fig2-data1.zip › Figure 2-Source Data 1/Figure 2 uncroppped blot with relevant bands.docx]

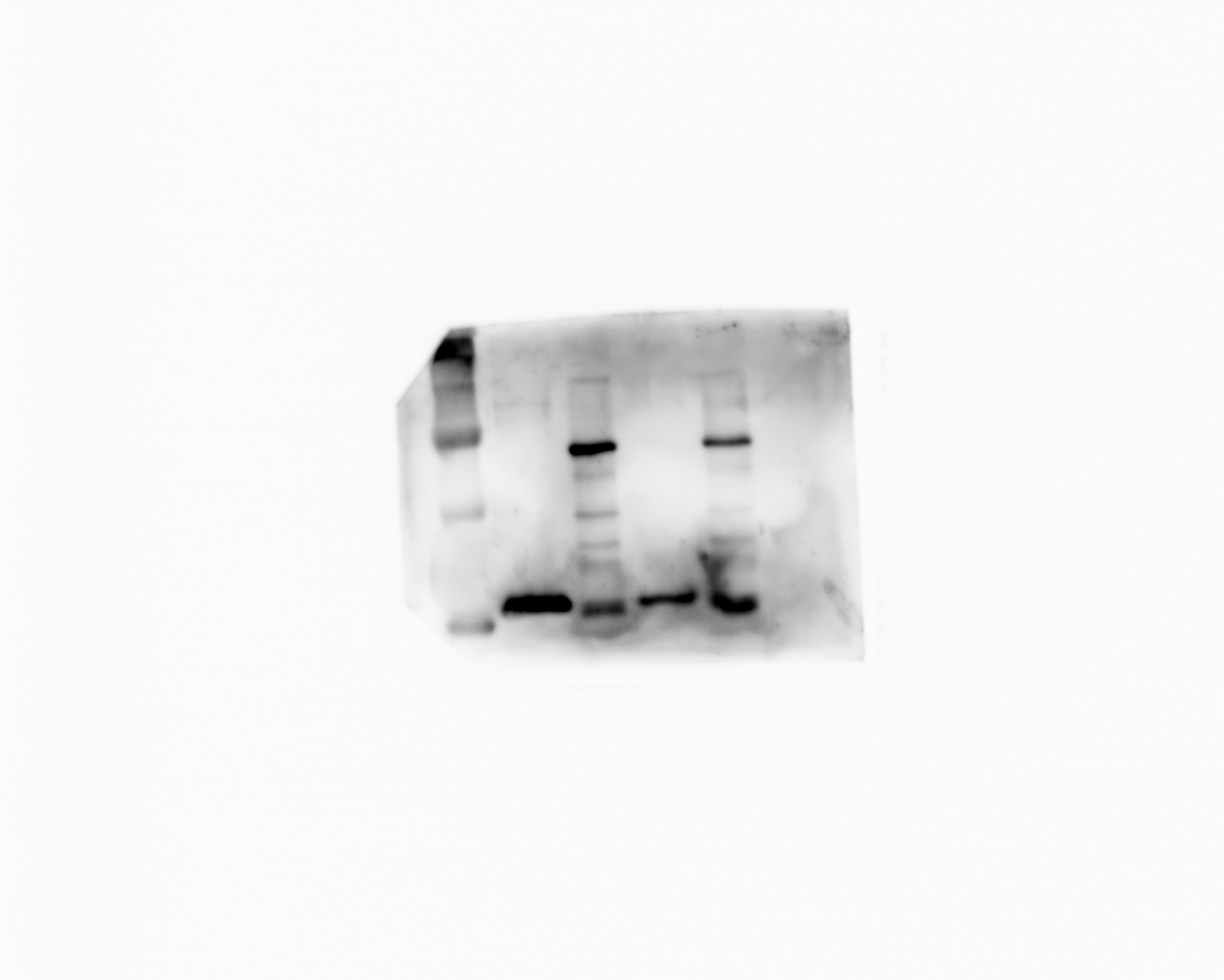

Supplement: Figure 2—source data 1. — Including uncropped Western blot images and raw statistics. [file elife-76436-fig2-data1.zip › Figure 2-Source Data 1/Figure 2B full raw unedited/IB-GST.tif]

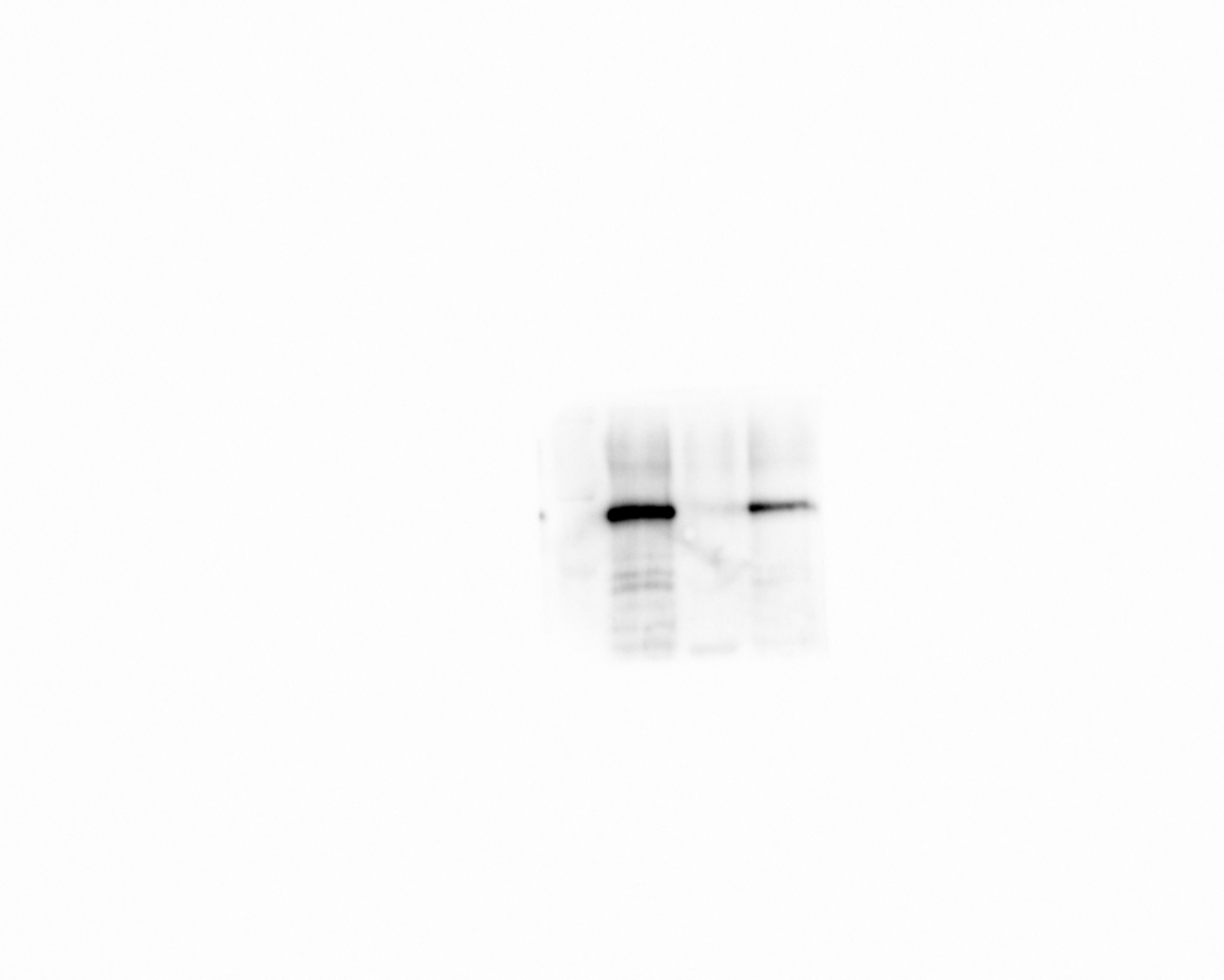

Supplement: Figure 2—source data 1. — Including uncropped Western blot images and raw statistics. [file elife-76436-fig2-data1.zip › Figure 2-Source Data 1/Figure 2B full raw unedited/IB-MYC.tif]

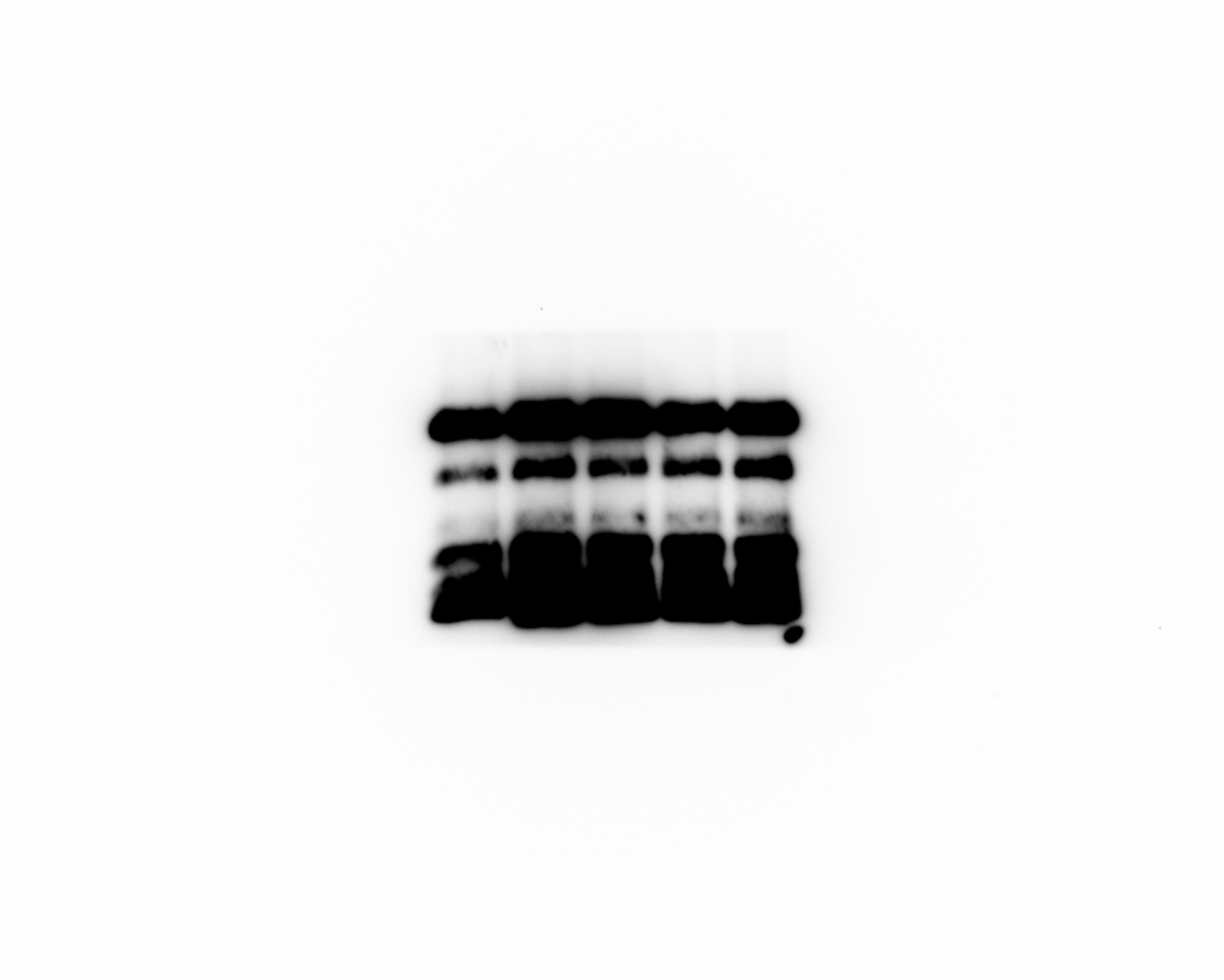

Supplement: Figure 2—source data 1. — Including uncropped Western blot images and raw statistics. [file elife-76436-fig2-data1.zip › Figure 2-Source Data 1/Figure 2E full raw unedited/IB-GST.tif]

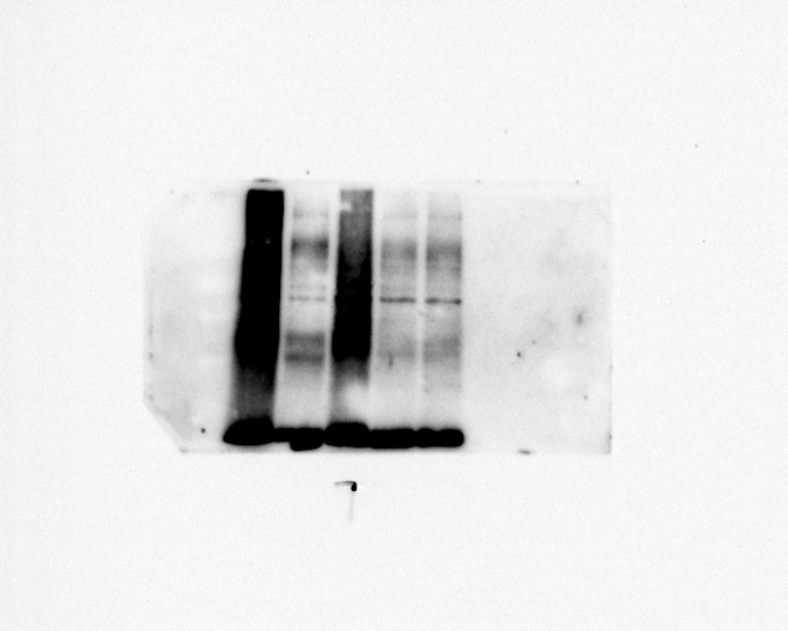

Supplement: Figure 2—source data 1. — Including uncropped Western blot images and raw statistics. [file elife-76436-fig2-data1.zip › Figure 2-Source Data 1/Figure 2E full raw unedited/IB-HA-long.tif]

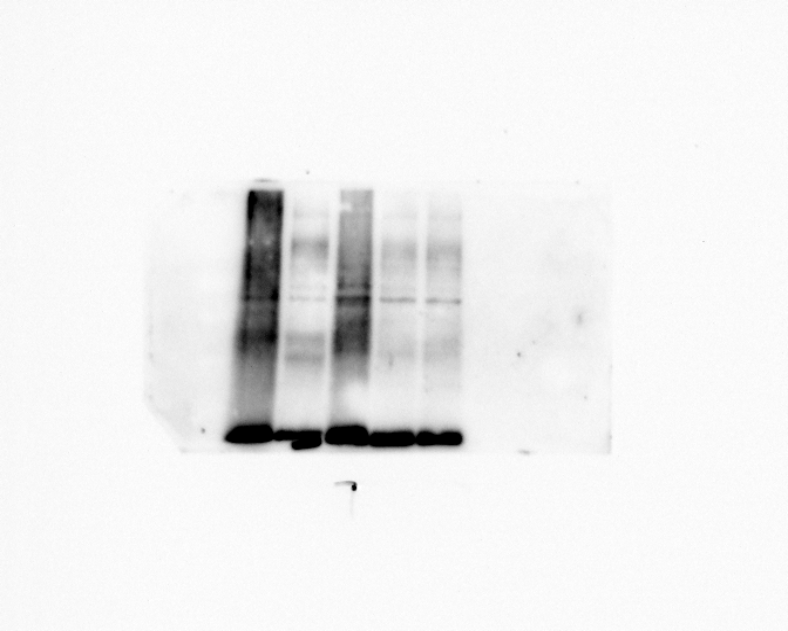

Supplement: Figure 2—source data 1. — Including uncropped Western blot images and raw statistics. [file elife-76436-fig2-data1.zip › Figure 2-Source Data 1/Figure 2E full raw unedited/IB-HA-short.tif]

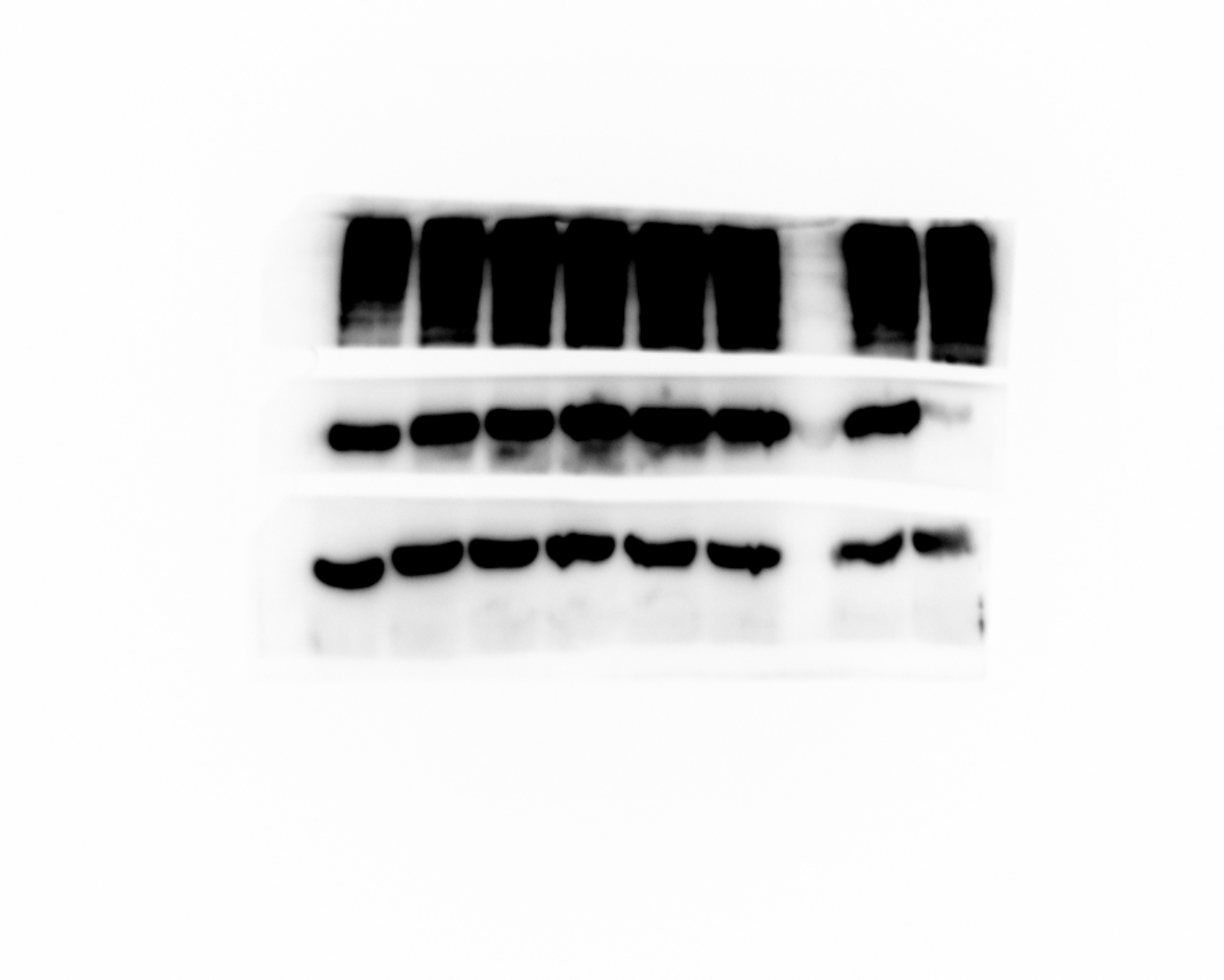

Supplement: Figure 2—source data 1. — Including uncropped Western blot images and raw statistics. [file elife-76436-fig2-data1.zip › Figure 2-Source Data 1/Figure 2F full raw unedited/Input-IB-Actin.tif]

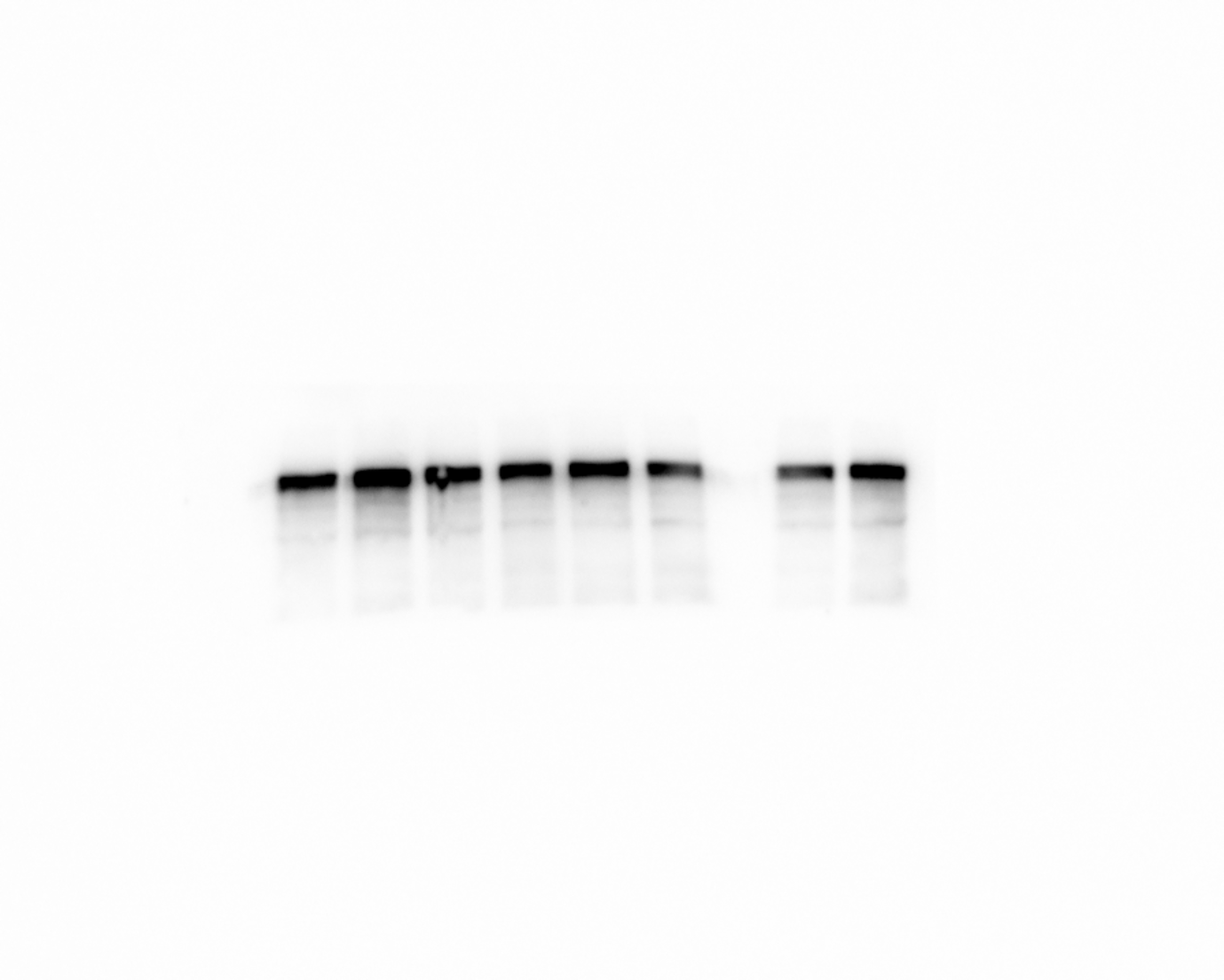

Supplement: Figure 2—source data 1. — Including uncropped Western blot images and raw statistics. [file elife-76436-fig2-data1.zip › Figure 2-Source Data 1/Figure 2F full raw unedited/Input-IB-FLAG.tif]

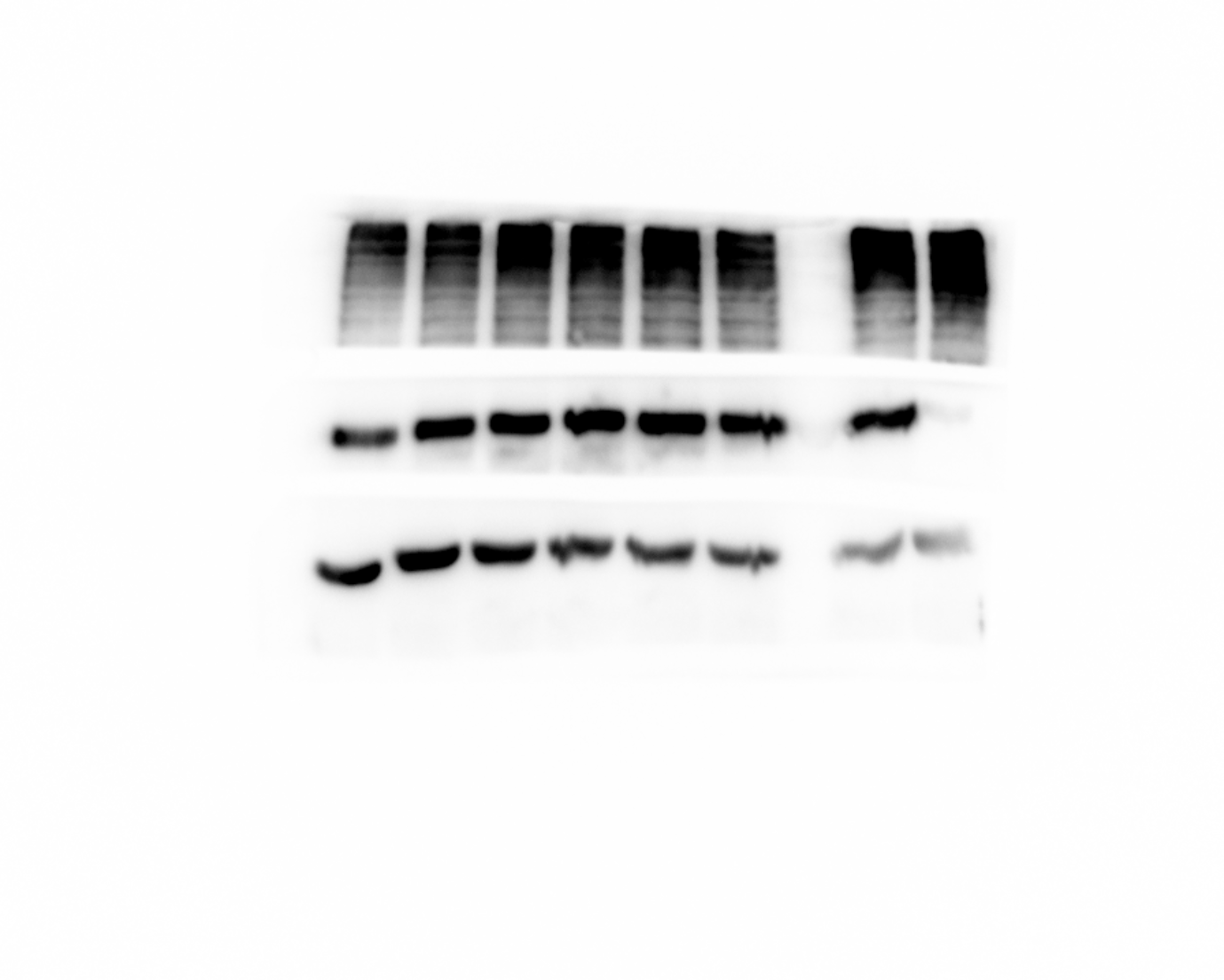

Supplement: Figure 2—source data 1. — Including uncropped Western blot images and raw statistics. [file elife-76436-fig2-data1.zip › Figure 2-Source Data 1/Figure 2F full raw unedited/Input-IB-HA.tif]

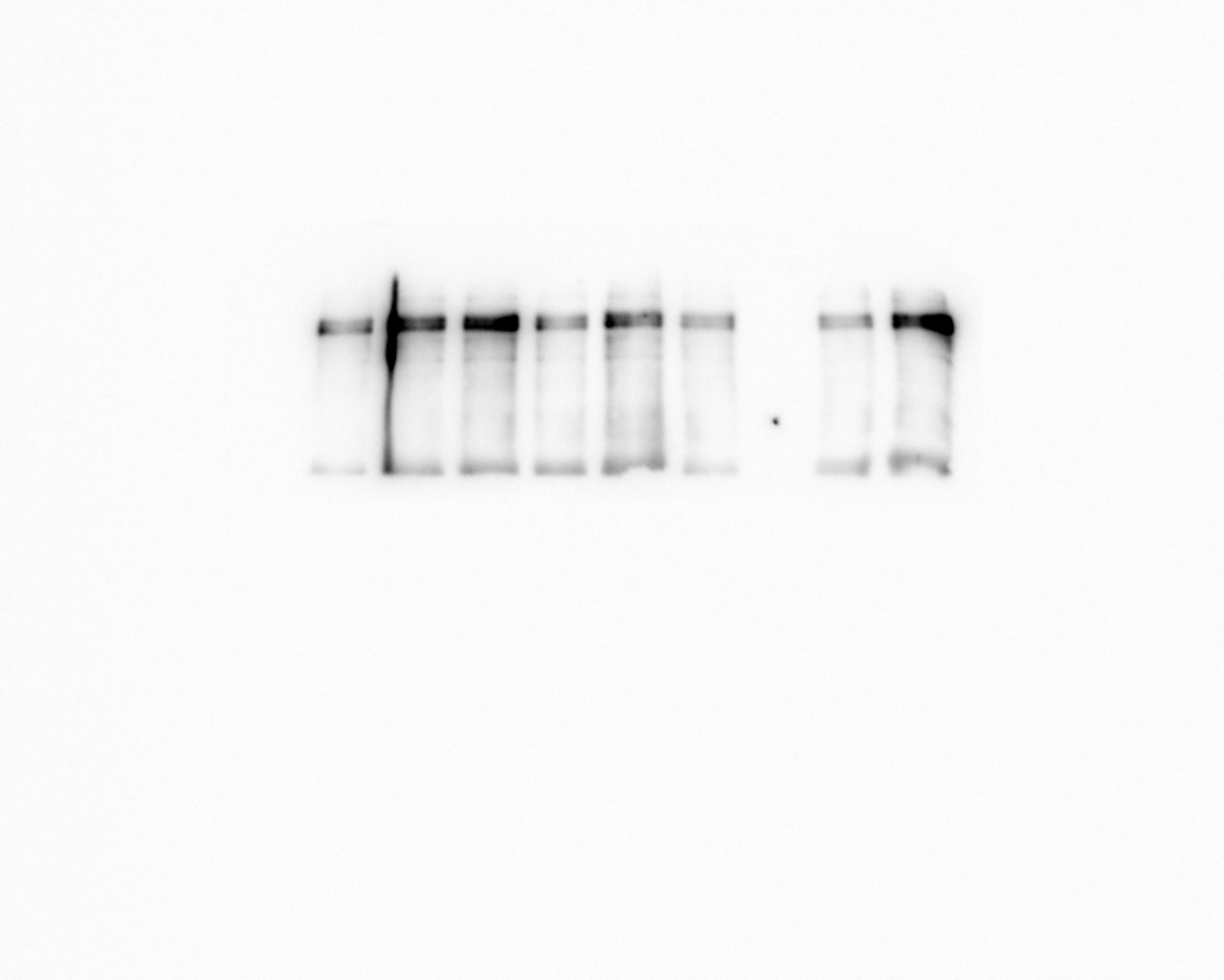

Supplement: Figure 2—source data 1. — Including uncropped Western blot images and raw statistics. [file elife-76436-fig2-data1.zip › Figure 2-Source Data 1/Figure 2F full raw unedited/IP-IB-FLAG.tif]

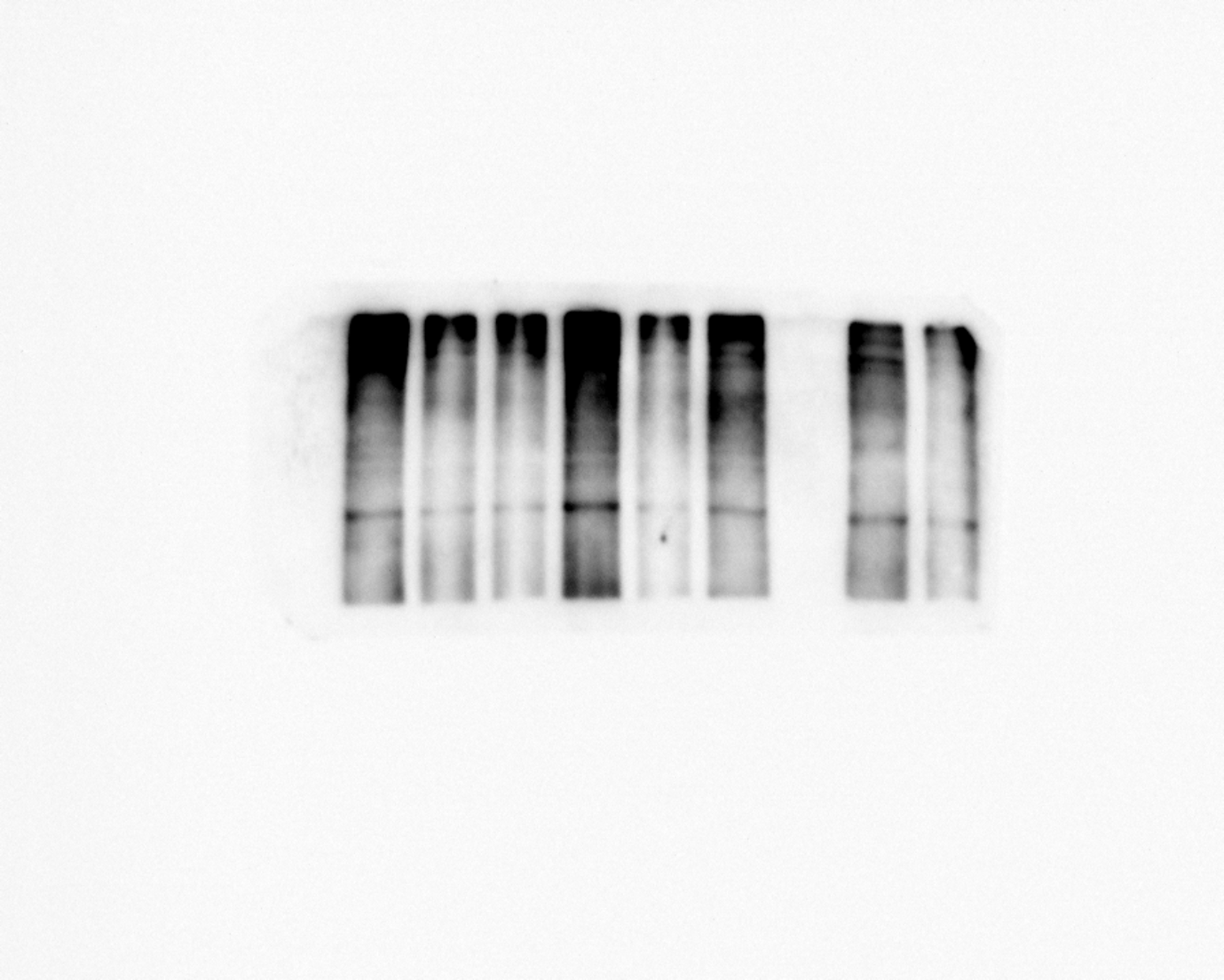

Supplement: Figure 2—source data 1. — Including uncropped Western blot images and raw statistics. [file elife-76436-fig2-data1.zip › Figure 2-Source Data 1/Figure 2F full raw unedited/IP-IB-HA.tif]

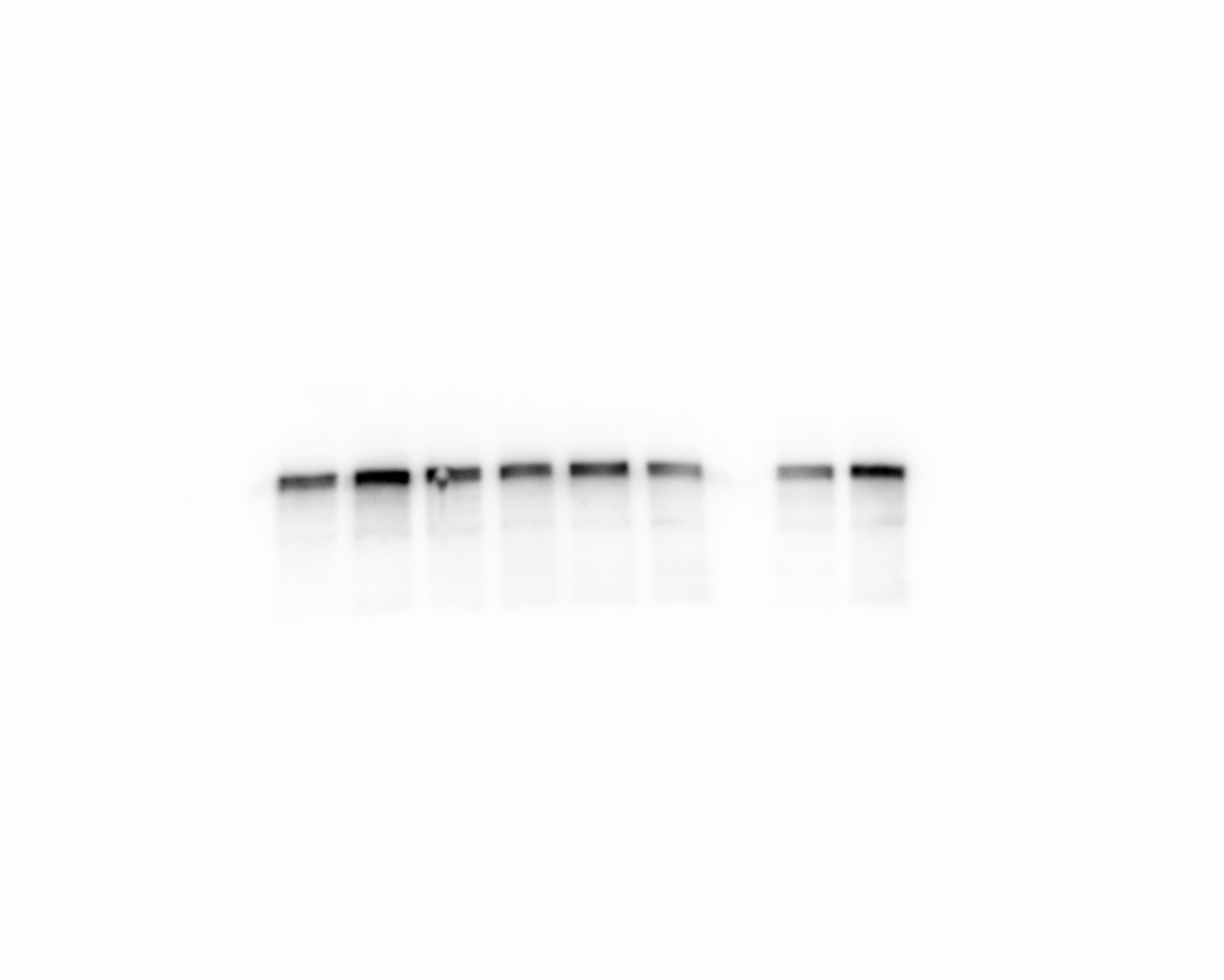

Supplement: Figure 2—source data 1. — Including uncropped Western blot images and raw statistics. [file elife-76436-fig2-data1.zip › Figure 2-Source Data 1/Figure 2G full raw unedited/Input-IB-FLAG.tif]

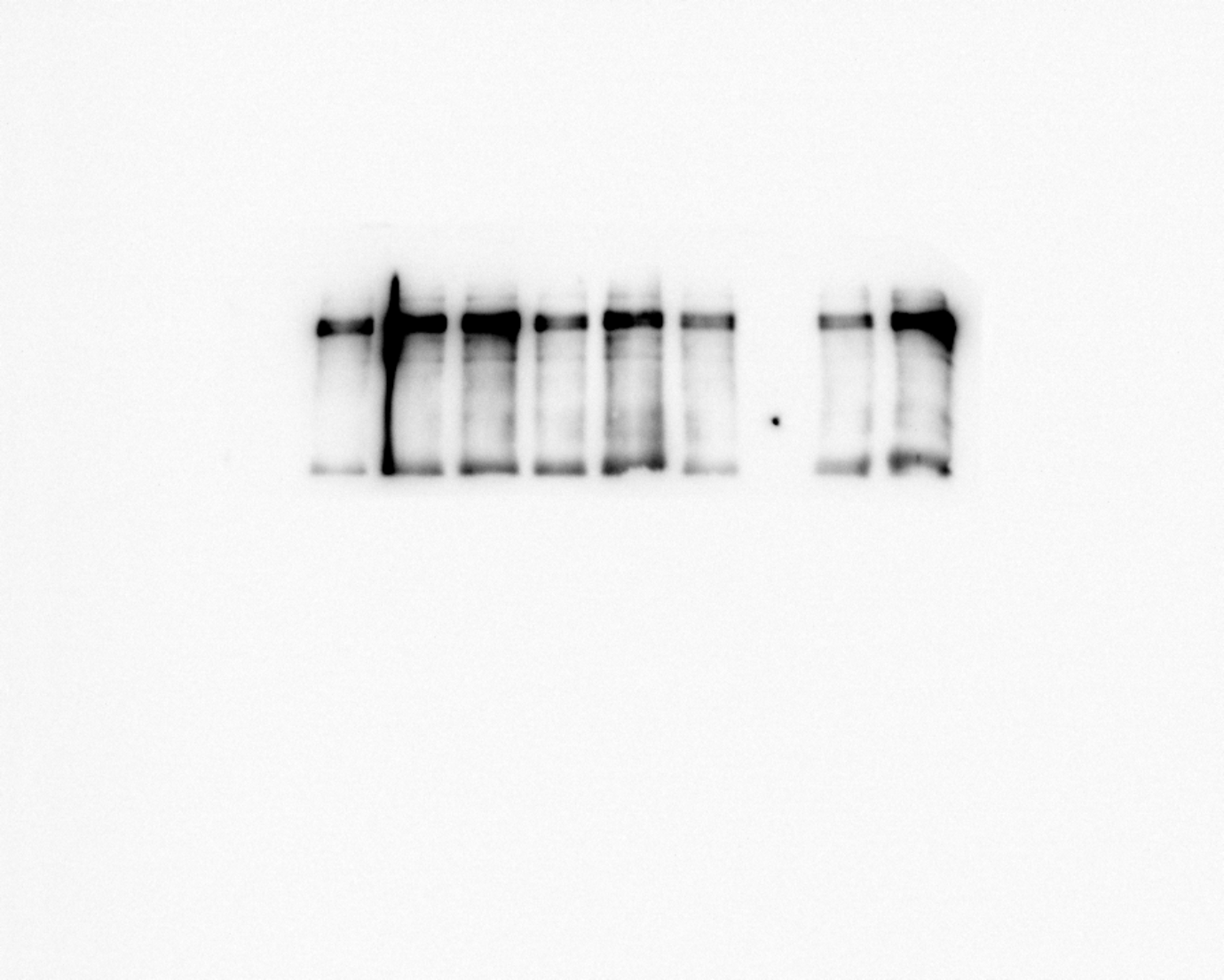

Supplement: Figure 2—source data 1. — Including uncropped Western blot images and raw statistics. [file elife-76436-fig2-data1.zip › Figure 2-Source Data 1/Figure 2G full raw unedited/IP-IB-FLAG.tif]

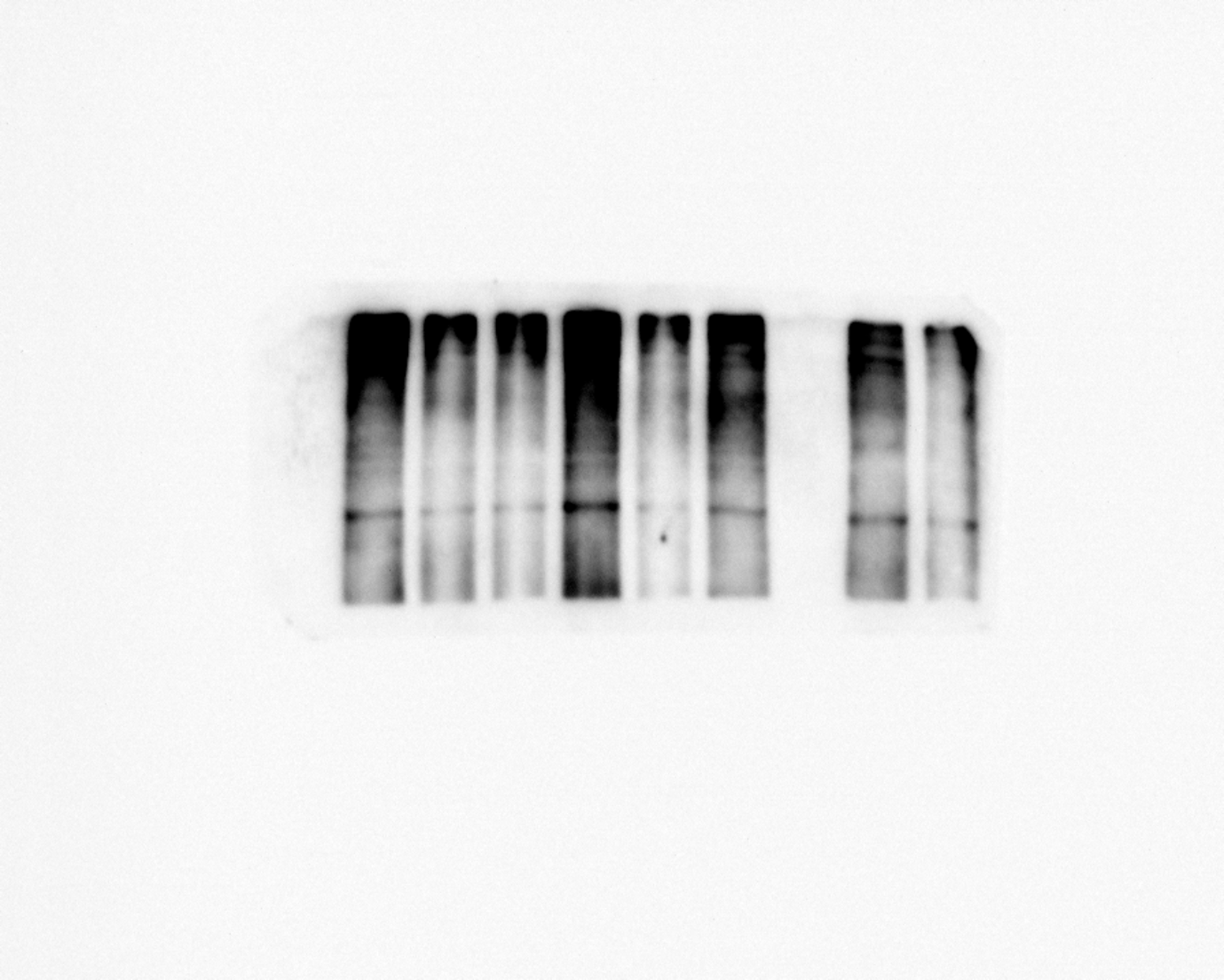

Supplement: Figure 2—source data 1. — Including uncropped Western blot images and raw statistics. [file elife-76436-fig2-data1.zip › Figure 2-Source Data 1/Figure 2G full raw unedited/IP-IB-HA.tif]

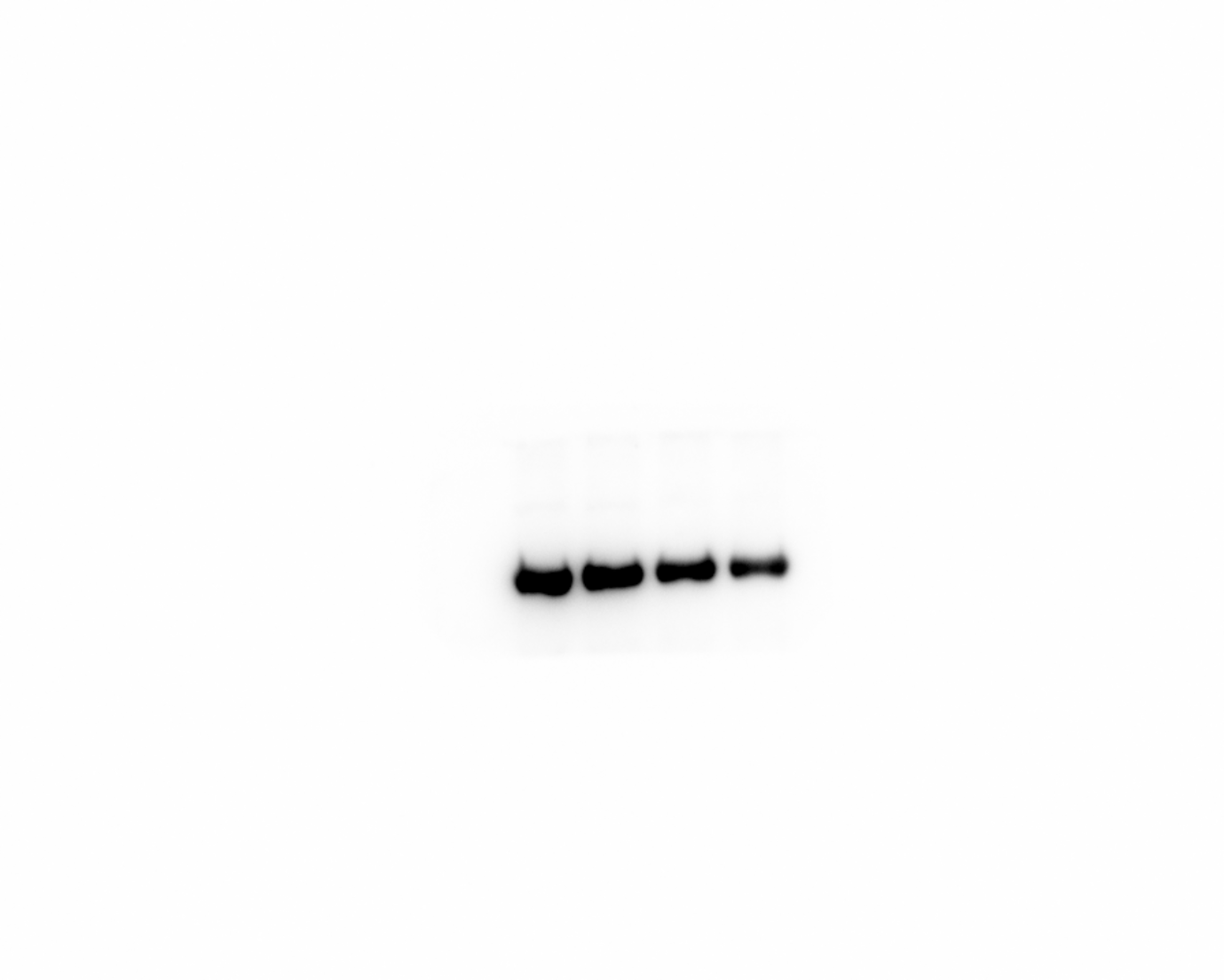

Supplement: Figure 2—source data 1. — Including uncropped Western blot images and raw statistics. [file elife-76436-fig2-data1.zip › Figure 2-Source Data 1/Figure 2I full raw unedited/Input-IB-Actin.tif]

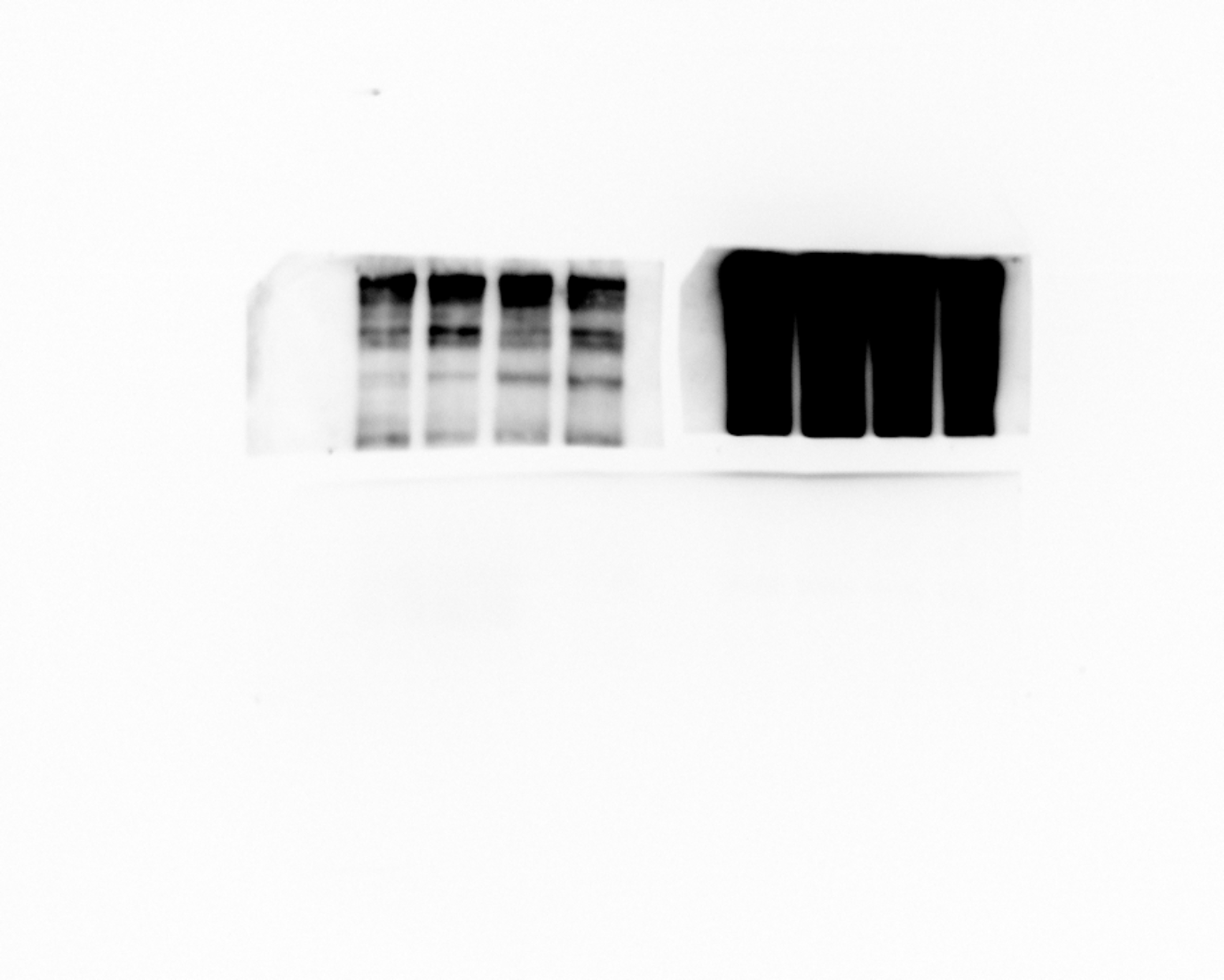

Supplement: Figure 2—source data 1. — Including uncropped Western blot images and raw statistics. [file elife-76436-fig2-data1.zip › Figure 2-Source Data 1/Figure 2I full raw unedited/Input-IB-FLAG.tif]

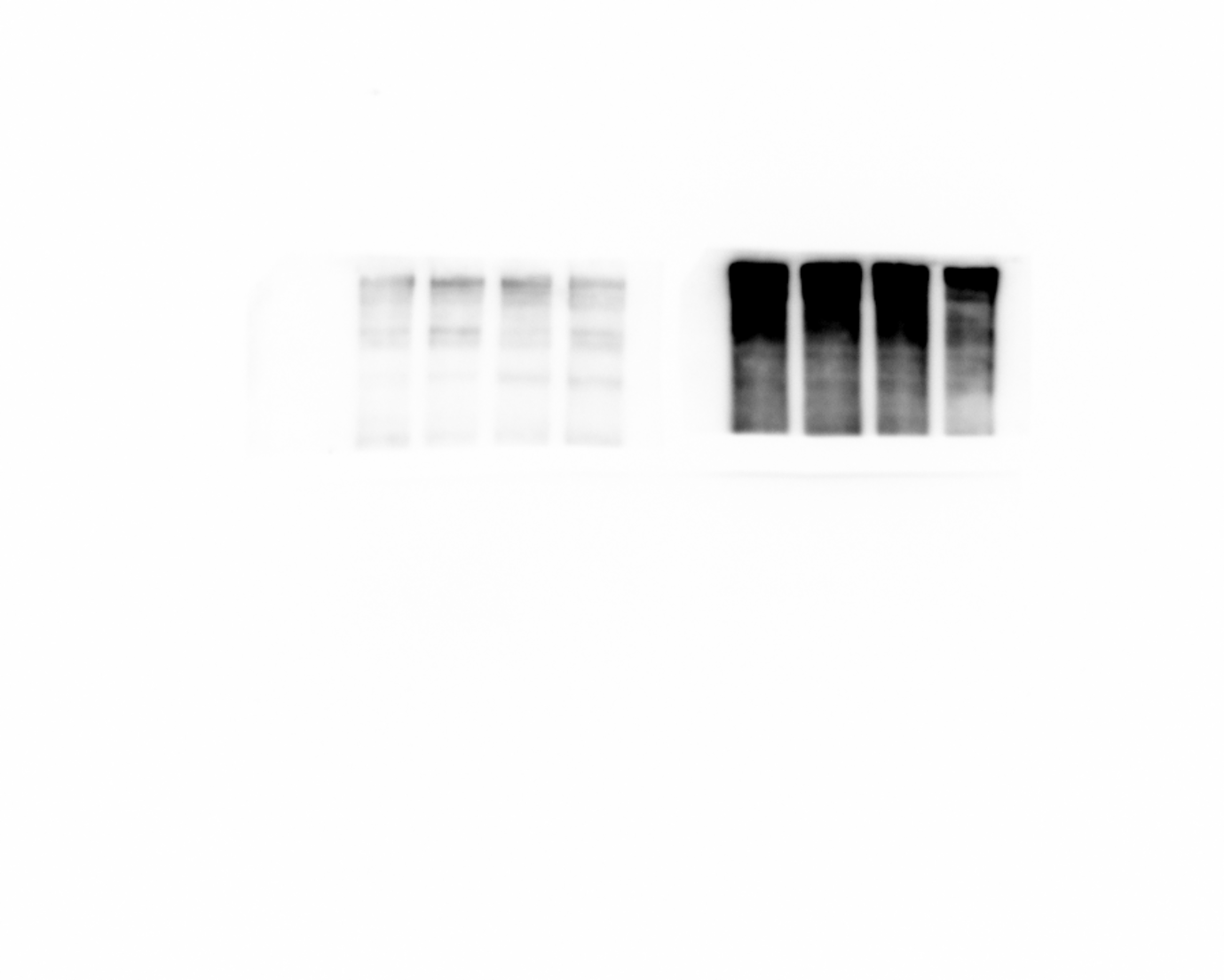

Supplement: Figure 2—source data 1. — Including uncropped Western blot images and raw statistics. [file elife-76436-fig2-data1.zip › Figure 2-Source Data 1/Figure 2I full raw unedited/Input-IB-HA.tif]

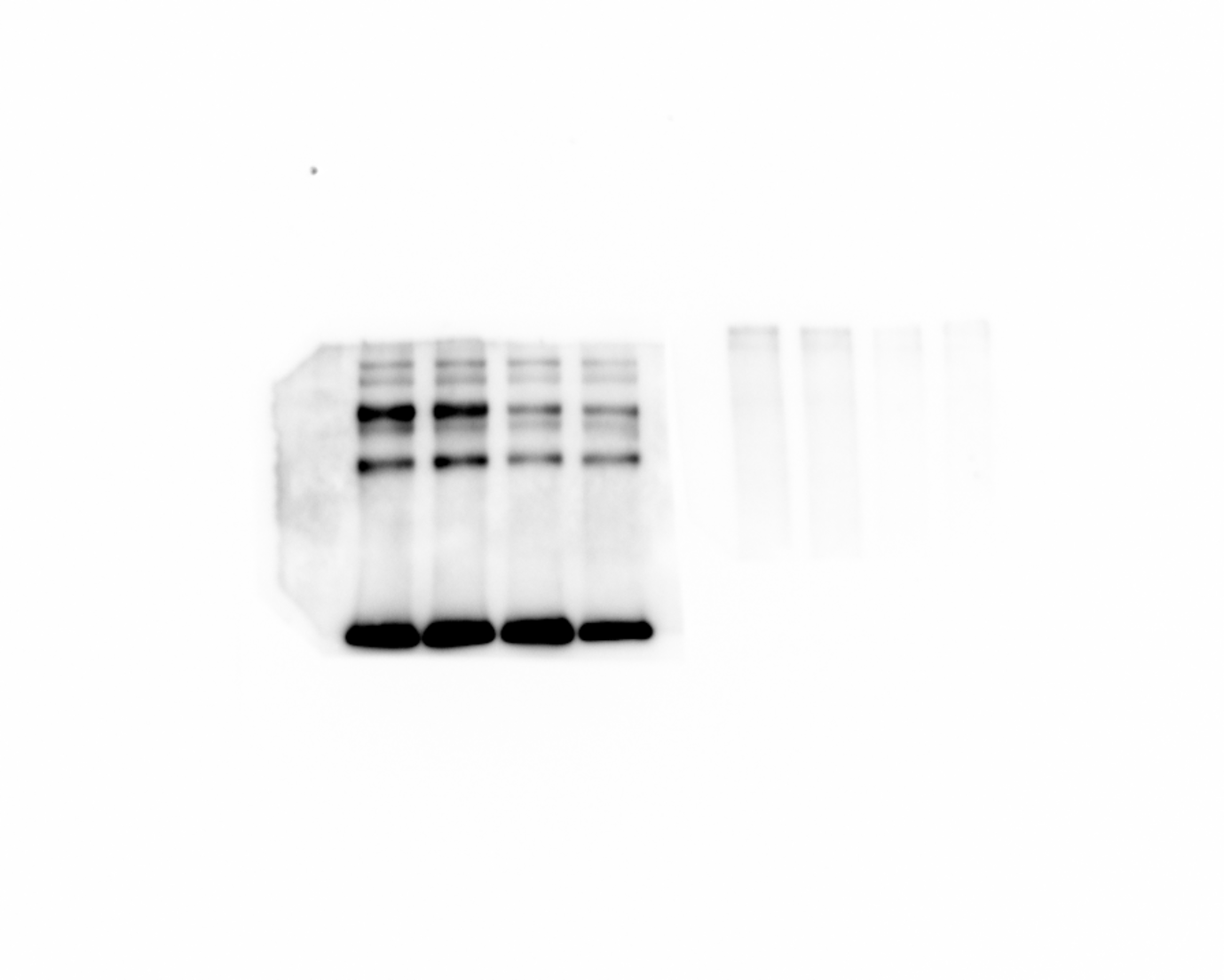

Supplement: Figure 2—source data 1. — Including uncropped Western blot images and raw statistics. [file elife-76436-fig2-data1.zip › Figure 2-Source Data 1/Figure 2I full raw unedited/IP-IB-FLAG.tif]

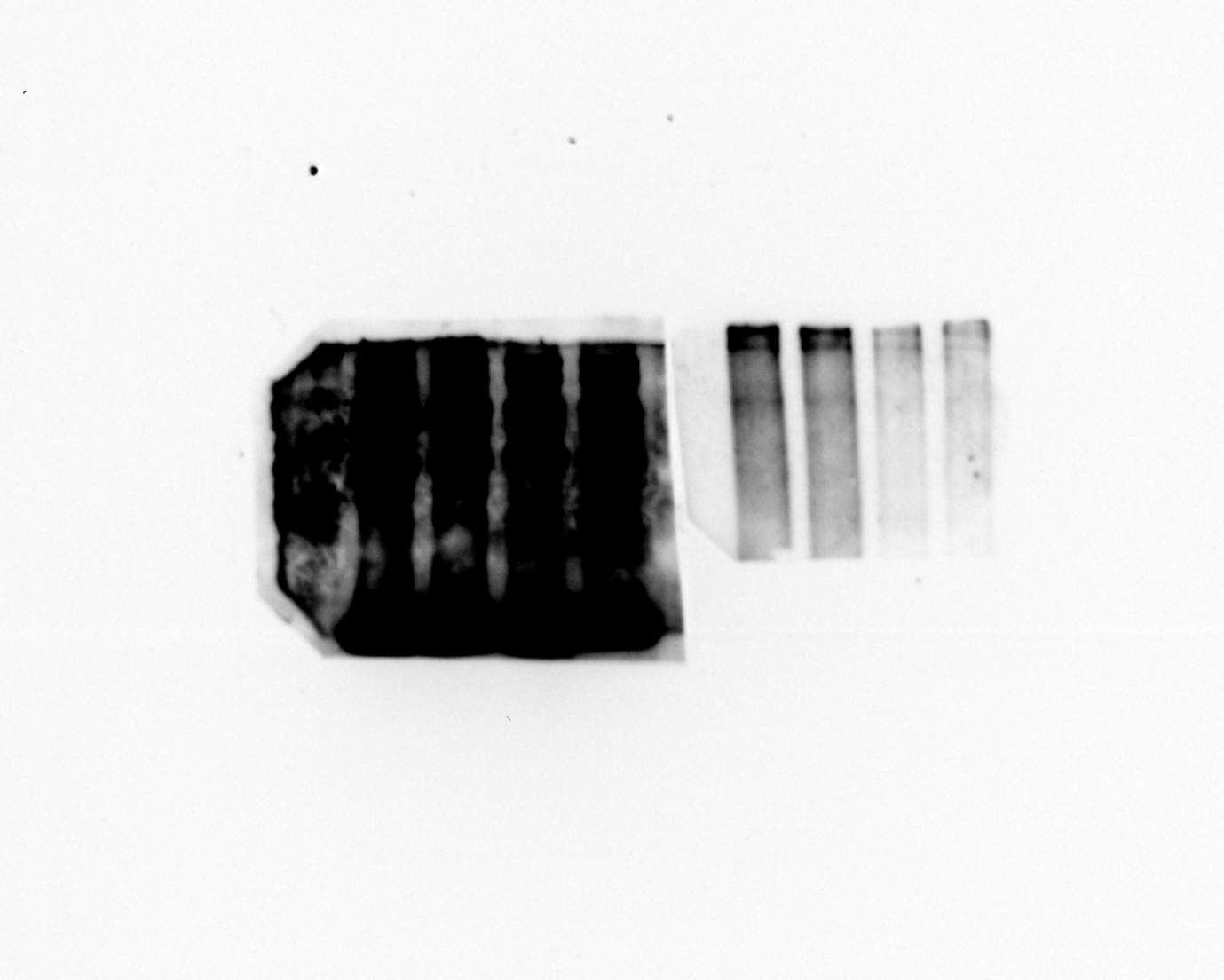

Supplement: Figure 2—source data 1. — Including uncropped Western blot images and raw statistics. [file elife-76436-fig2-data1.zip › Figure 2-Source Data 1/Figure 2I full raw unedited/IP-IB-HA.tif]

Figure 3A


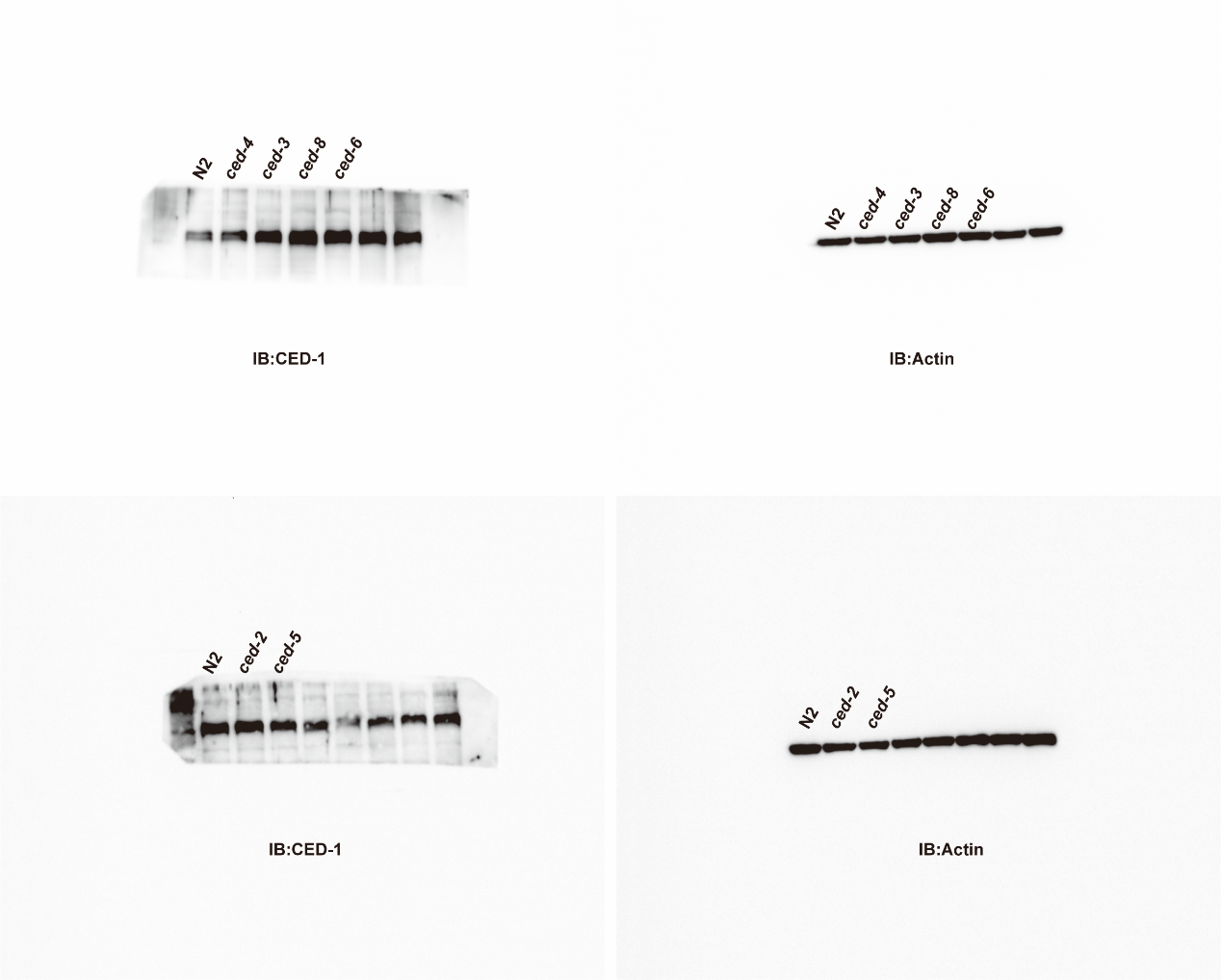


Figure 3B


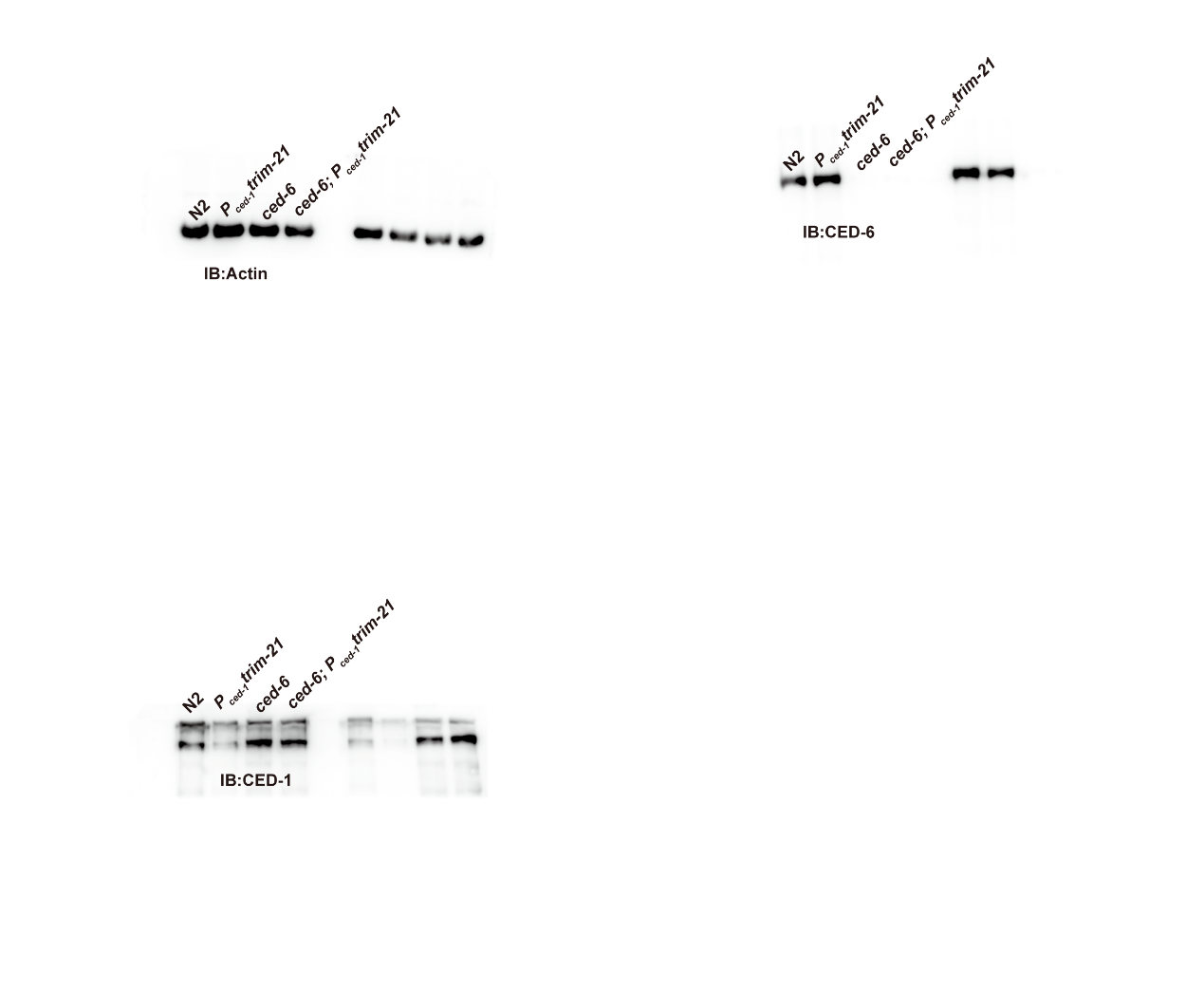


Figure 3C


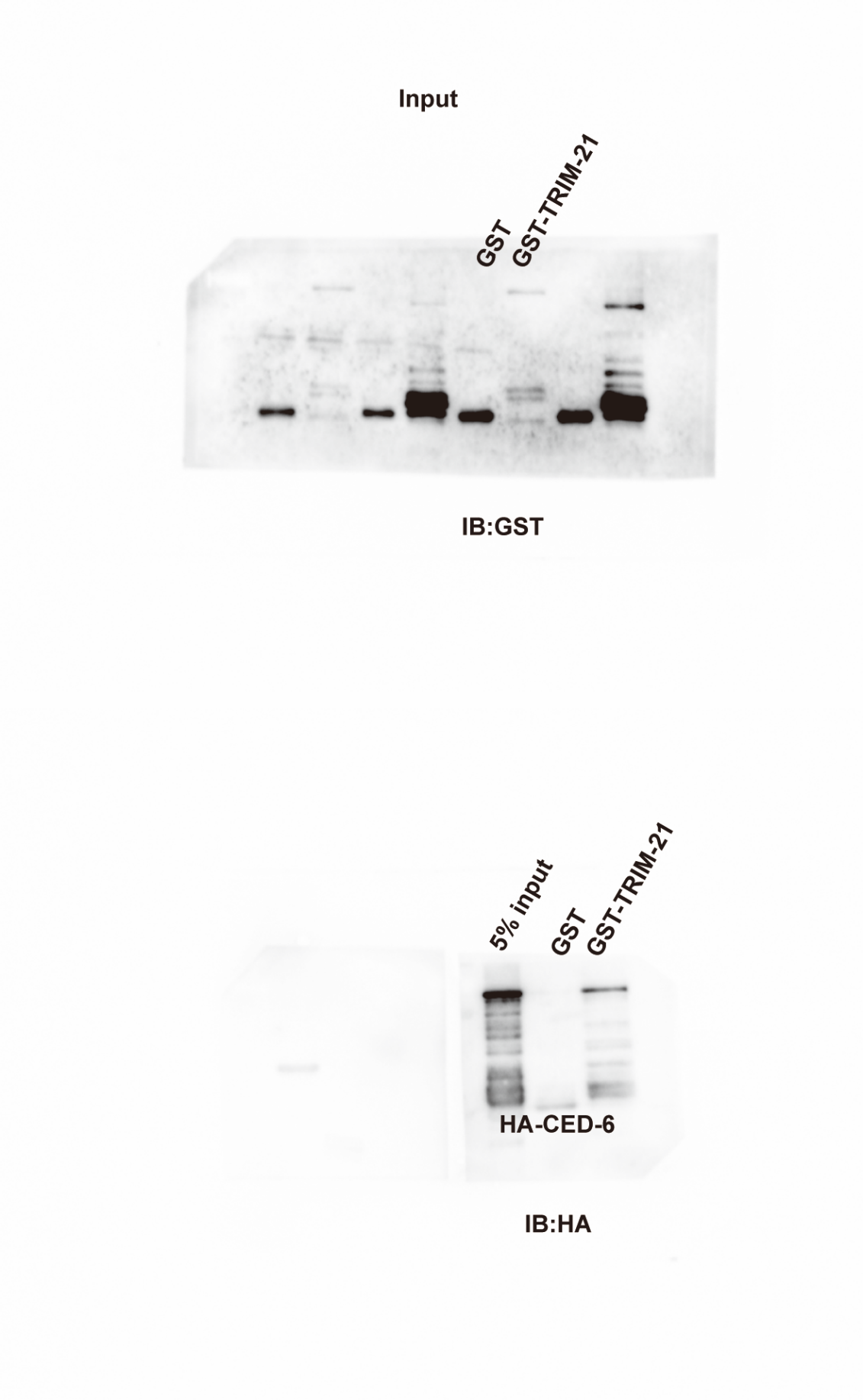


Figure 3D


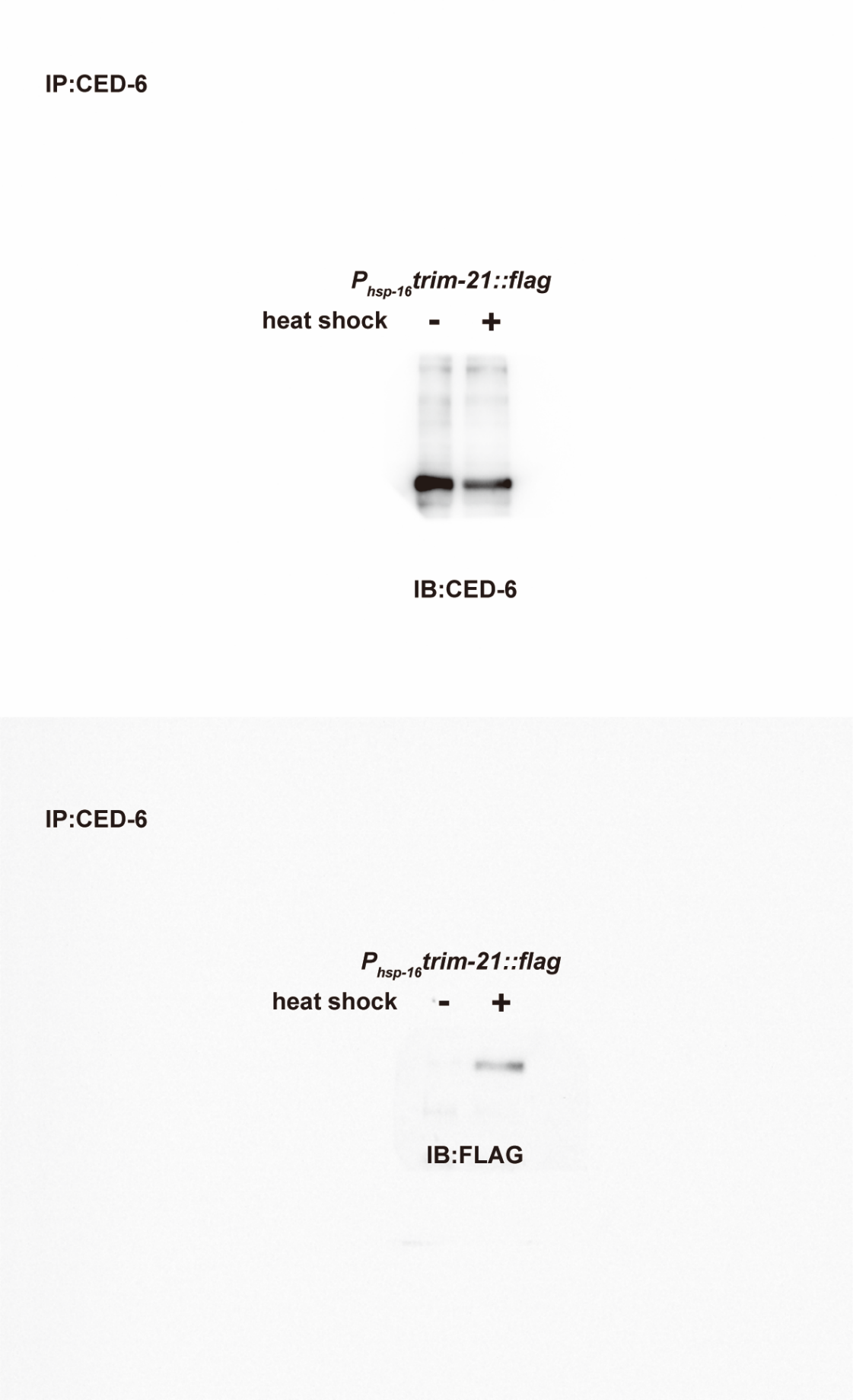


Figure 3E


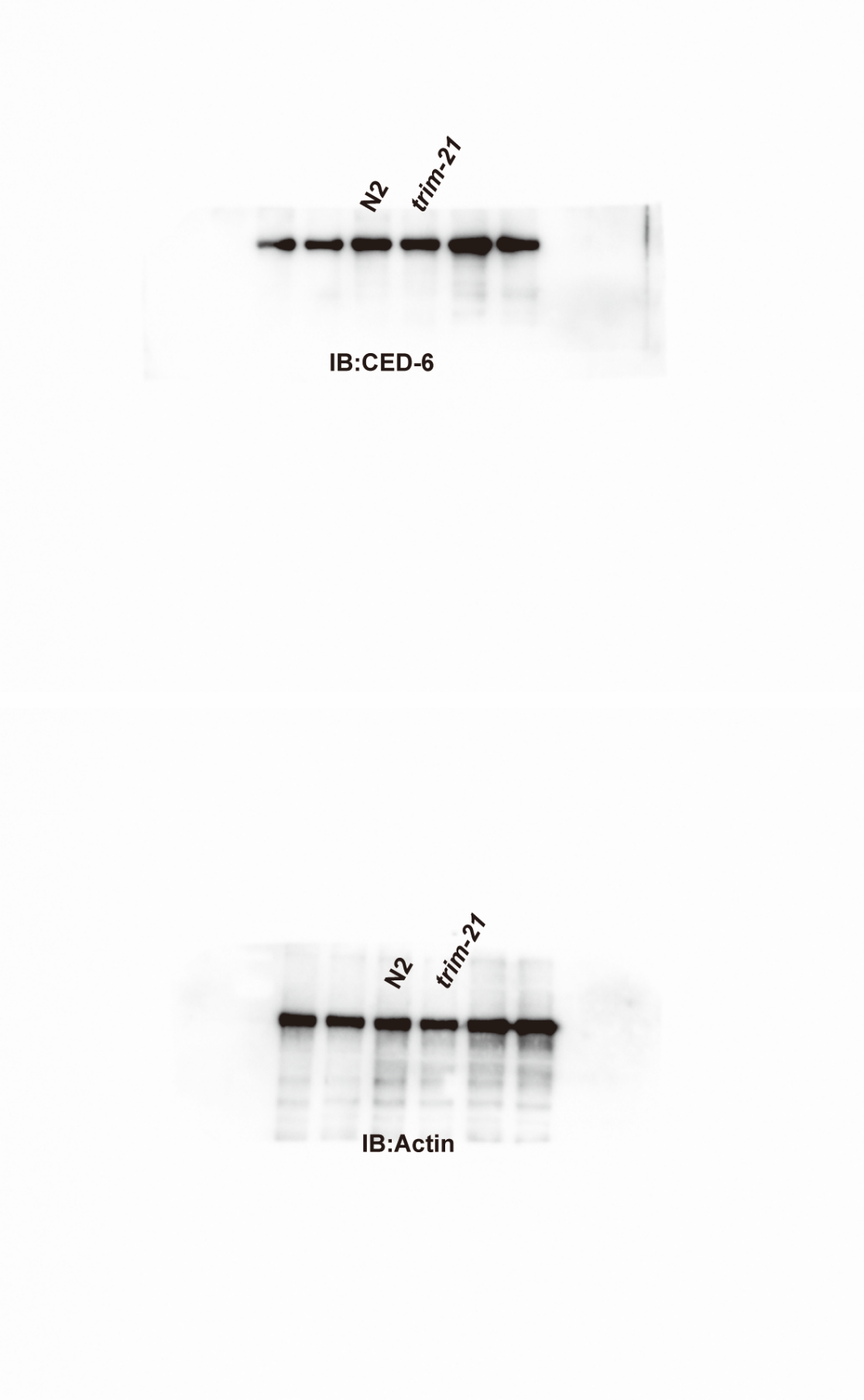


Figure 3H


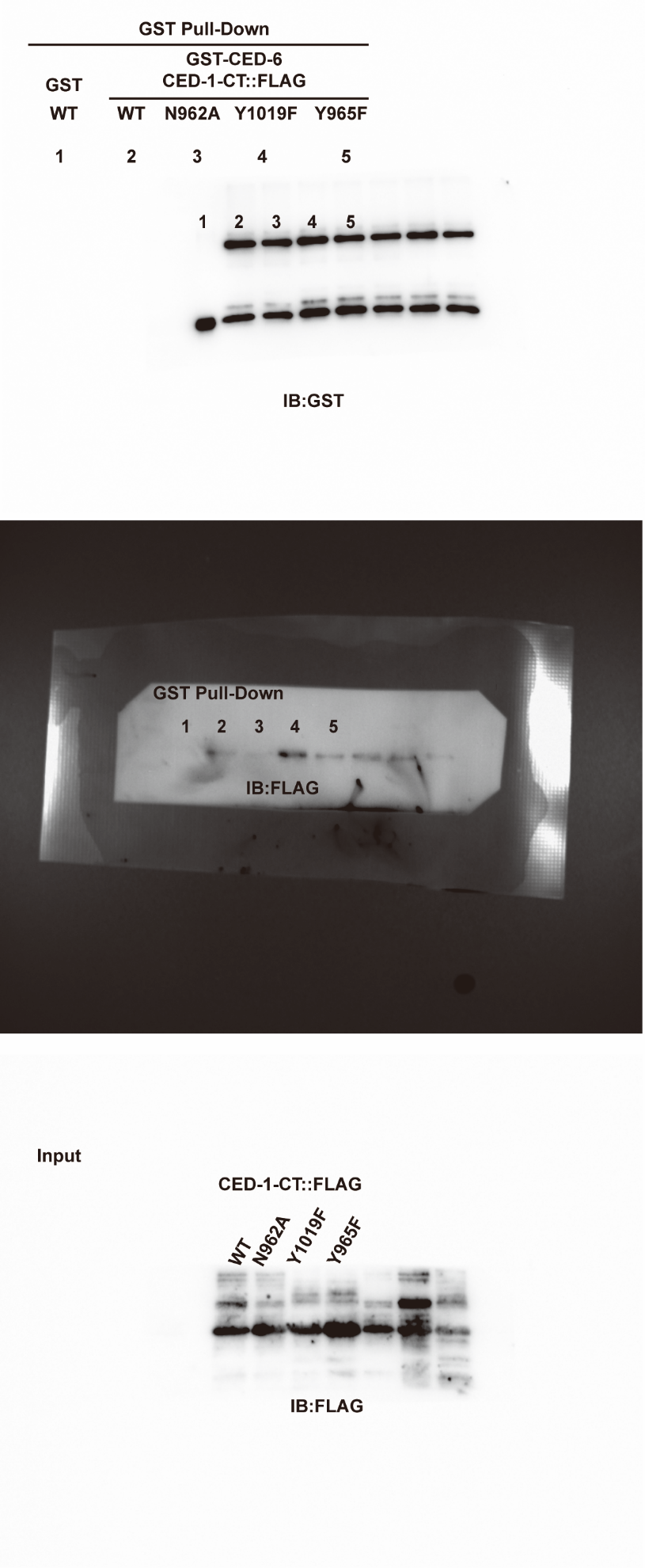


Figure 3I


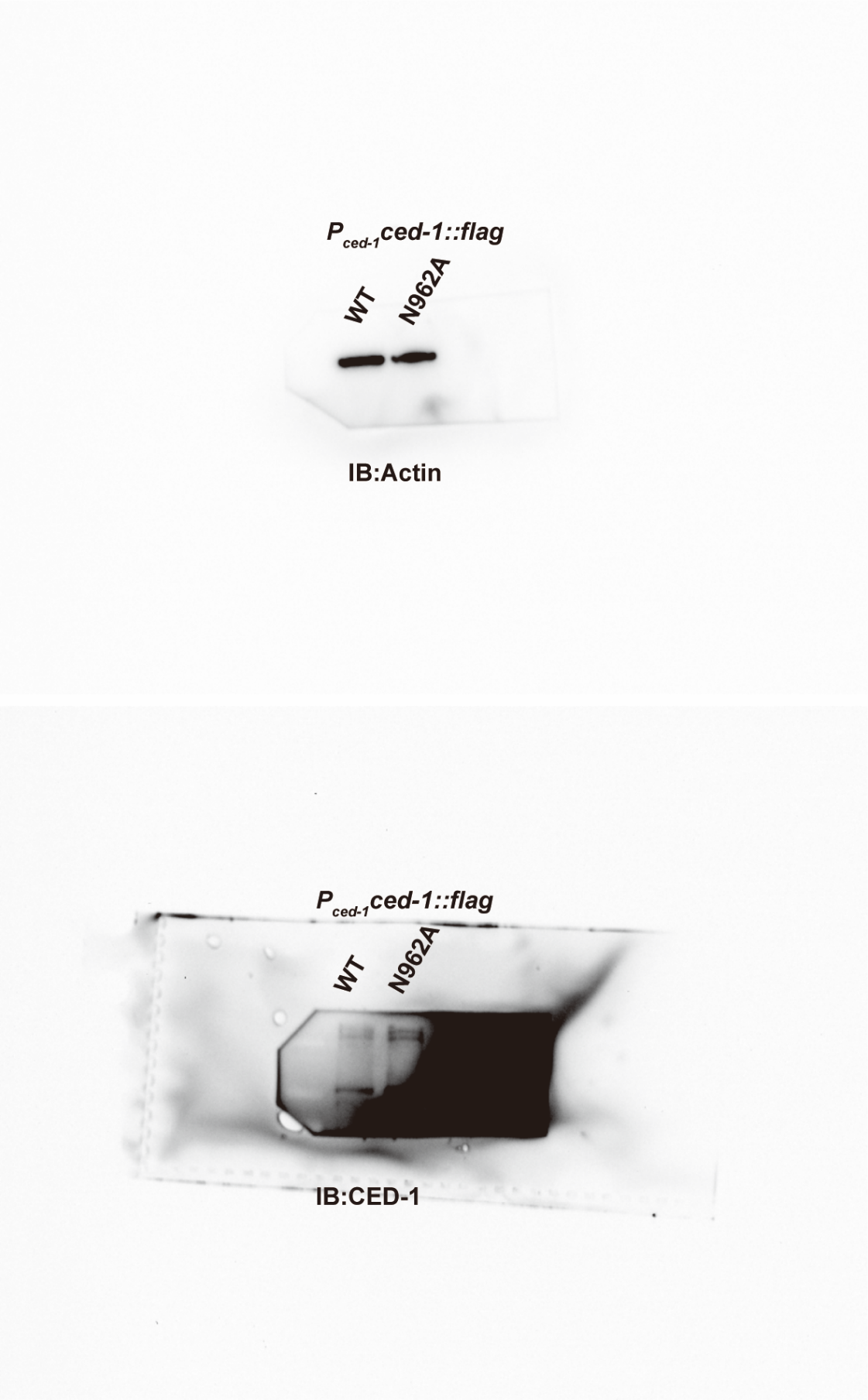


Figure 3J


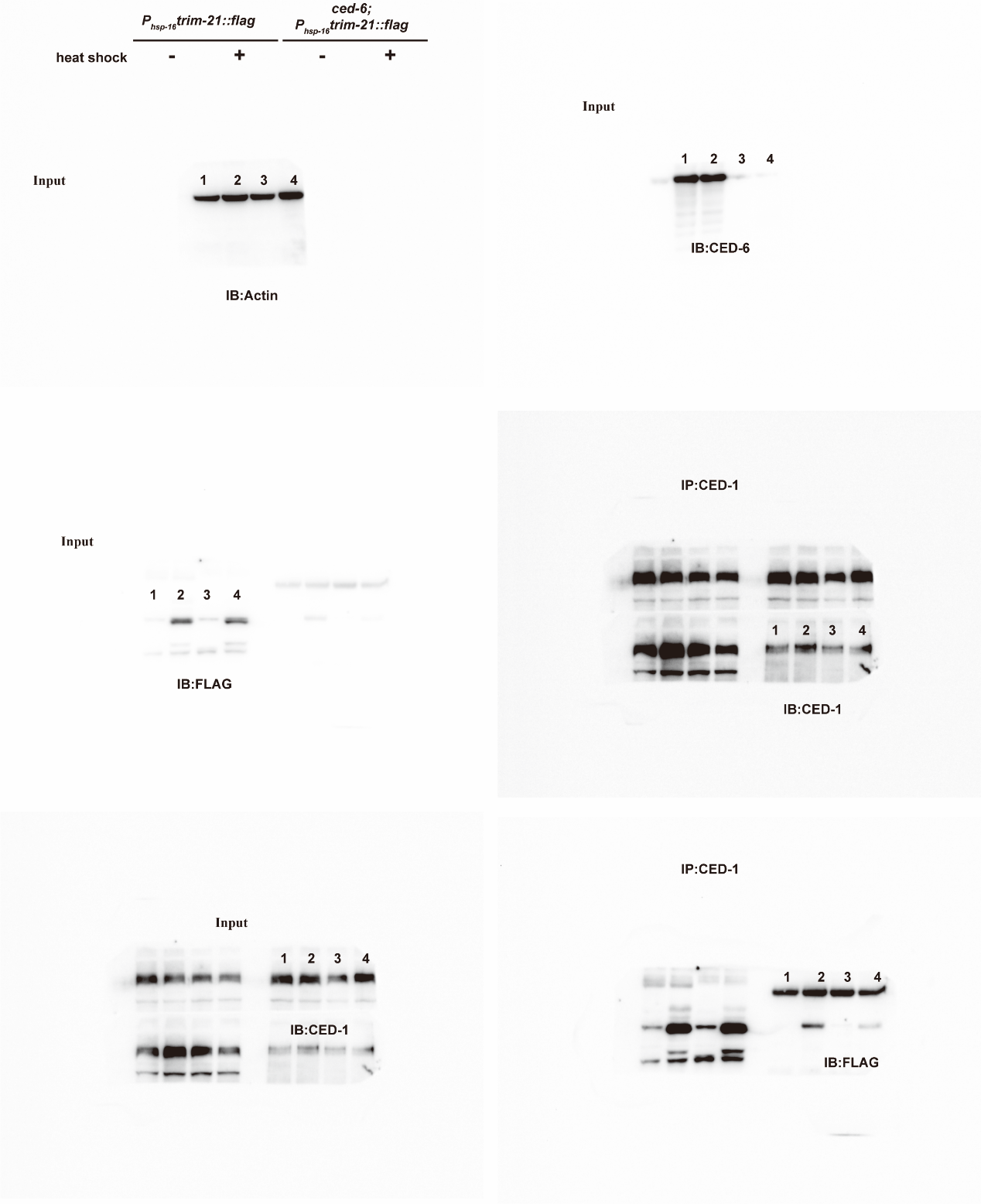


Figure 3L


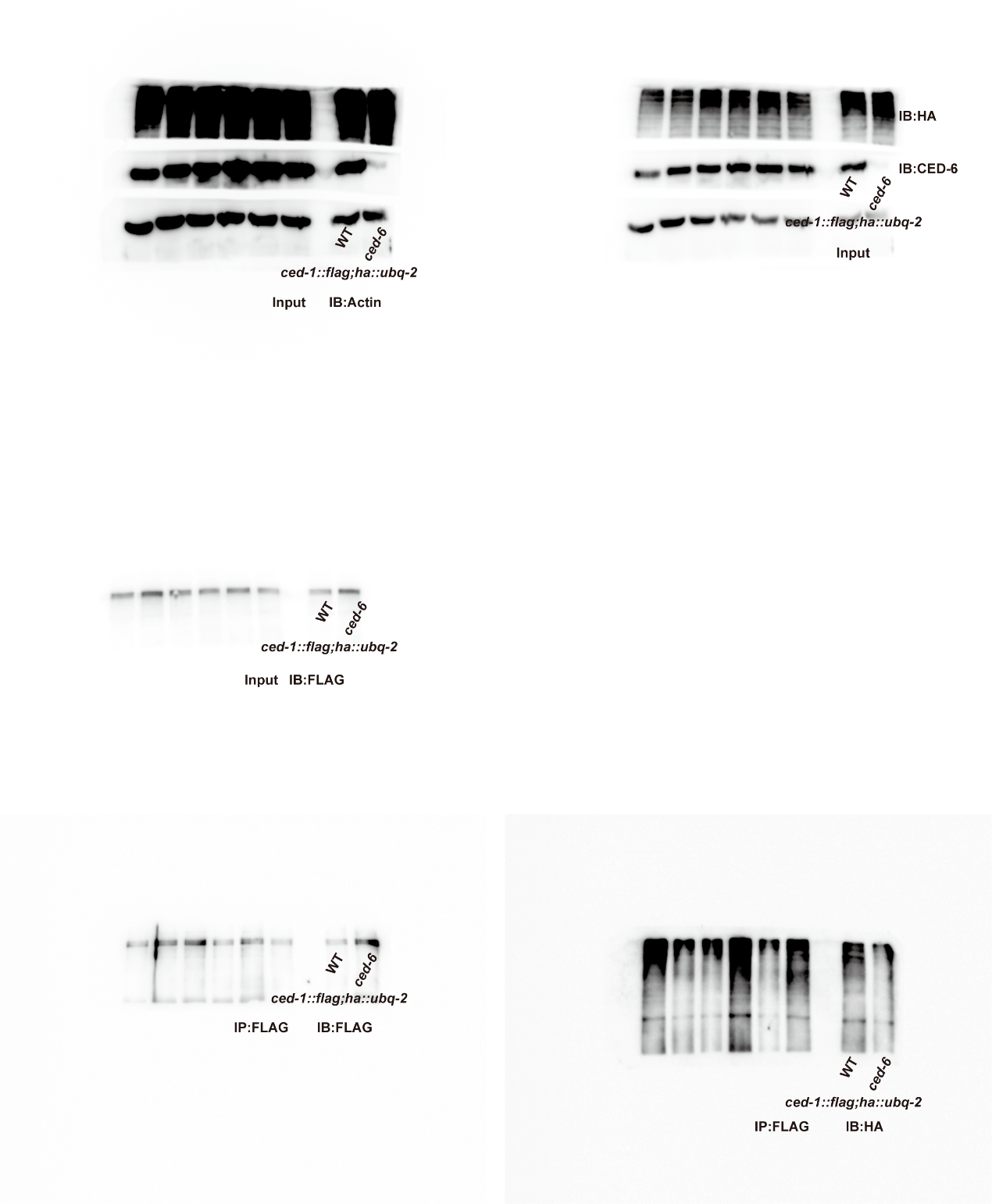

Supplement: Figure 3—source data 1. — Including uncropped Western blot images and raw statistics. [file elife-76436-fig3-data1.zip › Figure 3-Source Data 1/Figure 3 uncroppped blot with relevant bands.docx]

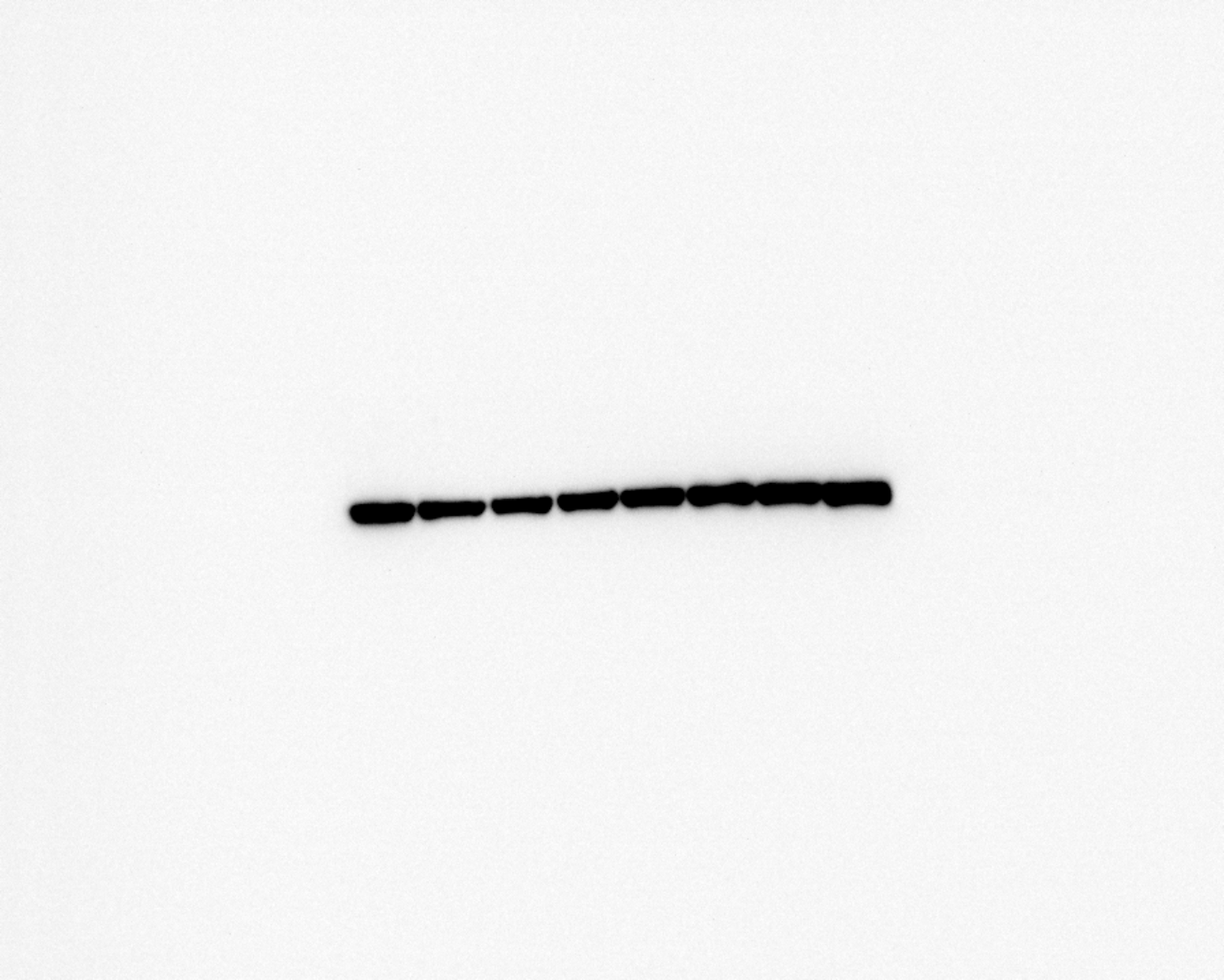

Supplement: Figure 3—source data 1. — Including uncropped Western blot images and raw statistics. [file elife-76436-fig3-data1.zip › Figure 3-Source Data 1/Figure 3A full raw unedited/IB-Actin-N2-ced-2-5.tif]

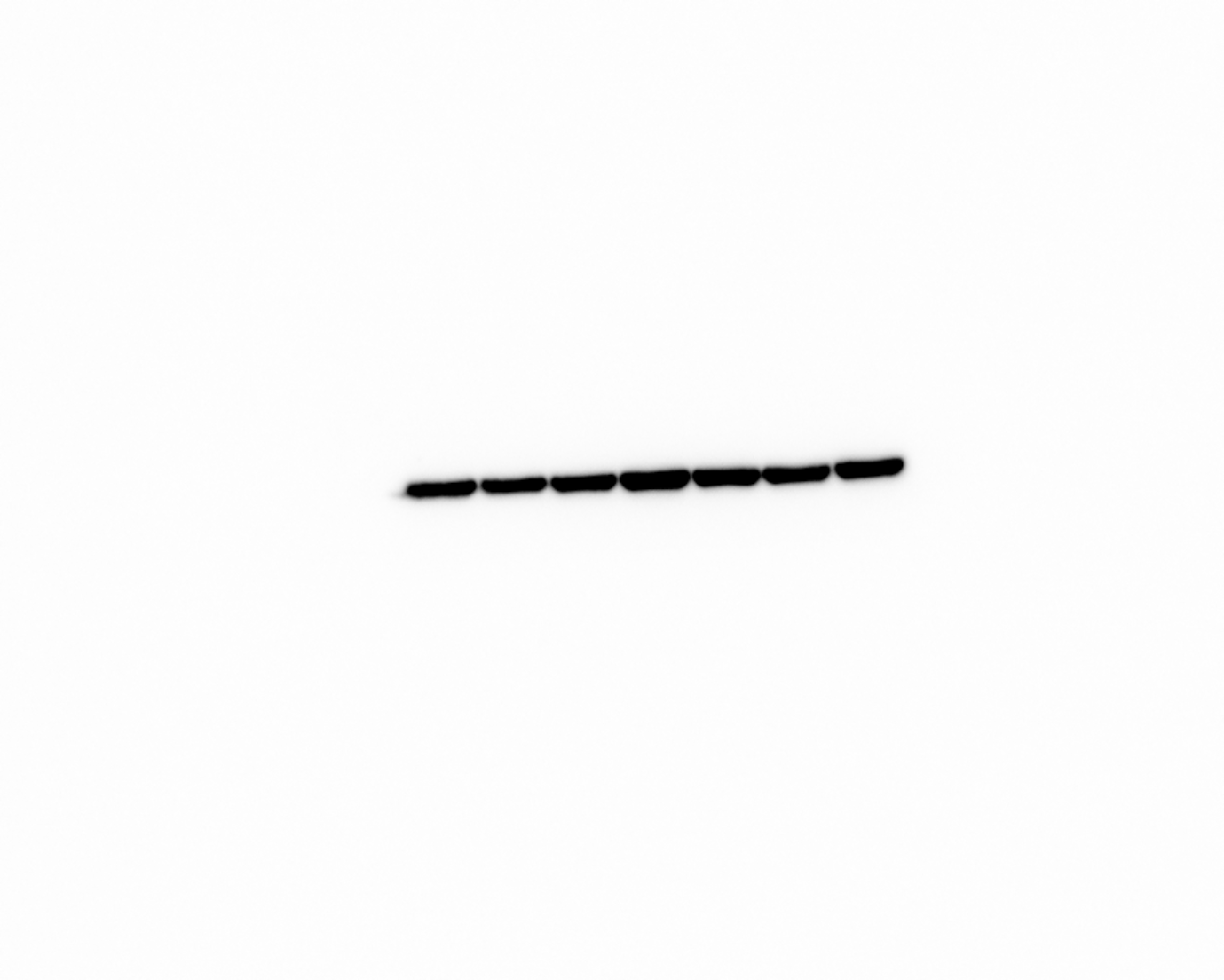

Supplement: Figure 3—source data 1. — Including uncropped Western blot images and raw statistics. [file elife-76436-fig3-data1.zip › Figure 3-Source Data 1/Figure 3A full raw unedited/IB-Actin-N2-ced-4-3-8-6.tif]

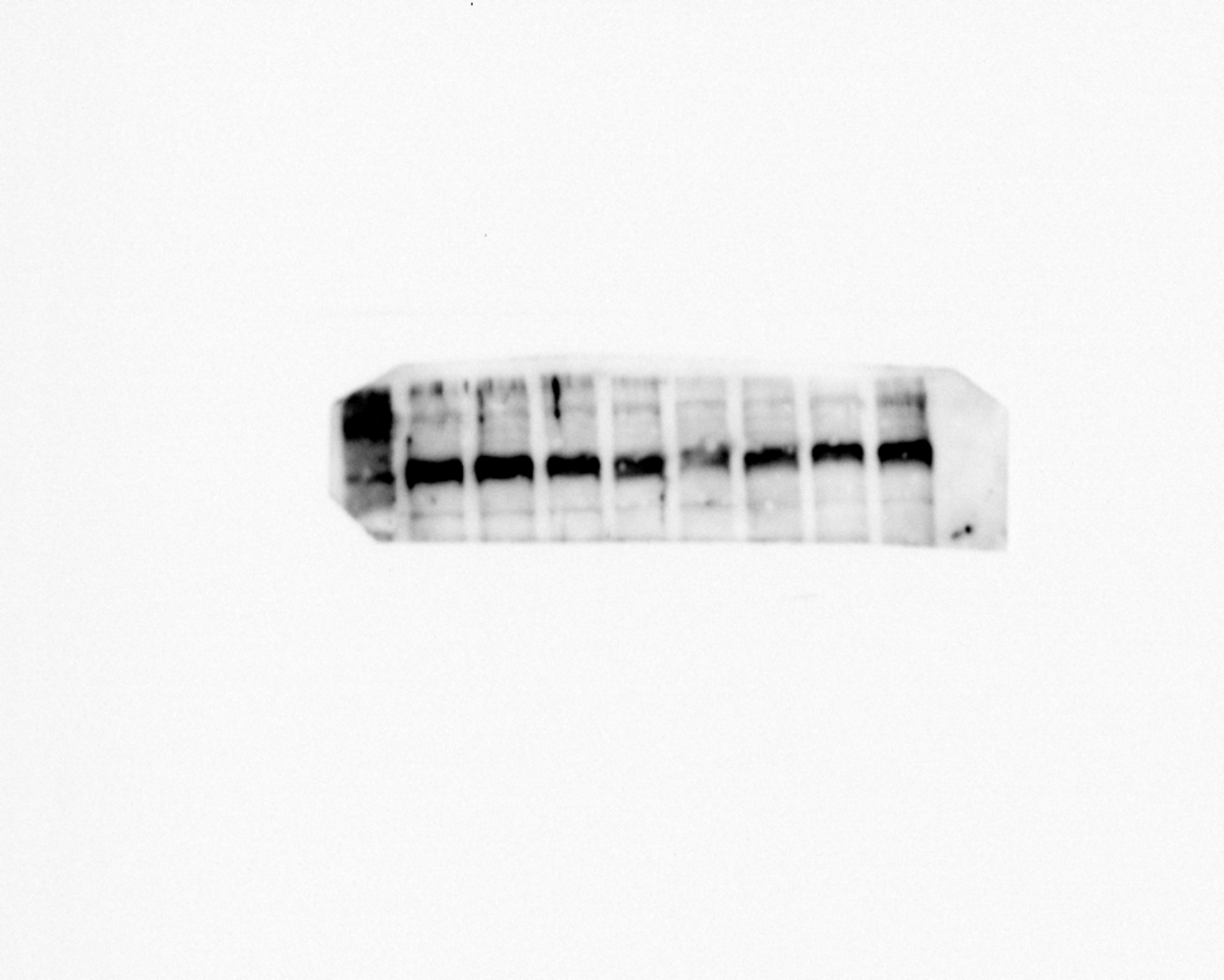

Supplement: Figure 3—source data 1. — Including uncropped Western blot images and raw statistics. [file elife-76436-fig3-data1.zip › Figure 3-Source Data 1/Figure 3A full raw unedited/IB-CED-1-N2-ced-2-5.tif]

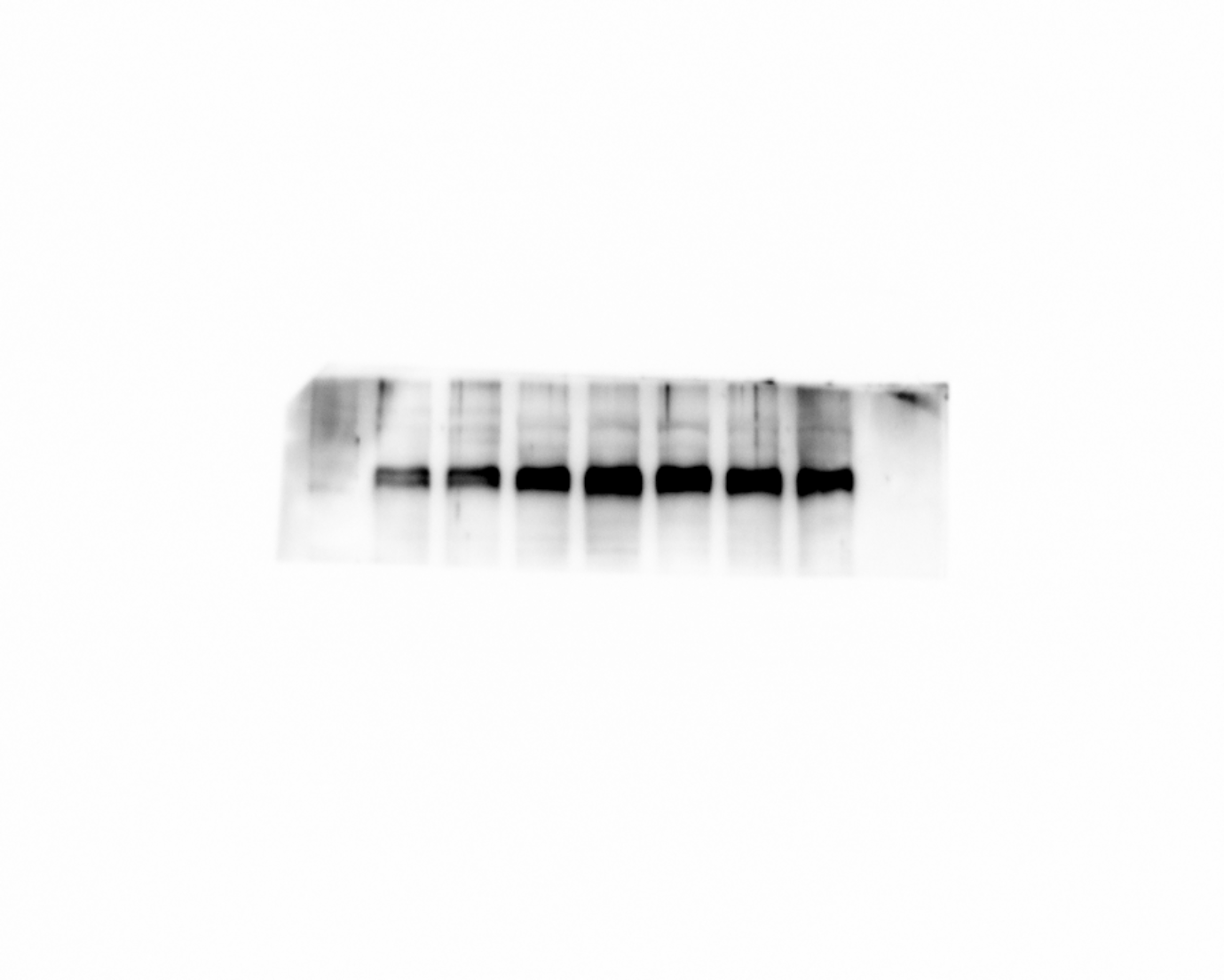

Supplement: Figure 3—source data 1. — Including uncropped Western blot images and raw statistics. [file elife-76436-fig3-data1.zip › Figure 3-Source Data 1/Figure 3A full raw unedited/IB-CED-1-N2-ced-4-3-8-6.tif]

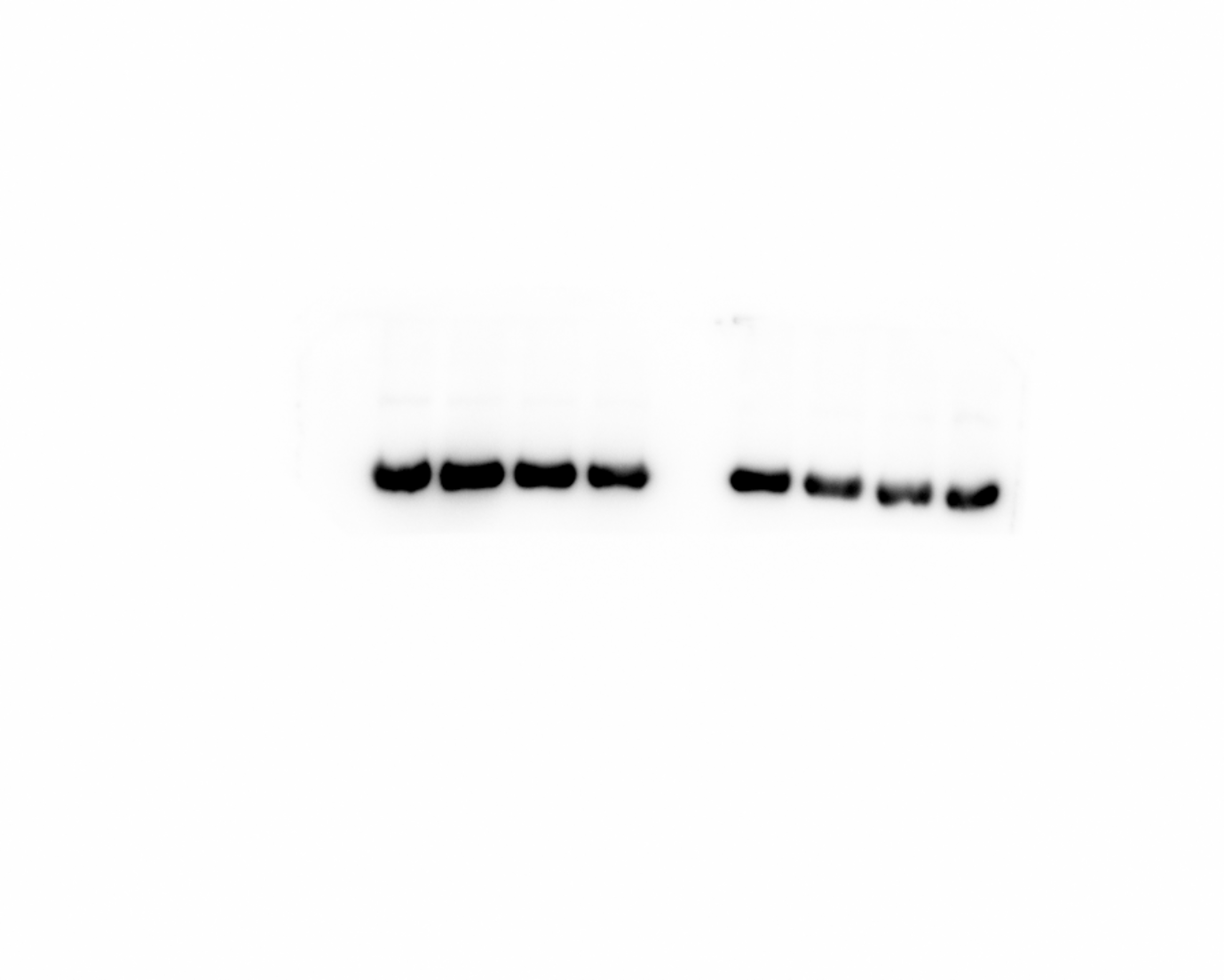

Supplement: Figure 3—source data 1. — Including uncropped Western blot images and raw statistics. [file elife-76436-fig3-data1.zip › Figure 3-Source Data 1/Figure 3B full raw unedited/IB-Actin.tif]

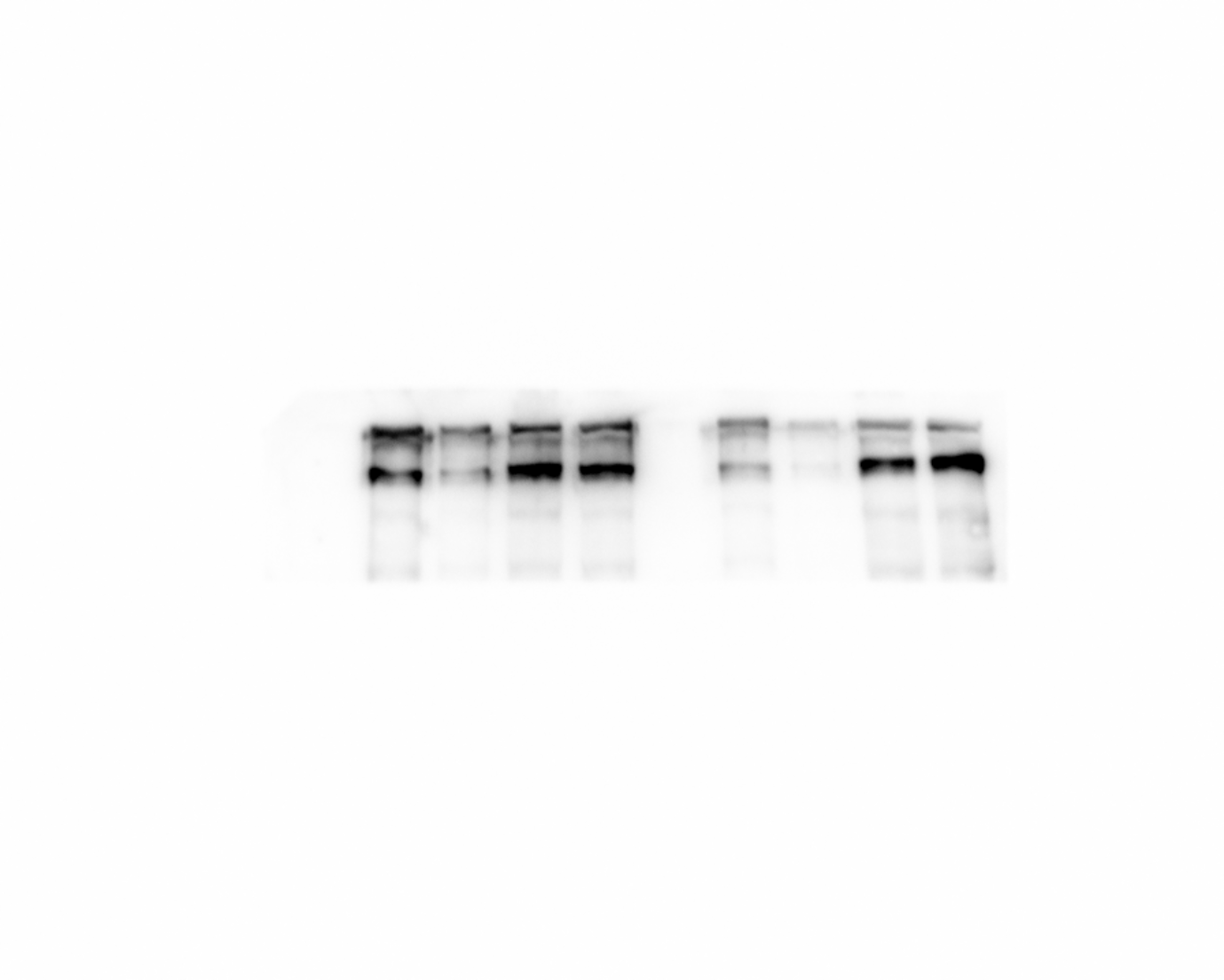

Supplement: Figure 3—source data 1. — Including uncropped Western blot images and raw statistics. [file elife-76436-fig3-data1.zip › Figure 3-Source Data 1/Figure 3B full raw unedited/IB-CED-1.tif]

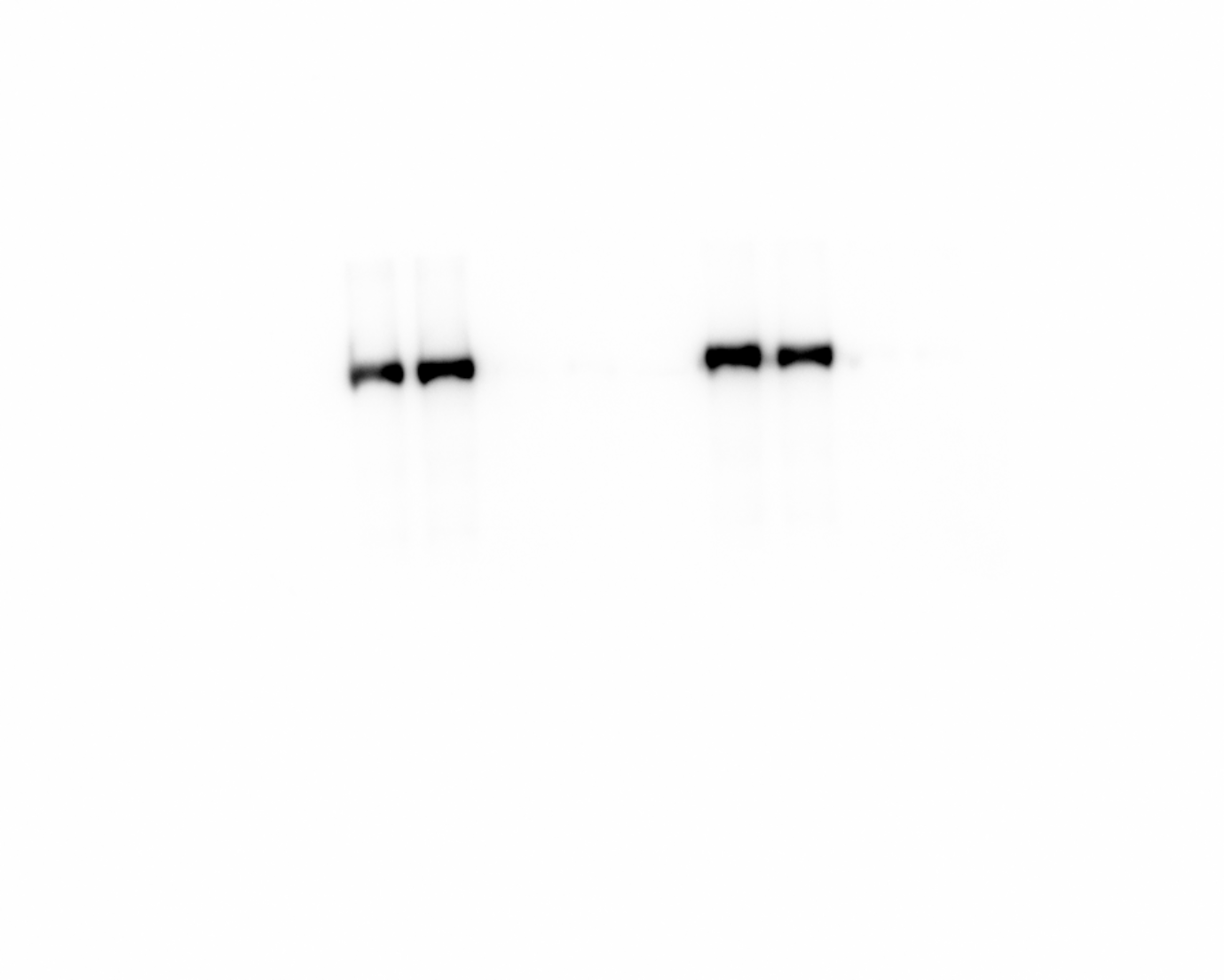

Supplement: Figure 3—source data 1. — Including uncropped Western blot images and raw statistics. [file elife-76436-fig3-data1.zip › Figure 3-Source Data 1/Figure 3B full raw unedited/IB-CED-6.tif]

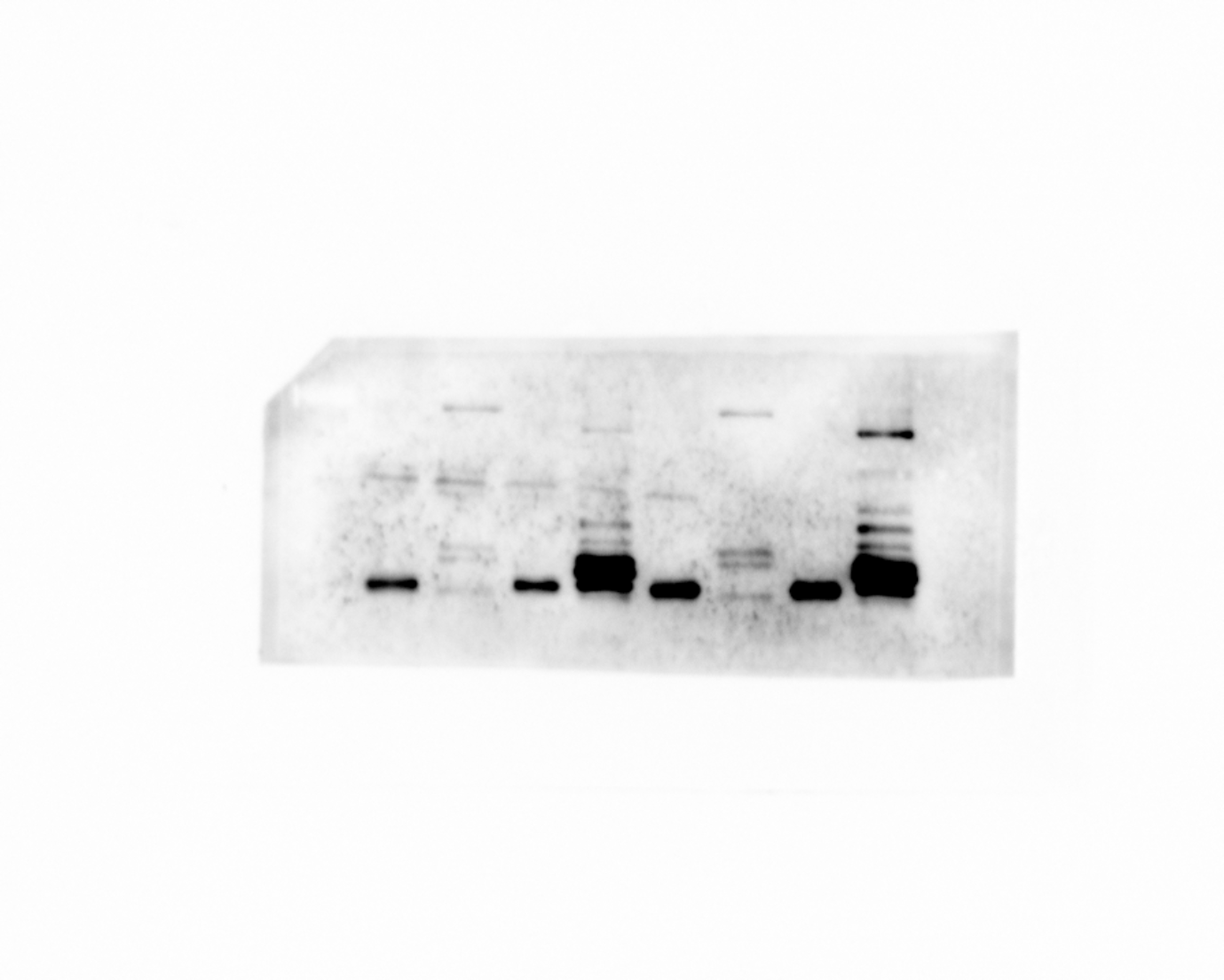

Supplement: Figure 3—source data 1. — Including uncropped Western blot images and raw statistics. [file elife-76436-fig3-data1.zip › Figure 3-Source Data 1/Figure 3C full raw unedited/IB-GST.tif]

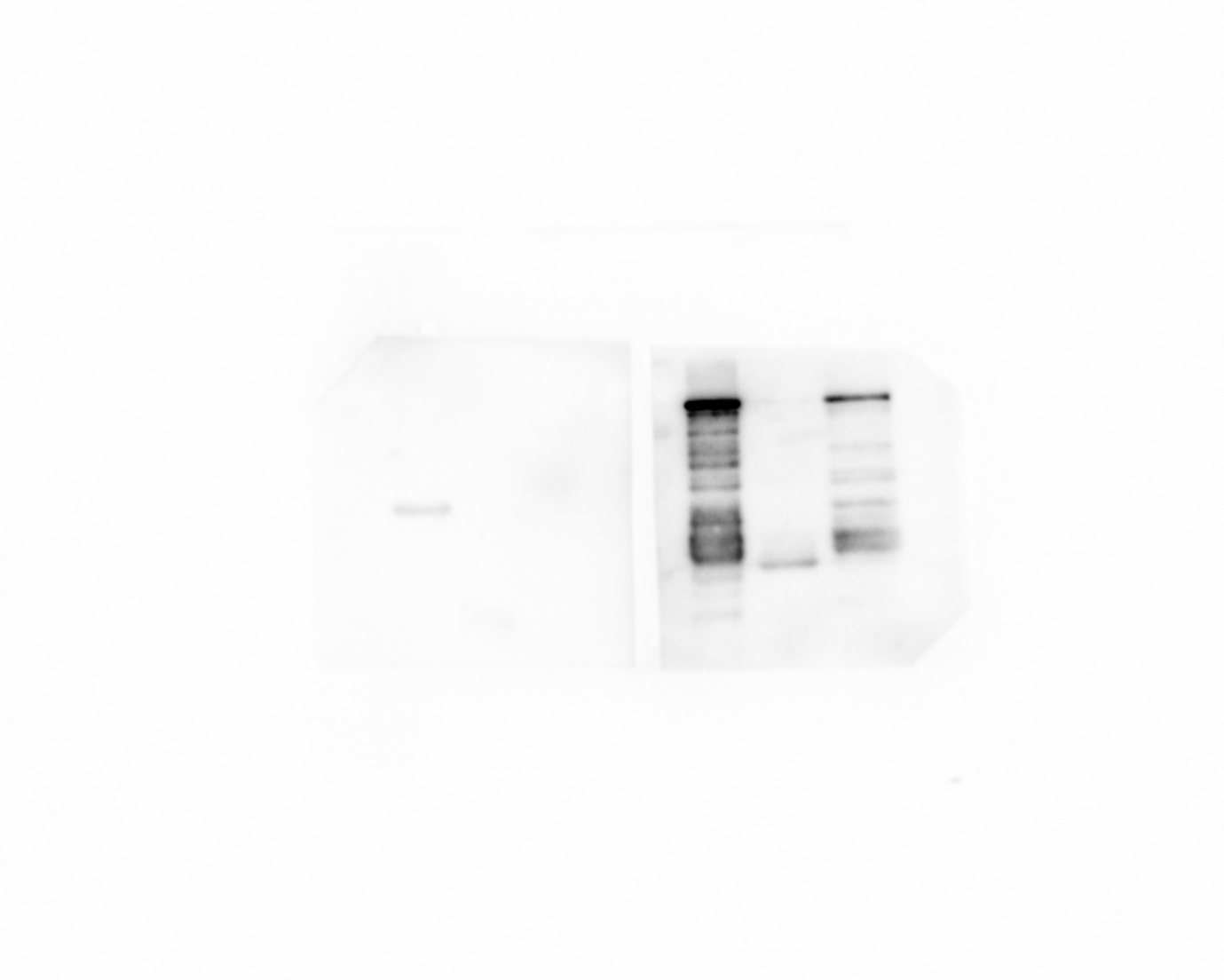

Supplement: Figure 3—source data 1. — Including uncropped Western blot images and raw statistics. [file elife-76436-fig3-data1.zip › Figure 3-Source Data 1/Figure 3C full raw unedited/IB-HA.tif]

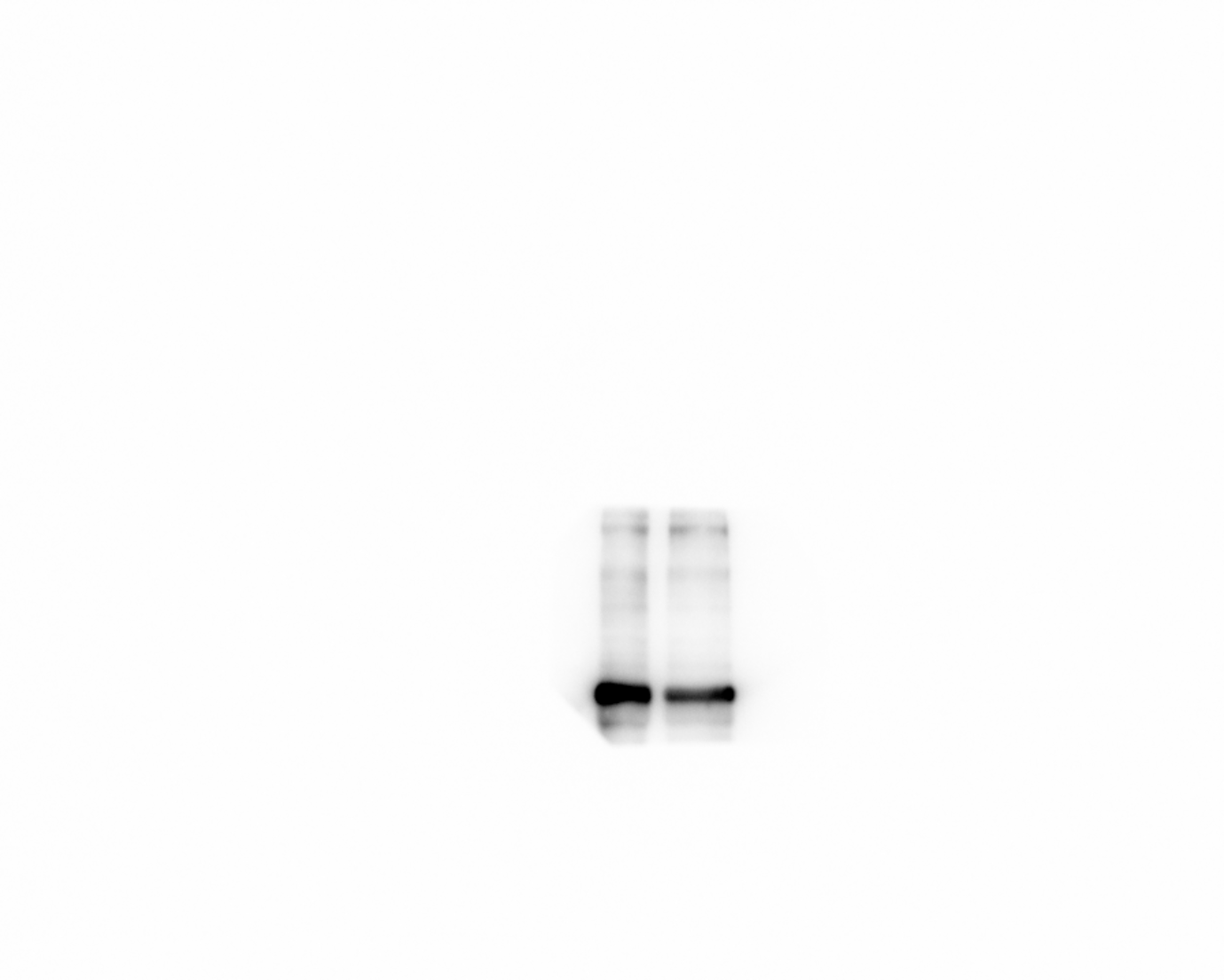

Supplement: Figure 3—source data 1. — Including uncropped Western blot images and raw statistics. [file elife-76436-fig3-data1.zip › Figure 3-Source Data 1/Figure 3D full raw unedited/IP-IB-CED-6.tif]

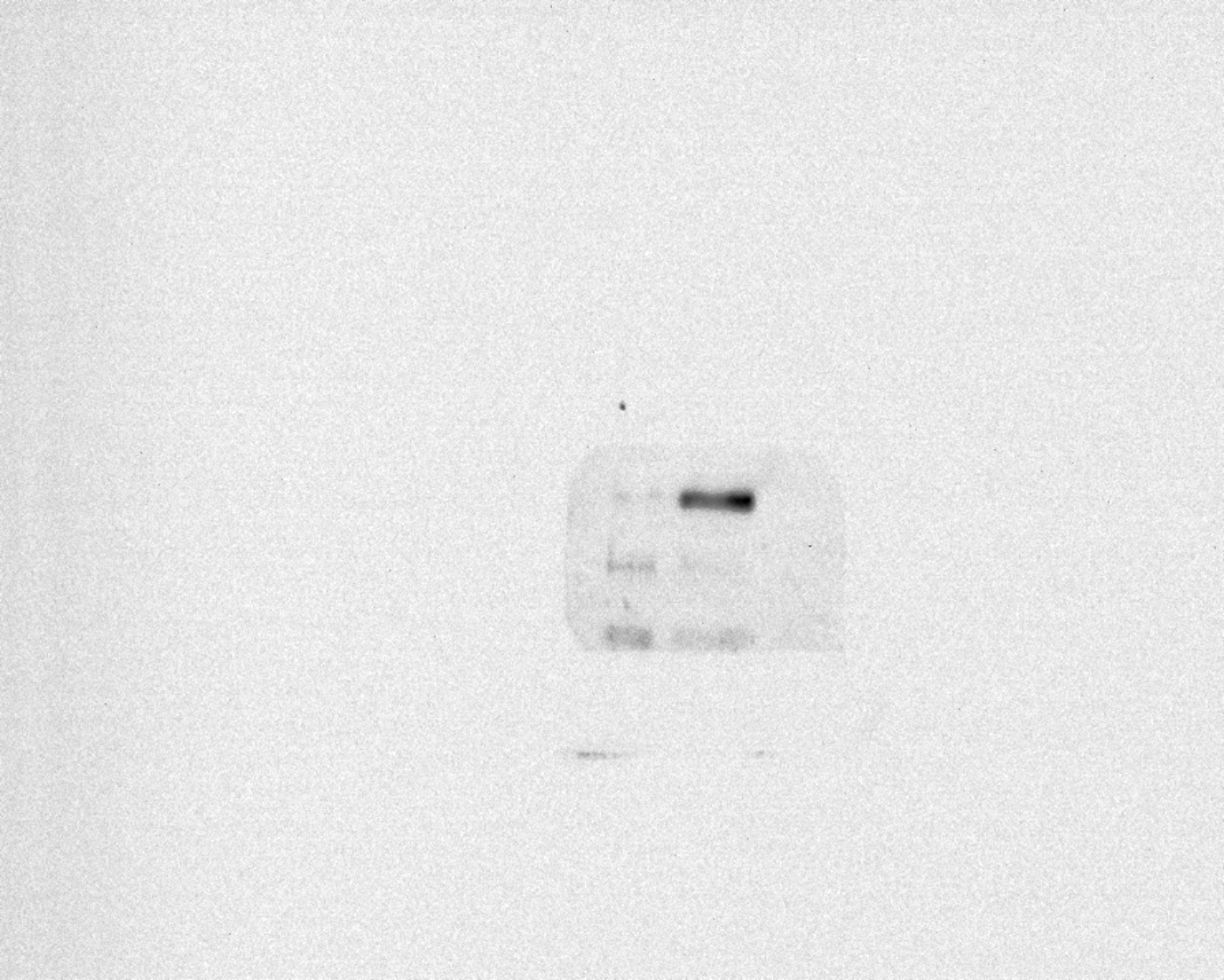

Supplement: Figure 3—source data 1. — Including uncropped Western blot images and raw statistics. [file elife-76436-fig3-data1.zip › Figure 3-Source Data 1/Figure 3D full raw unedited/IP-IB-FLAG.tif]

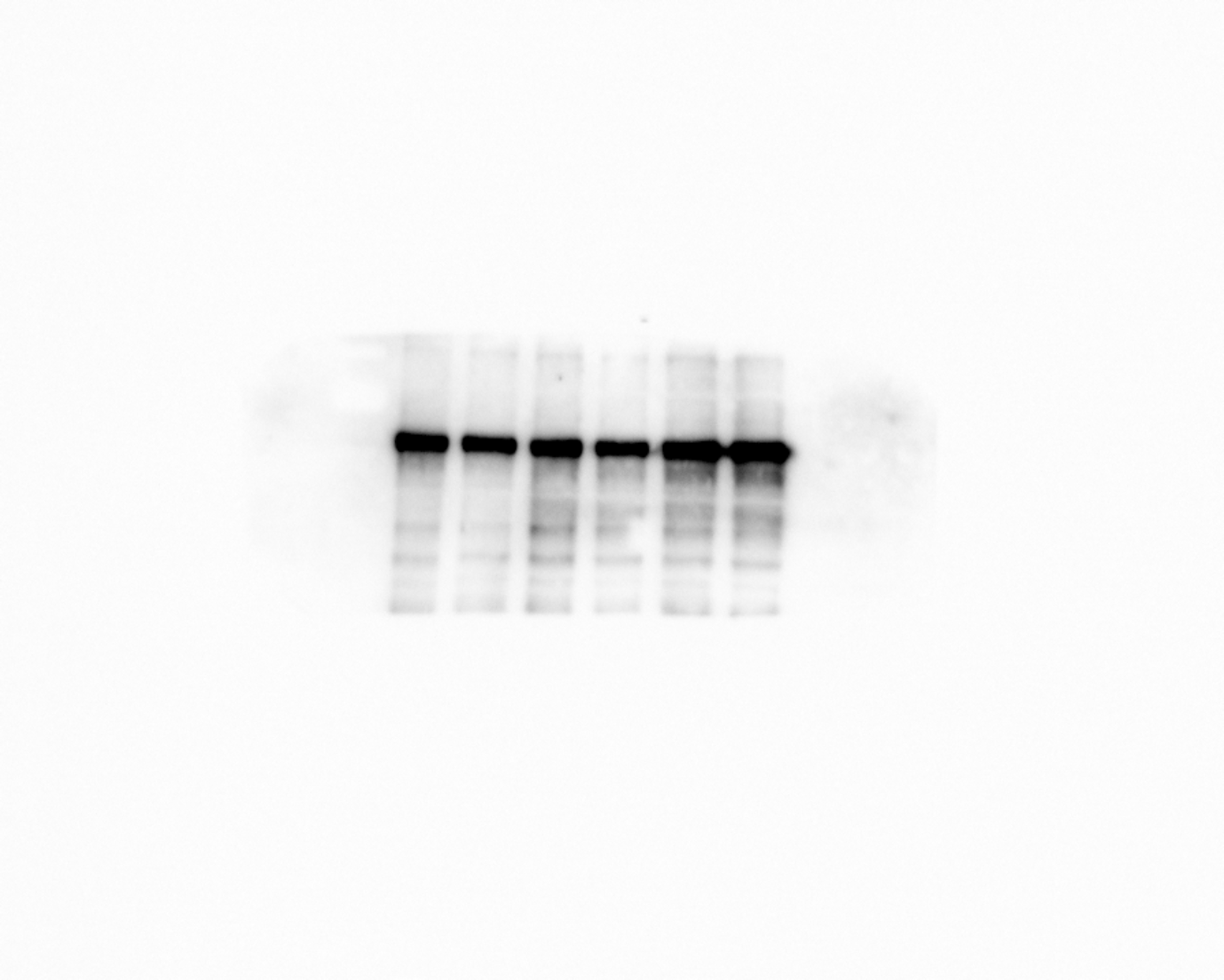

Supplement: Figure 3—source data 1. — Including uncropped Western blot images and raw statistics. [file elife-76436-fig3-data1.zip › Figure 3-Source Data 1/Figure 3E full raw unedited/IB-Actin.tif]

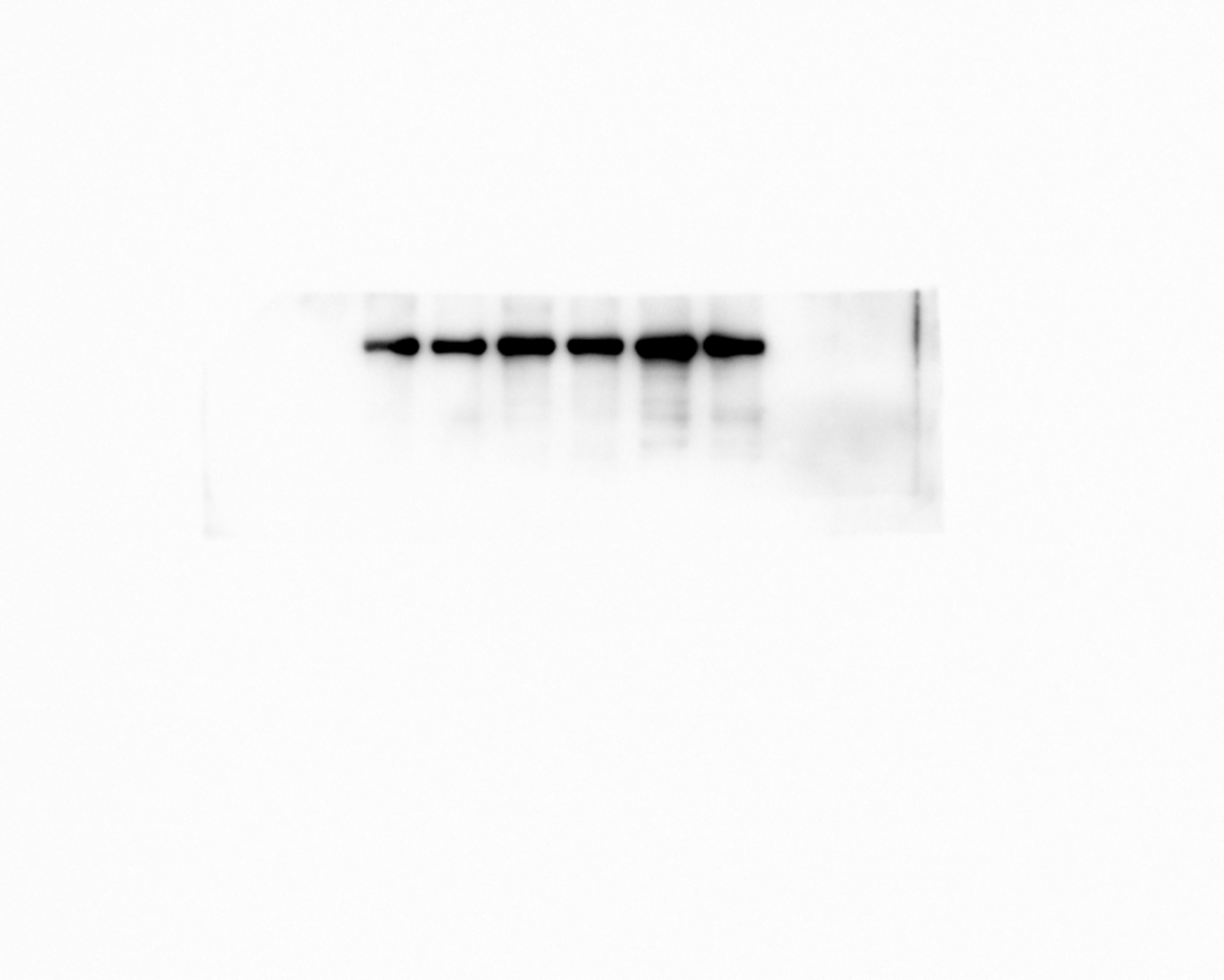

Supplement: Figure 3—source data 1. — Including uncropped Western blot images and raw statistics. [file elife-76436-fig3-data1.zip › Figure 3-Source Data 1/Figure 3E full raw unedited/IB-CED-6.tif]

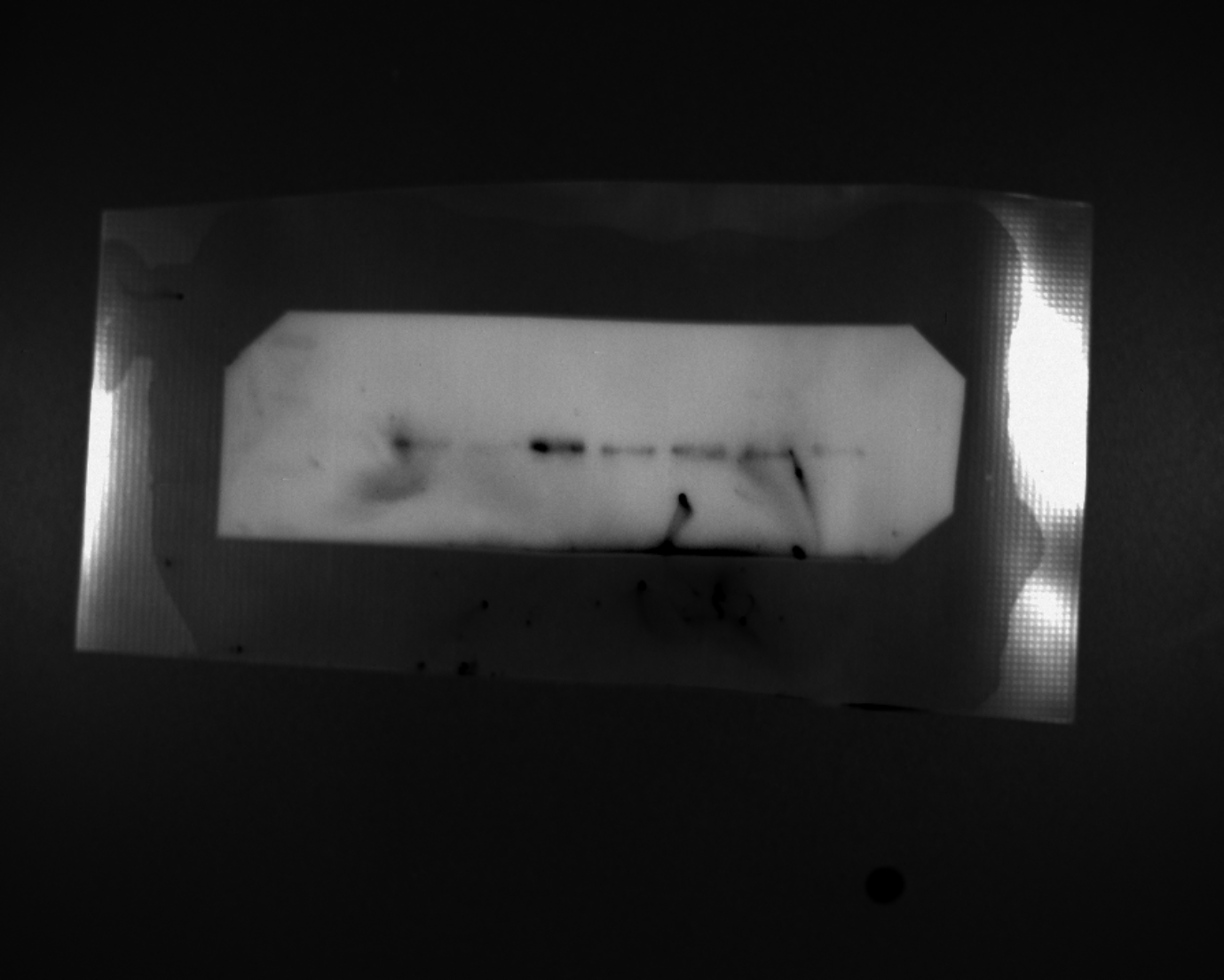

Supplement: Figure 3—source data 1. — Including uncropped Western blot images and raw statistics. [file elife-76436-fig3-data1.zip › Figure 3-Source Data 1/Figure 3H full raw unedited/GST-Pull down-IB-FLAG.tif]

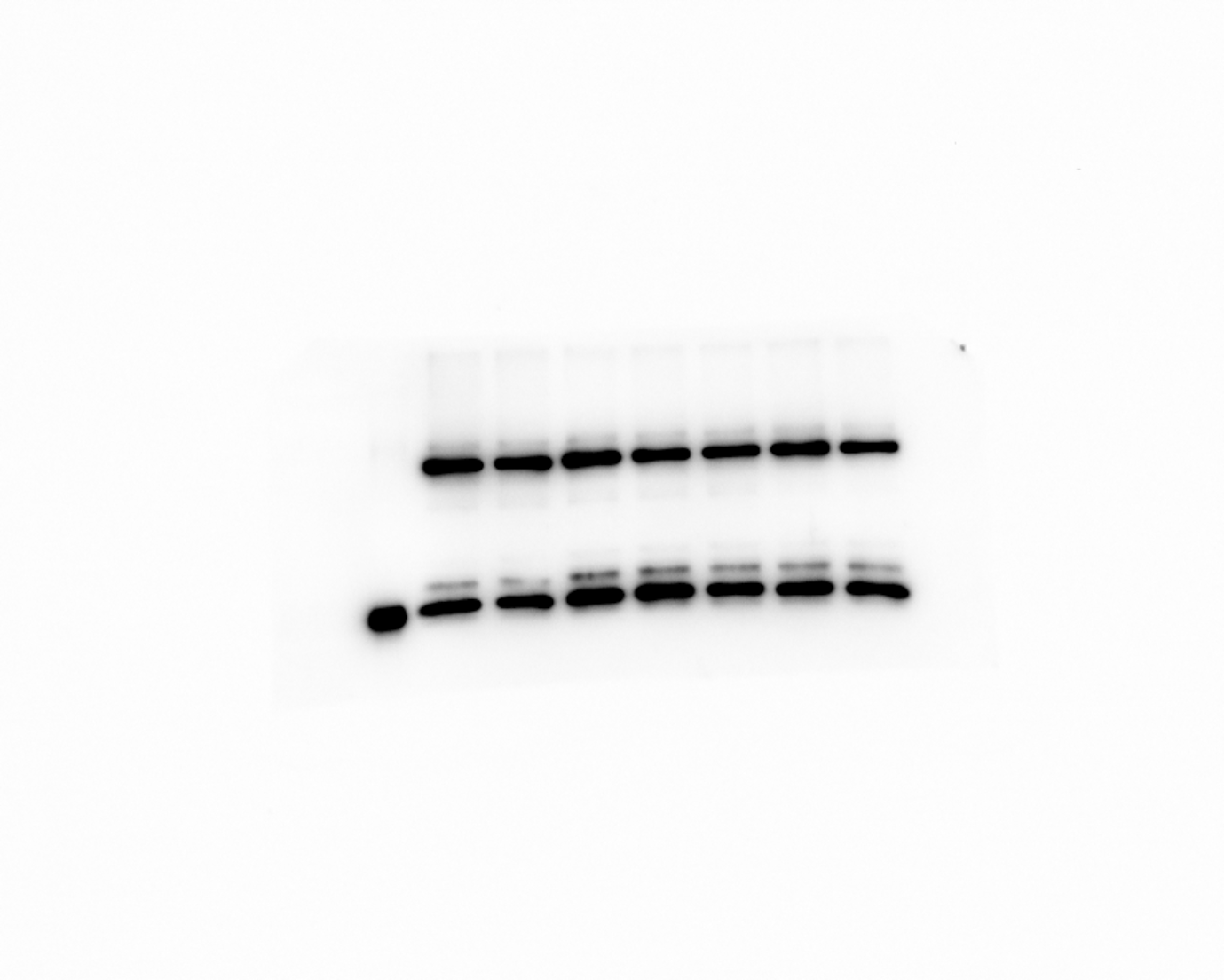

Supplement: Figure 3—source data 1. — Including uncropped Western blot images and raw statistics. [file elife-76436-fig3-data1.zip › Figure 3-Source Data 1/Figure 3H full raw unedited/GST-Pull down-IB-GST.tif]

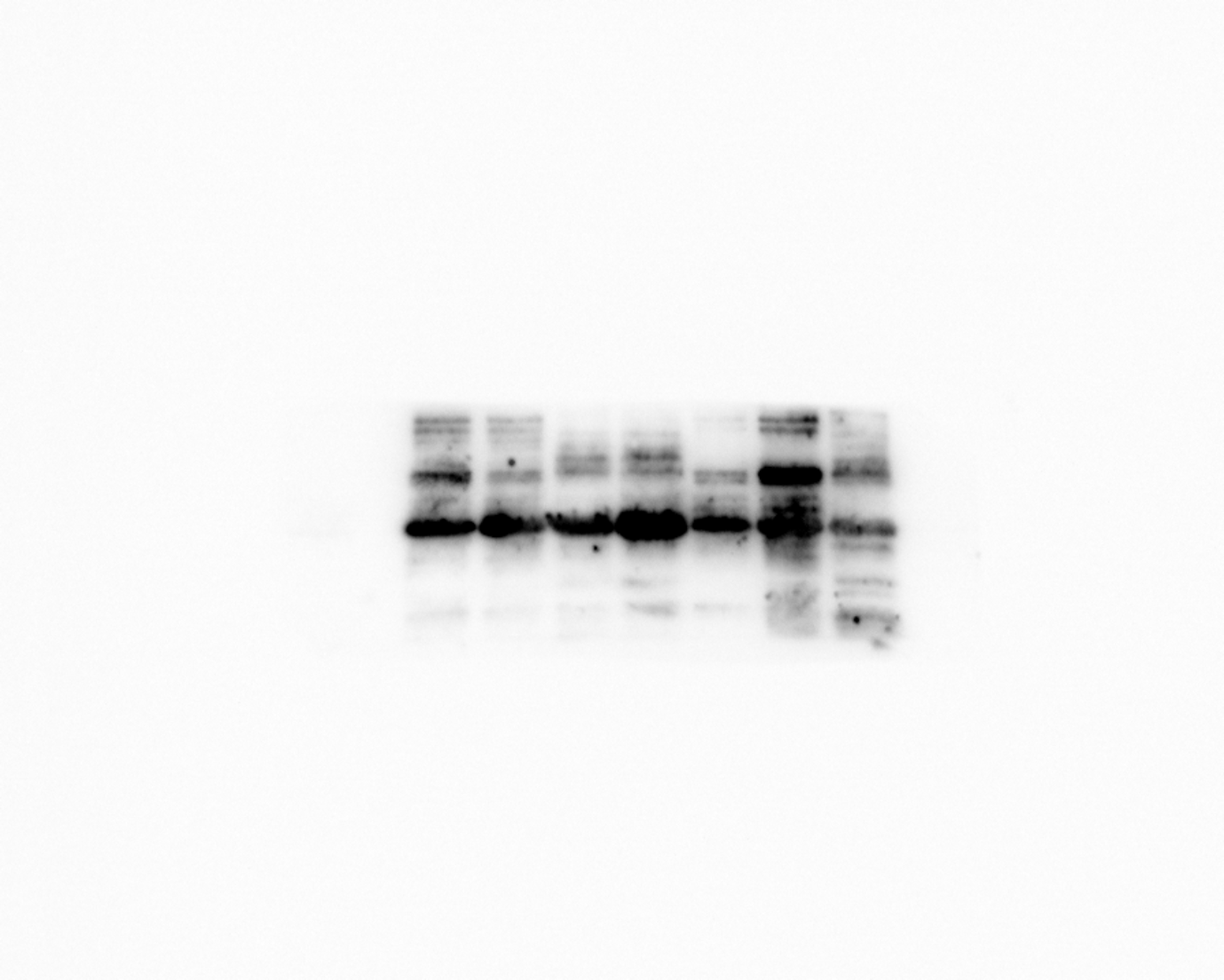

Supplement: Figure 3—source data 1. — Including uncropped Western blot images and raw statistics. [file elife-76436-fig3-data1.zip › Figure 3-Source Data 1/Figure 3H full raw unedited/Input-IB-FLAG.tif]

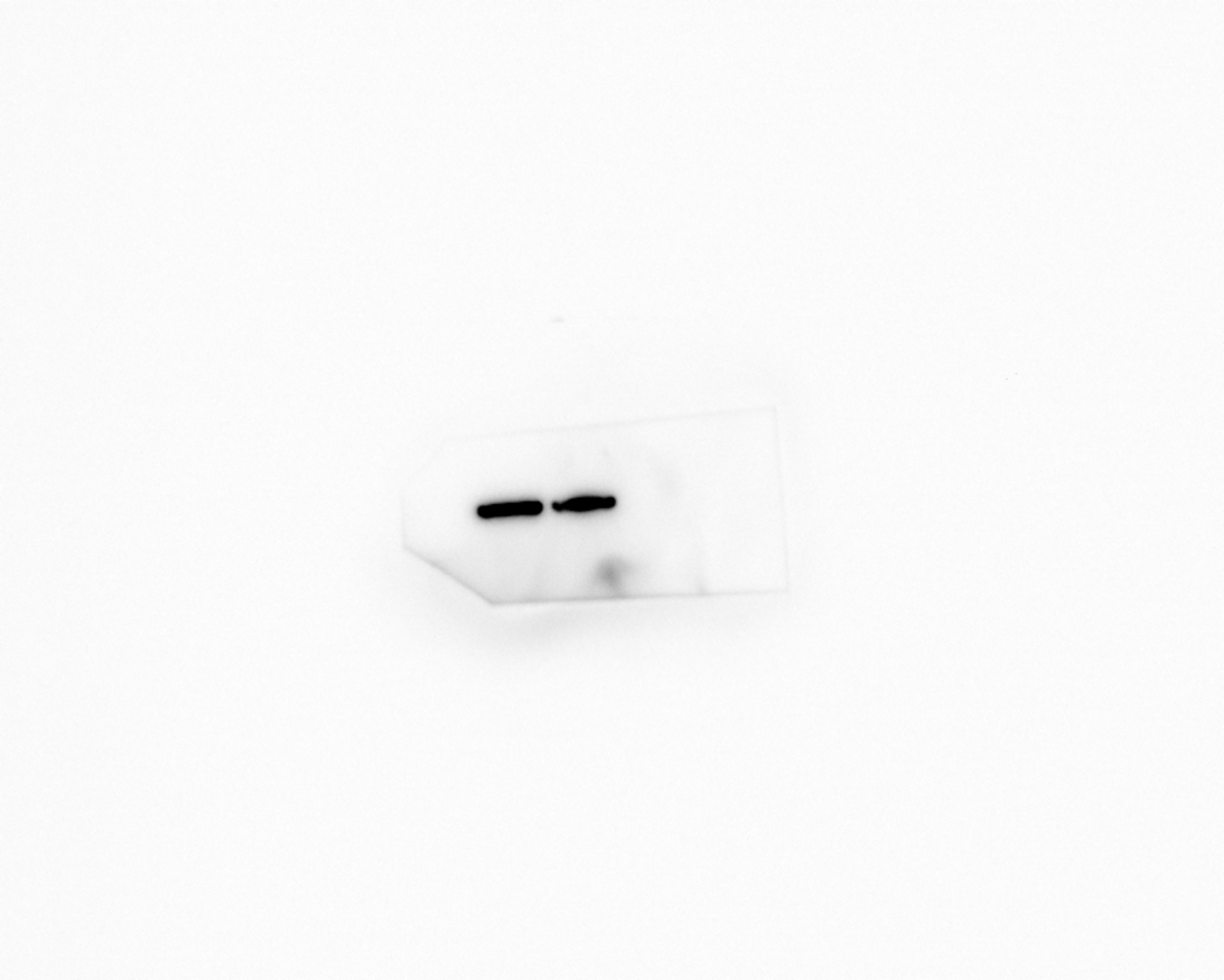

Supplement: Figure 3—source data 1. — Including uncropped Western blot images and raw statistics. [file elife-76436-fig3-data1.zip › Figure 3-Source Data 1/Figure 3I full raw unedited/IB-Actin.tif]

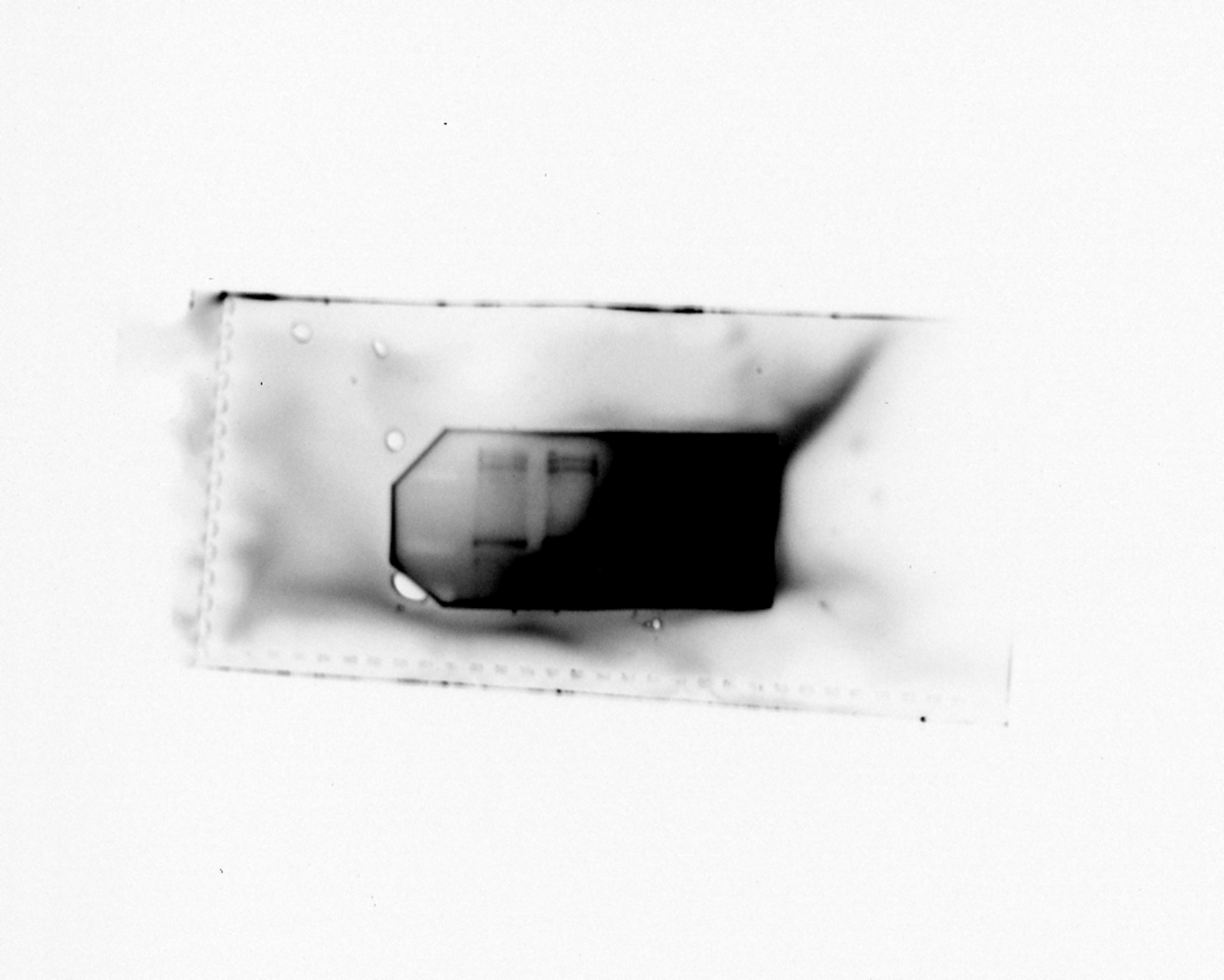

Supplement: Figure 3—source data 1. — Including uncropped Western blot images and raw statistics. [file elife-76436-fig3-data1.zip › Figure 3-Source Data 1/Figure 3I full raw unedited/IB-CED-1.tif]

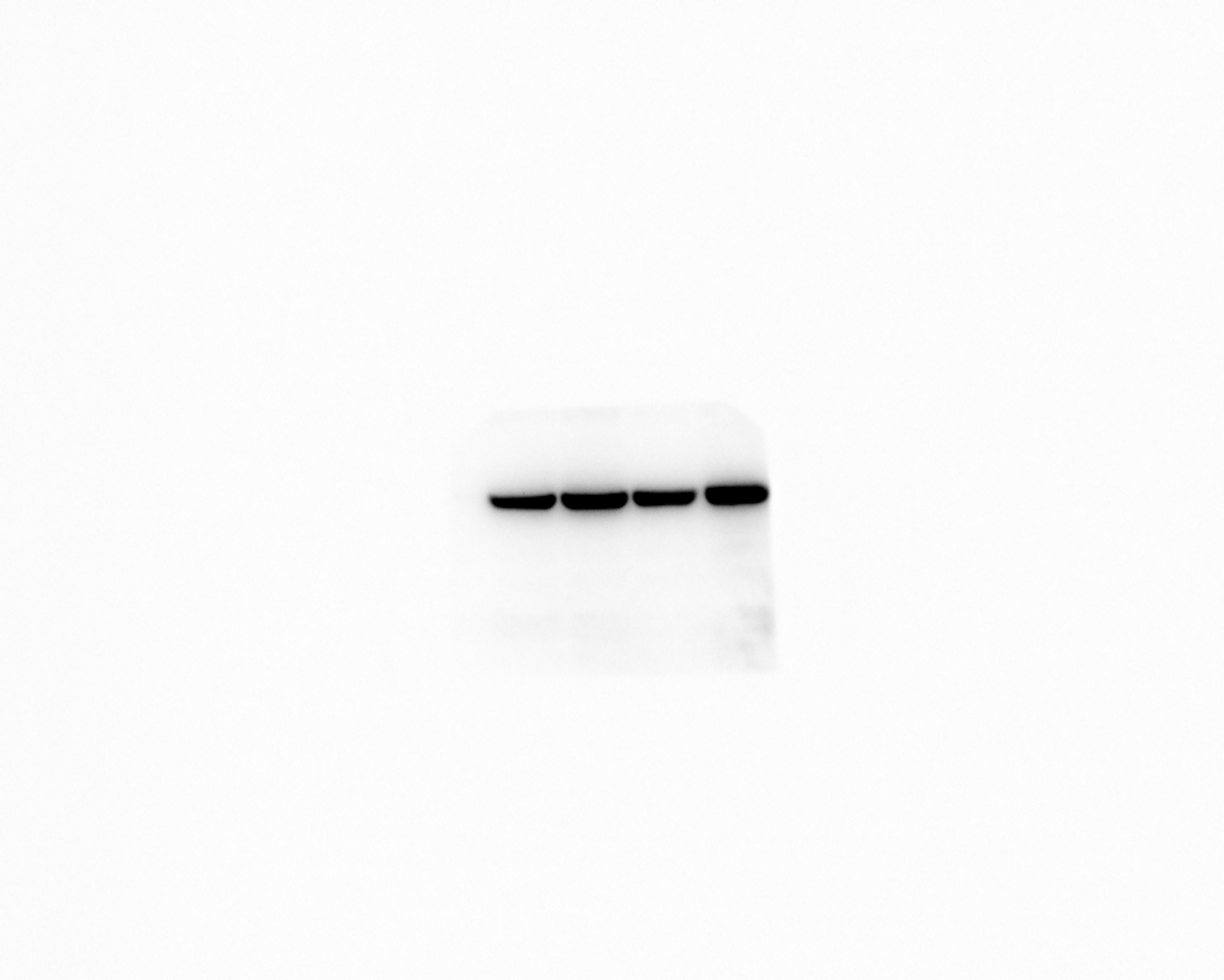

Supplement: Figure 3—source data 1. — Including uncropped Western blot images and raw statistics. [file elife-76436-fig3-data1.zip › Figure 3-Source Data 1/Figure 3J full raw unedited/Input-IB-Actin.tif]

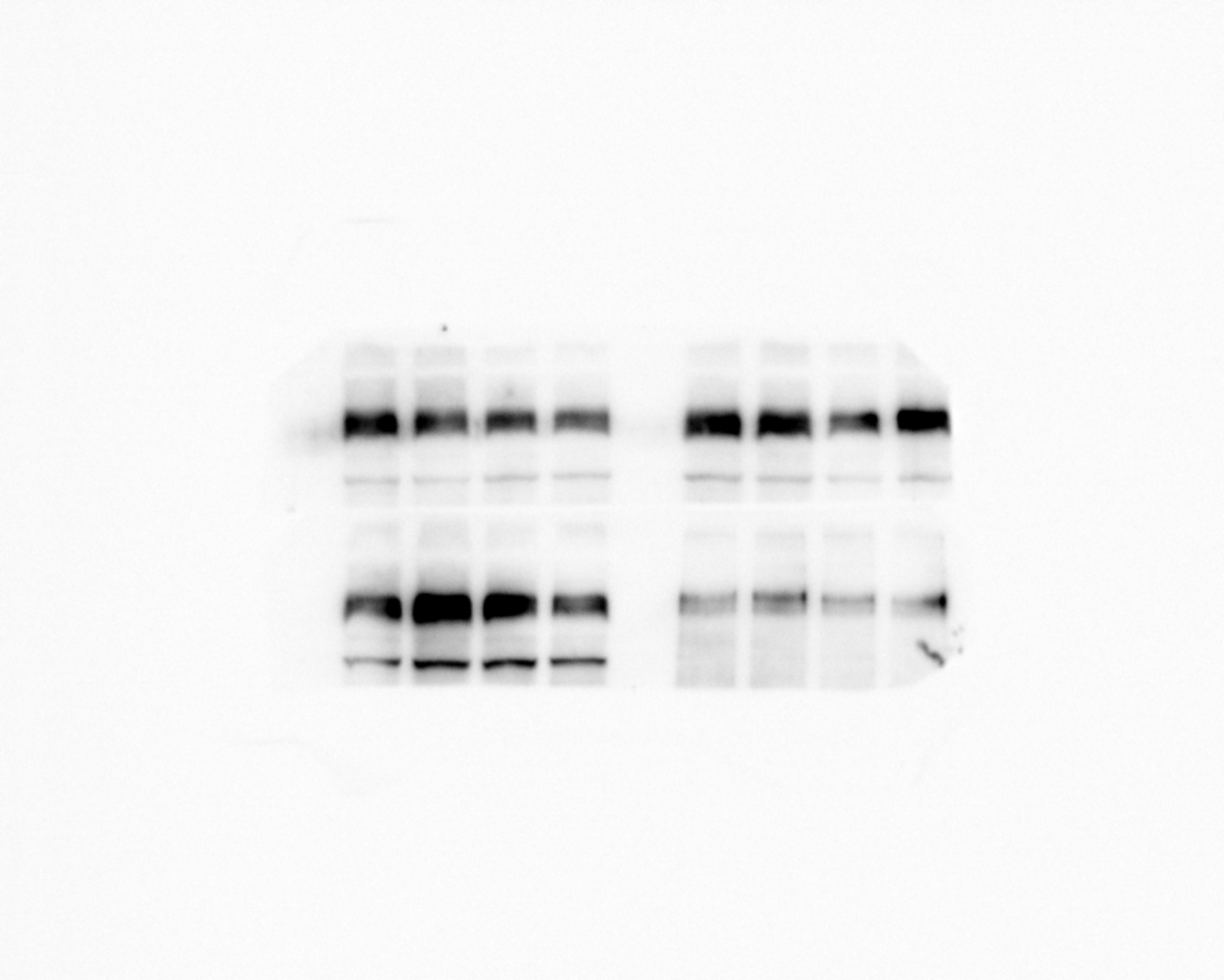

Supplement: Figure 3—source data 1. — Including uncropped Western blot images and raw statistics. [file elife-76436-fig3-data1.zip › Figure 3-Source Data 1/Figure 3J full raw unedited/Input-IB-CED-1.tif]

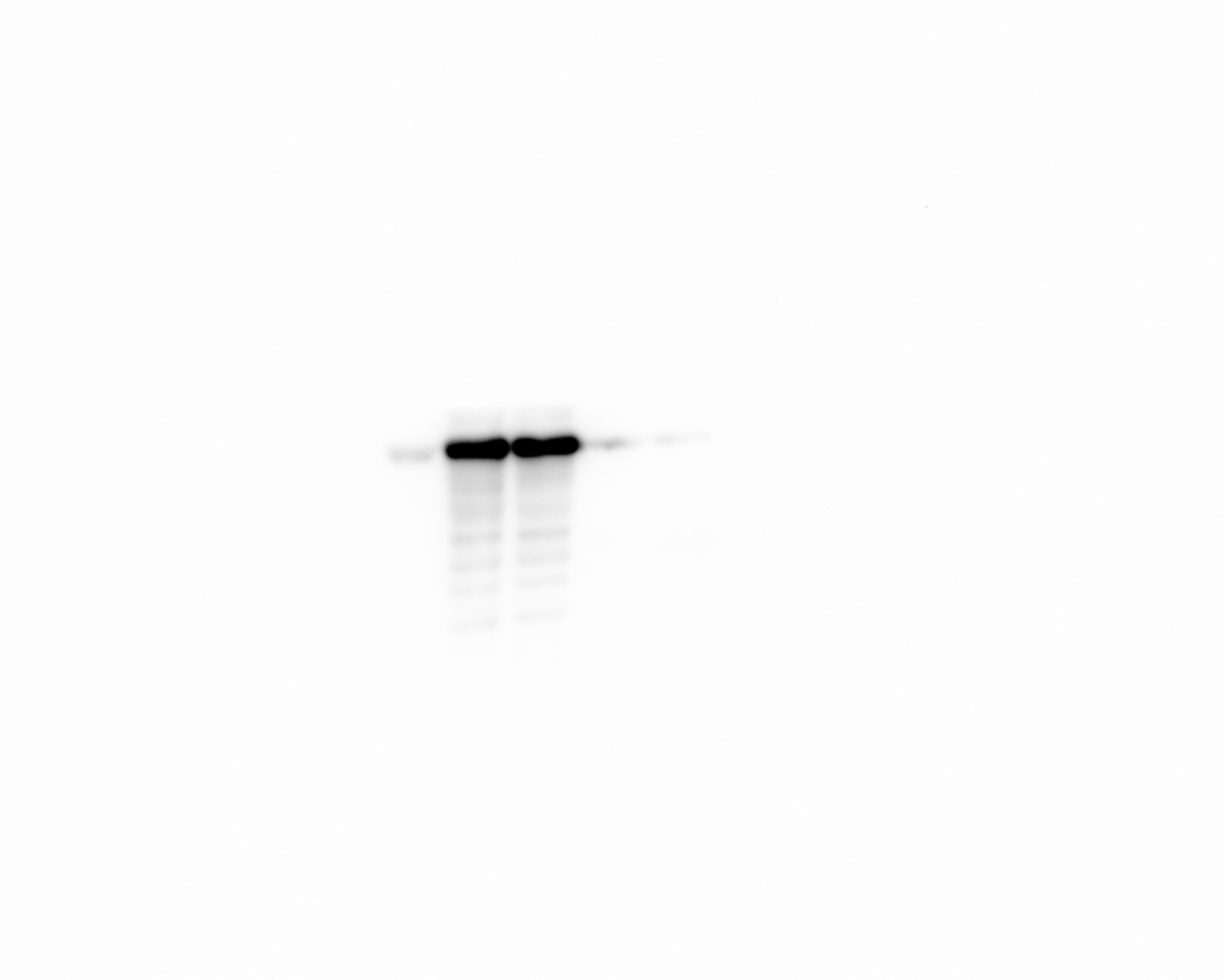

Supplement: Figure 3—source data 1. — Including uncropped Western blot images and raw statistics. [file elife-76436-fig3-data1.zip › Figure 3-Source Data 1/Figure 3J full raw unedited/Input-IB-CED-6.tif]

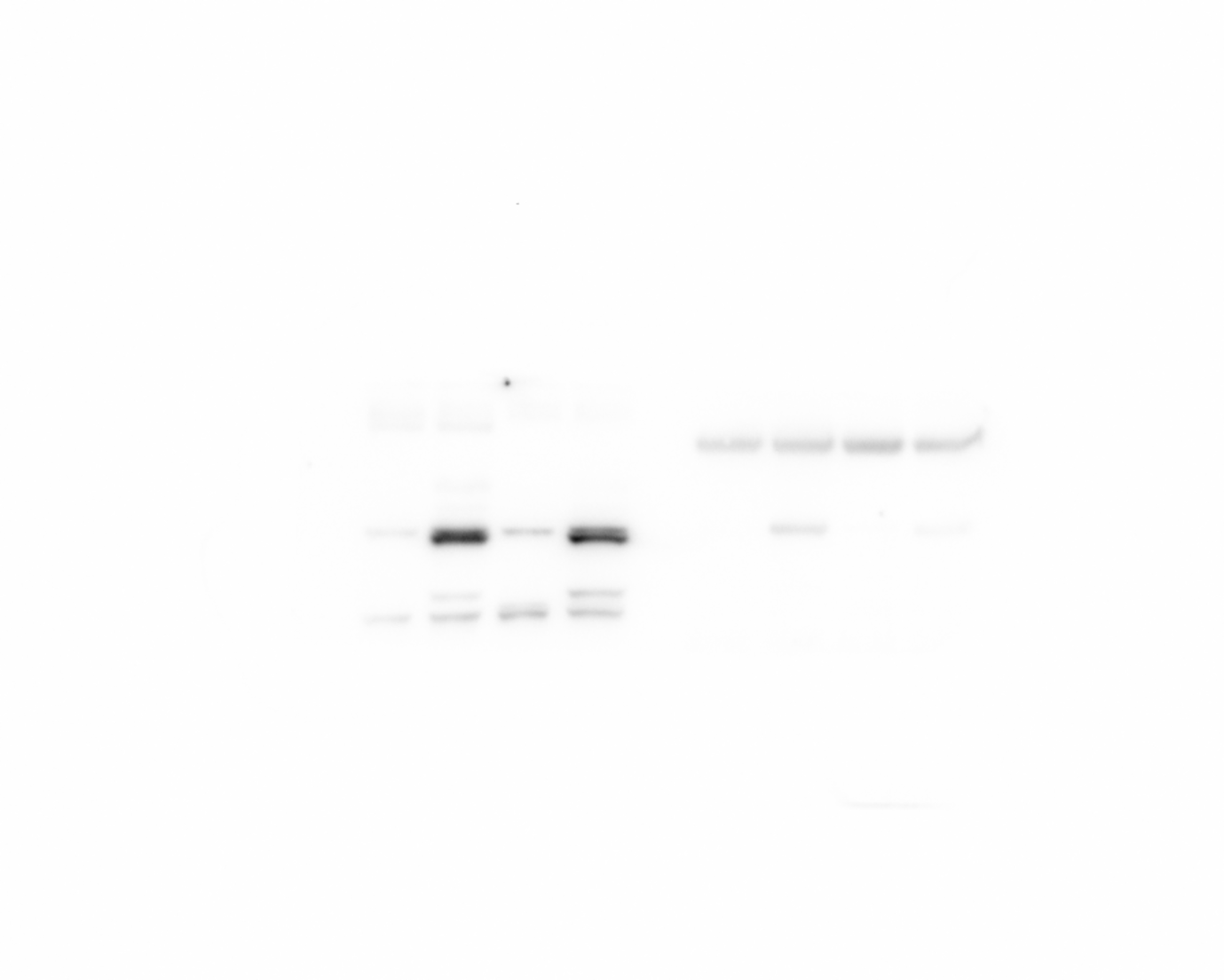

Supplement: Figure 3—source data 1. — Including uncropped Western blot images and raw statistics. [file elife-76436-fig3-data1.zip › Figure 3-Source Data 1/Figure 3J full raw unedited/Input-IB-FLAG.tif]

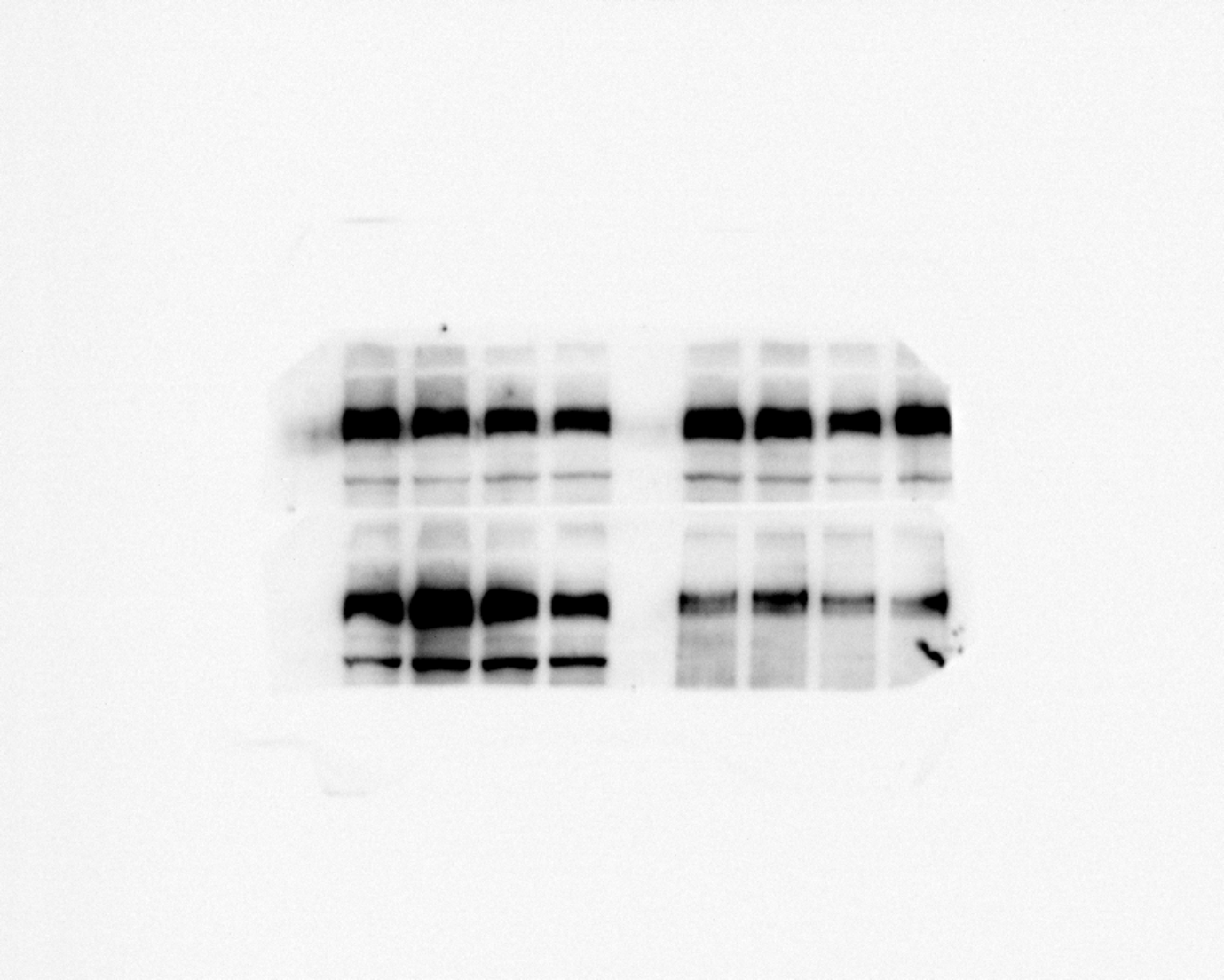

Supplement: Figure 3—source data 1. — Including uncropped Western blot images and raw statistics. [file elife-76436-fig3-data1.zip › Figure 3-Source Data 1/Figure 3J full raw unedited/IP-IB-CED-1.tif]

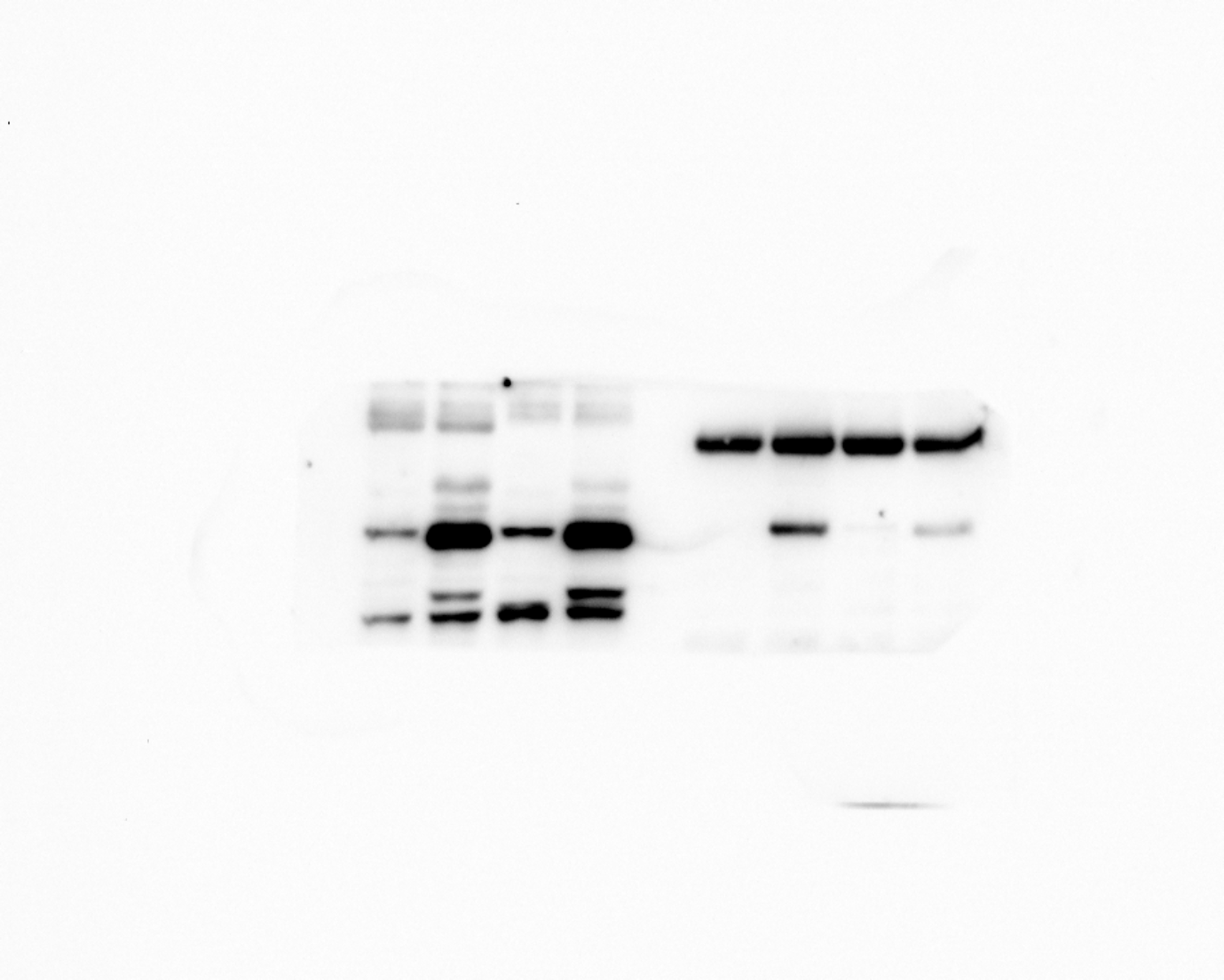

Supplement: Figure 3—source data 1. — Including uncropped Western blot images and raw statistics. [file elife-76436-fig3-data1.zip › Figure 3-Source Data 1/Figure 3J full raw unedited/IP-IB-FLAG.tif]

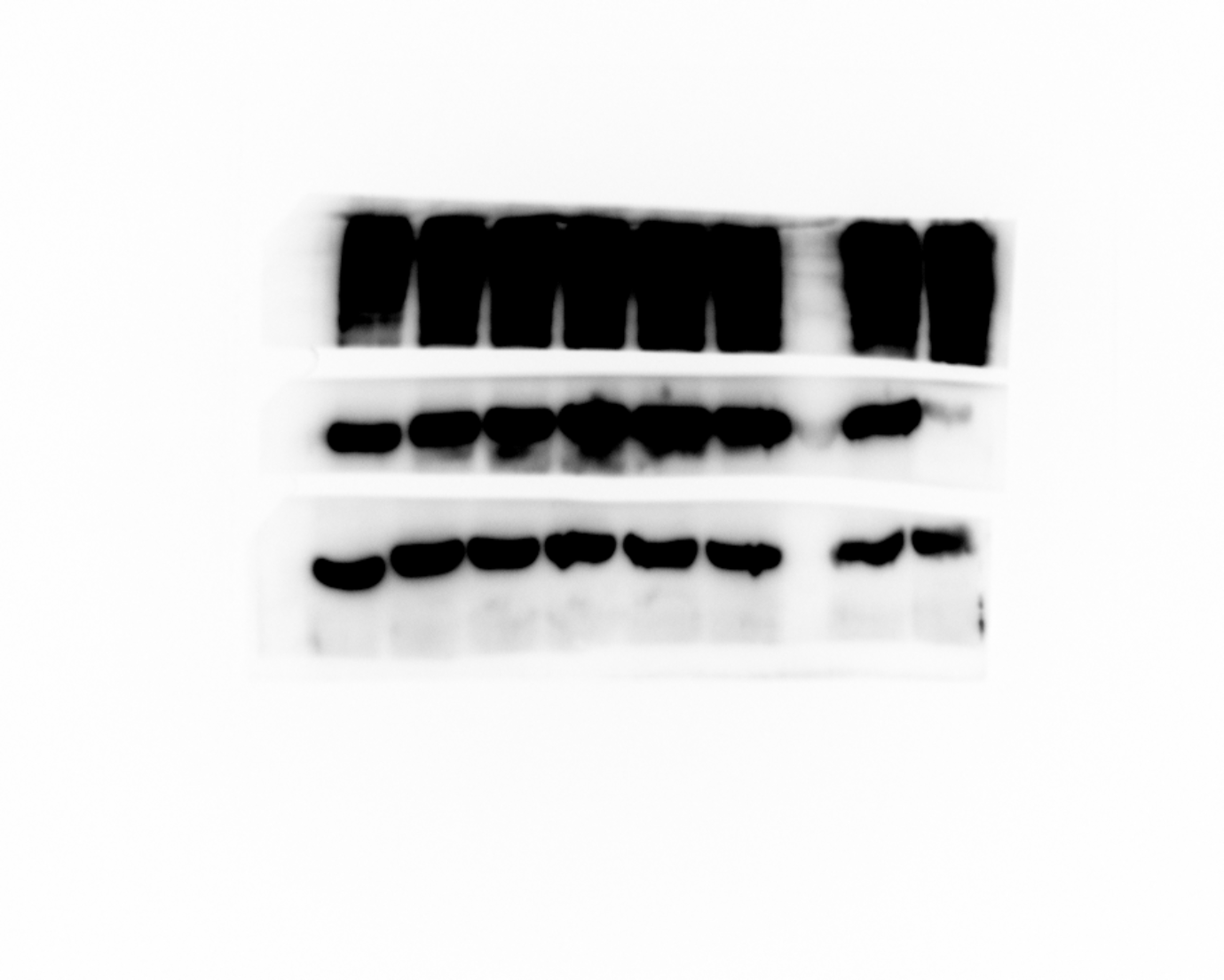

Supplement: Figure 3—source data 1. — Including uncropped Western blot images and raw statistics. [file elife-76436-fig3-data1.zip › Figure 3-Source Data 1/Figure 3L full raw unedited/Input-IB-Actin.tif]

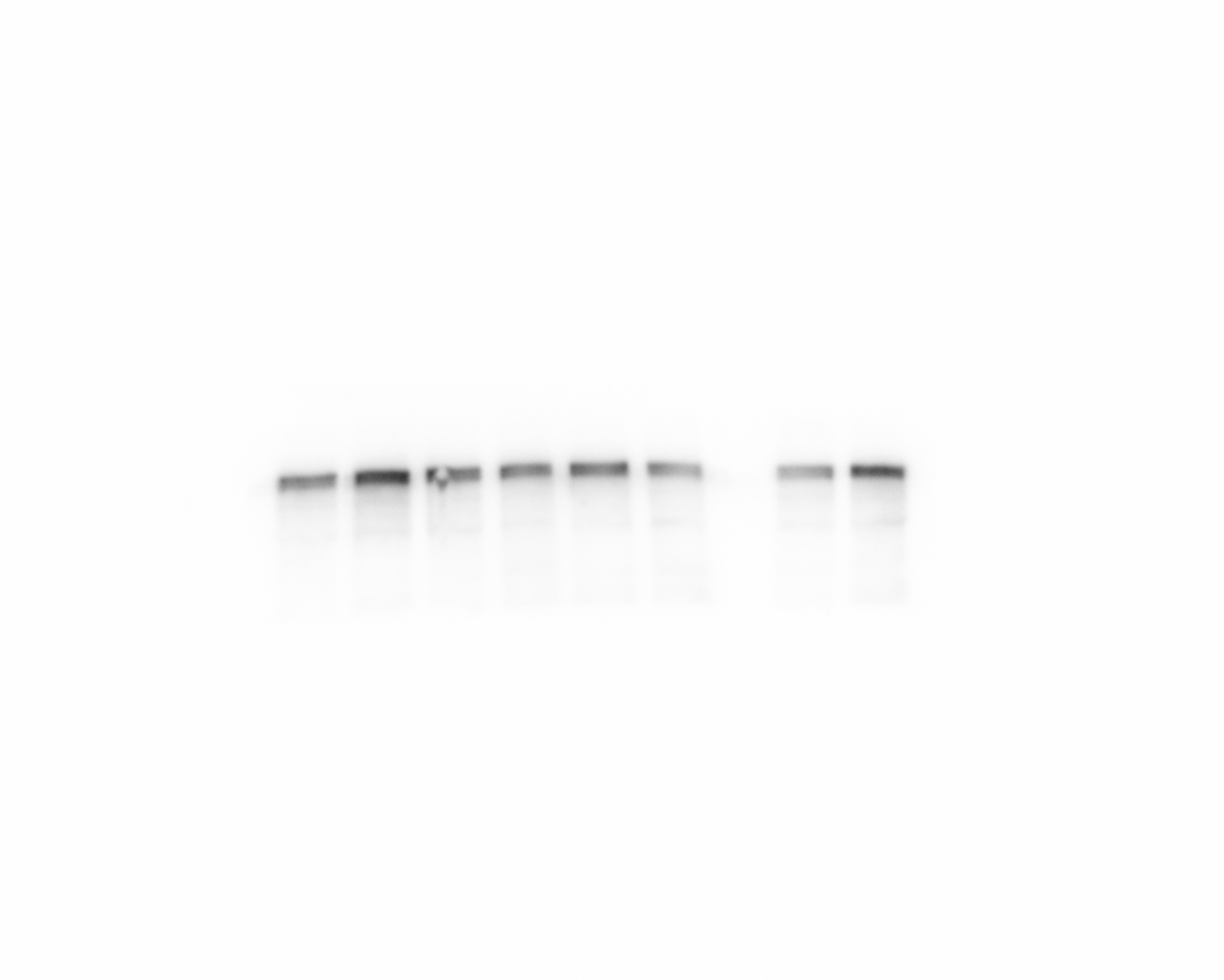

Supplement: Figure 3—source data 1. — Including uncropped Western blot images and raw statistics. [file elife-76436-fig3-data1.zip › Figure 3-Source Data 1/Figure 3L full raw unedited/Input-IB-FLAG.tif]

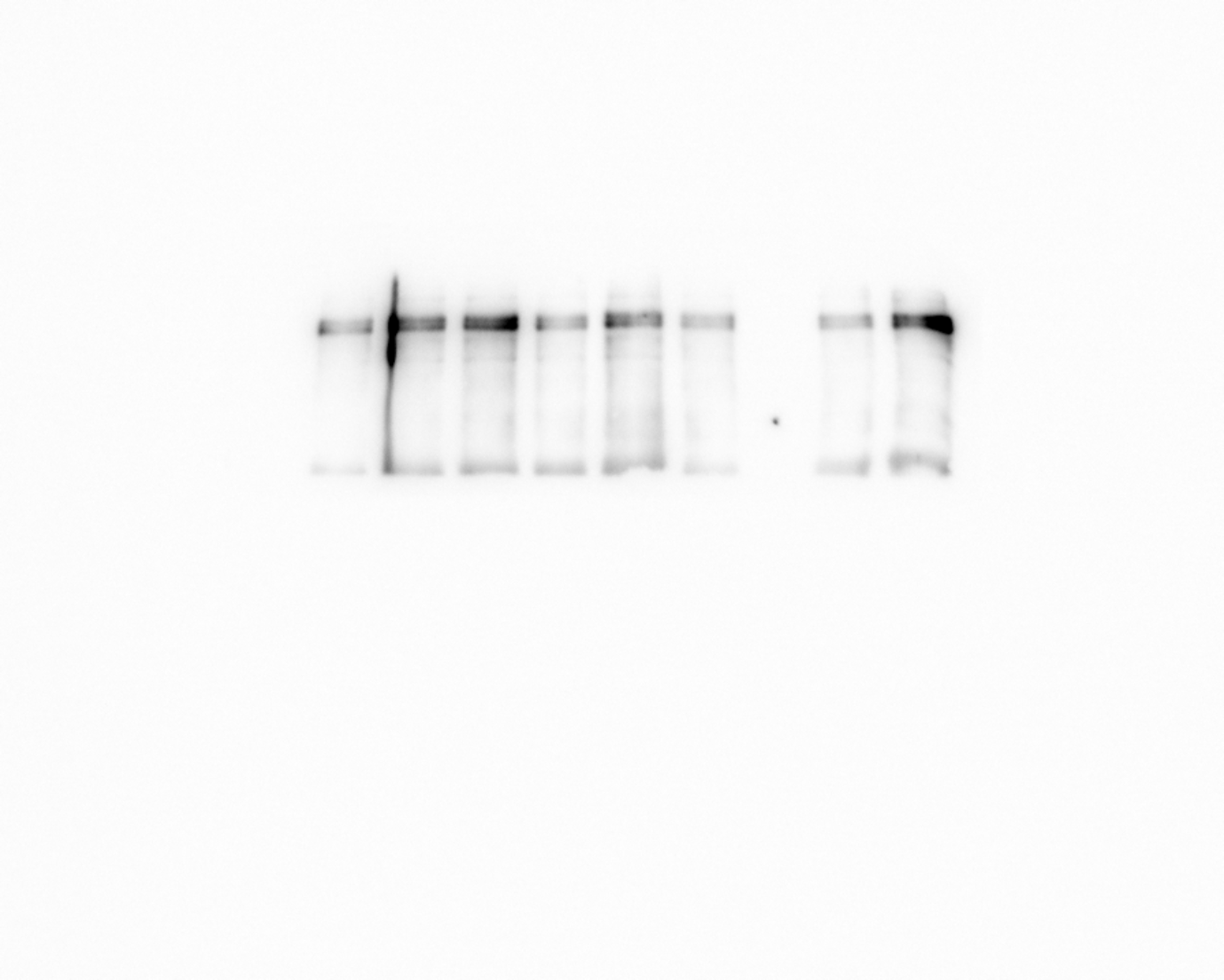

Supplement: Figure 3—source data 1. — Including uncropped Western blot images and raw statistics. [file elife-76436-fig3-data1.zip › Figure 3-Source Data 1/Figure 3L full raw unedited/IP-IB-FLAG.tif]

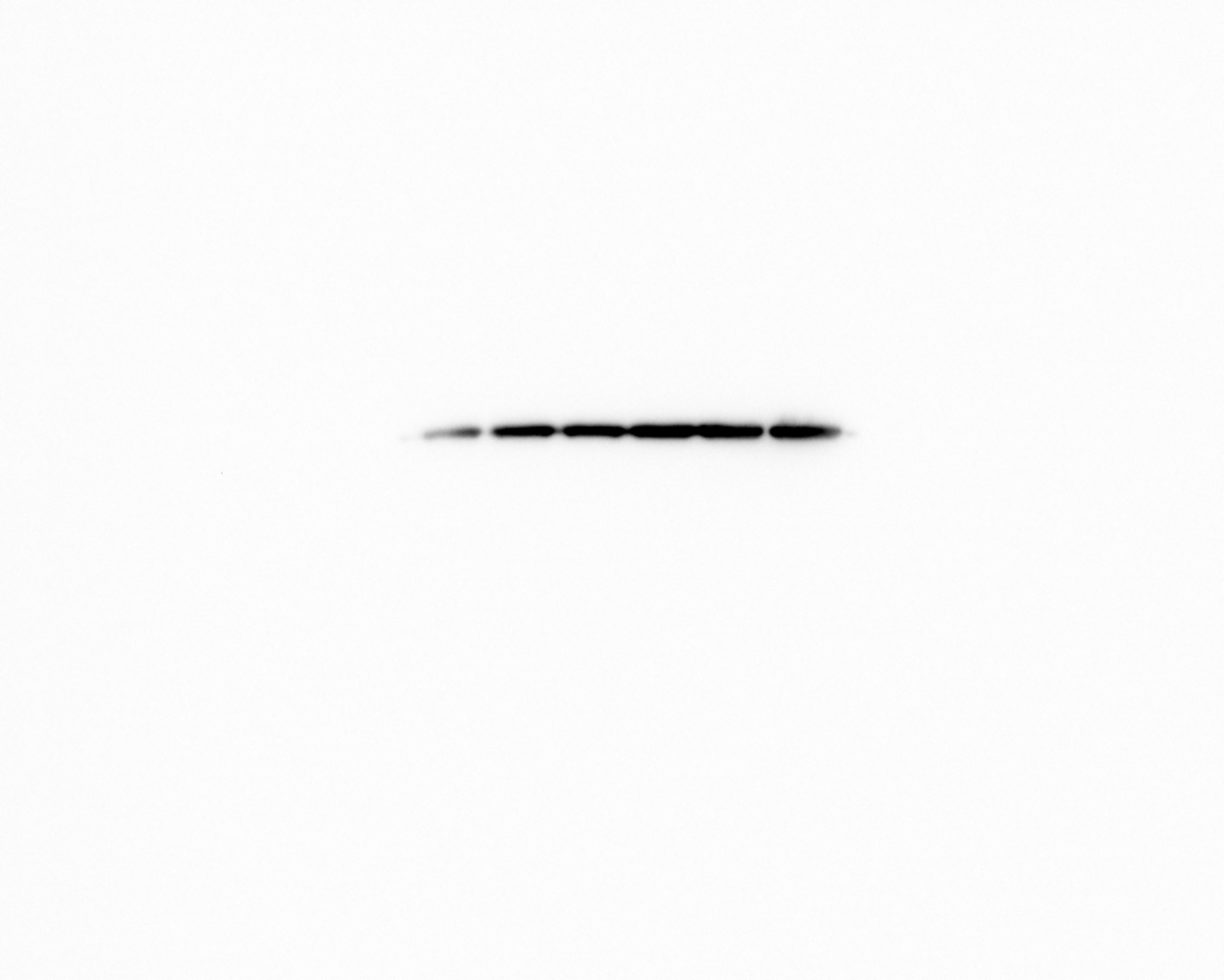

Supplement: Figure 3—figure supplement 1—source data 1. — Including uncropped Western blot images and raw statistics. [file elife-76436-fig3-figsupp1-data1.zip › Figure 3-figure supplement 1-Source Data 1/Figure 3-figure supplement 1A full raw unedited/IB-Actin-control-ttr-52 RNAi.tif]

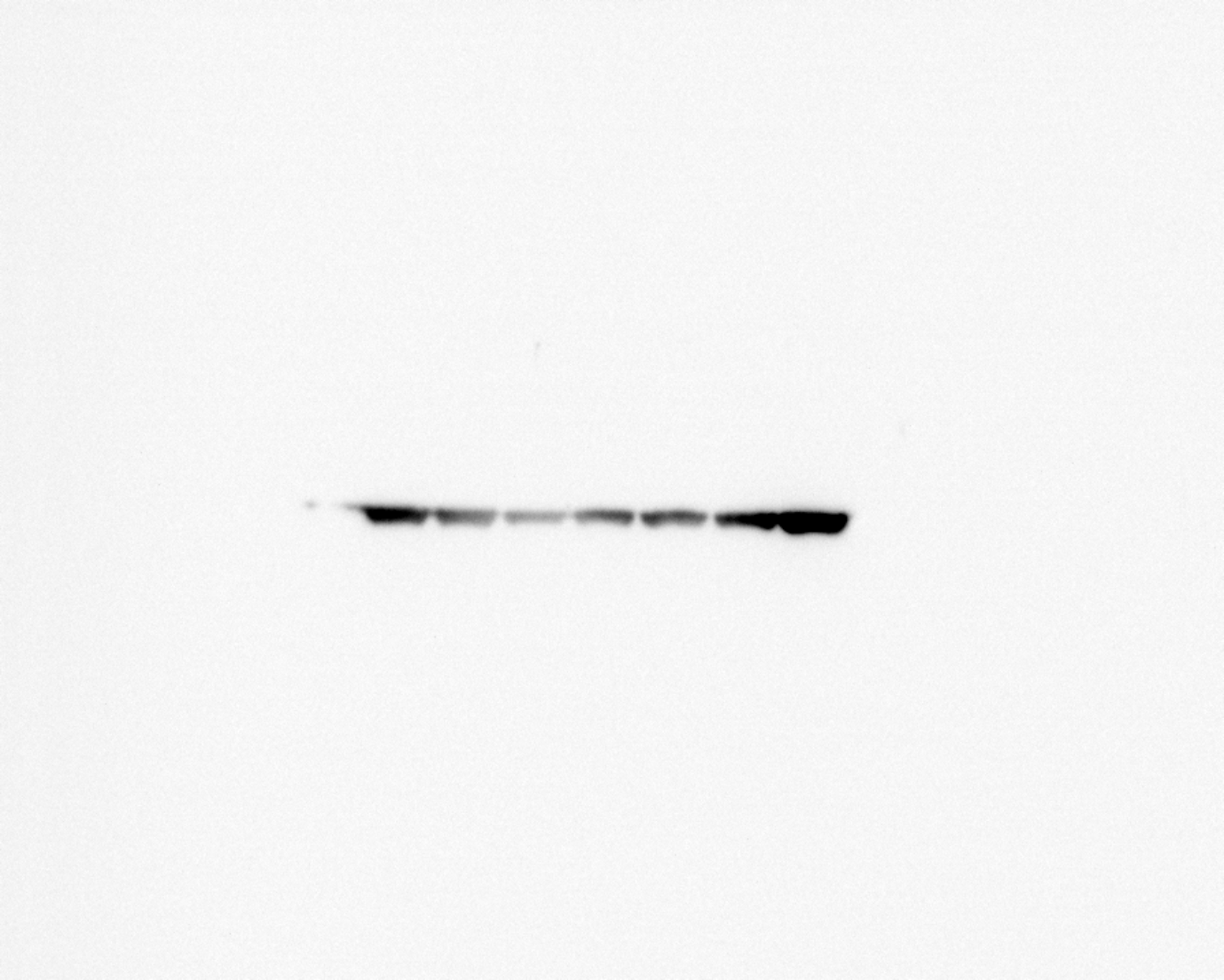

Supplement: Figure 3—figure supplement 1—source data 1. — Including uncropped Western blot images and raw statistics. [file elife-76436-fig3-figsupp1-data1.zip › Figure 3-figure supplement 1-Source Data 1/Figure 3-figure supplement 1A full raw unedited/IB-Actin-N2-ced-7.tif]

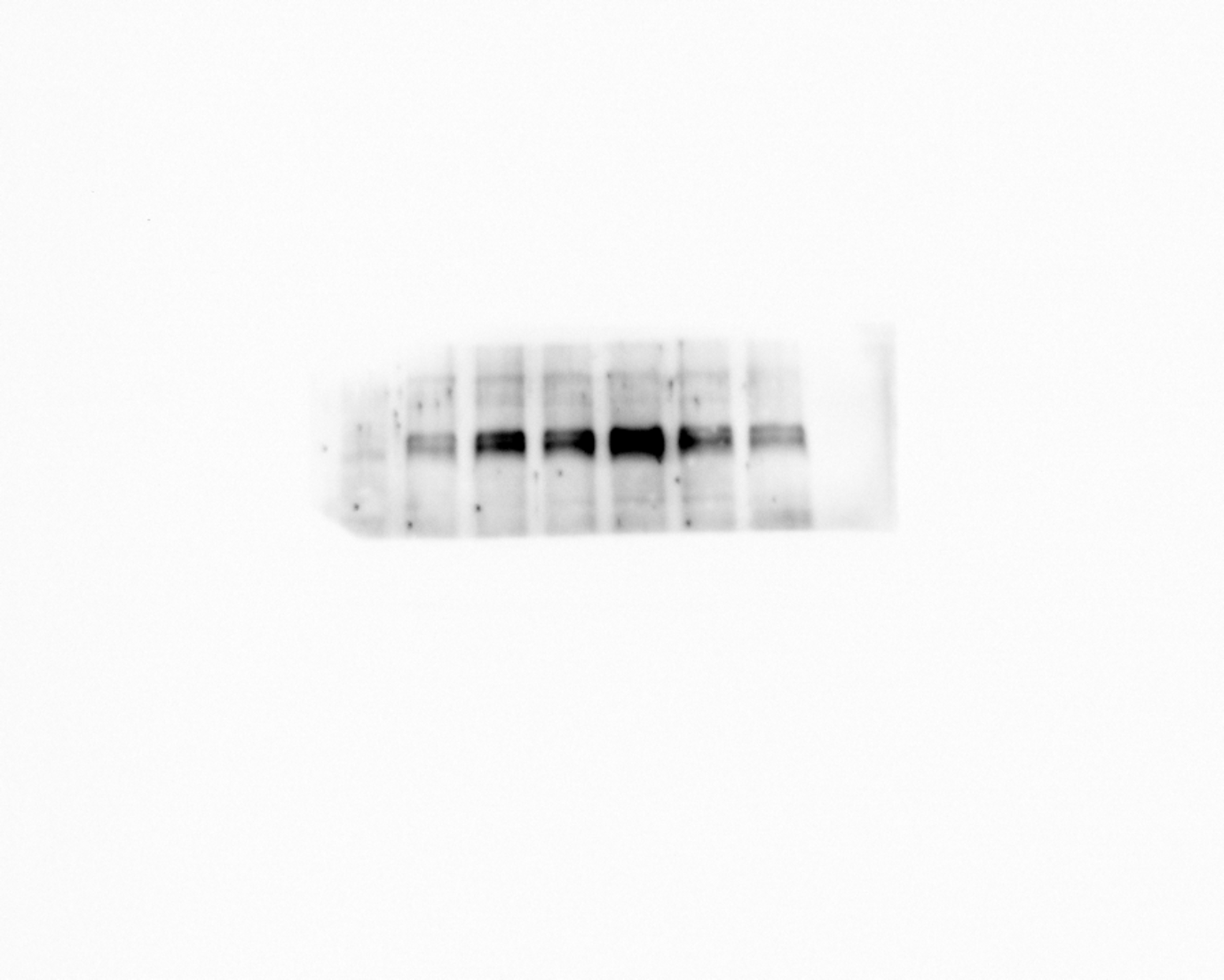

Supplement: Figure 3—figure supplement 1—source data 1. — Including uncropped Western blot images and raw statistics. [file elife-76436-fig3-figsupp1-data1.zip › Figure 3-figure supplement 1-Source Data 1/Figure 3-figure supplement 1A full raw unedited/IB-CED-1-control-ttr-52 RNAi.tif]

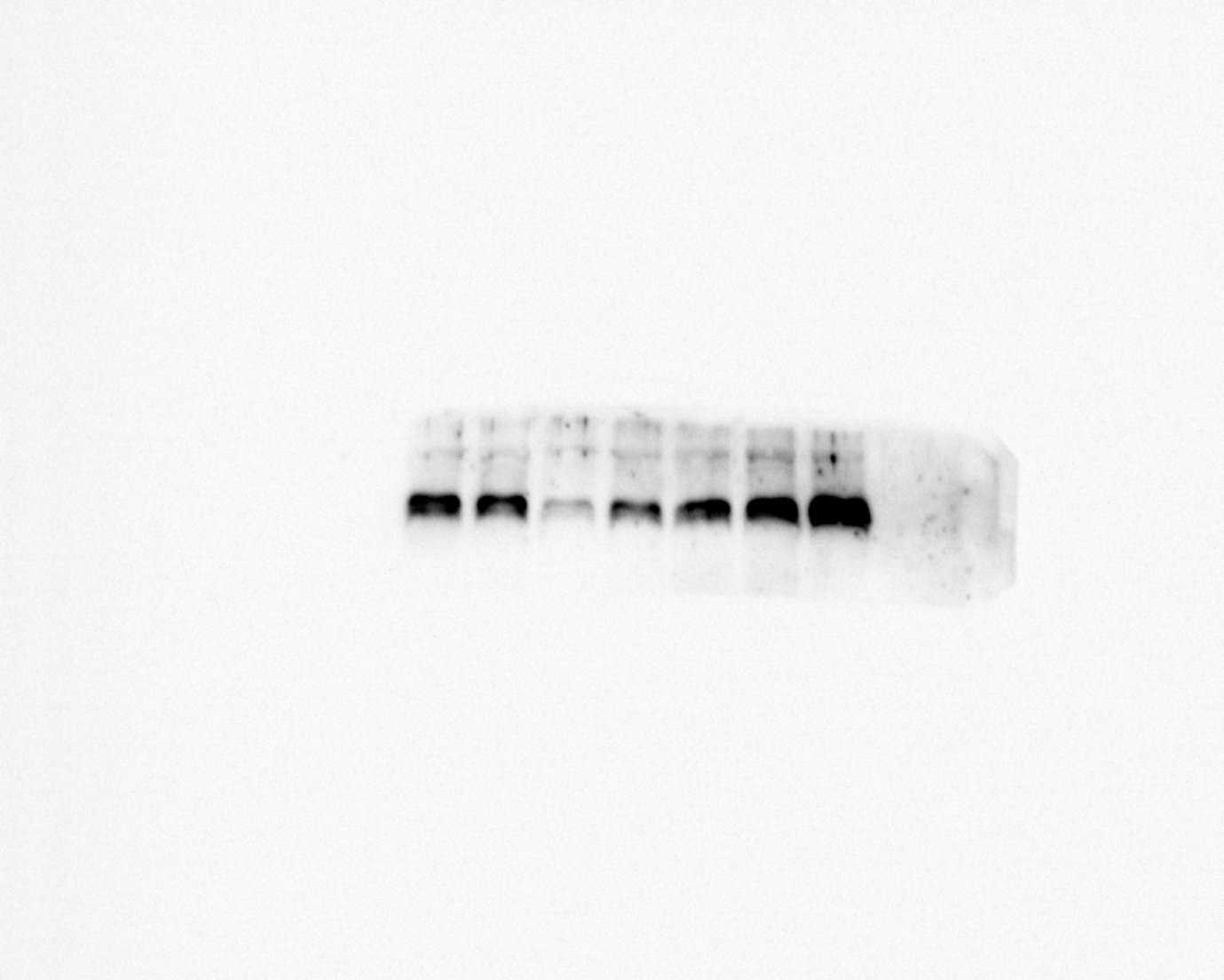

Supplement: Figure 3—figure supplement 1—source data 1. — Including uncropped Western blot images and raw statistics. [file elife-76436-fig3-figsupp1-data1.zip › Figure 3-figure supplement 1-Source Data 1/Figure 3-figure supplement 1A full raw unedited/IB-CED-1-N2-ced-7.tif]

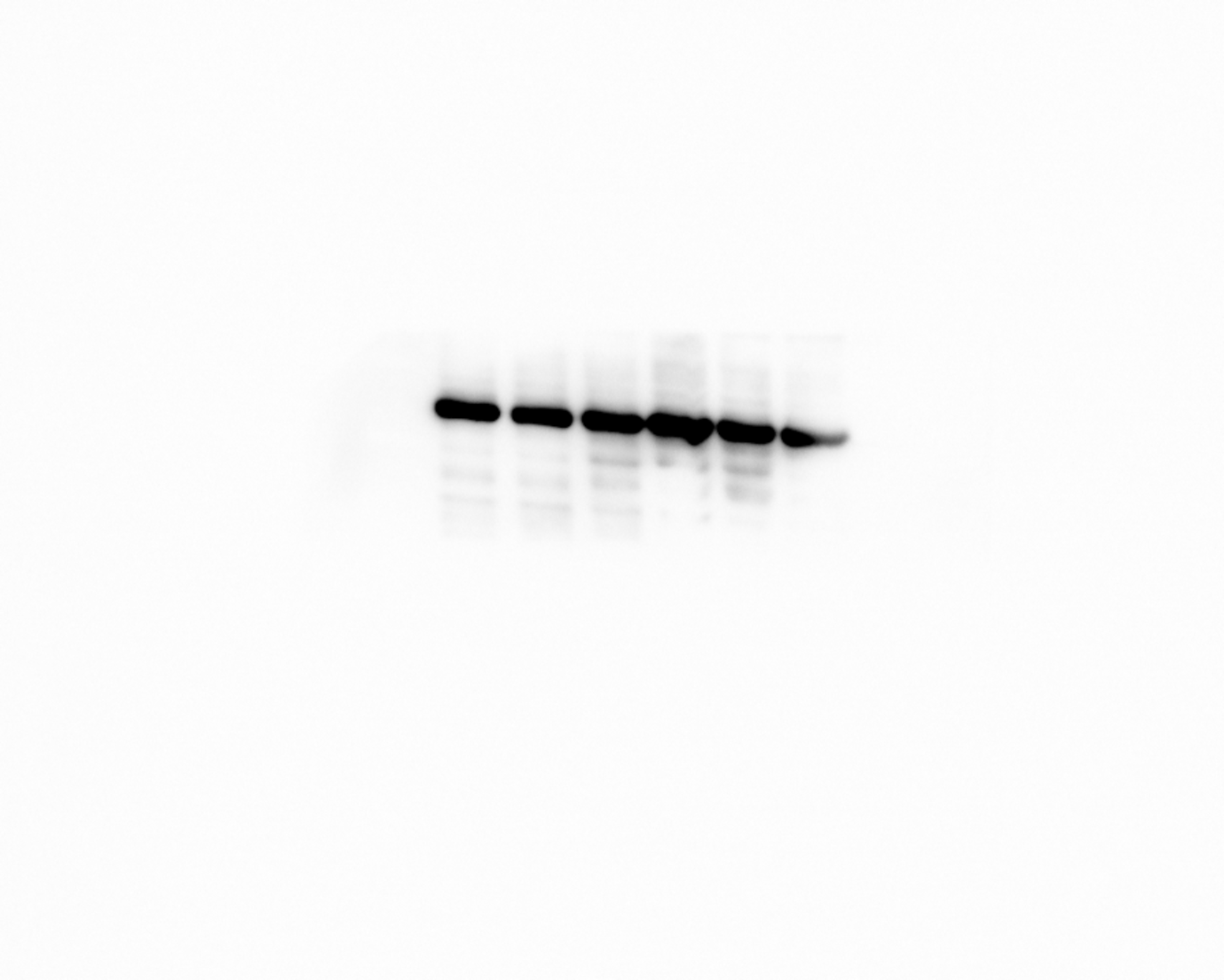

Supplement: Figure 3—figure supplement 1—source data 1. — Including uncropped Western blot images and raw statistics. [file elife-76436-fig3-figsupp1-data1.zip › Figure 3-figure supplement 1-Source Data 1/Figure 3-figure supplement 1B full raw unedited/IB-Actin-control-ap-2 RNAi.tif]

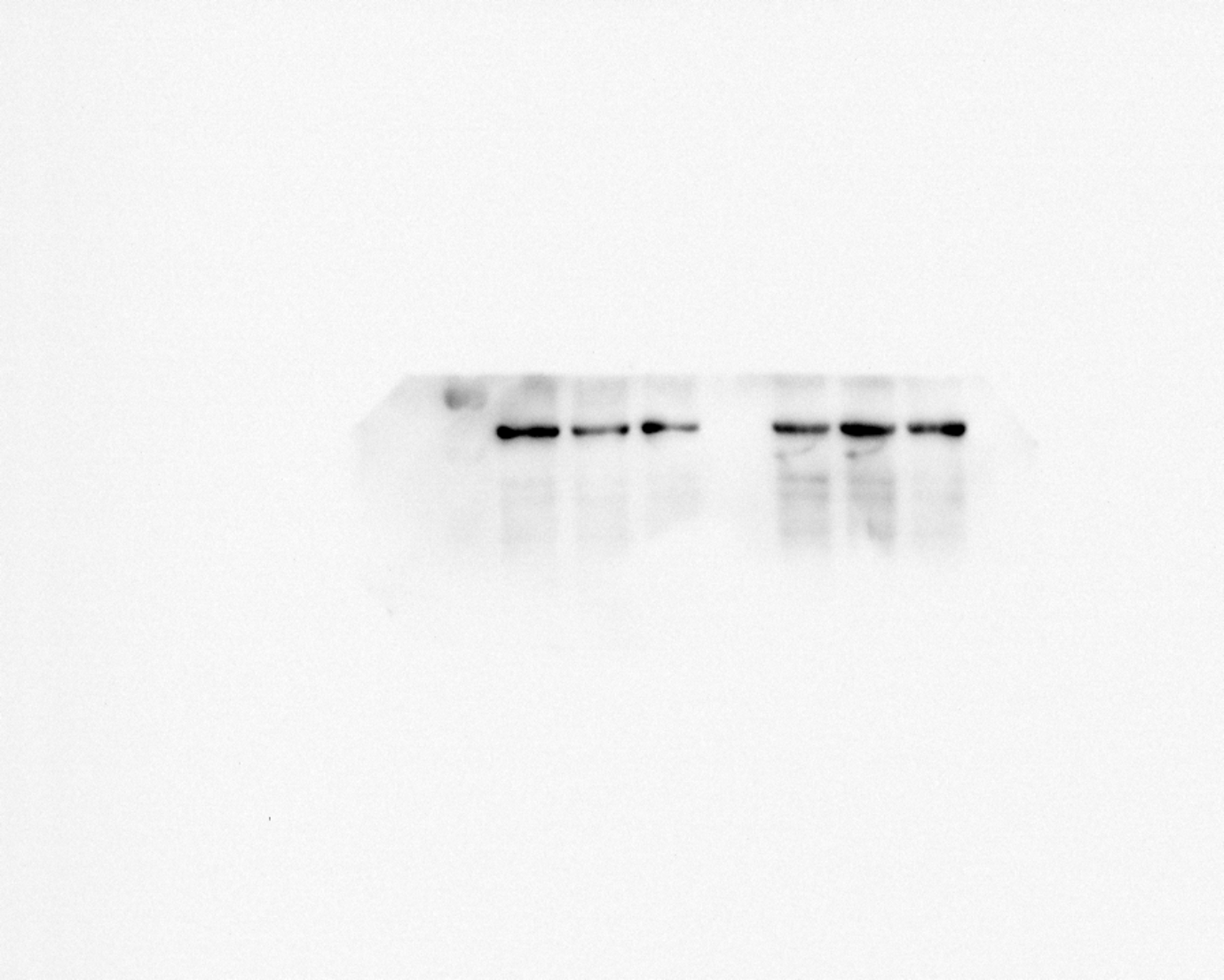

Supplement: Figure 3—figure supplement 1—source data 1. — Including uncropped Western blot images and raw statistics. [file elife-76436-fig3-figsupp1-data1.zip › Figure 3-figure supplement 1-Source Data 1/Figure 3-figure supplement 1B full raw unedited/IB-Actin-control-dyn-1 RNAi.tif]

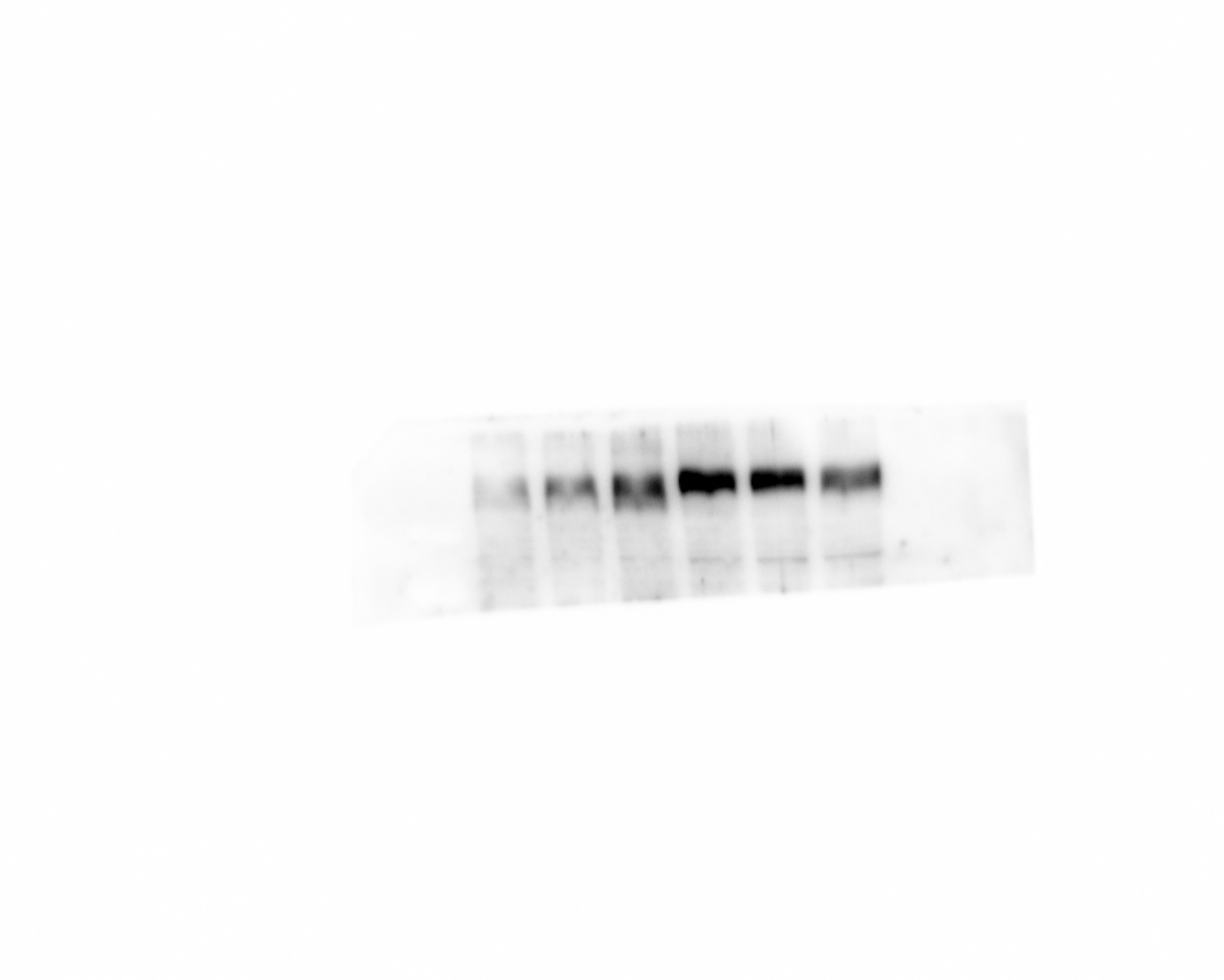

Supplement: Figure 3—figure supplement 1—source data 1. — Including uncropped Western blot images and raw statistics. [file elife-76436-fig3-figsupp1-data1.zip › Figure 3-figure supplement 1-Source Data 1/Figure 3-figure supplement 1B full raw unedited/IB-CED-1-control-ap-2 RNAi.tif]
