# Supplementary figures and images for: trim-21 promotes proteasomal degradation of CED-1 for apoptotic cell clearance in C. elegans (part 2 of 2)
Source: eLife. 2022 Aug 5;11:e76436. doi: 10.7554/eLife.76436 (PMC9388098; doi:10.7554/eLife.76436)

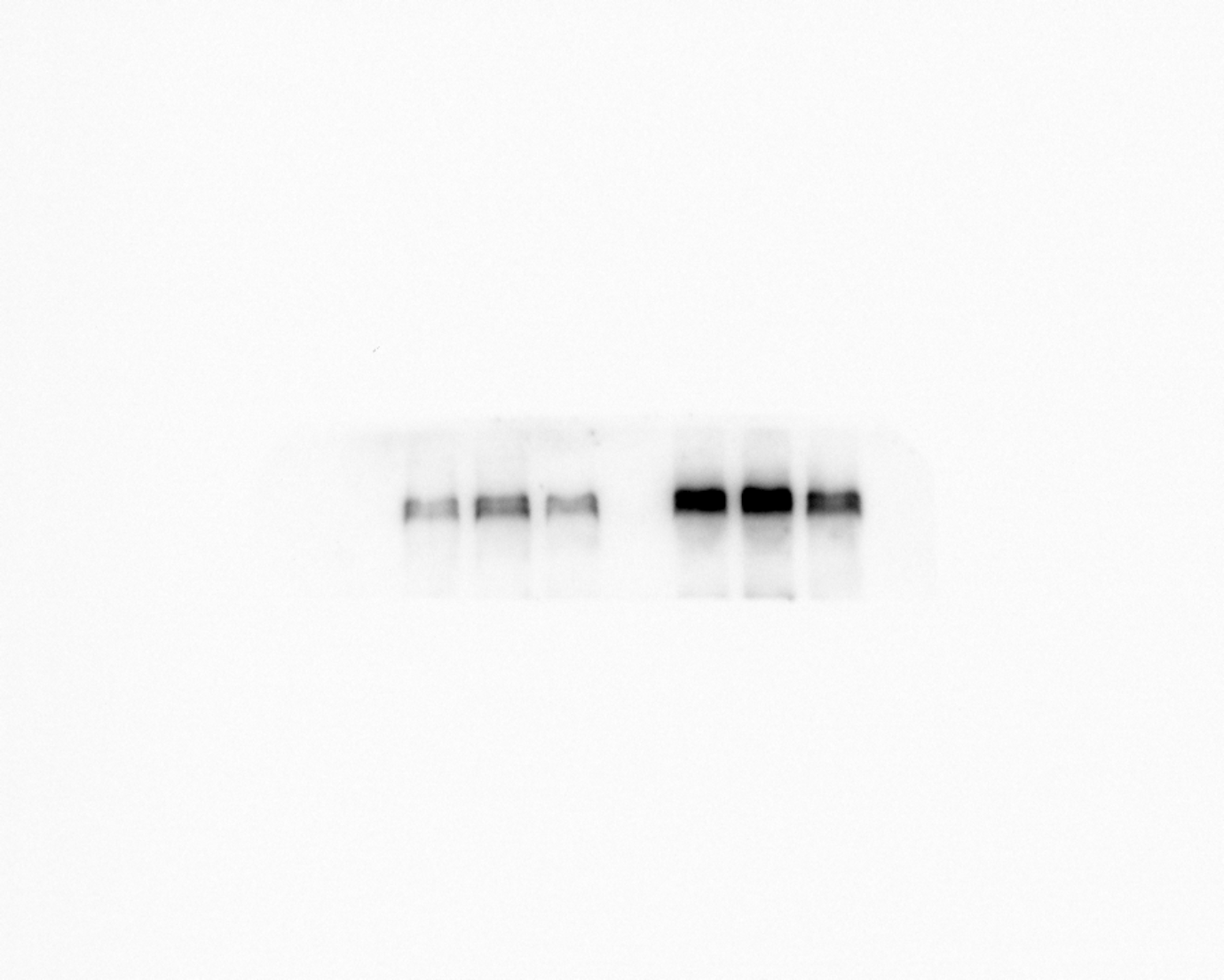

Supplement: Figure 3—figure supplement 1—source data 1. — Including uncropped Western blot images and raw statistics. [file elife-76436-fig3-figsupp1-data1.zip › Figure 3-figure supplement 1-Source Data 1/Figure 3-figure supplement 1B full raw unedited/IB-CED-1-control-dyn-1 RNAi.tif]

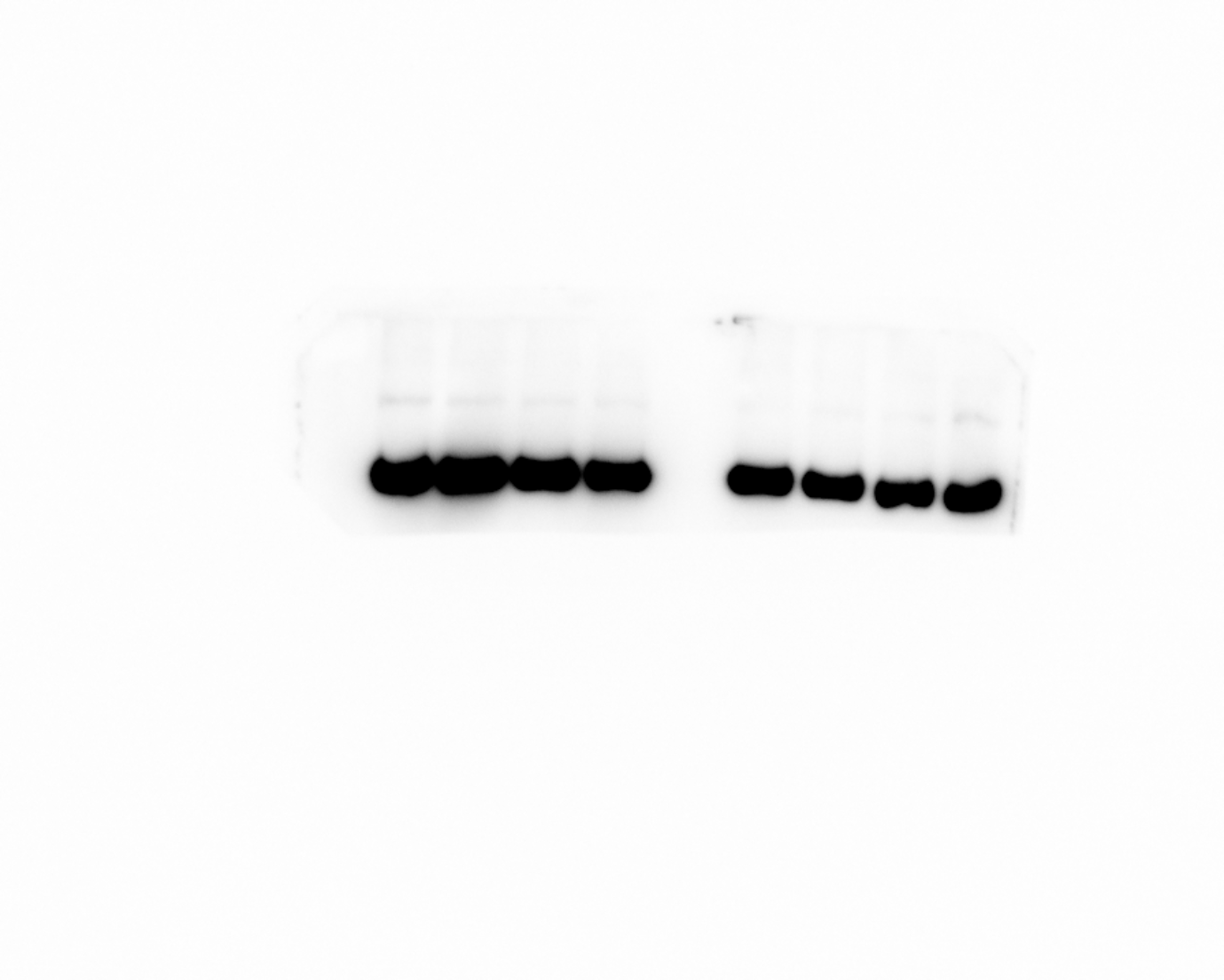

Supplement: Figure 3—figure supplement 1—source data 1. — Including uncropped Western blot images and raw statistics. [file elife-76436-fig3-figsupp1-data1.zip › Figure 3-figure supplement 1-Source Data 1/Figure 3-figure supplement 1D full raw unedited/IB-Actin.tif]

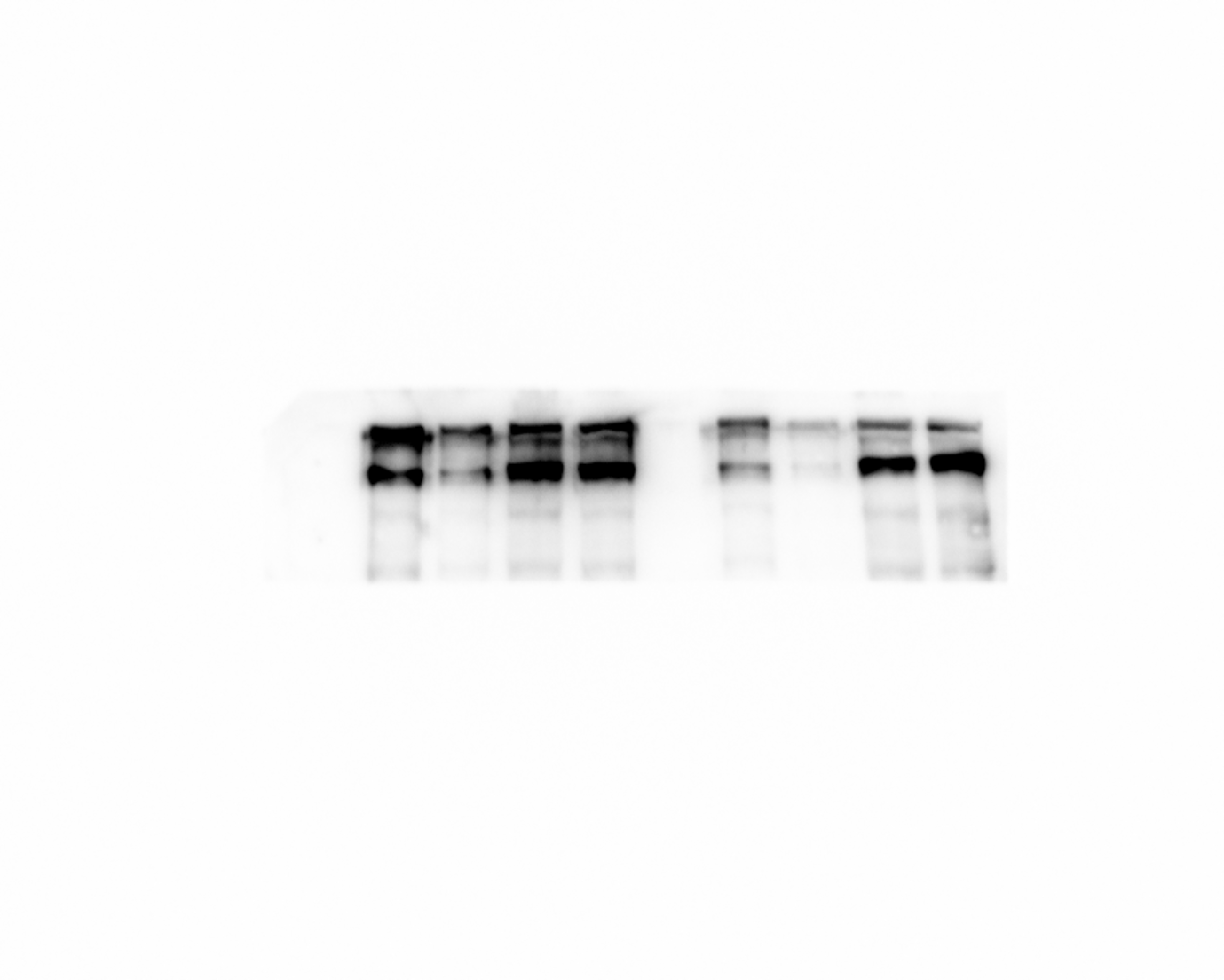

Supplement: Figure 3—figure supplement 1—source data 1. — Including uncropped Western blot images and raw statistics. [file elife-76436-fig3-figsupp1-data1.zip › Figure 3-figure supplement 1-Source Data 1/Figure 3-figure supplement 1D full raw unedited/IB-CED-1.tif]

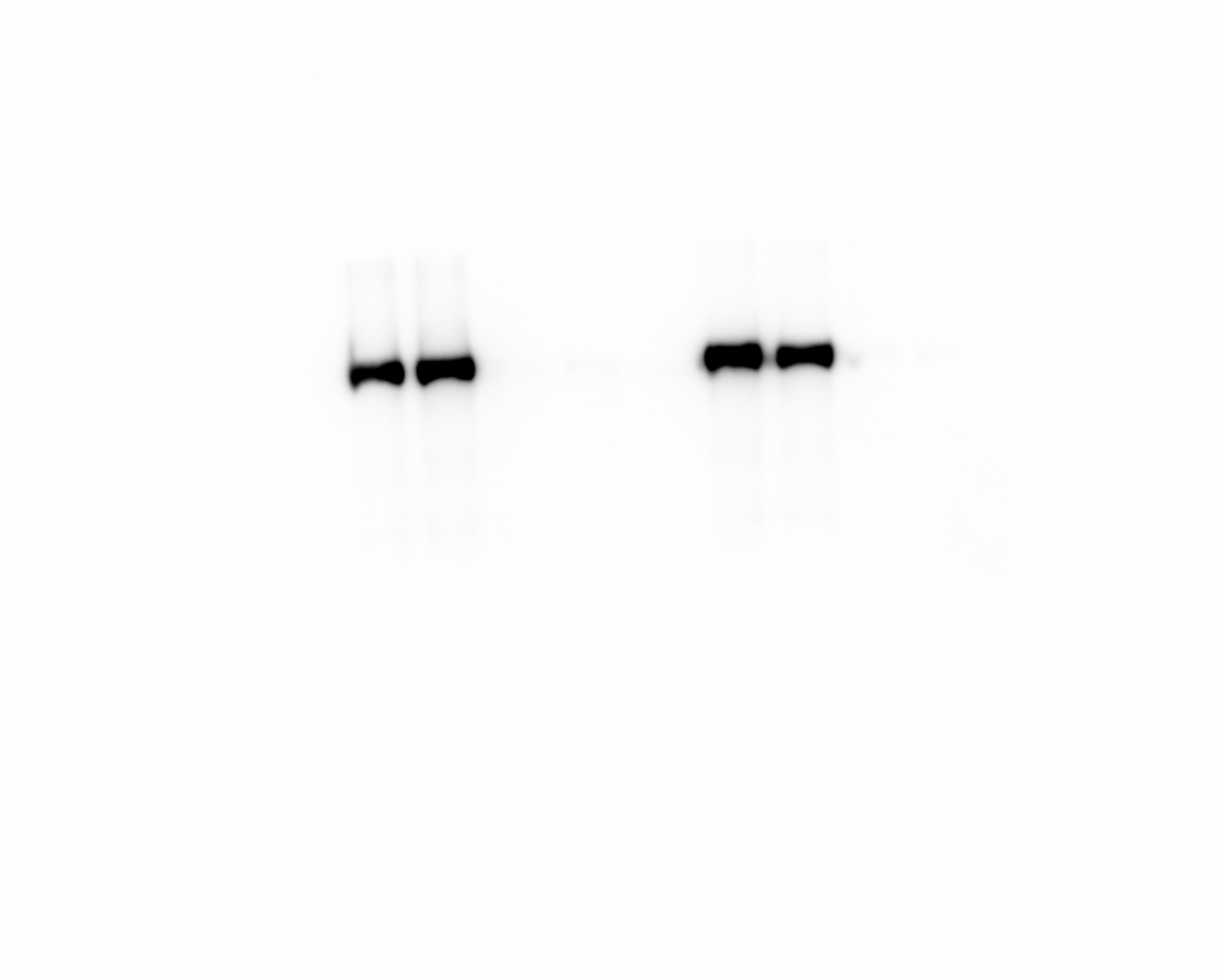

Supplement: Figure 3—figure supplement 1—source data 1. — Including uncropped Western blot images and raw statistics. [file elife-76436-fig3-figsupp1-data1.zip › Figure 3-figure supplement 1-Source Data 1/Figure 3-figure supplement 1D full raw unedited/IB-CED-6.tif]

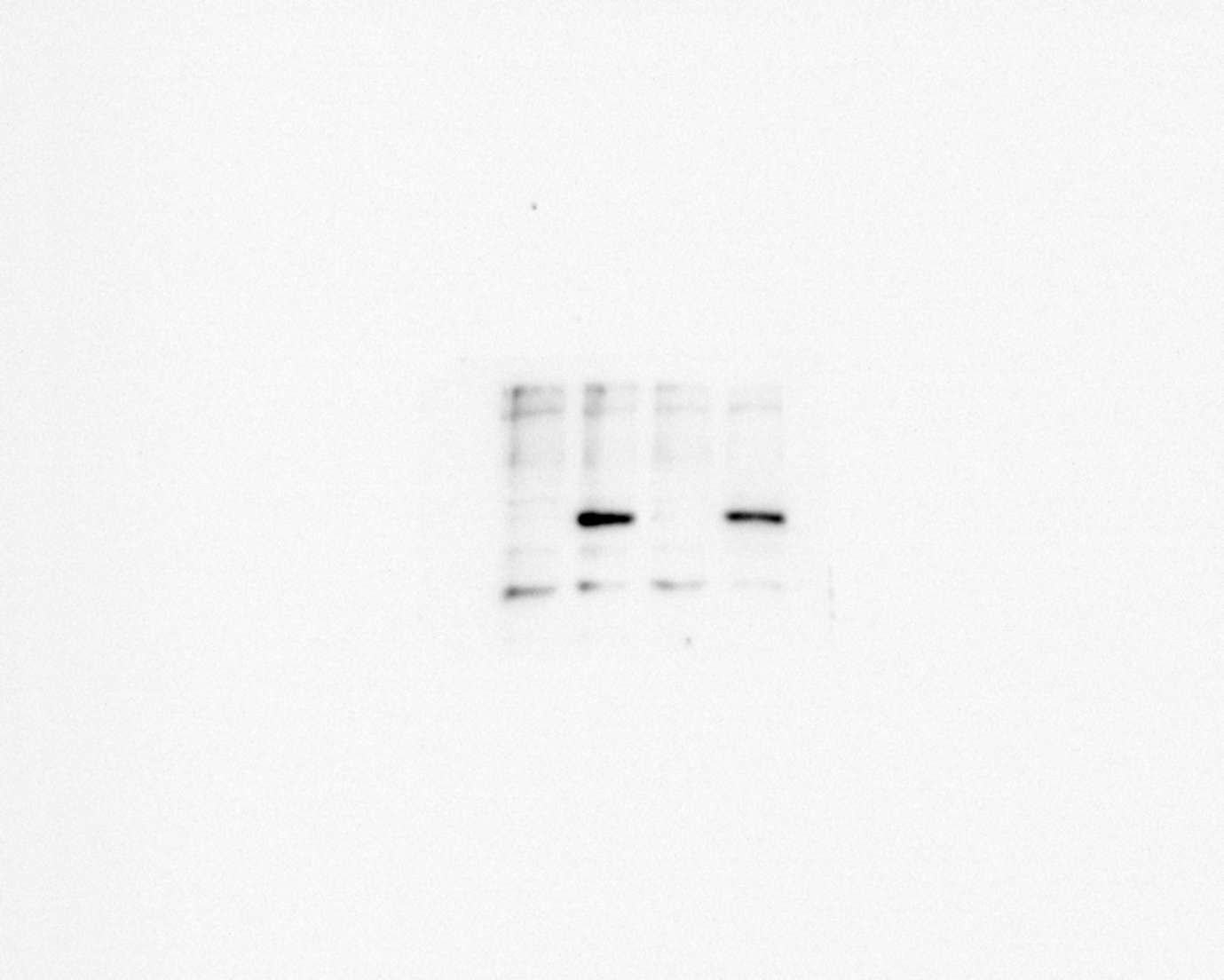

Supplement: Figure 3—figure supplement 1—source data 1. — Including uncropped Western blot images and raw statistics. [file elife-76436-fig3-figsupp1-data1.zip › Figure 3-figure supplement 1-Source Data 1/Figure 3-figure supplement 1D full raw unedited/IB-FLAG.tif]

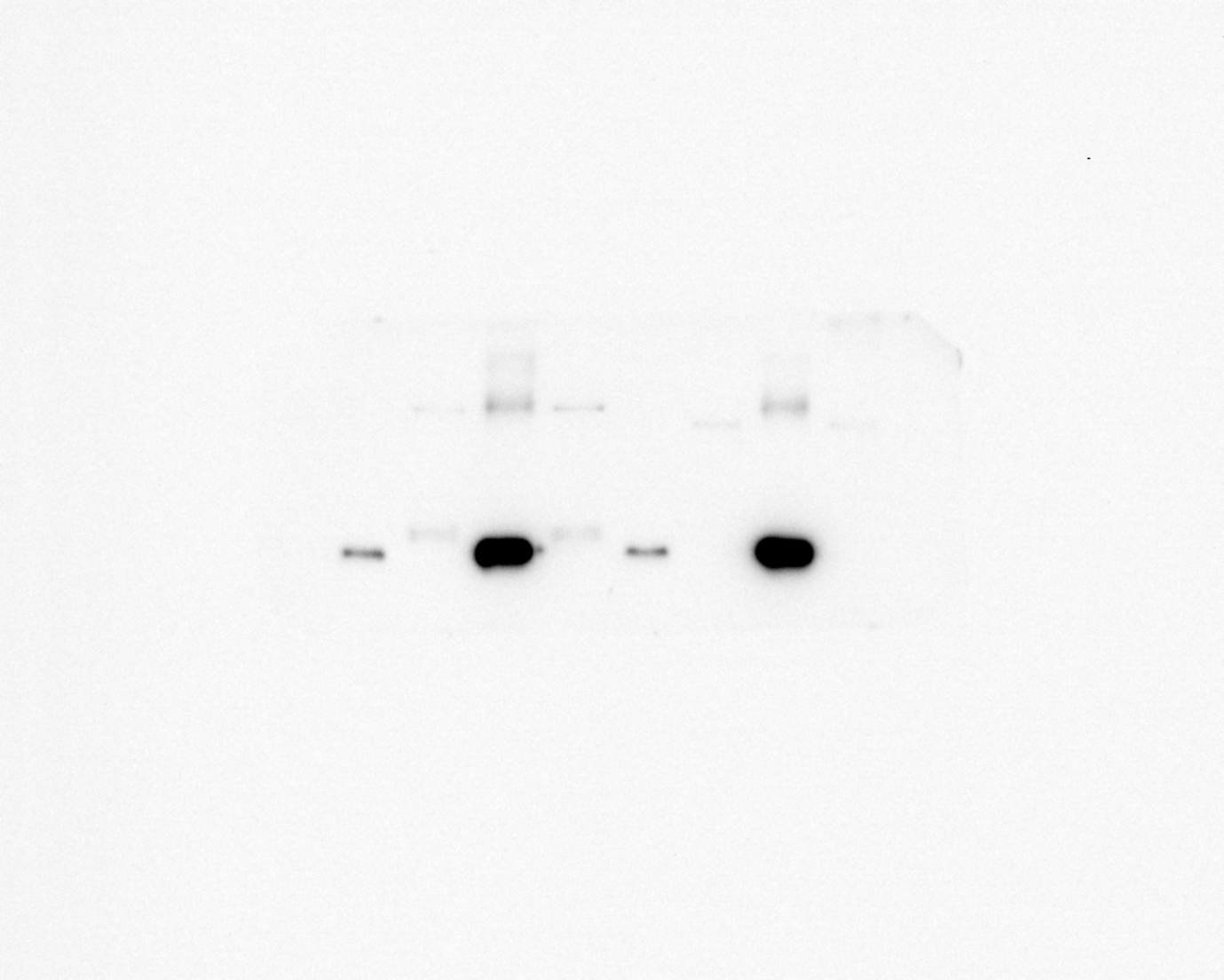

Supplement: Figure 3—figure supplement 1—source data 1. — Including uncropped Western blot images and raw statistics. [file elife-76436-fig3-figsupp1-data1.zip › Figure 3-figure supplement 1-Source Data 1/Figure 3-figure supplement 1E full raw unedited/IB-GST.tif]

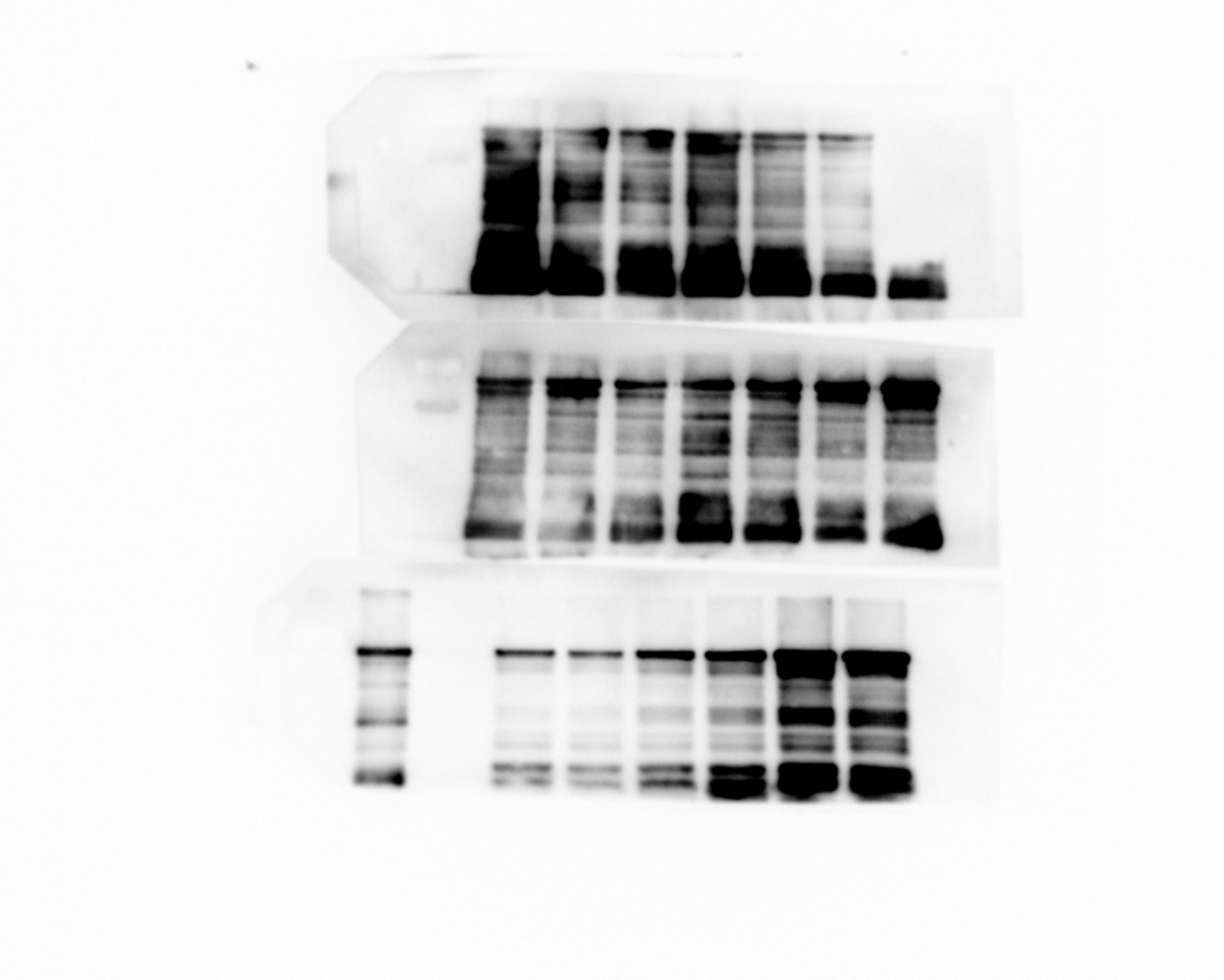

Supplement: Figure 3—figure supplement 1—source data 1. — Including uncropped Western blot images and raw statistics. [file elife-76436-fig3-figsupp1-data1.zip › Figure 3-figure supplement 1-Source Data 1/Figure 3-figure supplement 1H full raw unedited/IB-HA.tif]

Figure 4B


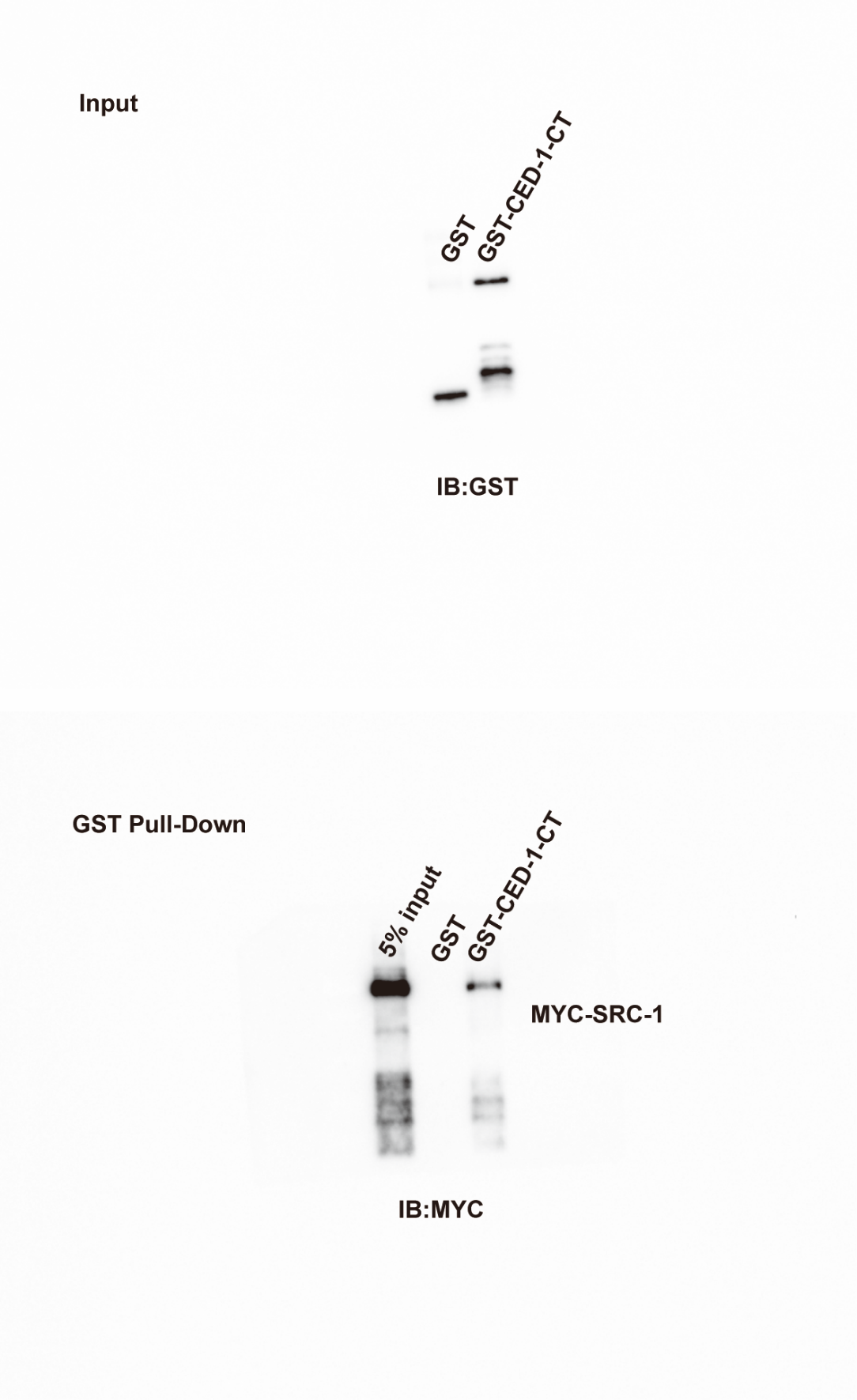


Figure 4C


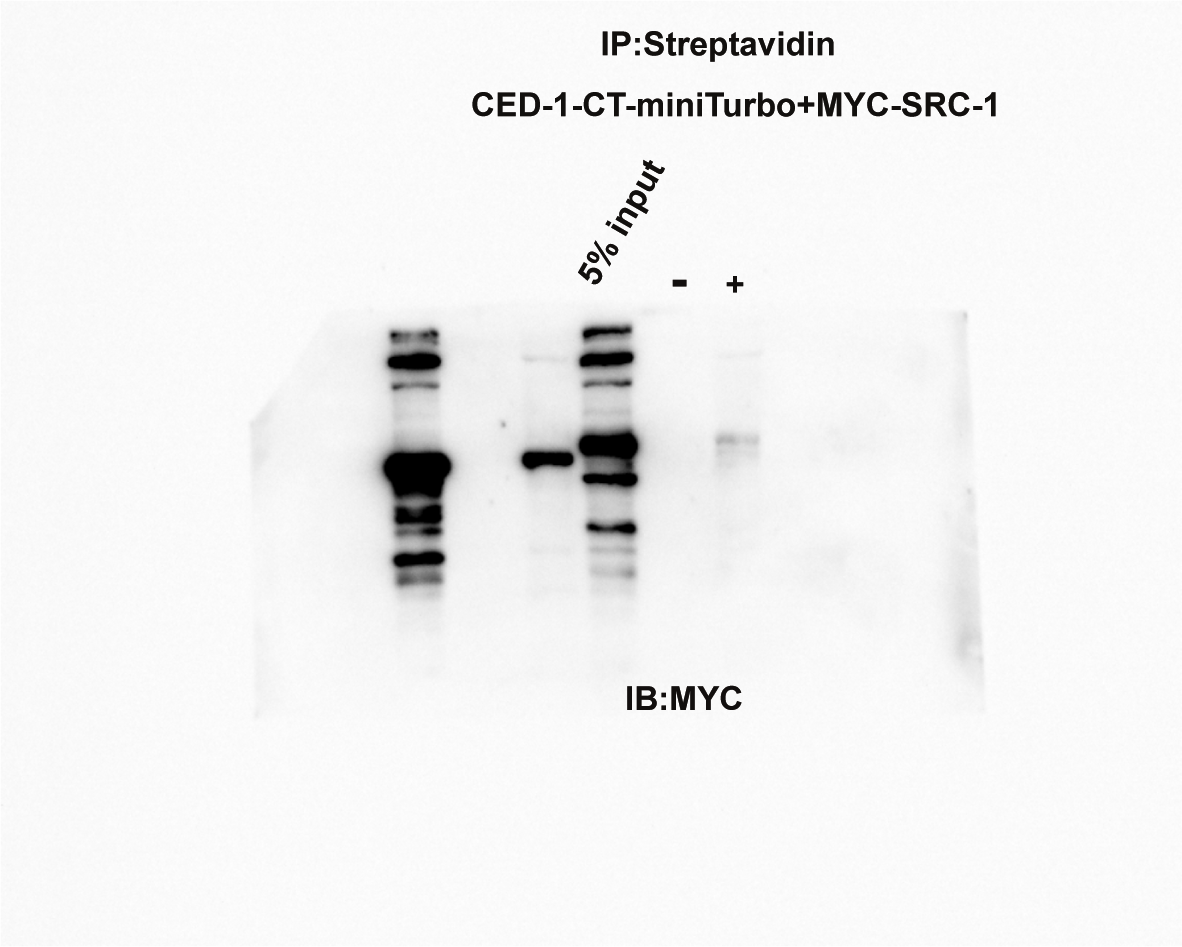


Figure 4E


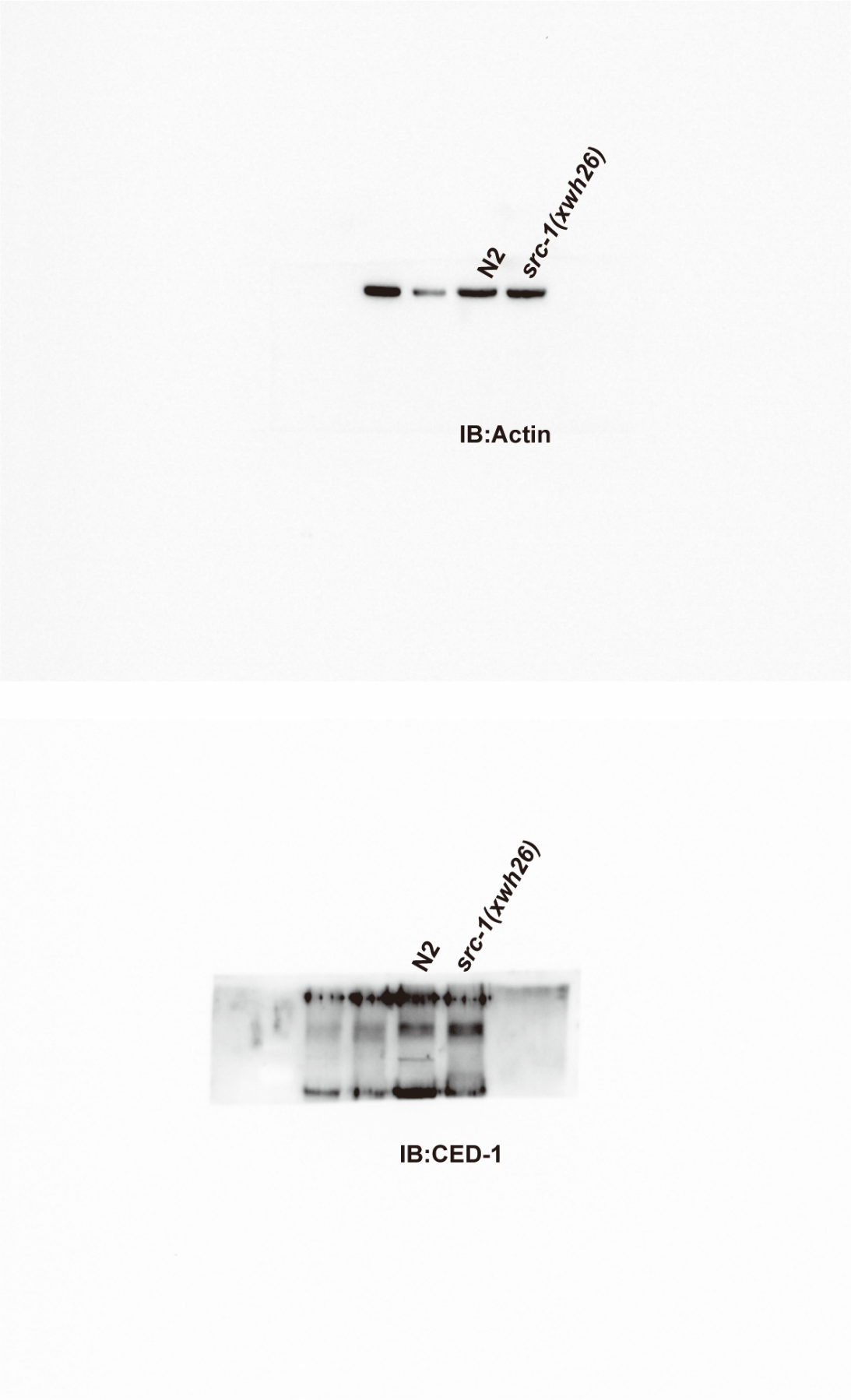


Figure 4F


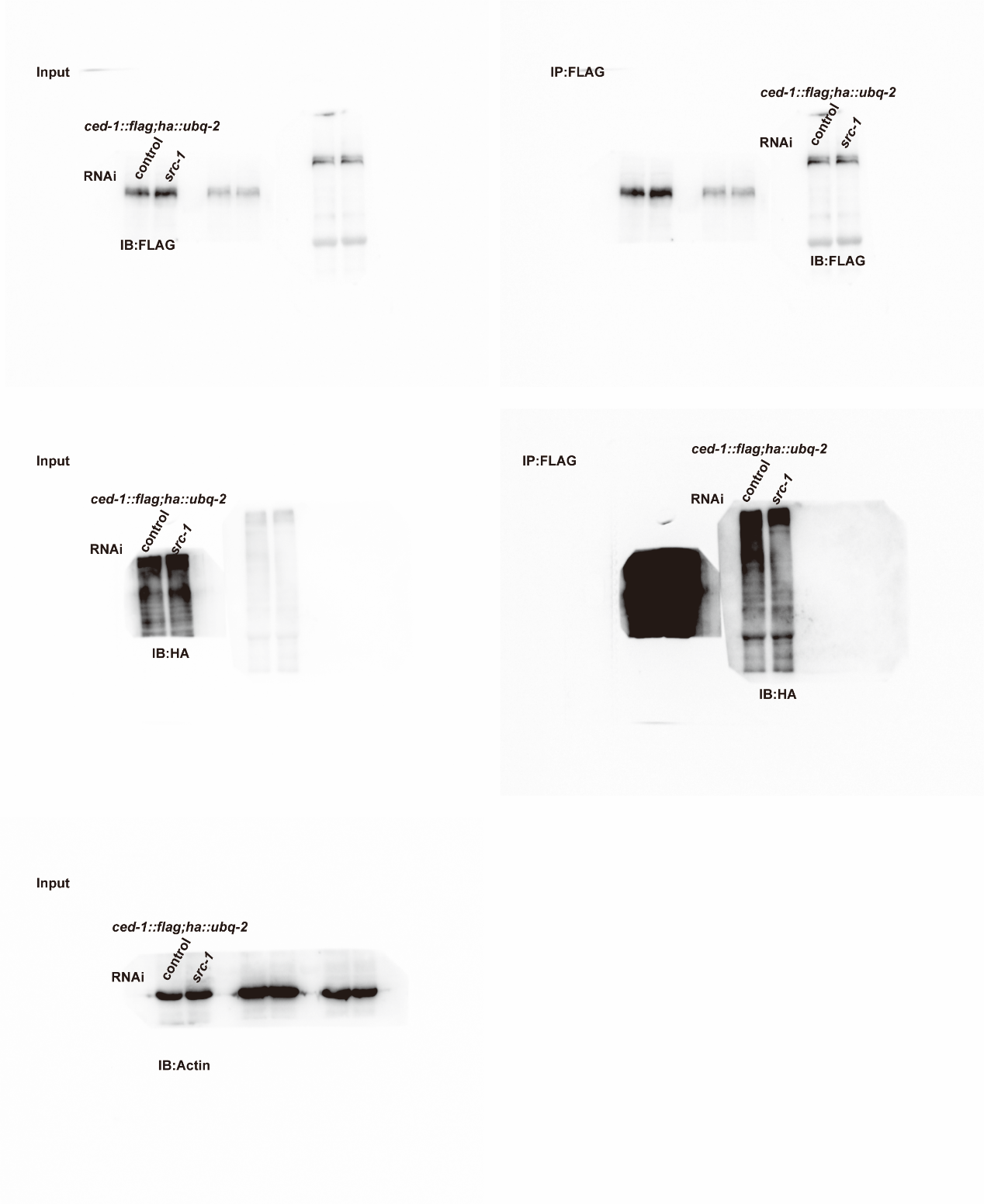


Figure 4I


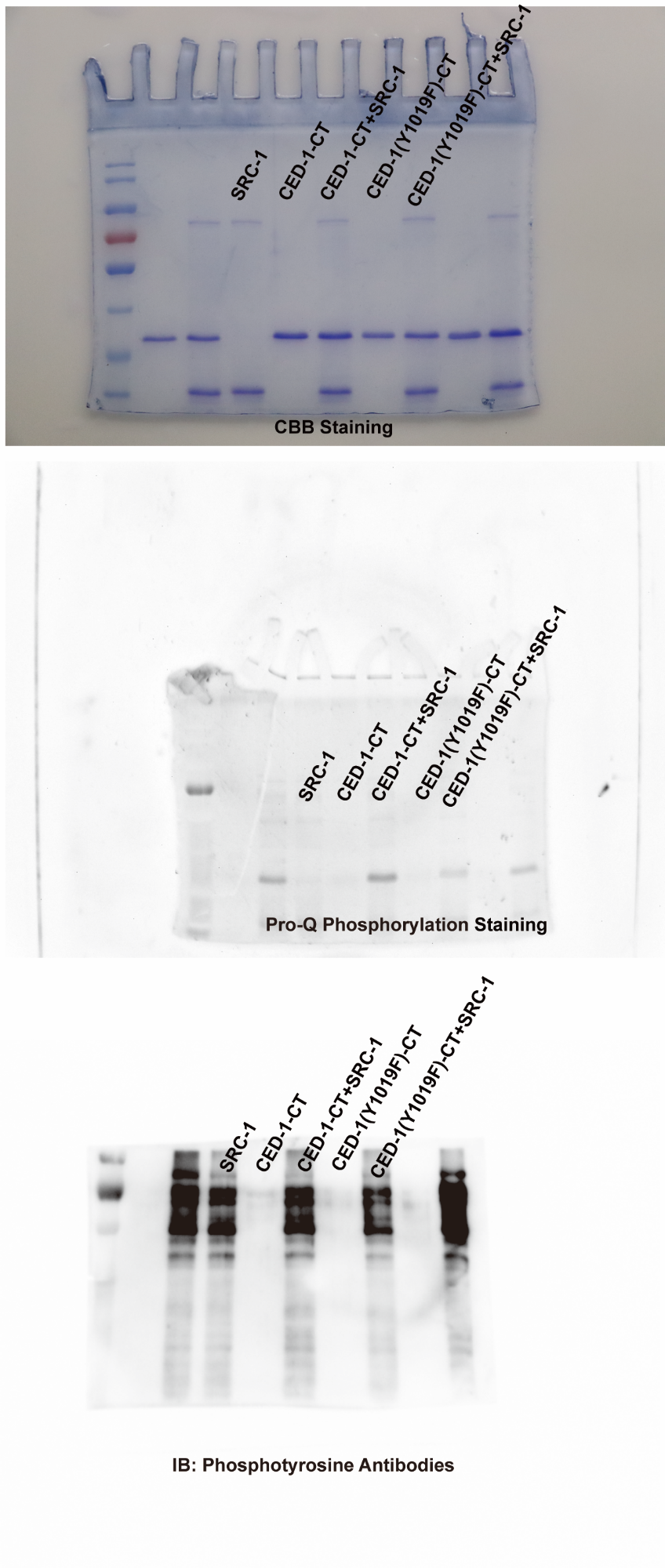

Supplement: Figure 4—source data 1. — Including uncropped Western blot images and raw statistics. [file elife-76436-fig4-data1.zip › Figure 4-Source Data 1/Figure 4 uncroppped blot with relevant bands.docx]

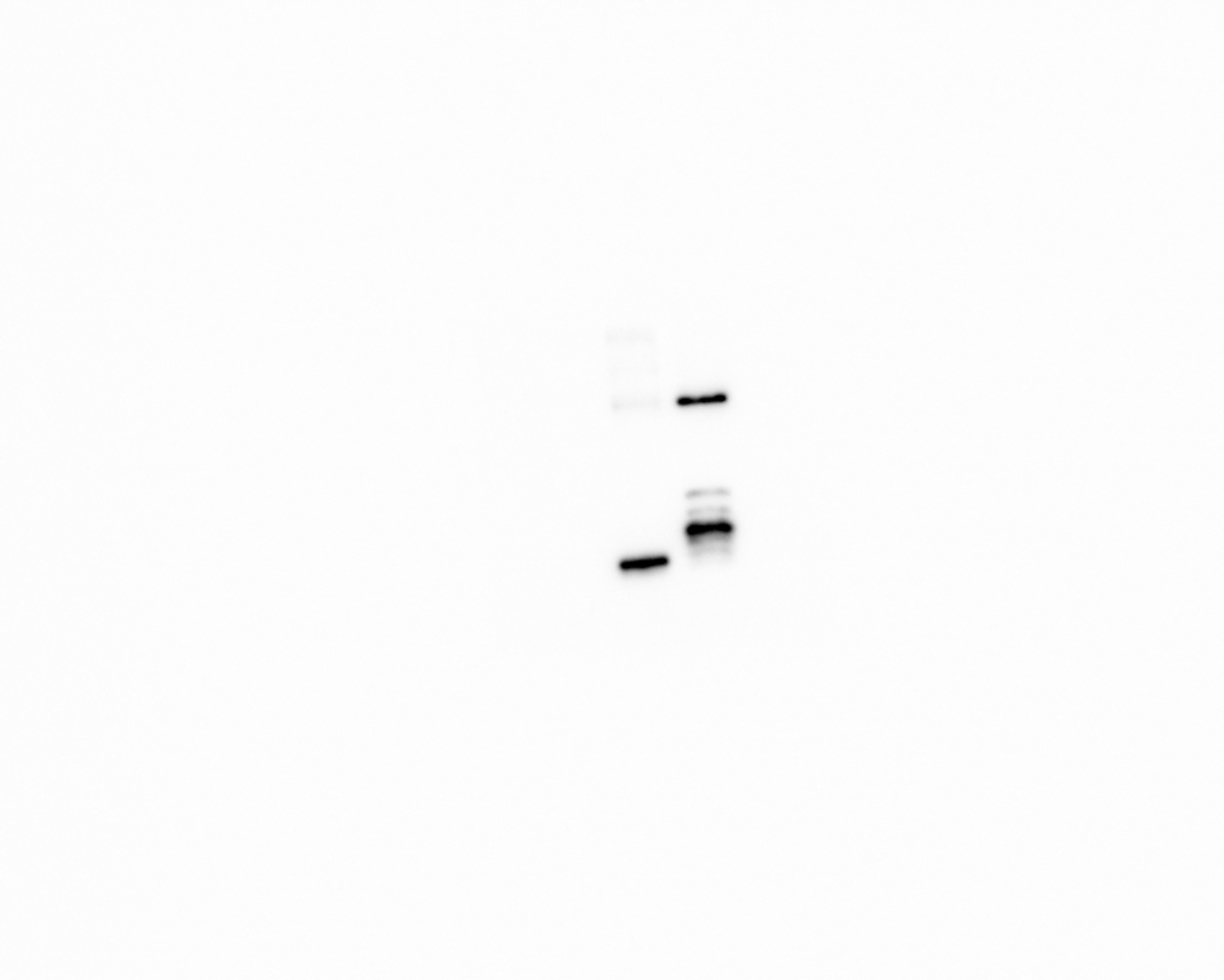

Supplement: Figure 4—source data 1. — Including uncropped Western blot images and raw statistics. [file elife-76436-fig4-data1.zip › Figure 4-Source Data 1/Figure 4B full raw unedited/IB-GST.tif]

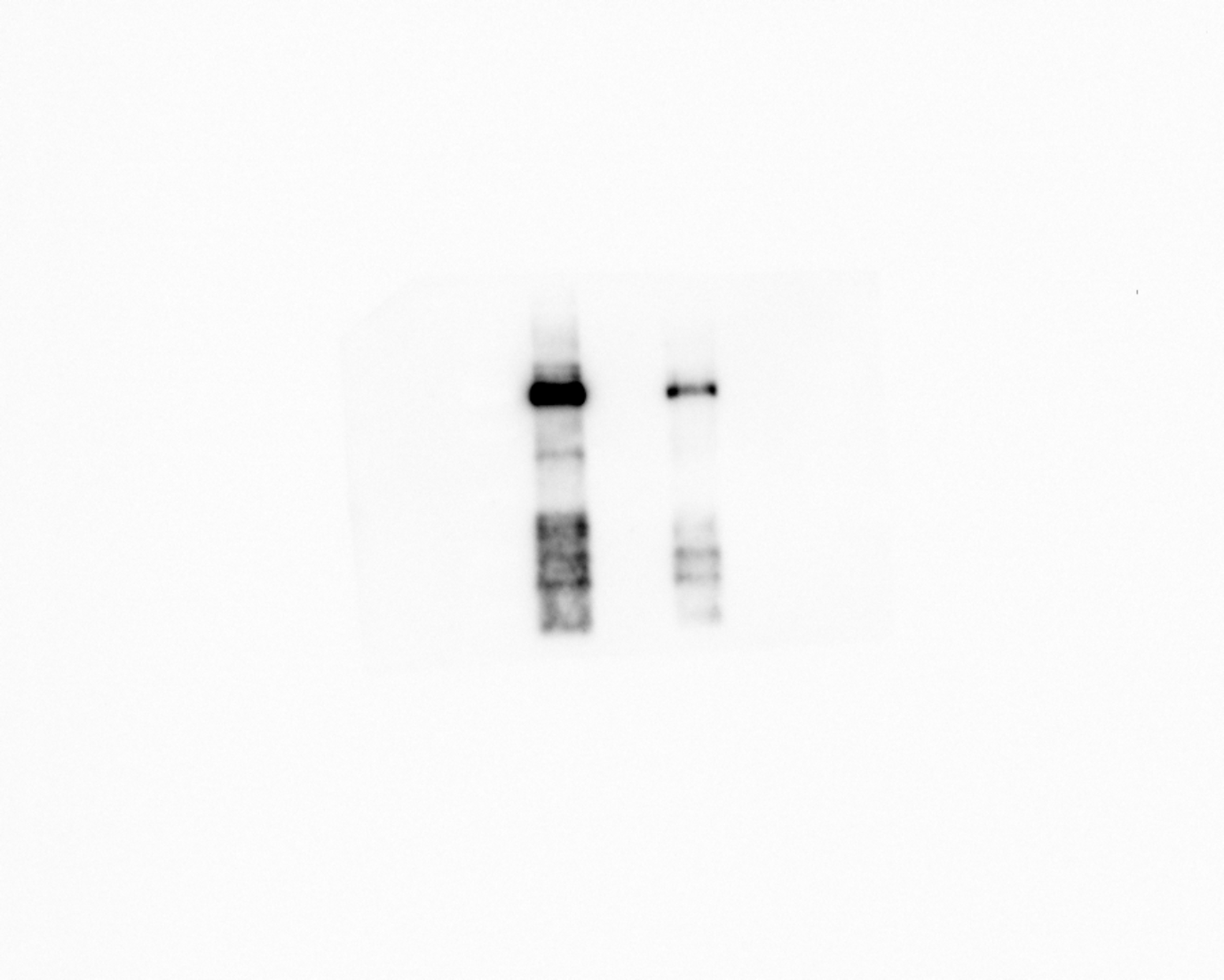

Supplement: Figure 4—source data 1. — Including uncropped Western blot images and raw statistics. [file elife-76436-fig4-data1.zip › Figure 4-Source Data 1/Figure 4B full raw unedited/IB-MYC.tif]

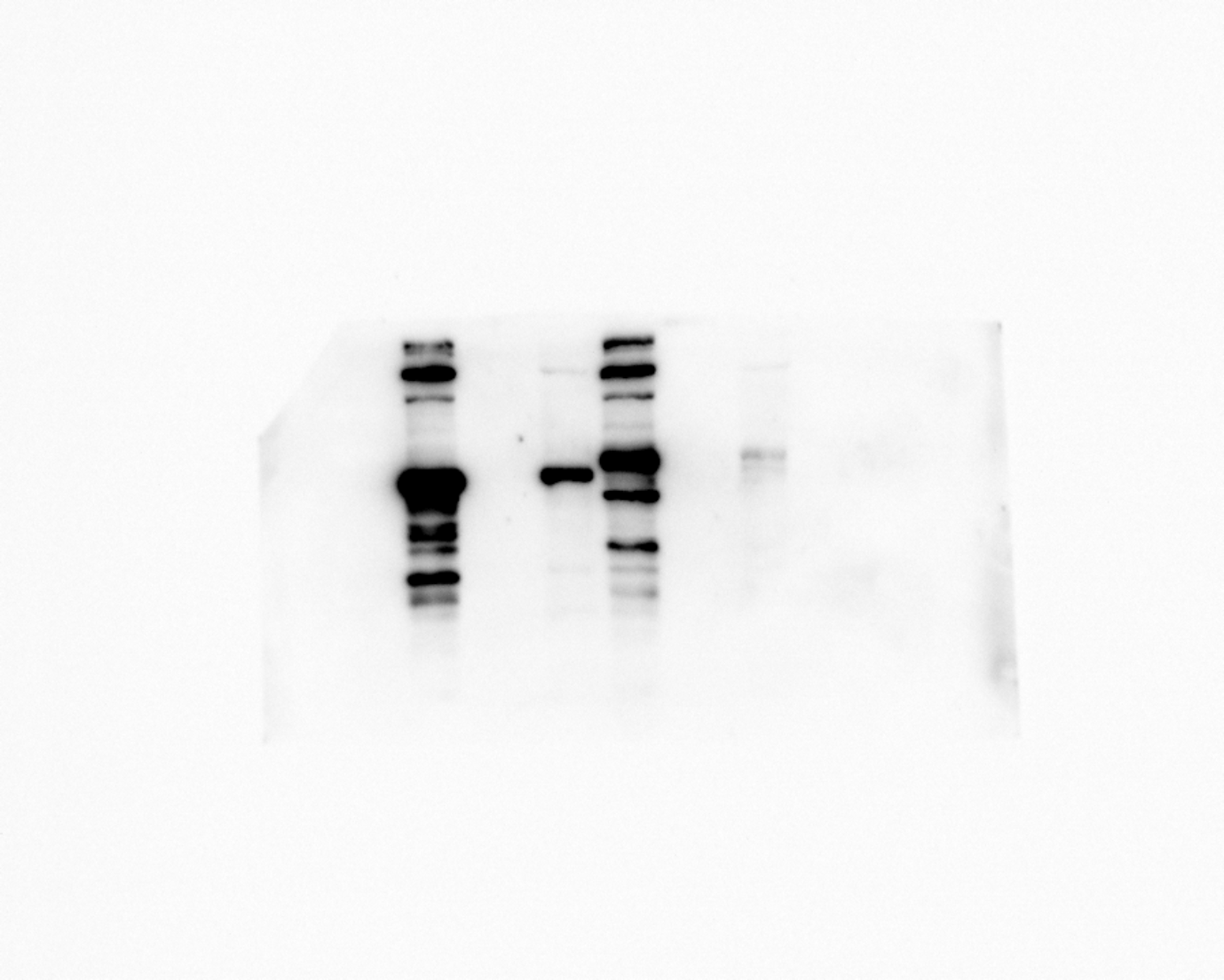

Supplement: Figure 4—source data 1. — Including uncropped Western blot images and raw statistics. [file elife-76436-fig4-data1.zip › Figure 4-Source Data 1/Figure 4C full raw unedited/IB-MYC.tif]

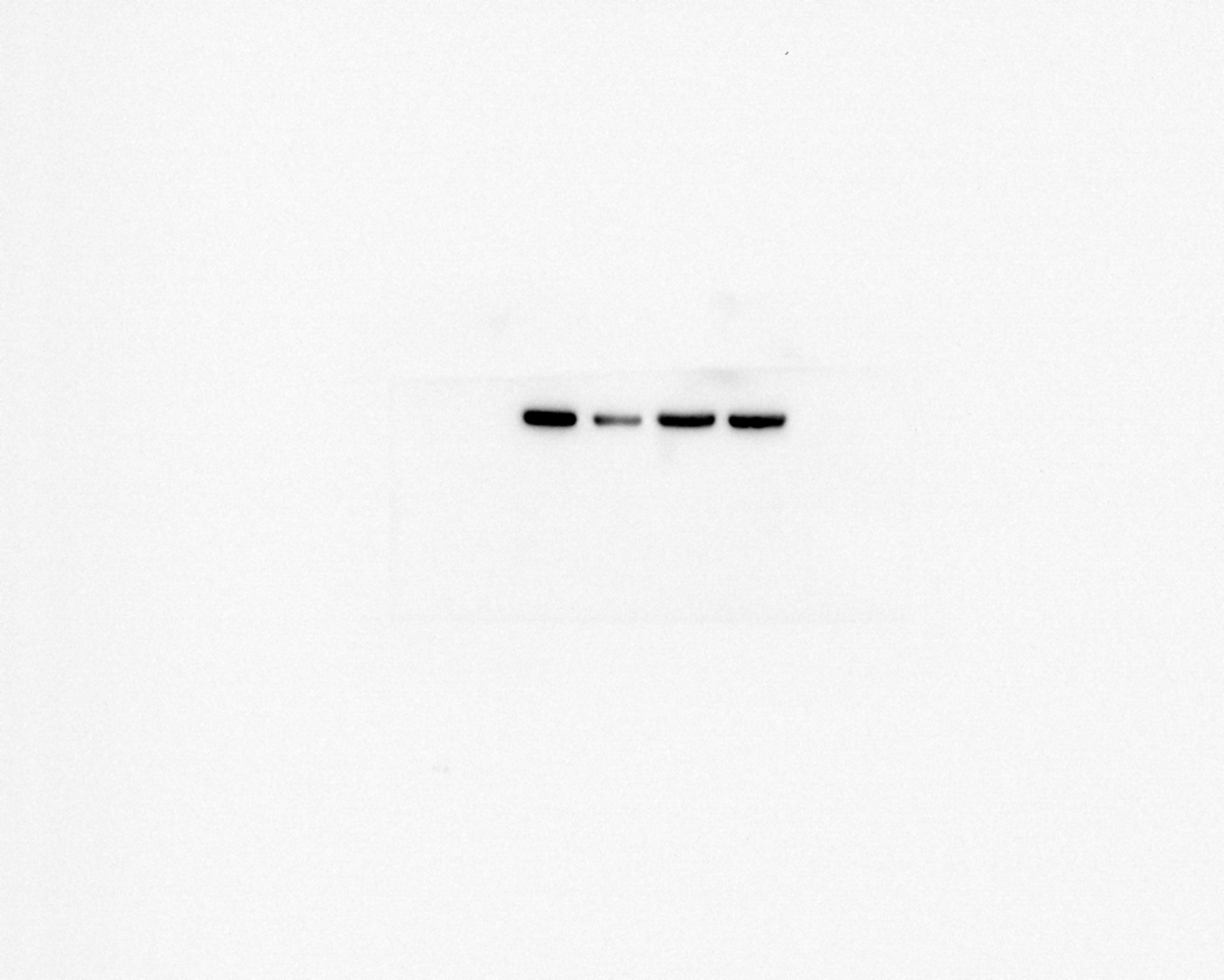

Supplement: Figure 4—source data 1. — Including uncropped Western blot images and raw statistics. [file elife-76436-fig4-data1.zip › Figure 4-Source Data 1/Figure 4E full raw unedited/IB-Actin.tif]

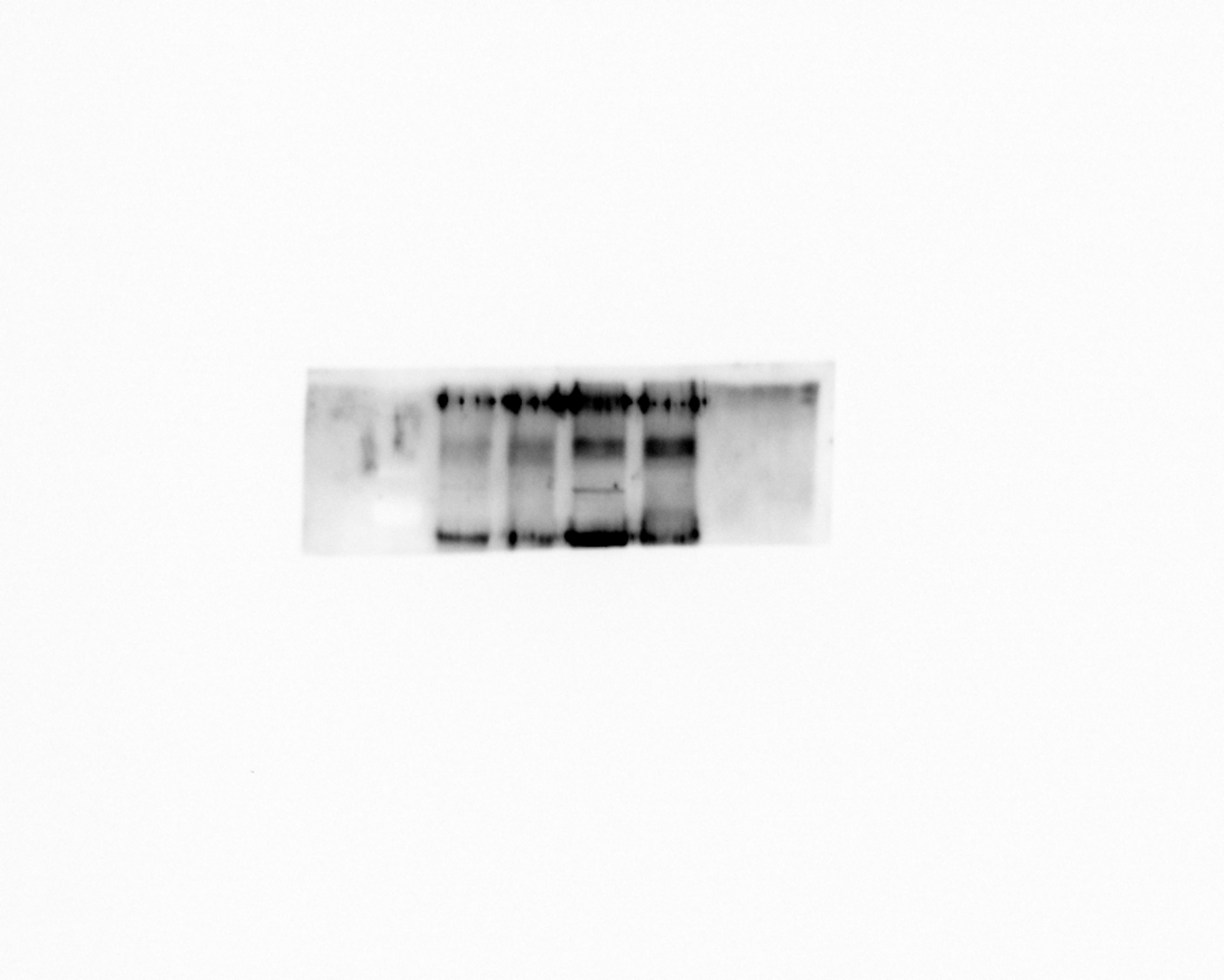

Supplement: Figure 4—source data 1. — Including uncropped Western blot images and raw statistics. [file elife-76436-fig4-data1.zip › Figure 4-Source Data 1/Figure 4E full raw unedited/IB-CED-1.tif]

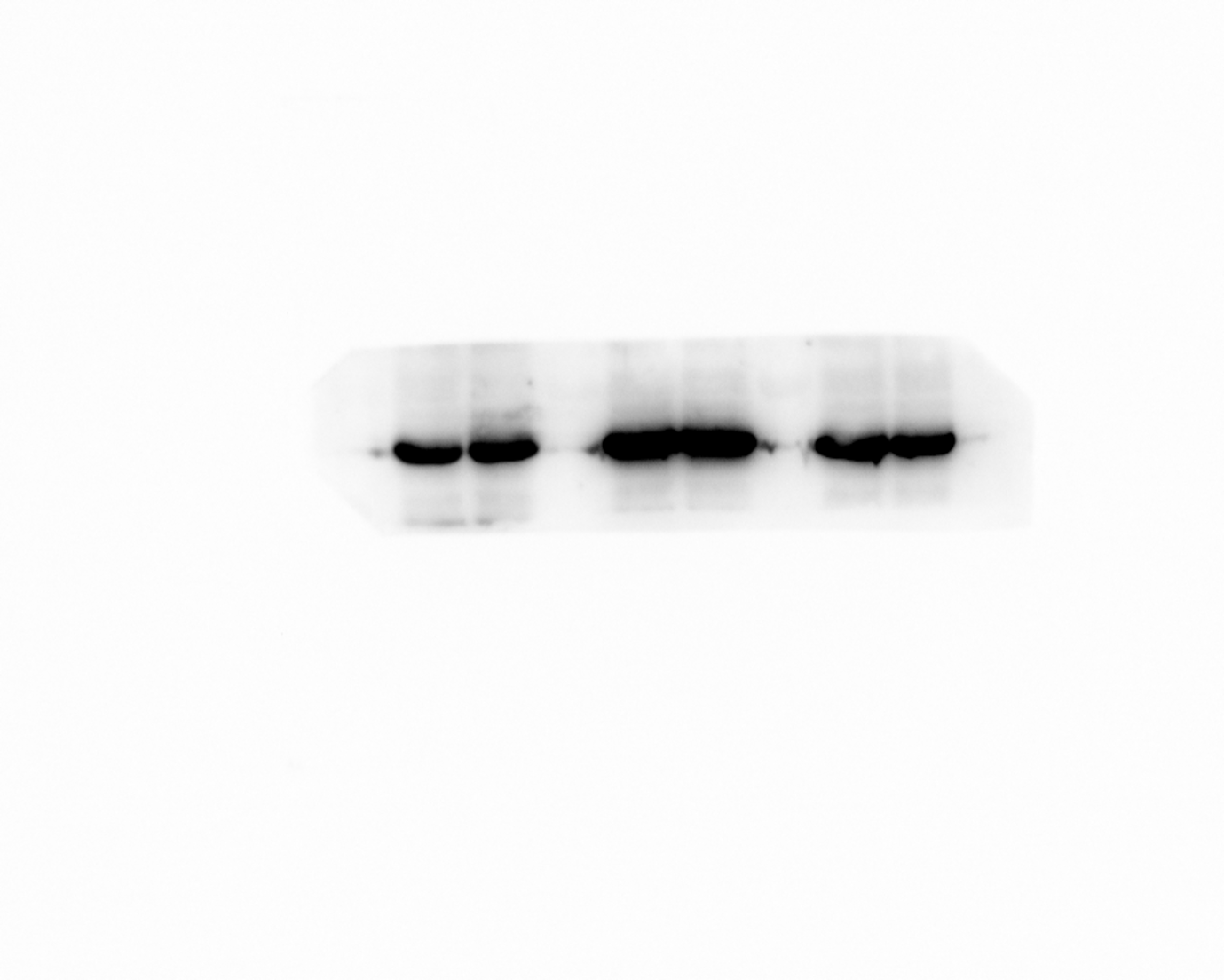

Supplement: Figure 4—source data 1. — Including uncropped Western blot images and raw statistics. [file elife-76436-fig4-data1.zip › Figure 4-Source Data 1/Figure 4F full raw unedited/Input-IB-Actin.tif]

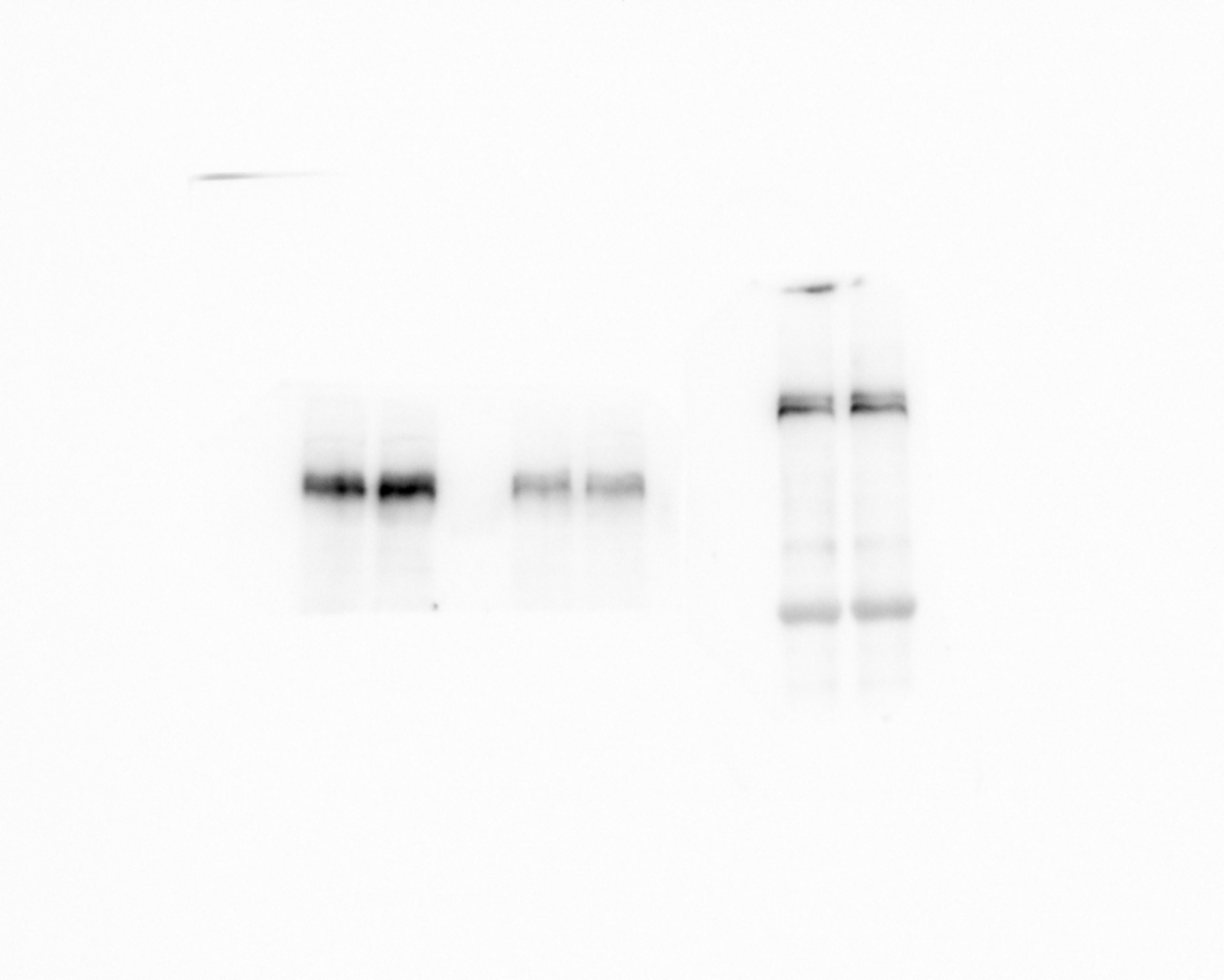

Supplement: Figure 4—source data 1. — Including uncropped Western blot images and raw statistics. [file elife-76436-fig4-data1.zip › Figure 4-Source Data 1/Figure 4F full raw unedited/Input-IB-FLAG.tif]

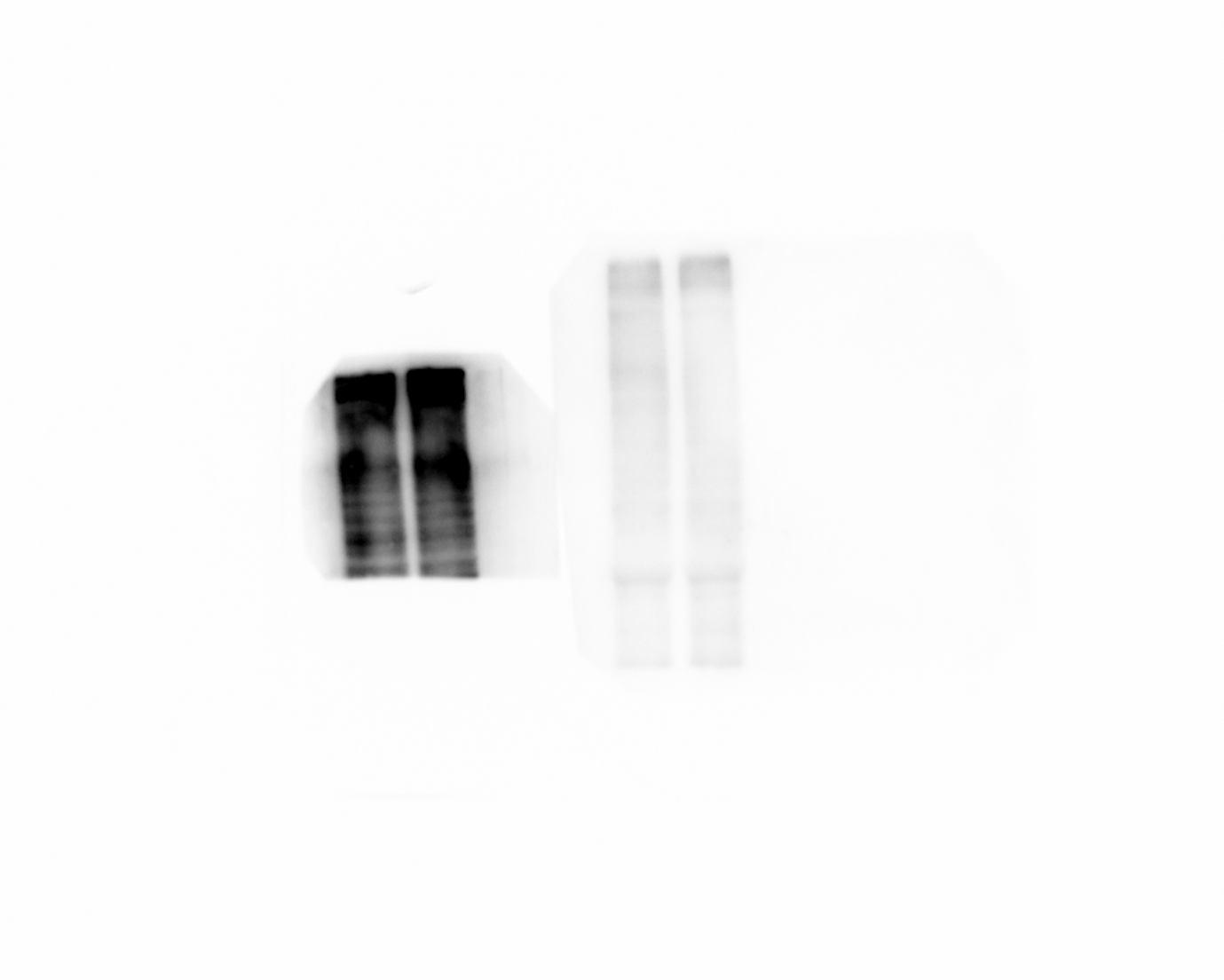

Supplement: Figure 4—source data 1. — Including uncropped Western blot images and raw statistics. [file elife-76436-fig4-data1.zip › Figure 4-Source Data 1/Figure 4F full raw unedited/Input-IB-HA.tif]

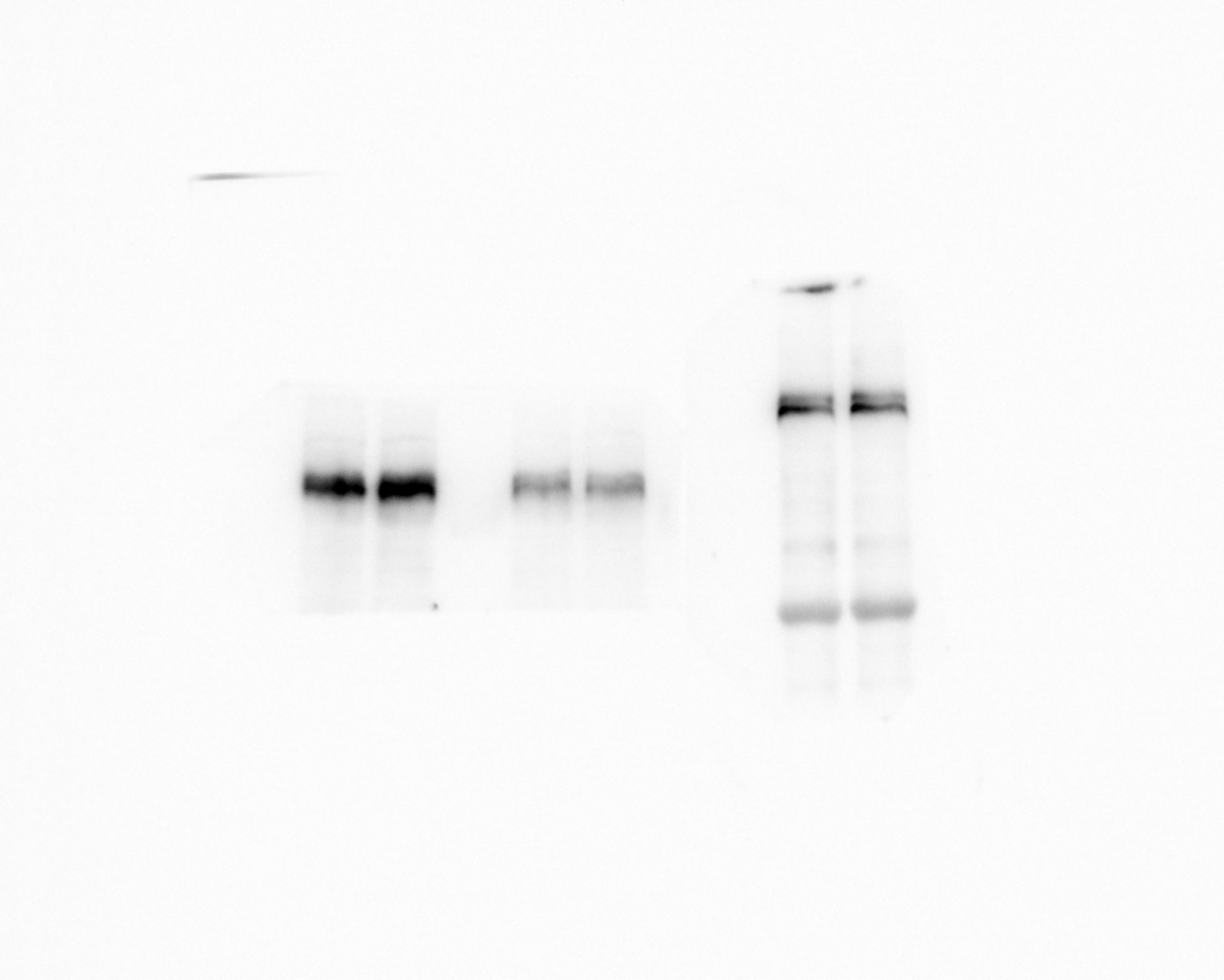

Supplement: Figure 4—source data 1. — Including uncropped Western blot images and raw statistics. [file elife-76436-fig4-data1.zip › Figure 4-Source Data 1/Figure 4F full raw unedited/IP-IB-FLAG.tif]

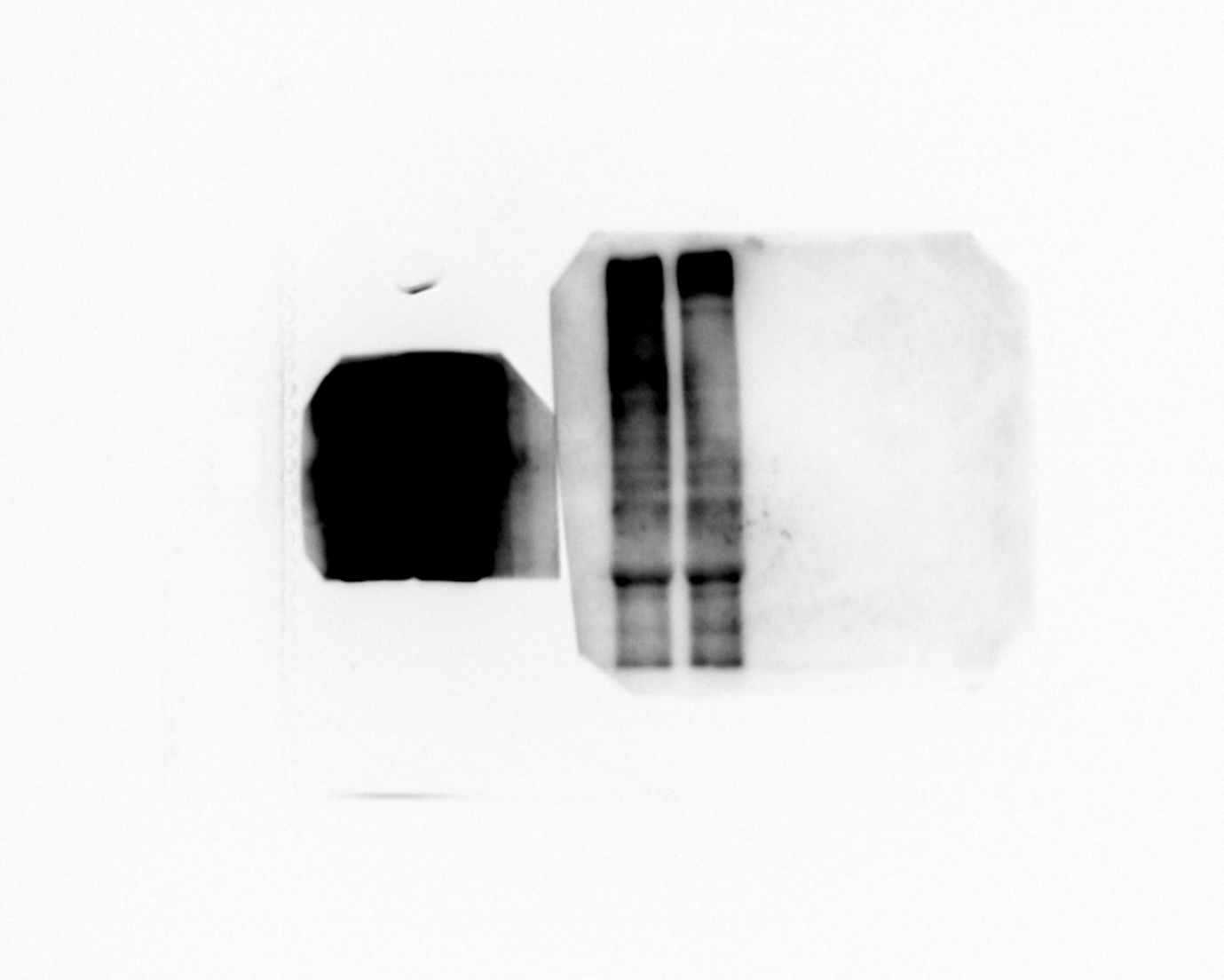

Supplement: Figure 4—source data 1. — Including uncropped Western blot images and raw statistics. [file elife-76436-fig4-data1.zip › Figure 4-Source Data 1/Figure 4F full raw unedited/IP-IB-HA.tif]

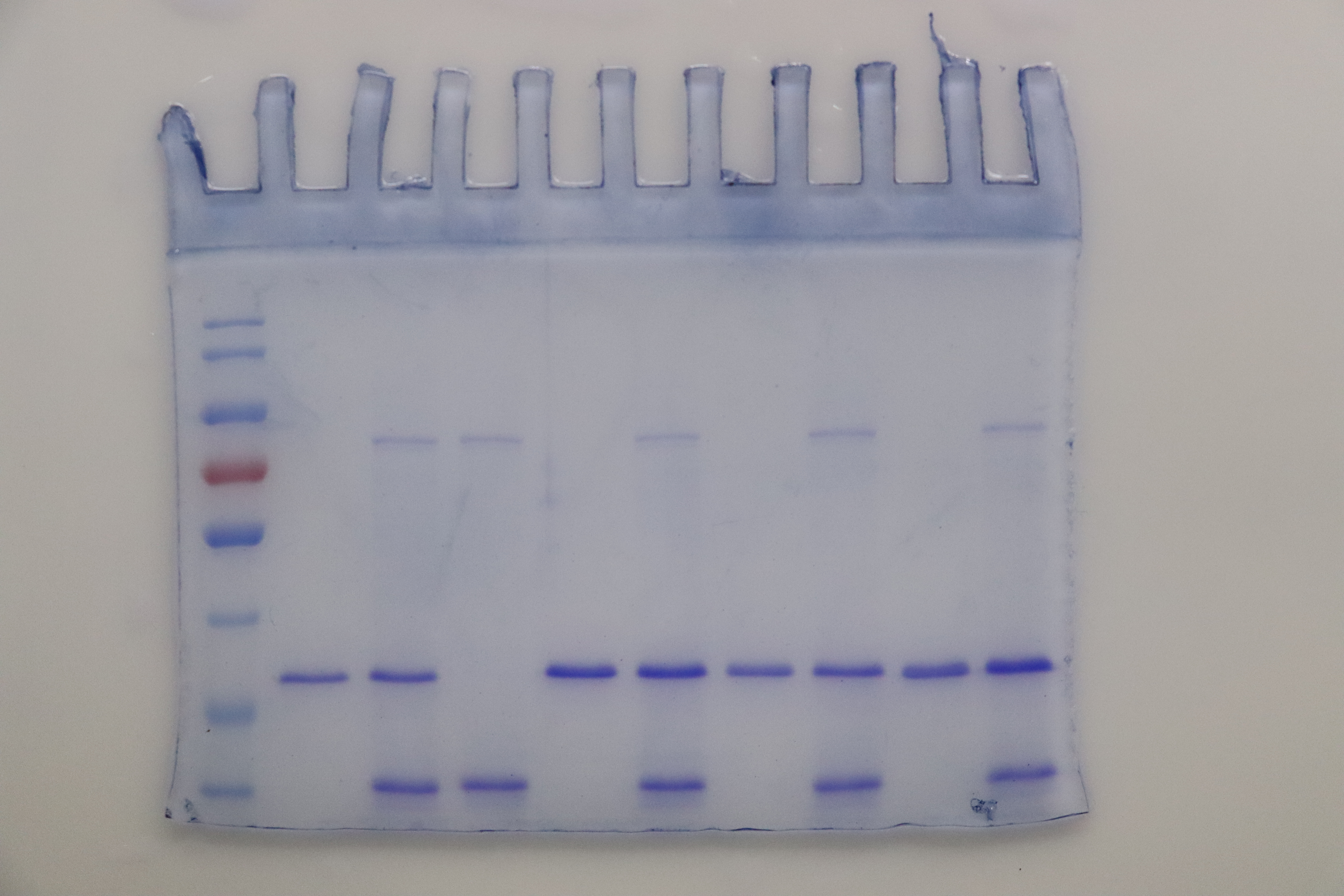

Supplement: Figure 4—source data 1. — Including uncropped Western blot images and raw statistics. [file elife-76436-fig4-data1.zip › Figure 4-Source Data 1/Figure 4I full raw unedited/CCB Staining.JPG]

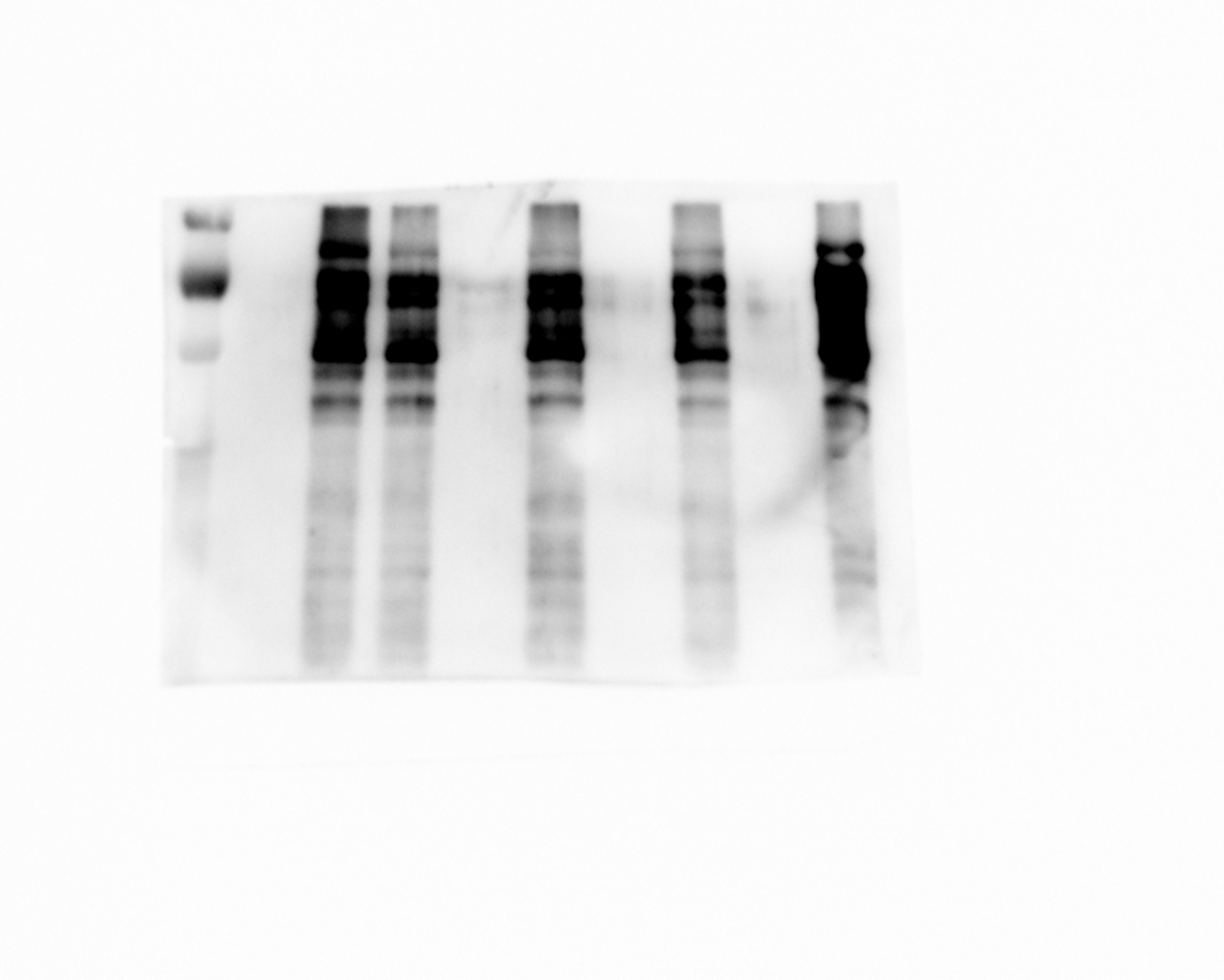

Supplement: Figure 4—source data 1. — Including uncropped Western blot images and raw statistics. [file elife-76436-fig4-data1.zip › Figure 4-Source Data 1/Figure 4I full raw unedited/IB-Phosphotyrosine Antibodies.tif]

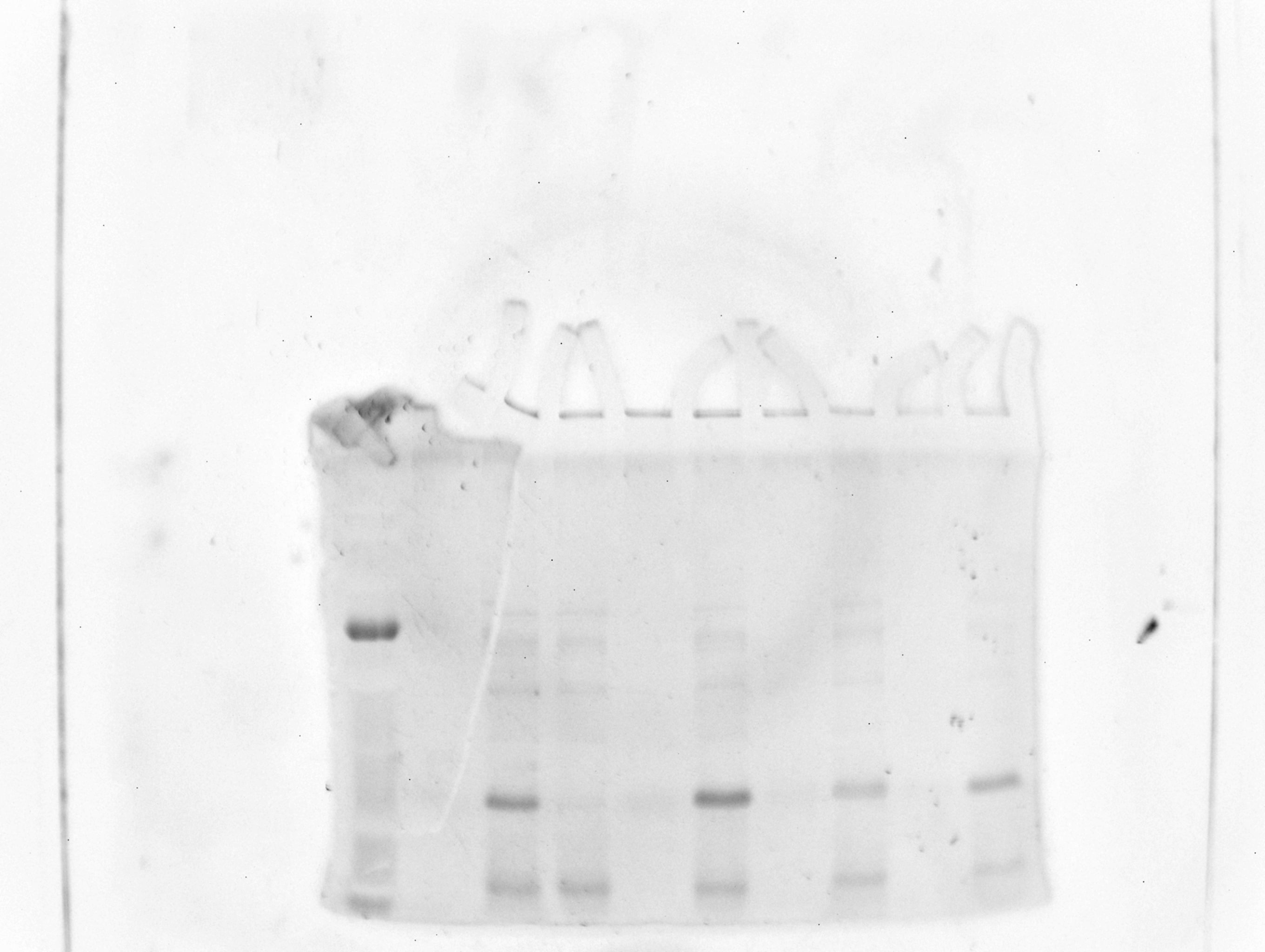

Supplement: Figure 4—source data 1. — Including uncropped Western blot images and raw statistics. [file elife-76436-fig4-data1.zip › Figure 4-Source Data 1/Figure 4I full raw unedited/Pro-Q Phosphorylation Staining.tif]

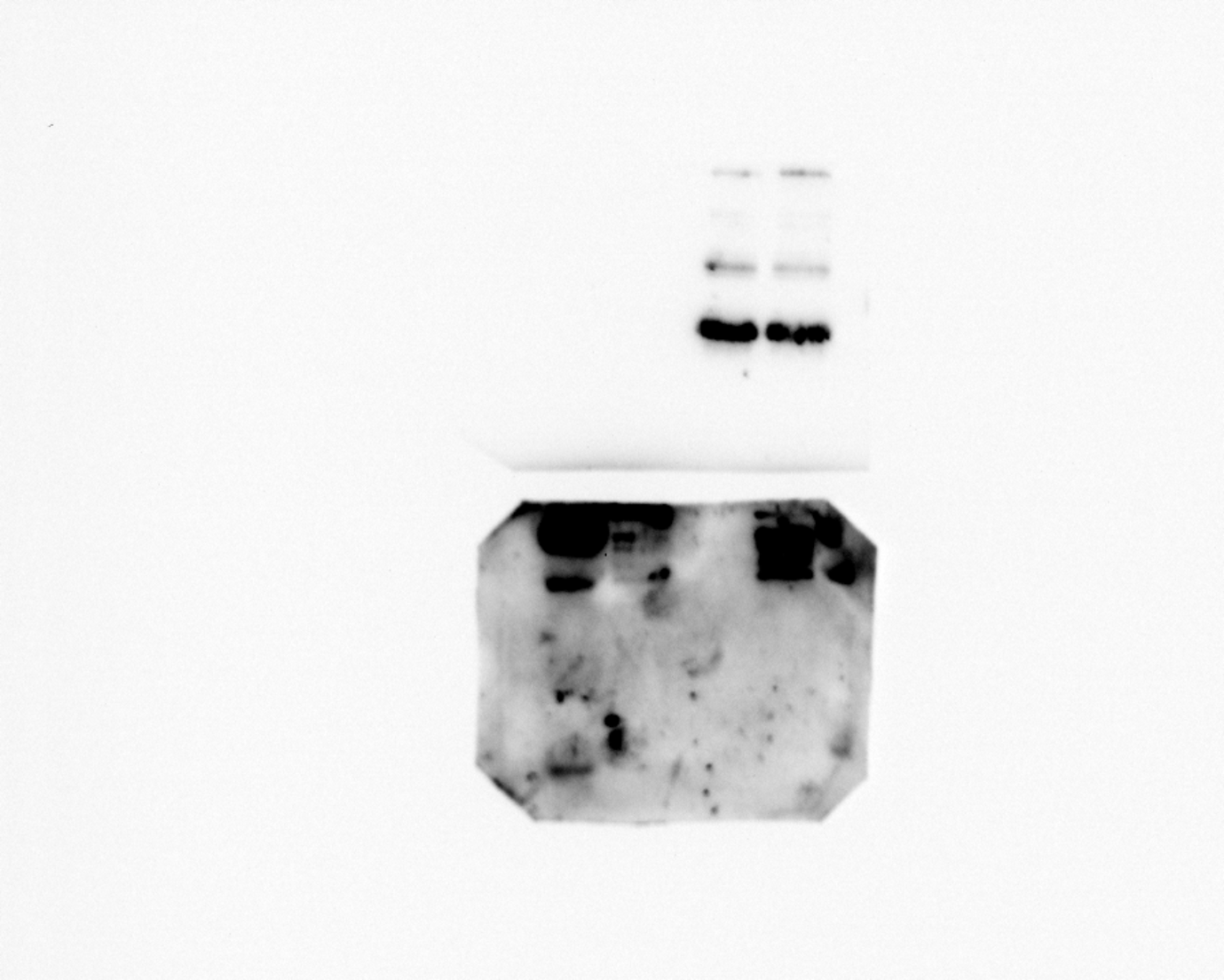

Supplement: Figure 4—figure supplement 1—source data 1. — Including uncropped Western blot images and raw statistics. [file elife-76436-fig4-figsupp1-data1.zip › Figure 4-figure supplement 1-Source Data 1/Figure 4-figure supplement 1B full raw unedited/IB-FLAG.tif]

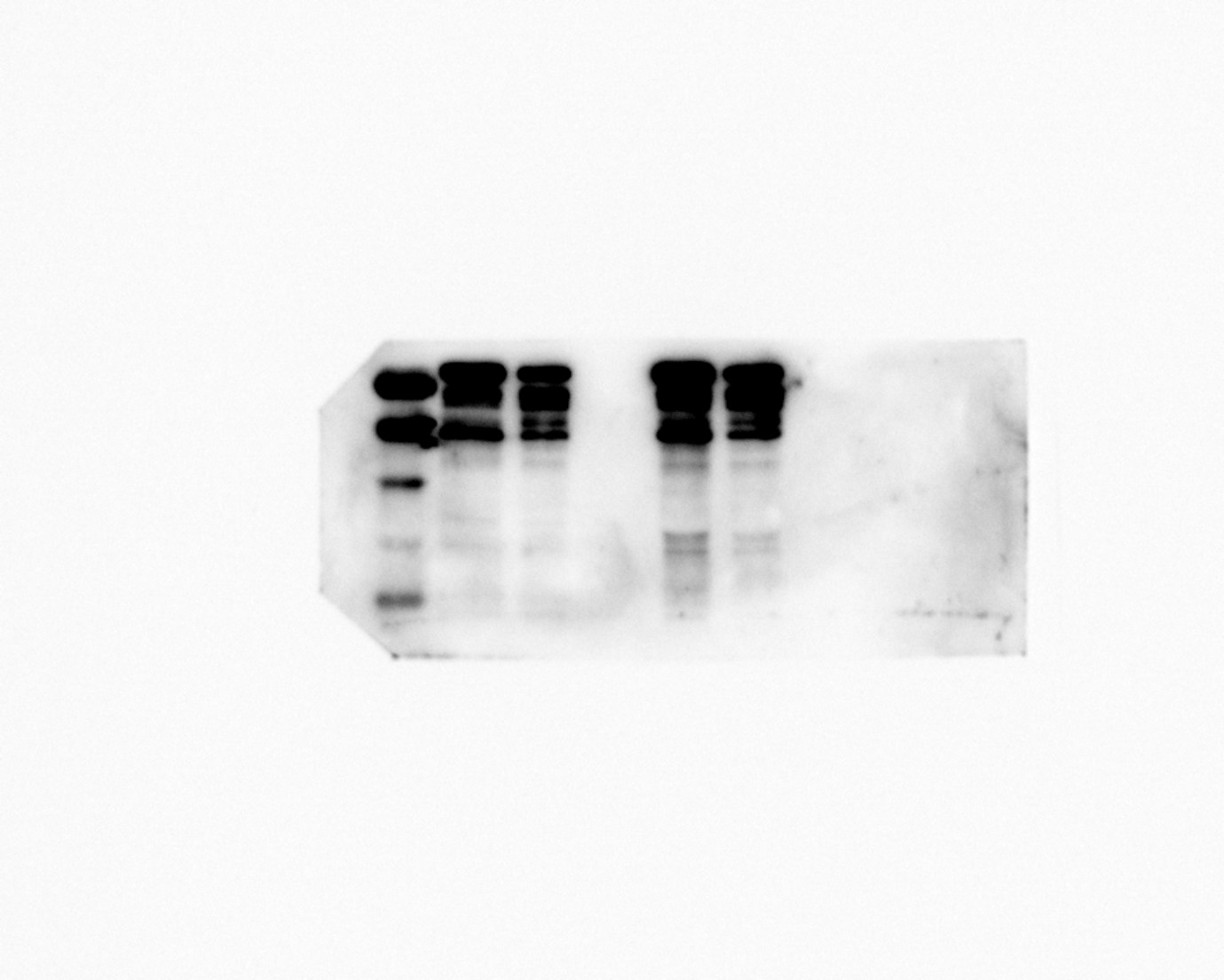

Supplement: Figure 4—figure supplement 1—source data 1. — Including uncropped Western blot images and raw statistics. [file elife-76436-fig4-figsupp1-data1.zip › Figure 4-figure supplement 1-Source Data 1/Figure 4-figure supplement 1B full raw unedited/IB-P-Tyr-100.tif]

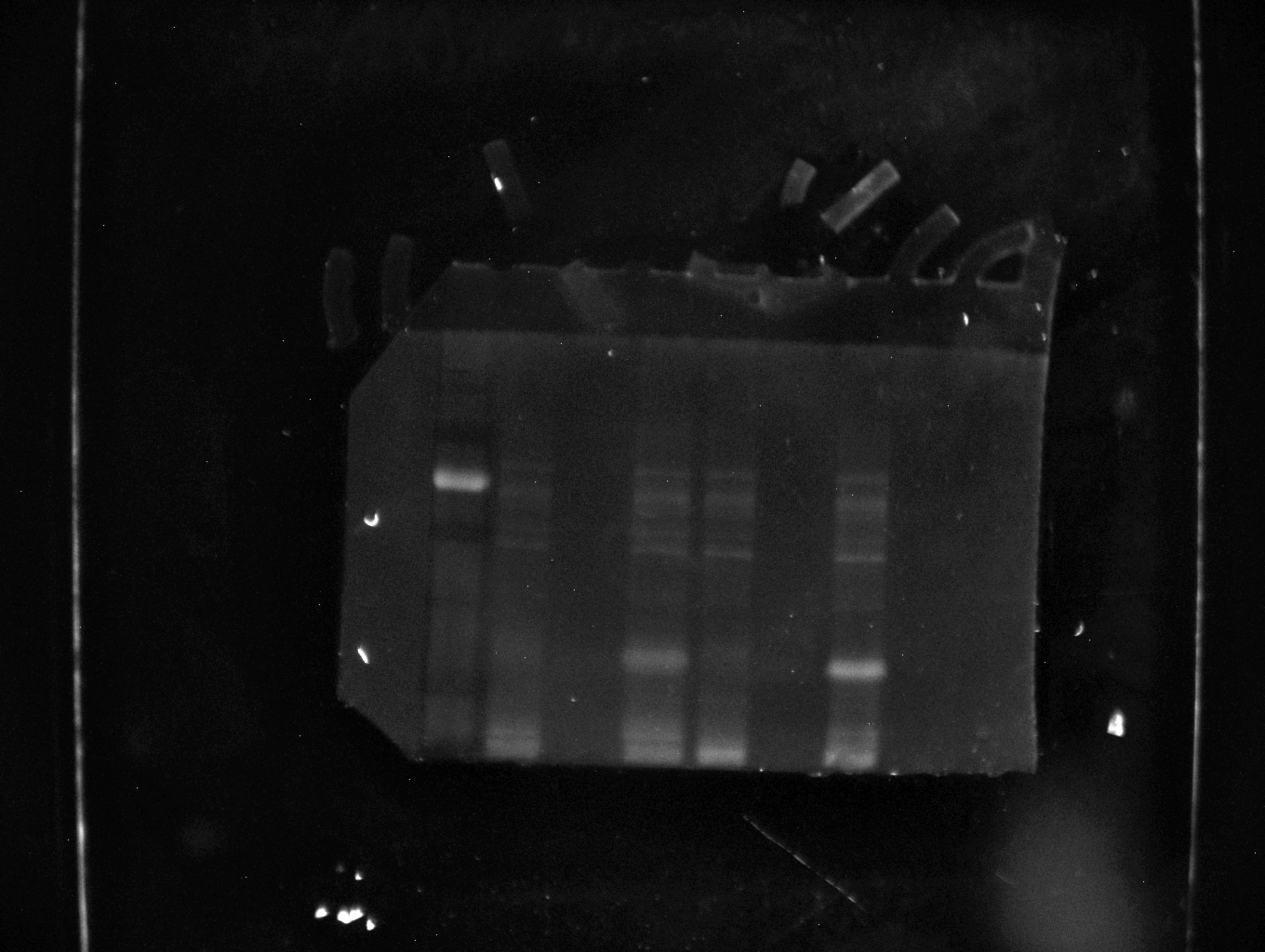

Supplement: Figure 4—figure supplement 1—source data 1. — Including uncropped Western blot images and raw statistics. [file elife-76436-fig4-figsupp1-data1.zip › Figure 4-figure supplement 1-Source Data 1/Figure 4-figure supplement 1B full raw unedited/Phosphorylation Staining.tif]

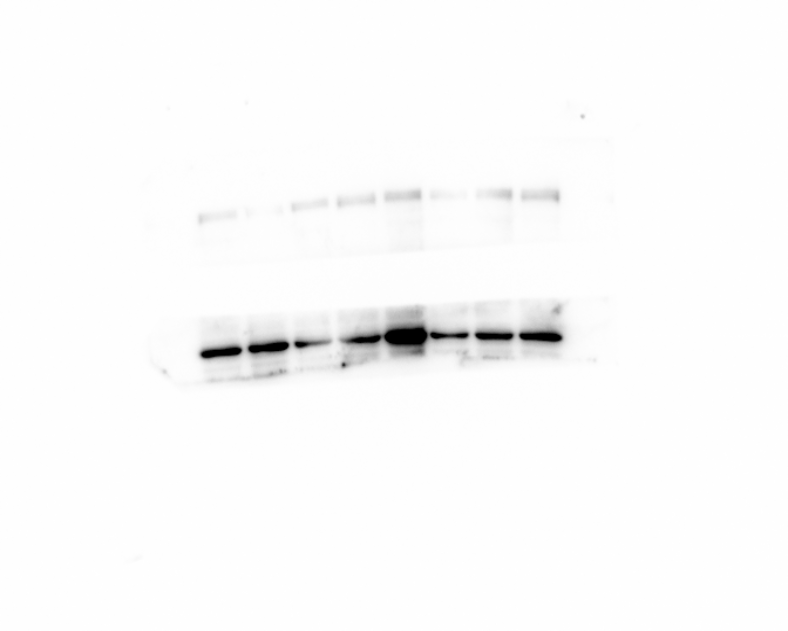

Supplement: Figure 4—figure supplement 1—source data 1. — Including uncropped Western blot images and raw statistics. [file elife-76436-fig4-figsupp1-data1.zip › Figure 4-figure supplement 1-Source Data 1/Figure 4-figure supplement 1D full raw unedited/IB-Actin.tif]

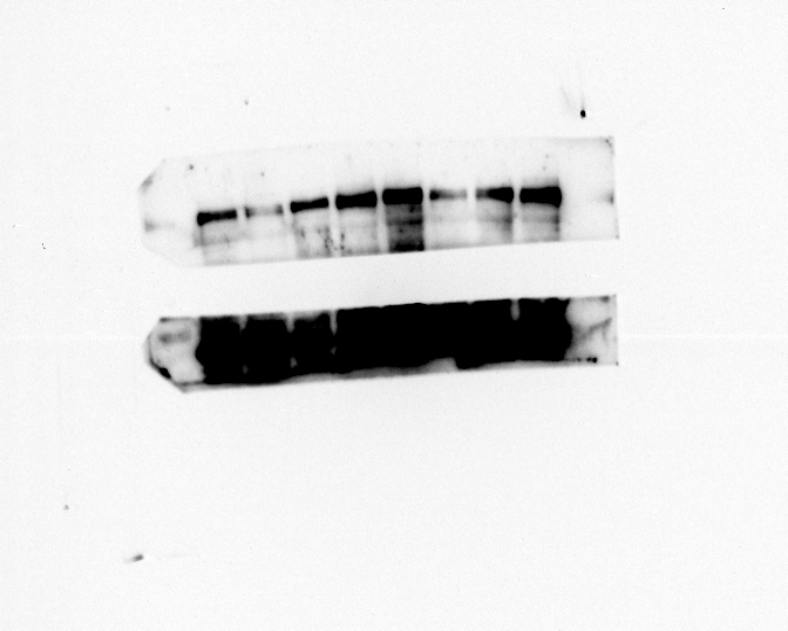

Supplement: Figure 4—figure supplement 1—source data 1. — Including uncropped Western blot images and raw statistics. [file elife-76436-fig4-figsupp1-data1.zip › Figure 4-figure supplement 1-Source Data 1/Figure 4-figure supplement 1D full raw unedited/IB-CED-1.tif]

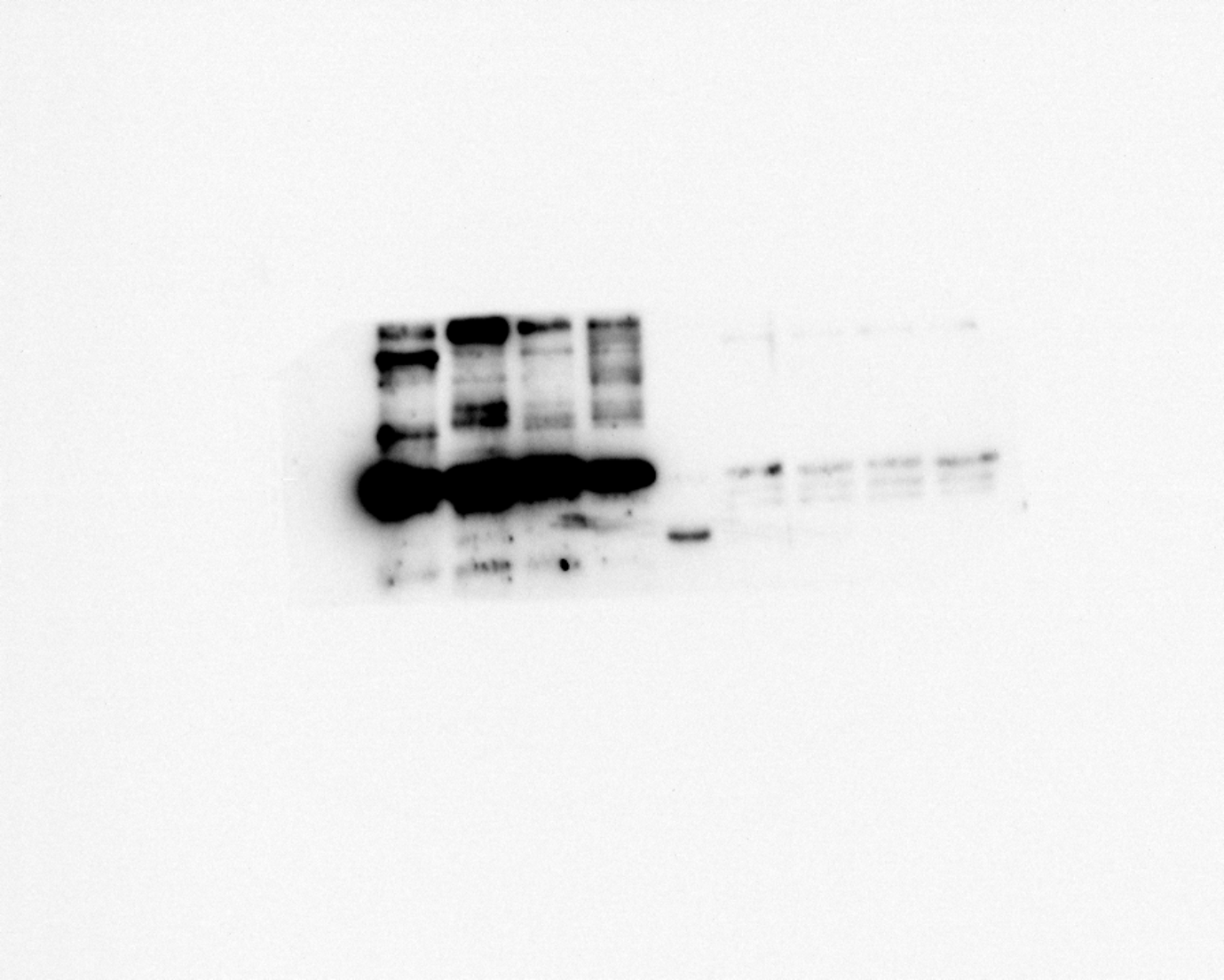

Supplement: Figure 4—figure supplement 1—source data 1. — Including uncropped Western blot images and raw statistics. [file elife-76436-fig4-figsupp1-data1.zip › Figure 4-figure supplement 1-Source Data 1/Figure 4-figure supplement 1I full raw unedited/GST Pull down-IB-FLAG.tif]

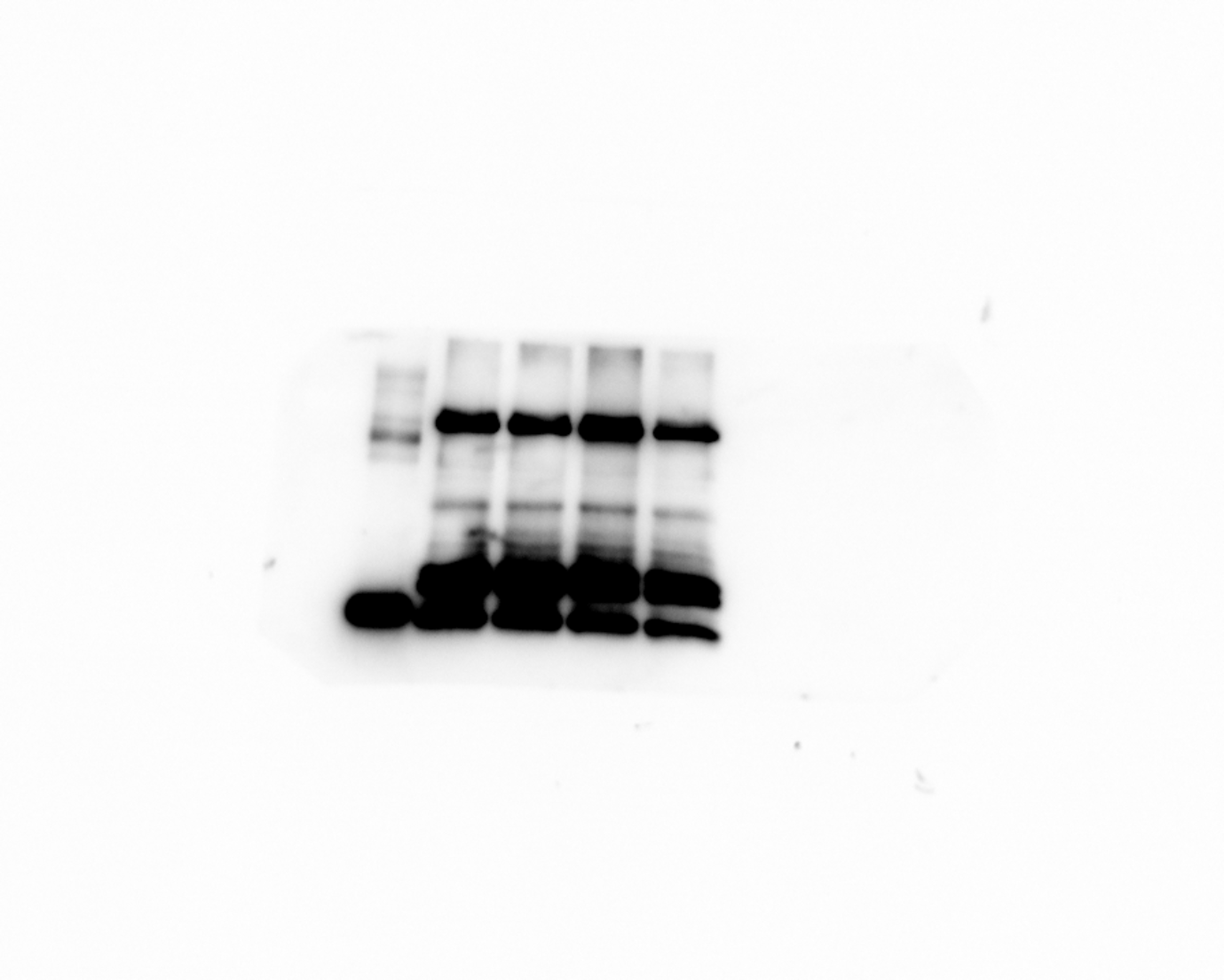

Supplement: Figure 4—figure supplement 1—source data 1. — Including uncropped Western blot images and raw statistics. [file elife-76436-fig4-figsupp1-data1.zip › Figure 4-figure supplement 1-Source Data 1/Figure 4-figure supplement 1I full raw unedited/GST Pull down-IB-GST.tif]

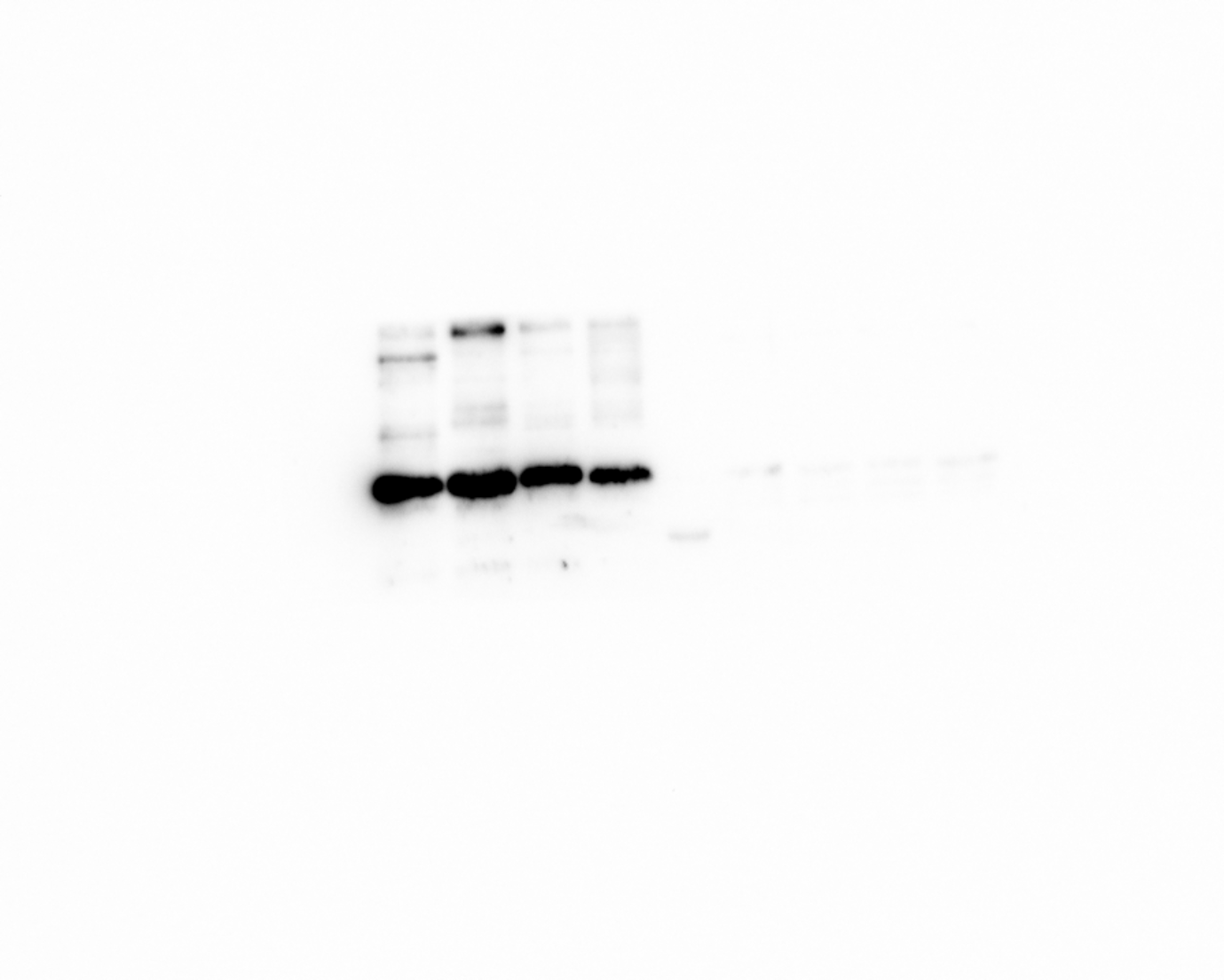

Supplement: Figure 4—figure supplement 1—source data 1. — Including uncropped Western blot images and raw statistics. [file elife-76436-fig4-figsupp1-data1.zip › Figure 4-figure supplement 1-Source Data 1/Figure 4-figure supplement 1I full raw unedited/Input-IB-FLAG.tif]

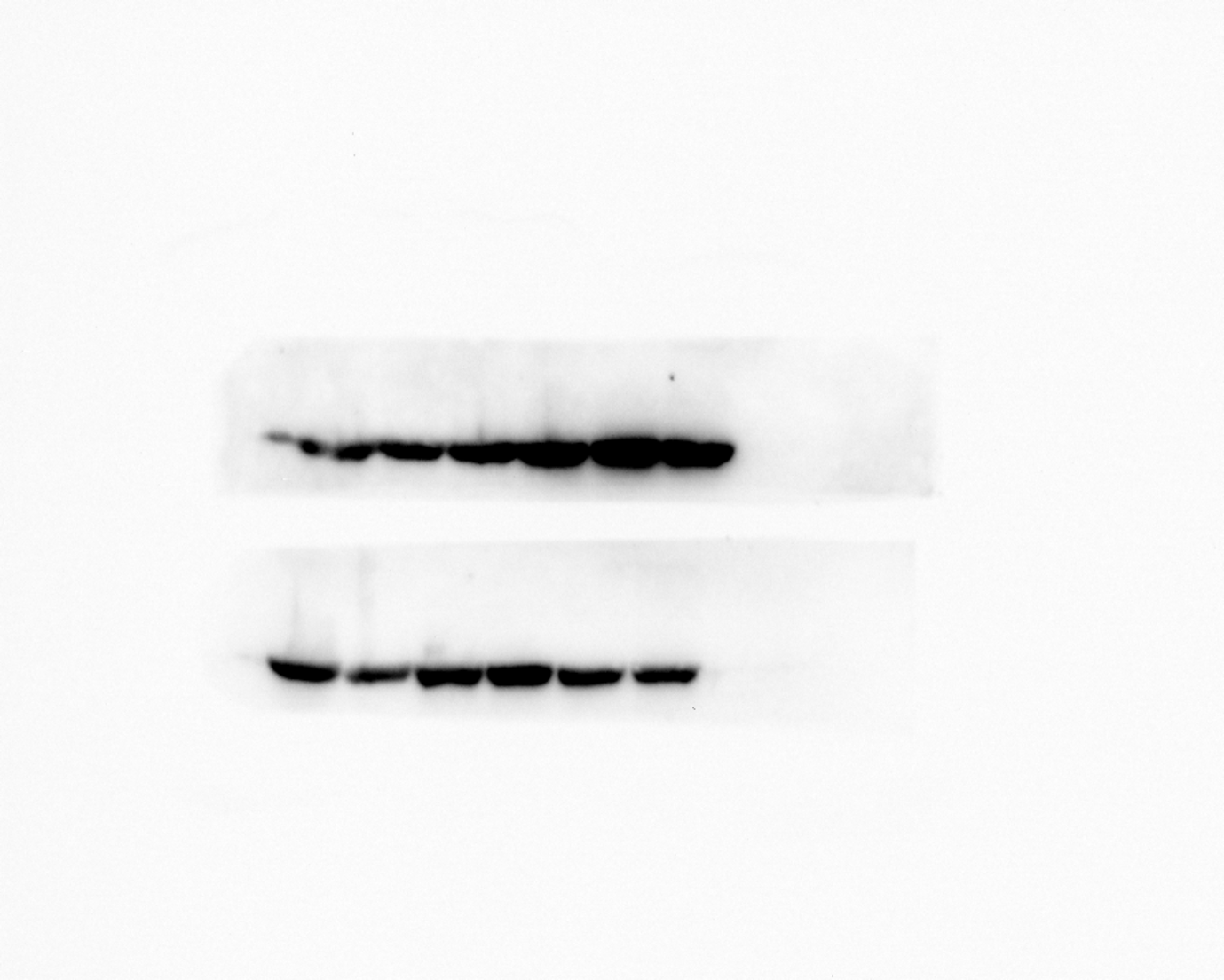

Supplement: Figure 4—figure supplement 1—source data 1. — Including uncropped Western blot images and raw statistics. [file elife-76436-fig4-figsupp1-data1.zip › Figure 4-figure supplement 1-Source Data 1/Figure 4-figure supplement 1J full raw unedited/ced-1-flag-IB-Actin.tif]

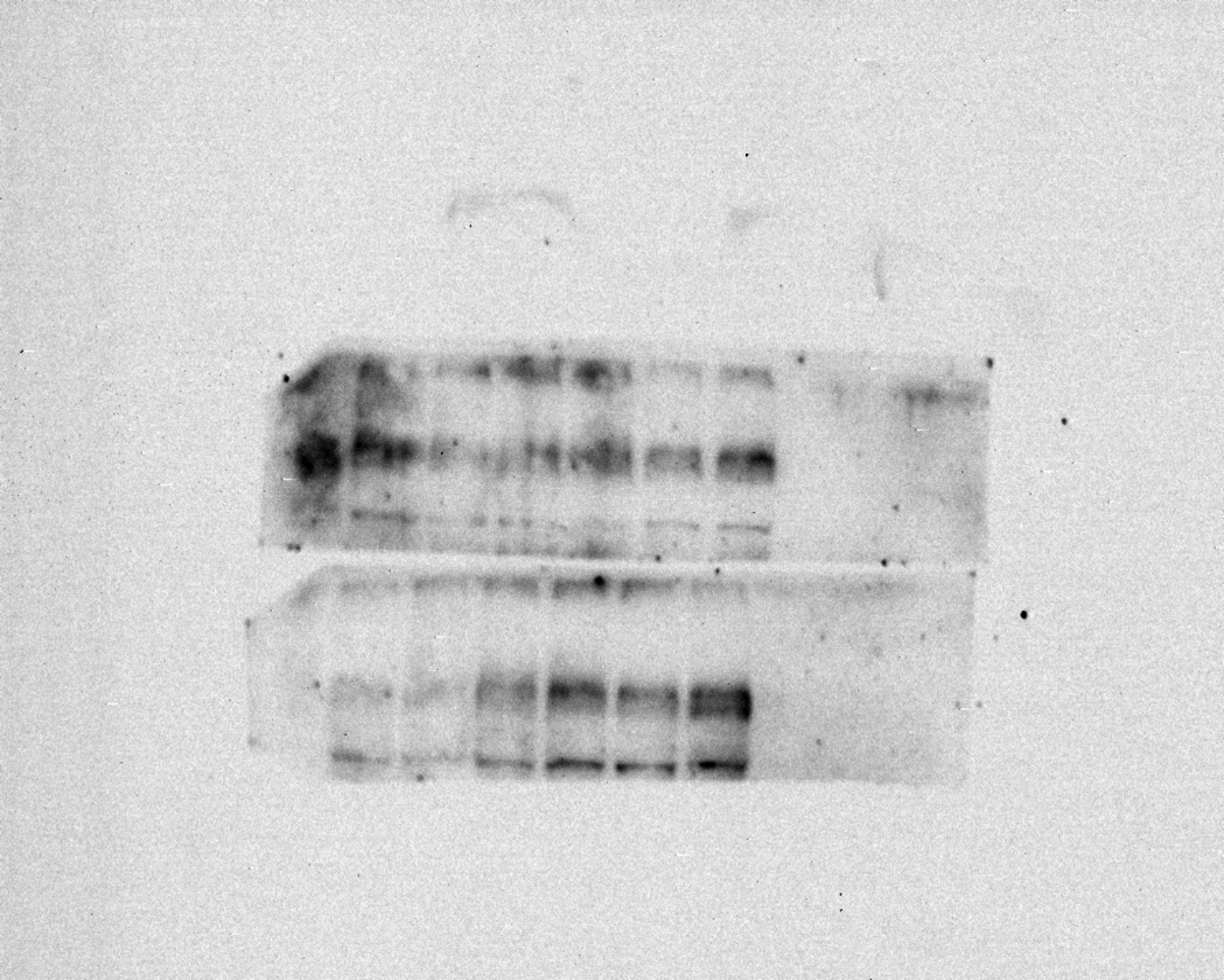

Supplement: Figure 4—figure supplement 1—source data 1. — Including uncropped Western blot images and raw statistics. [file elife-76436-fig4-figsupp1-data1.zip › Figure 4-figure supplement 1-Source Data 1/Figure 4-figure supplement 1J full raw unedited/ced-1-flag-IB-CED-1.tif]

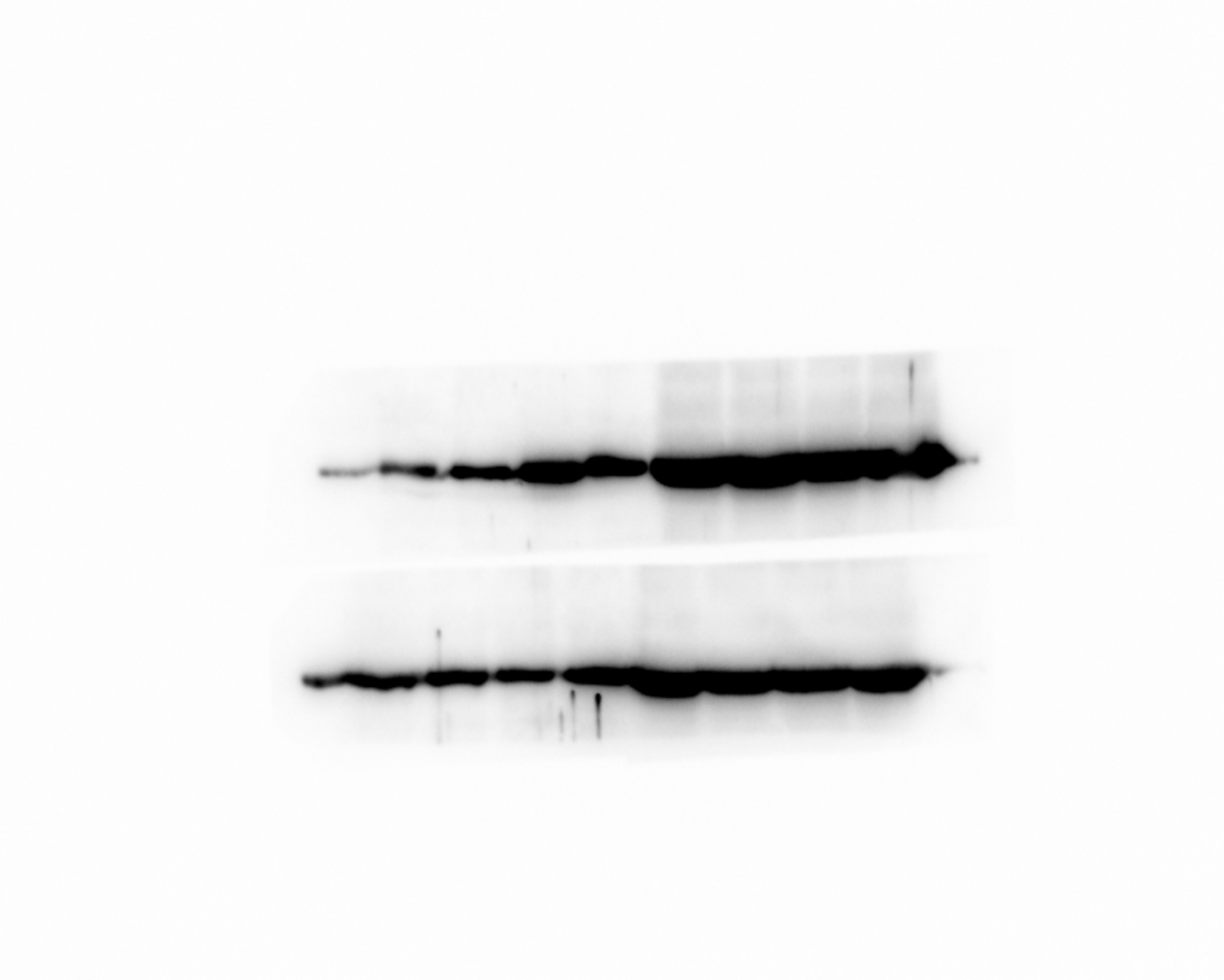

Supplement: Figure 4—figure supplement 1—source data 1. — Including uncropped Western blot images and raw statistics. [file elife-76436-fig4-figsupp1-data1.zip › Figure 4-figure supplement 1-Source Data 1/Figure 4-figure supplement 1J full raw unedited/ced-1-N962A-IB-Actin.tif]

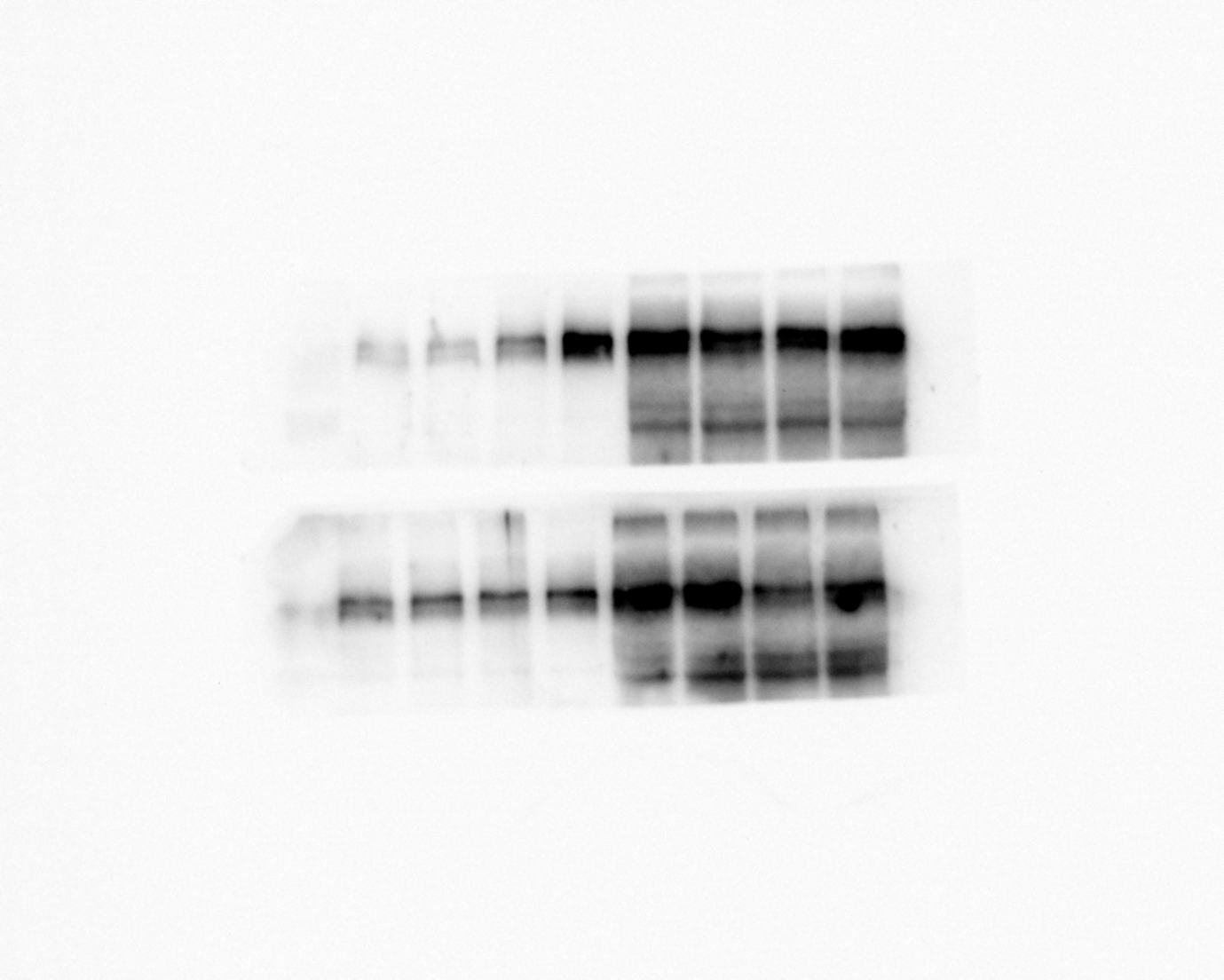

Supplement: Figure 4—figure supplement 1—source data 1. — Including uncropped Western blot images and raw statistics. [file elife-76436-fig4-figsupp1-data1.zip › Figure 4-figure supplement 1-Source Data 1/Figure 4-figure supplement 1J full raw unedited/ced-1-N962A-IB-CED-1.tif]

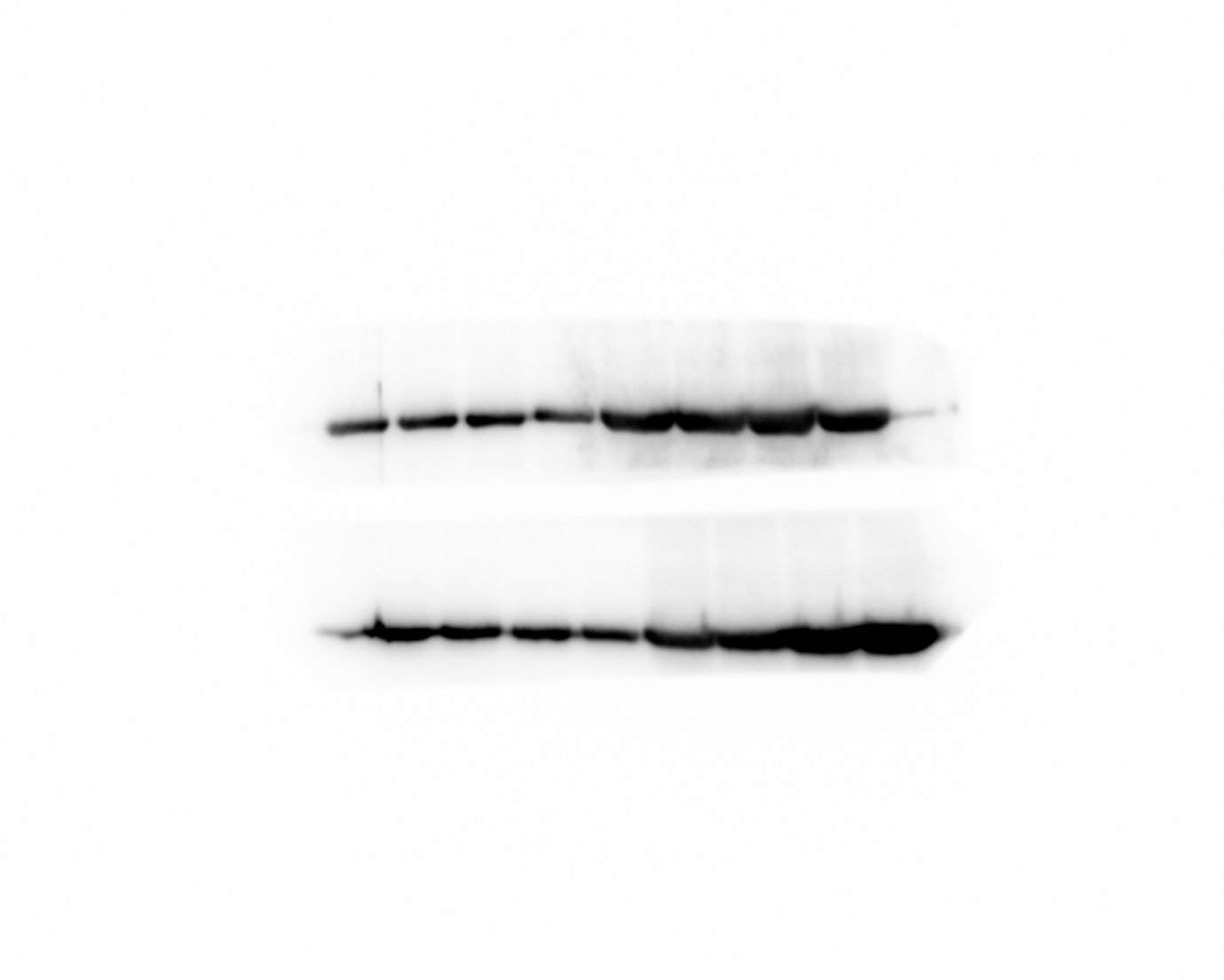

Supplement: Figure 4—figure supplement 1—source data 1. — Including uncropped Western blot images and raw statistics. [file elife-76436-fig4-figsupp1-data1.zip › Figure 4-figure supplement 1-Source Data 1/Figure 4-figure supplement 1J full raw unedited/ced-1-Y1019F-Actin.tif]

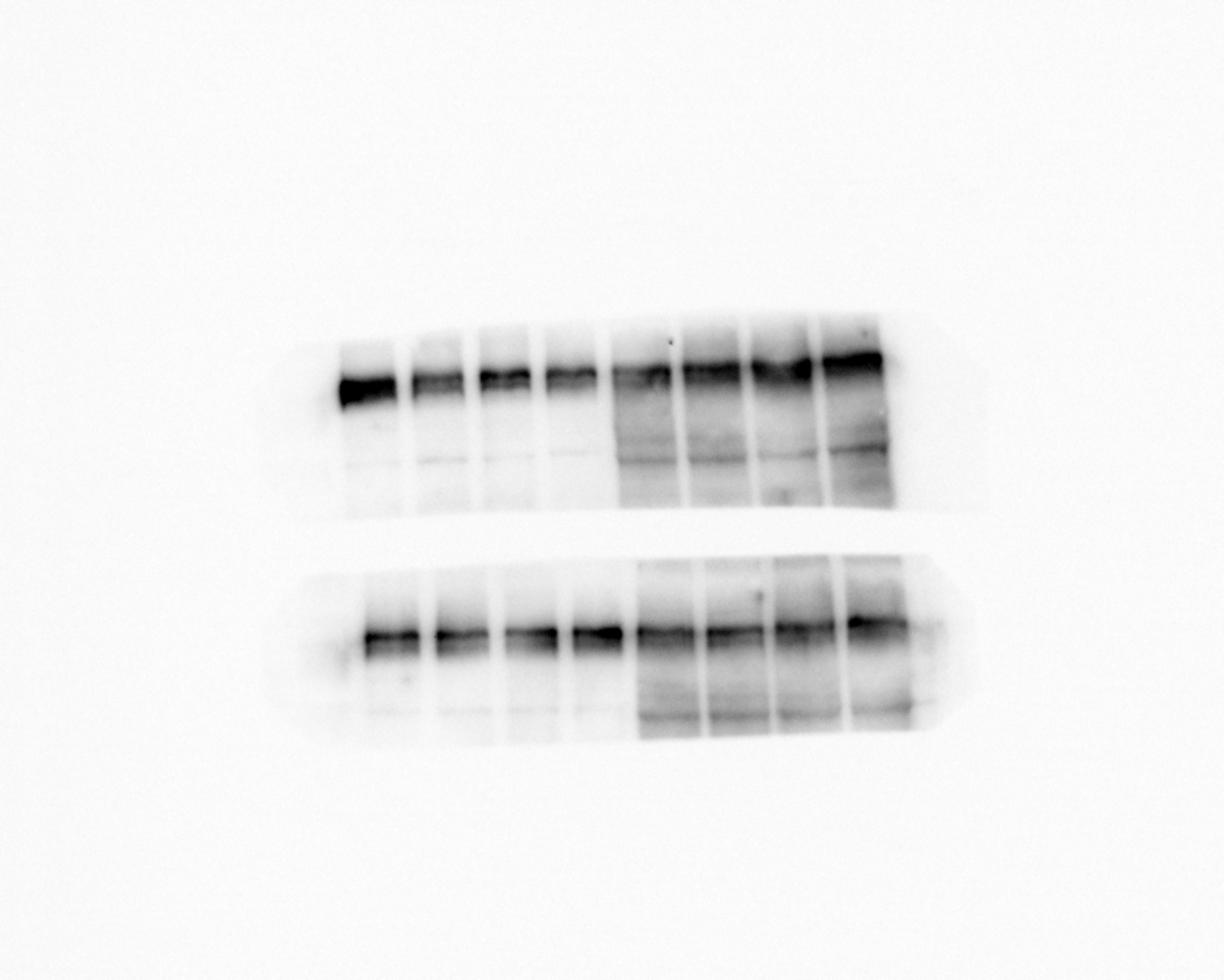

Supplement: Figure 4—figure supplement 1—source data 1. — Including uncropped Western blot images and raw statistics. [file elife-76436-fig4-figsupp1-data1.zip › Figure 4-figure supplement 1-Source Data 1/Figure 4-figure supplement 1J full raw unedited/ced-1-Y1019F-CED-1.tif]

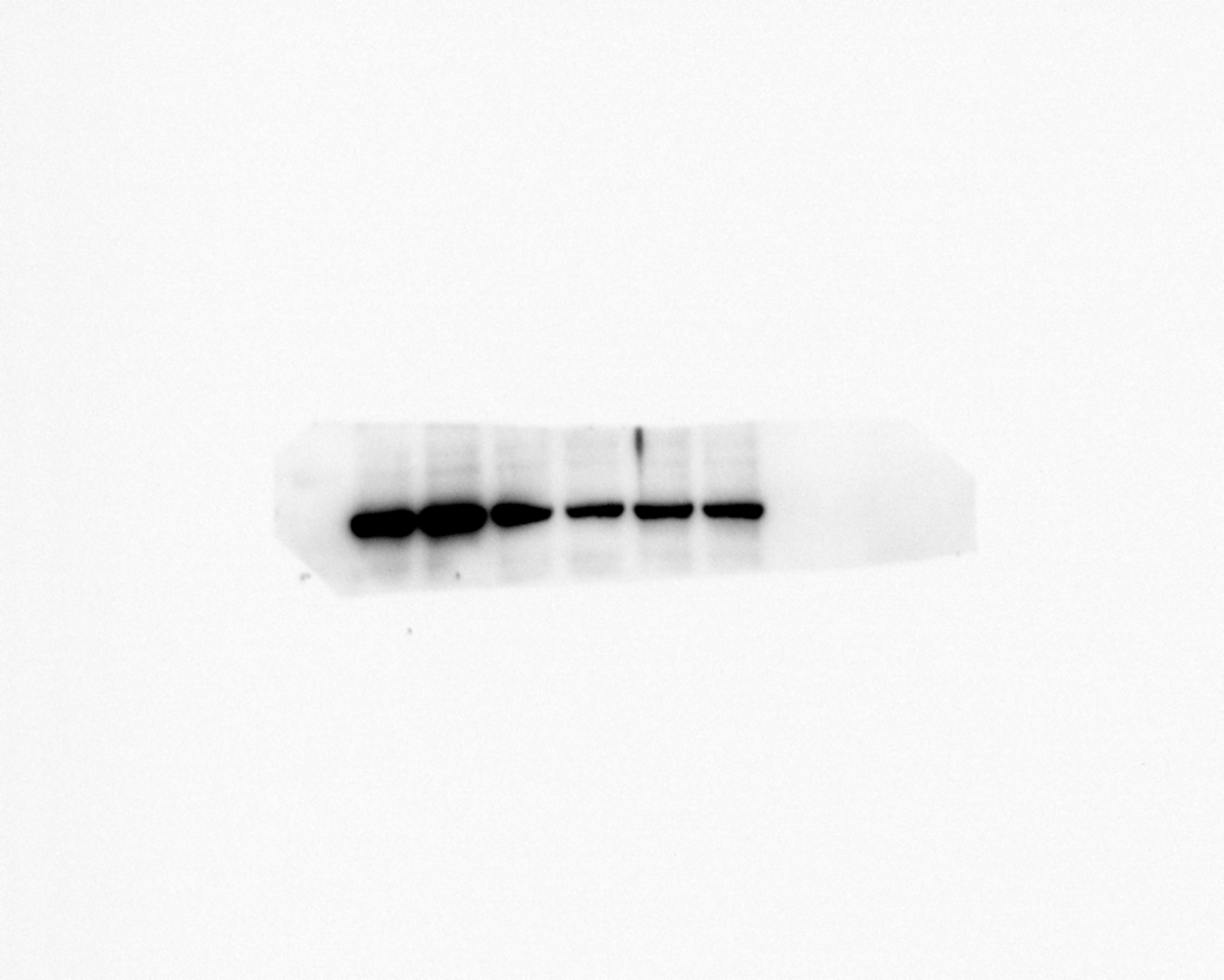

Supplement: Figure 4—figure supplement 1—source data 1. — Including uncropped Western blot images and raw statistics. [file elife-76436-fig4-figsupp1-data1.zip › Figure 4-figure supplement 1-Source Data 1/Figure 4-figure supplement 1L full raw unedited/Input-IB-Actin.tif]

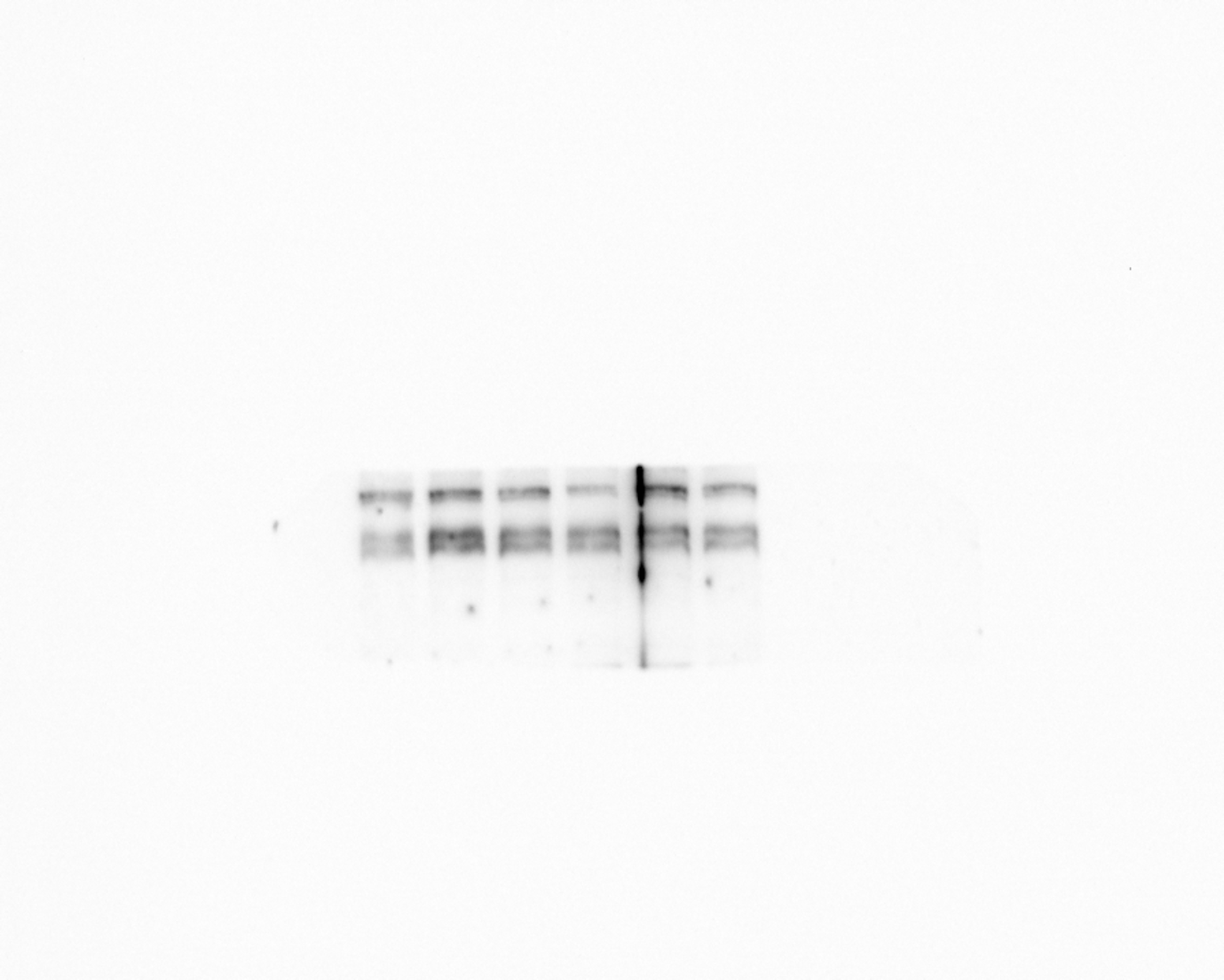

Supplement: Figure 4—figure supplement 1—source data 1. — Including uncropped Western blot images and raw statistics. [file elife-76436-fig4-figsupp1-data1.zip › Figure 4-figure supplement 1-Source Data 1/Figure 4-figure supplement 1L full raw unedited/Input-IB-FLAG.tif]

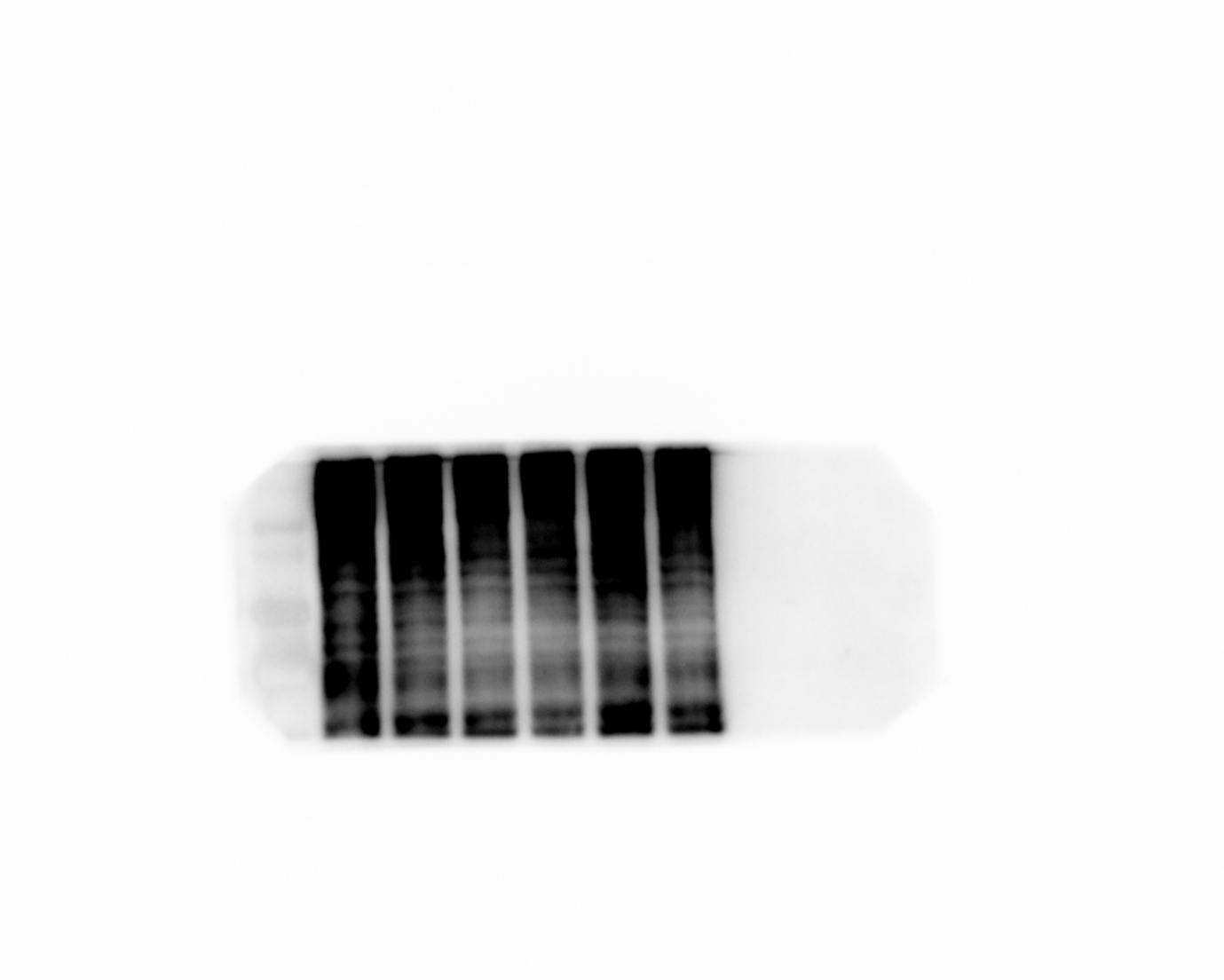

Supplement: Figure 4—figure supplement 1—source data 1. — Including uncropped Western blot images and raw statistics. [file elife-76436-fig4-figsupp1-data1.zip › Figure 4-figure supplement 1-Source Data 1/Figure 4-figure supplement 1L full raw unedited/Input-IB-HA.tif]

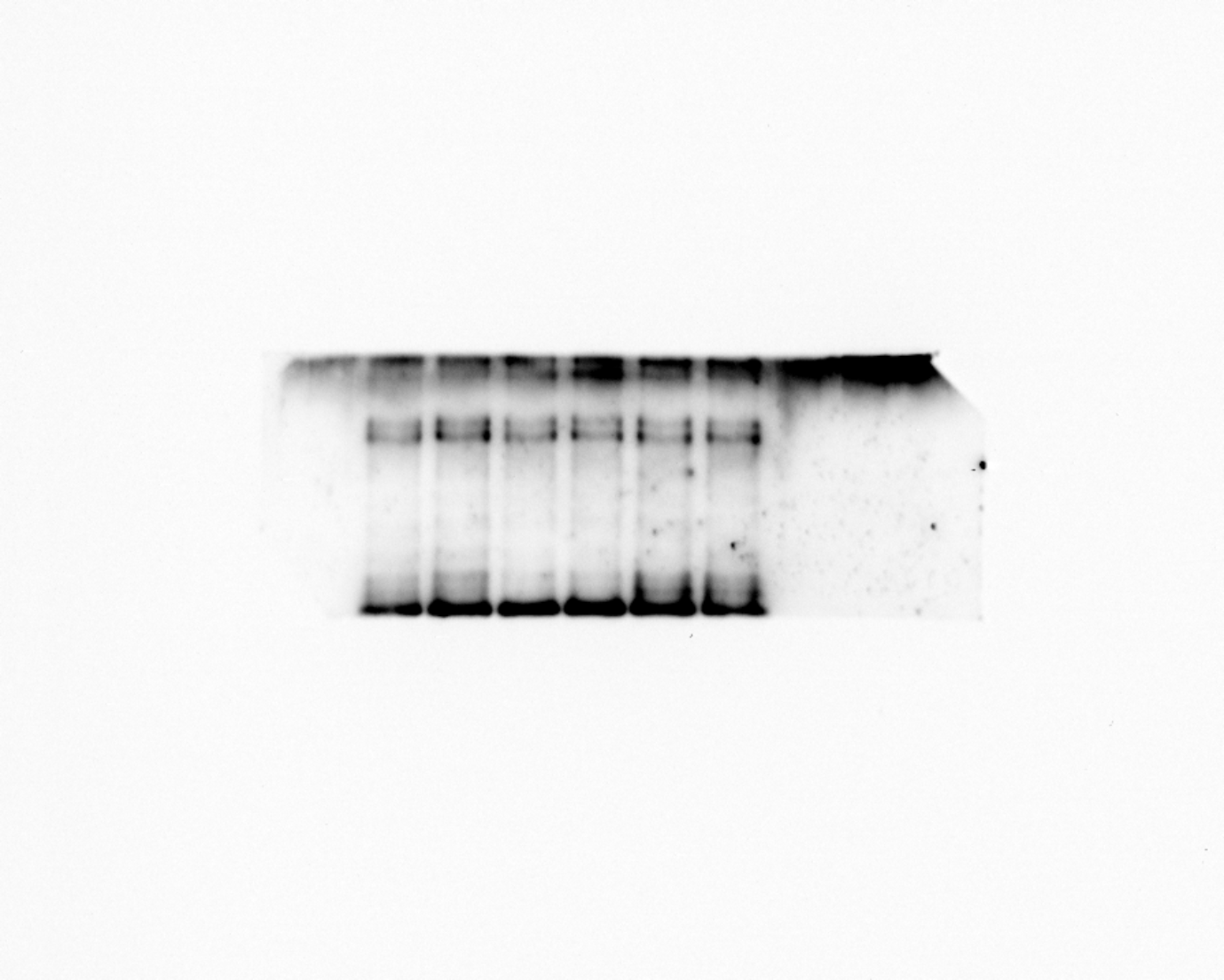

Supplement: Figure 4—figure supplement 1—source data 1. — Including uncropped Western blot images and raw statistics. [file elife-76436-fig4-figsupp1-data1.zip › Figure 4-figure supplement 1-Source Data 1/Figure 4-figure supplement 1L full raw unedited/IP-IB-FLAG.tif]

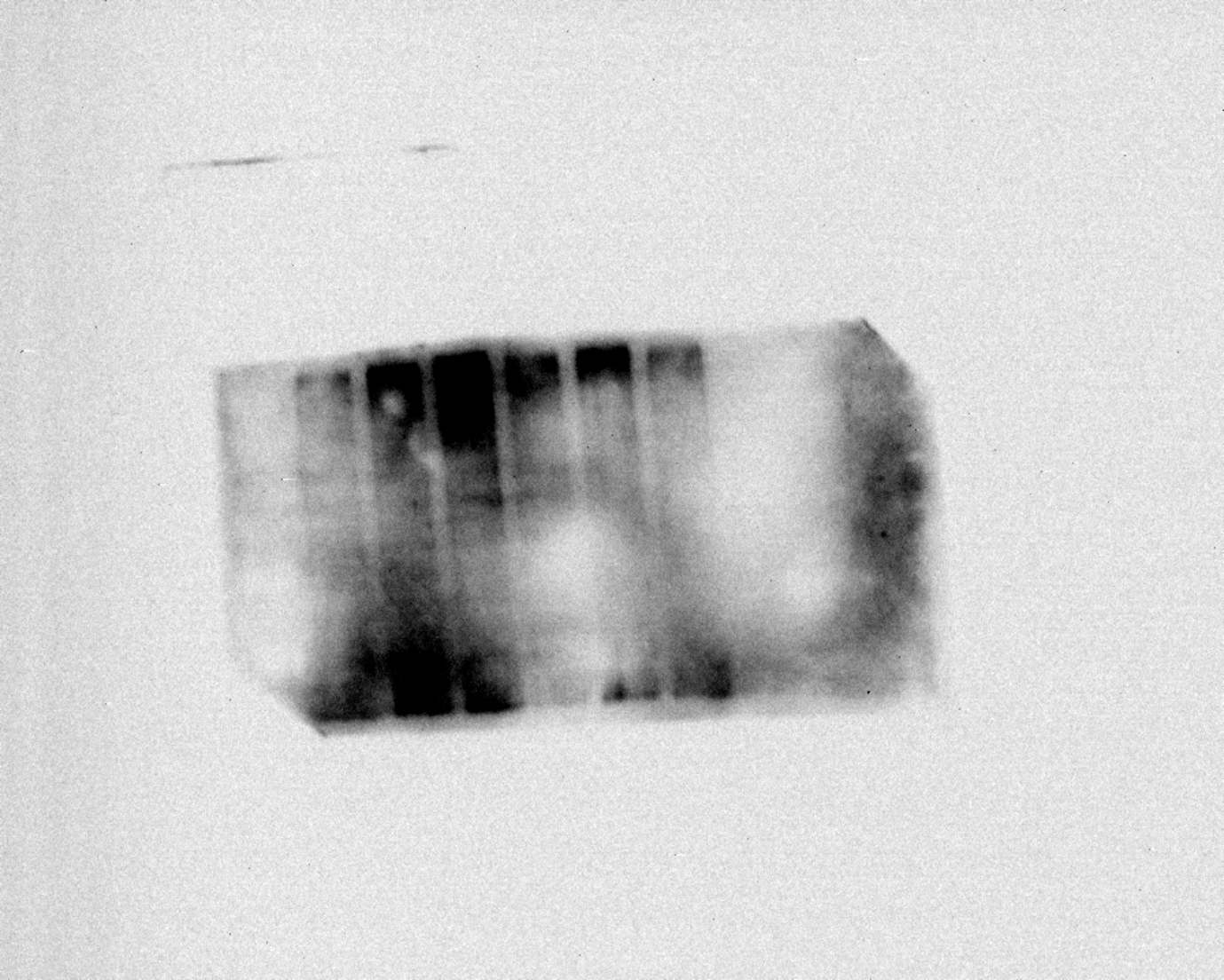

Supplement: Figure 4—figure supplement 1—source data 1. — Including uncropped Western blot images and raw statistics. [file elife-76436-fig4-figsupp1-data1.zip › Figure 4-figure supplement 1-Source Data 1/Figure 4-figure supplement 1L full raw unedited/IP-IB-HA.tif]

Figure 5A


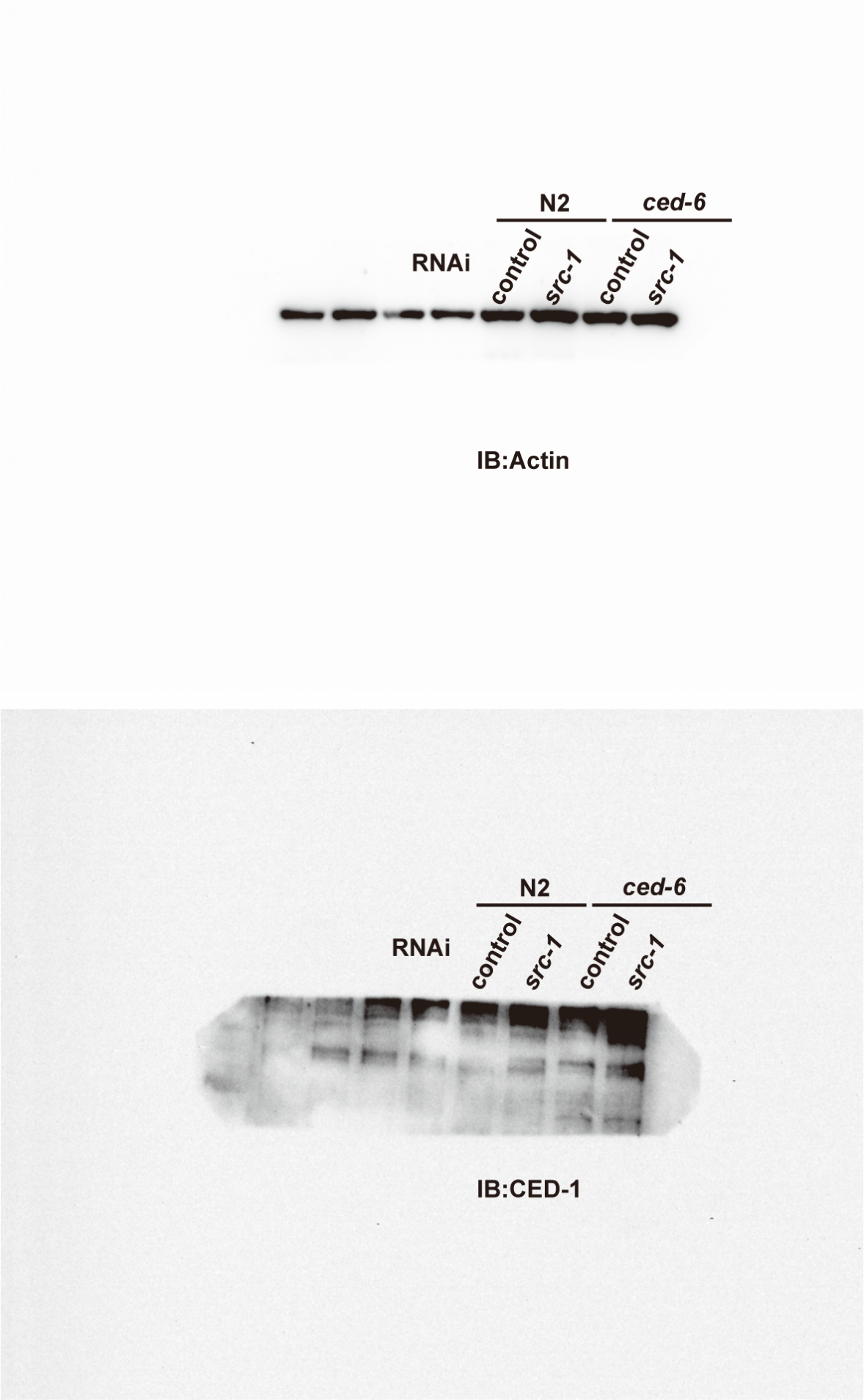


Figure 5C


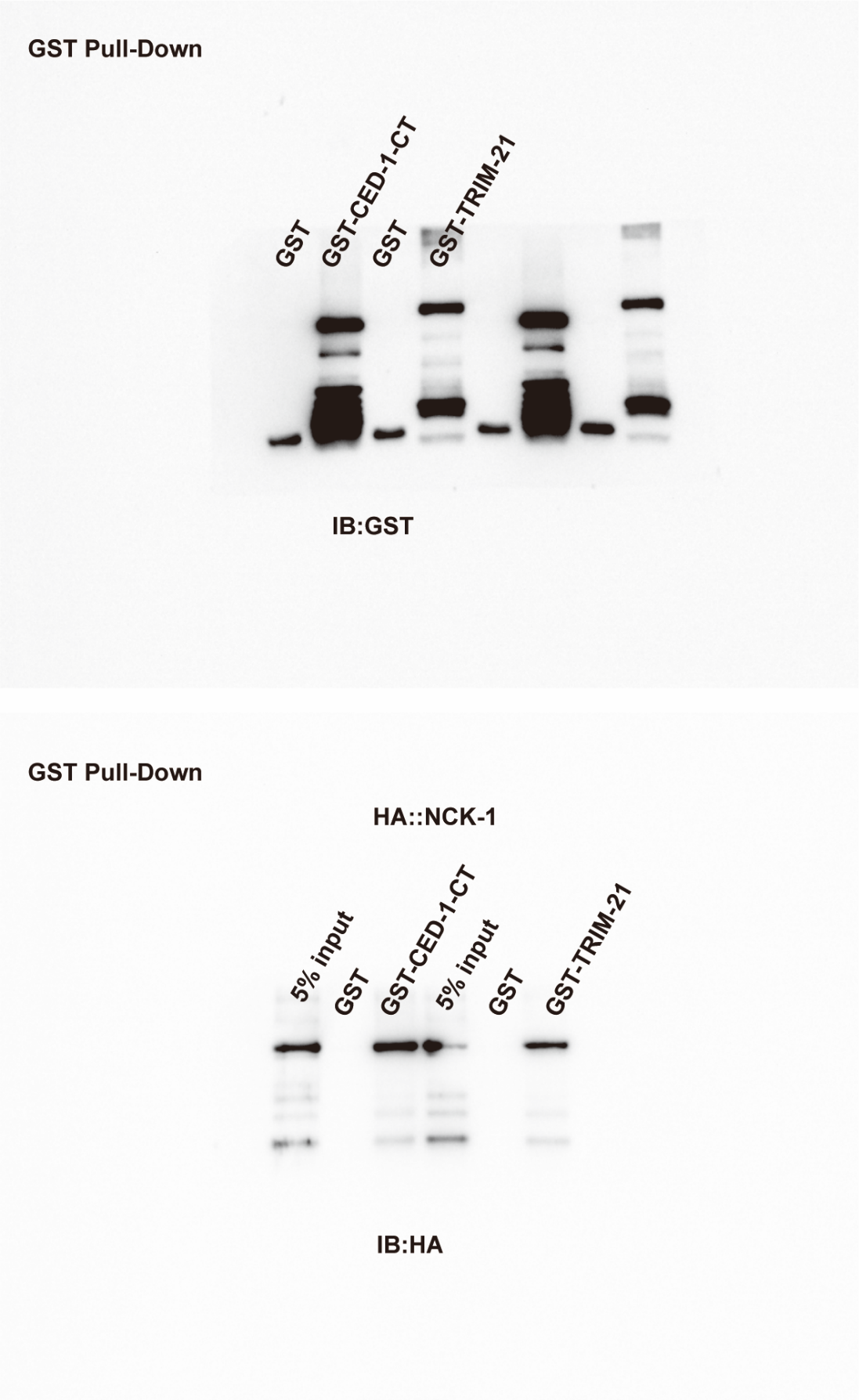


Figure 5D


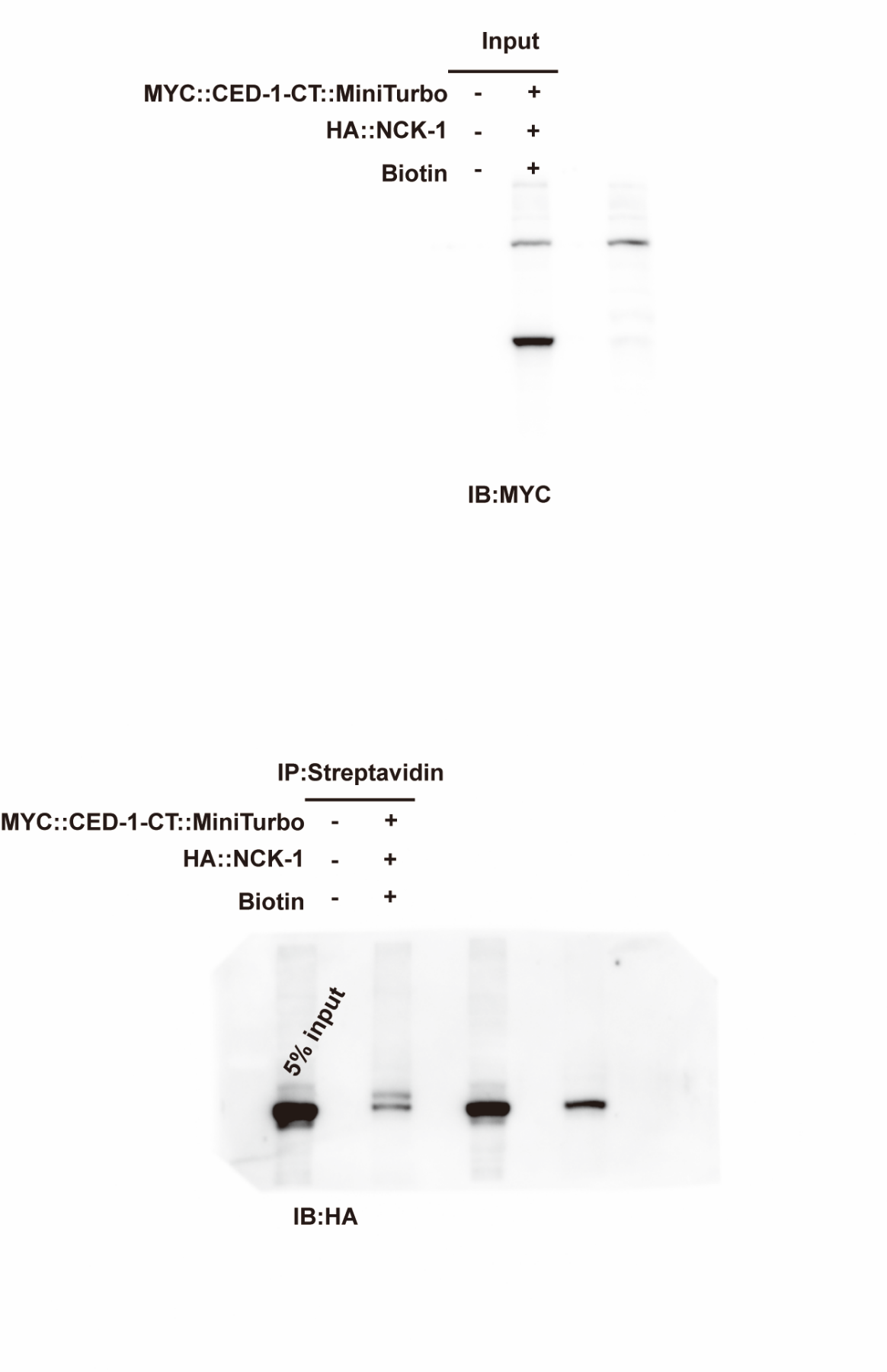


Figure 5E


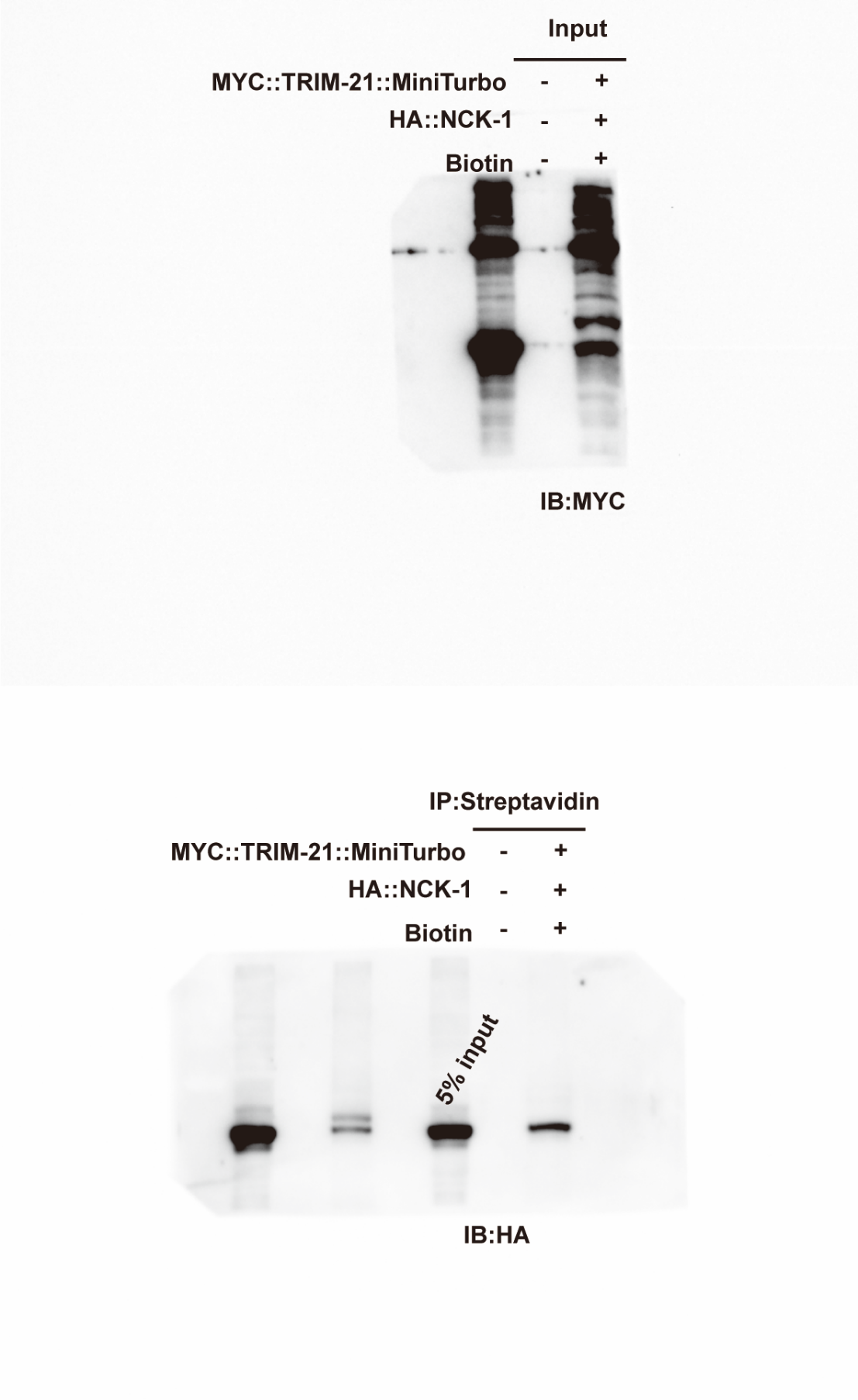


Figure 5F


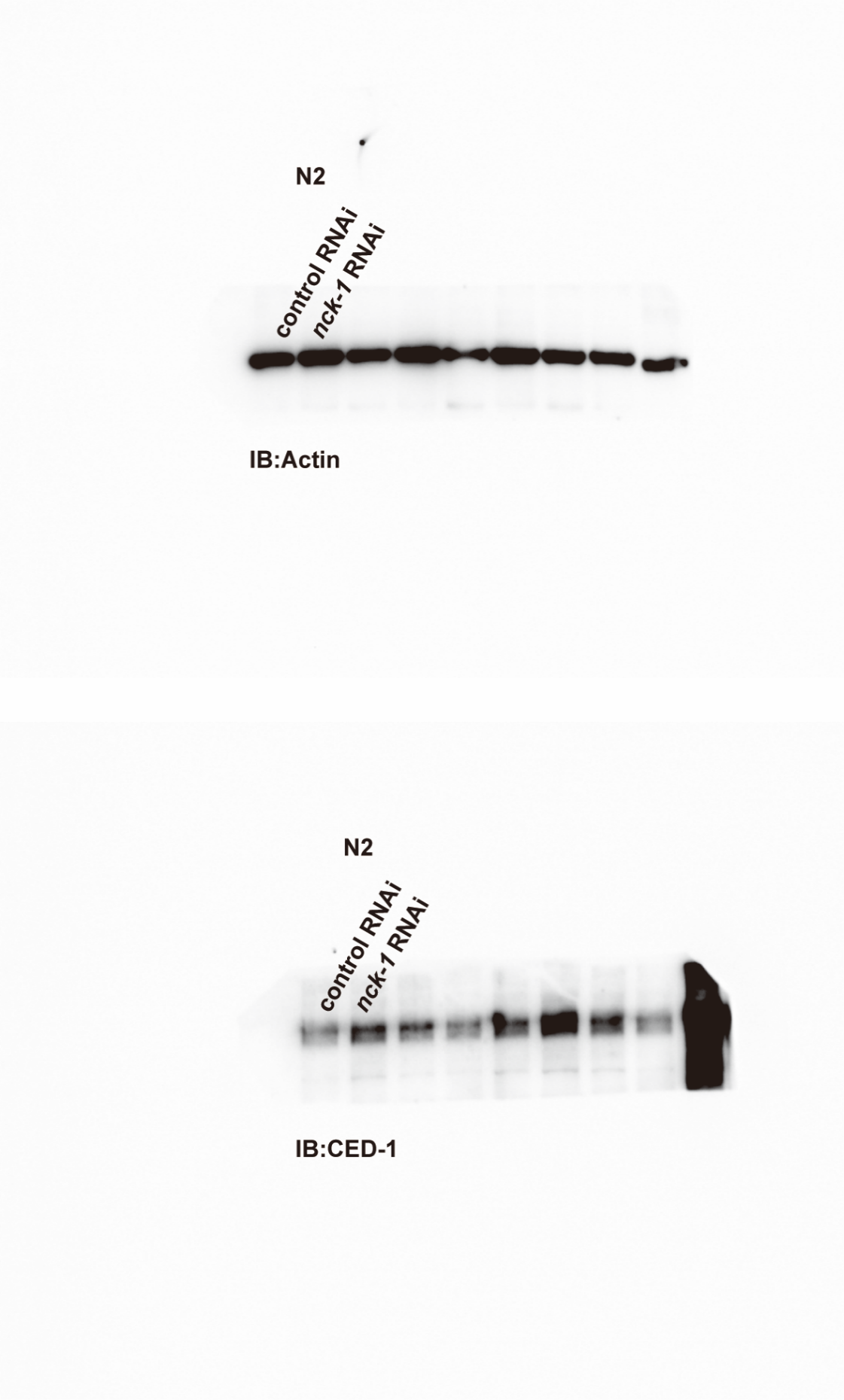


Figure 5G


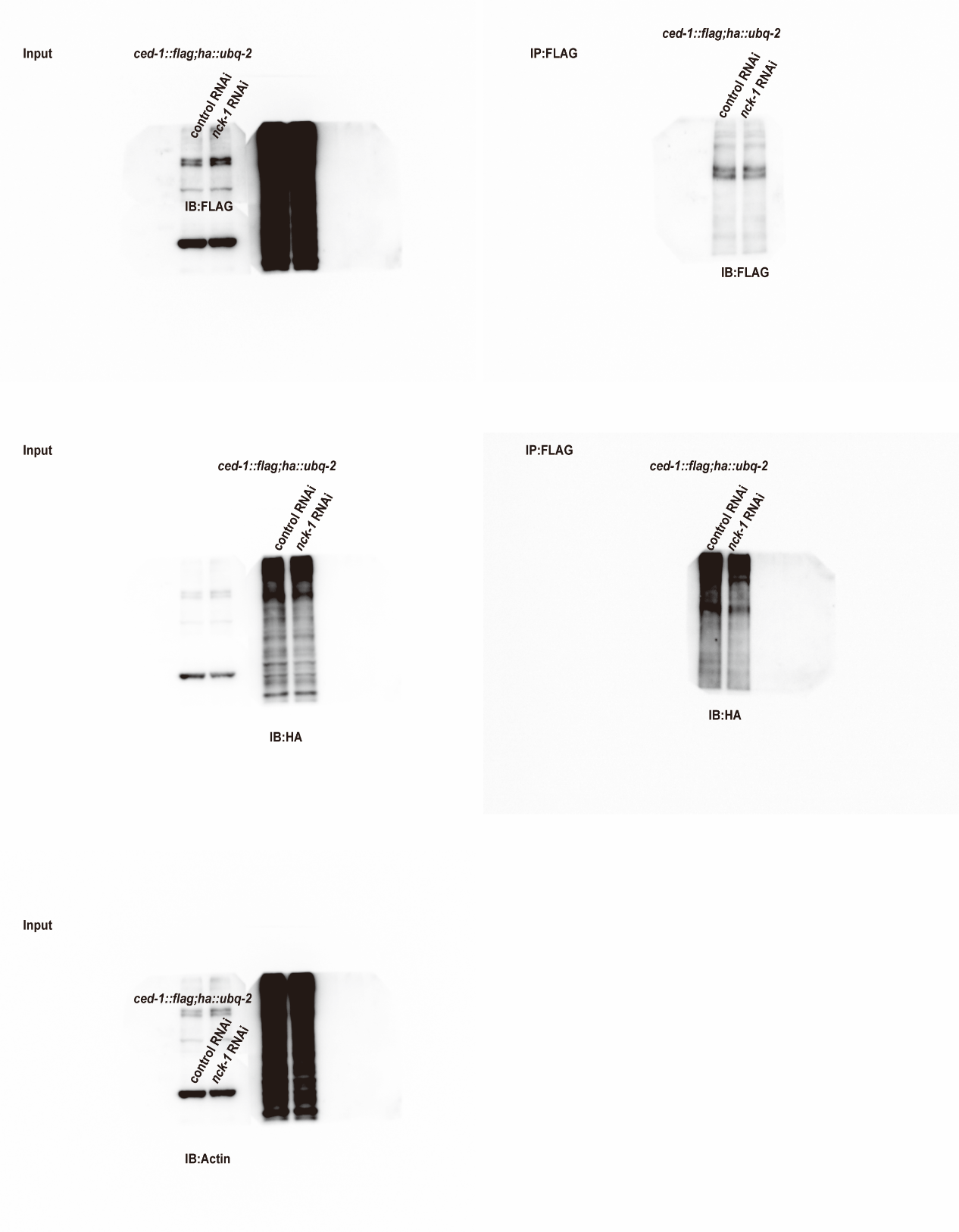


Figure 5K


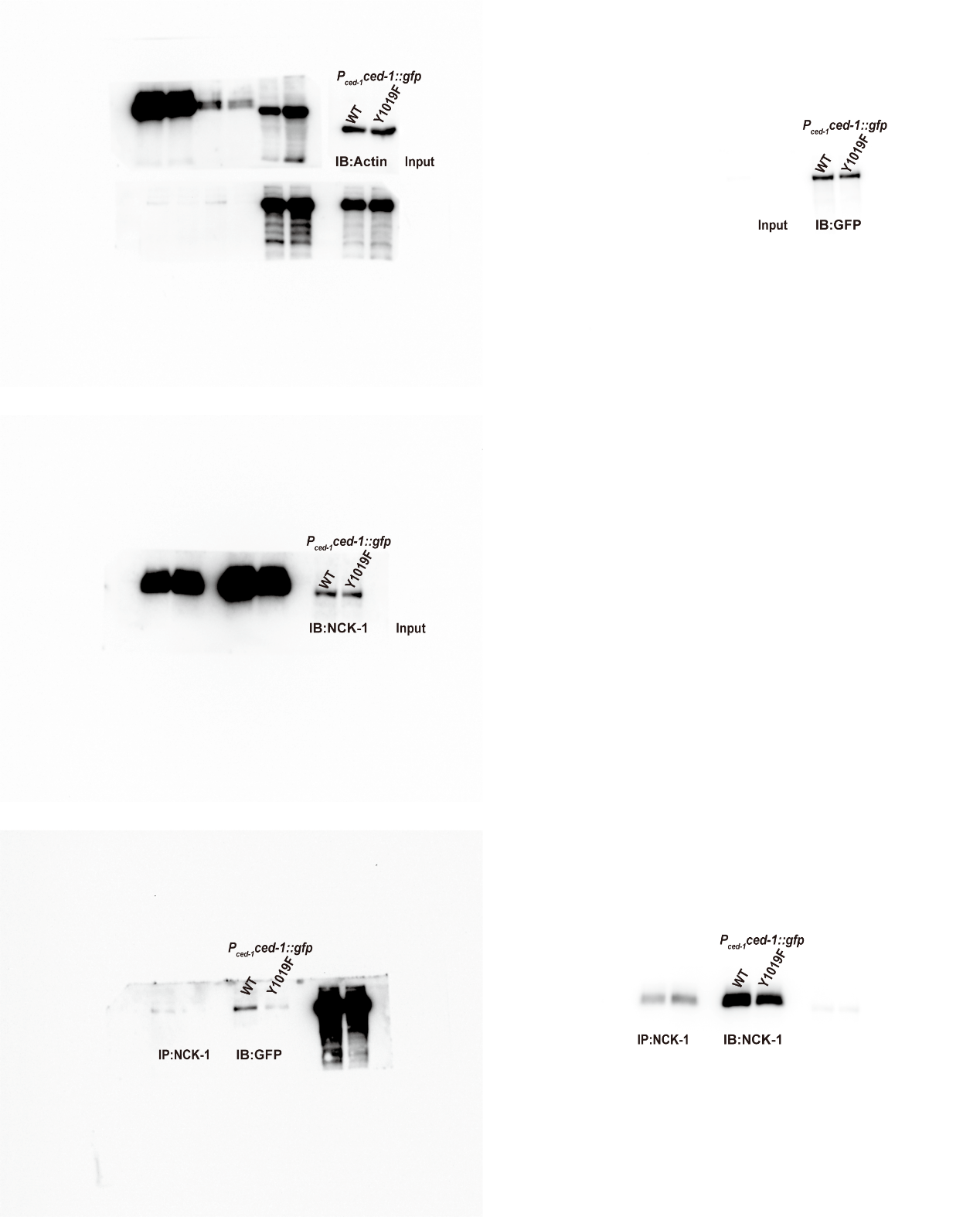

Supplement: Figure 5—source data 1. — Including uncropped Western blot images and raw statistics. [file elife-76436-fig5-data1.zip › Figure 5-Source Data 1/Figure 5 uncroppped blot with relevant bands.docx]

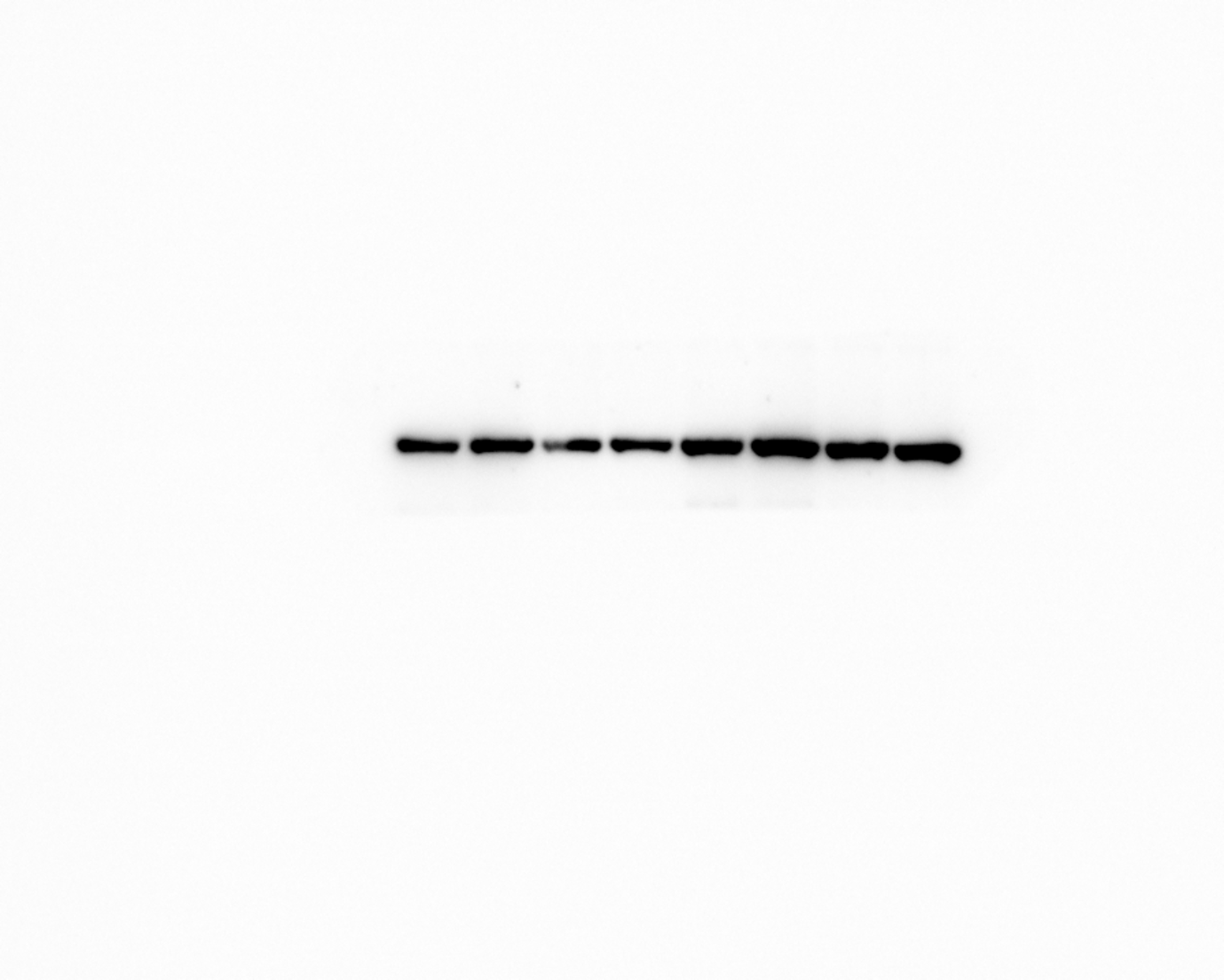

Supplement: Figure 5—source data 1. — Including uncropped Western blot images and raw statistics. [file elife-76436-fig5-data1.zip › Figure 5-Source Data 1/Figure 5A full raw unedited/IB-Actin.tif]

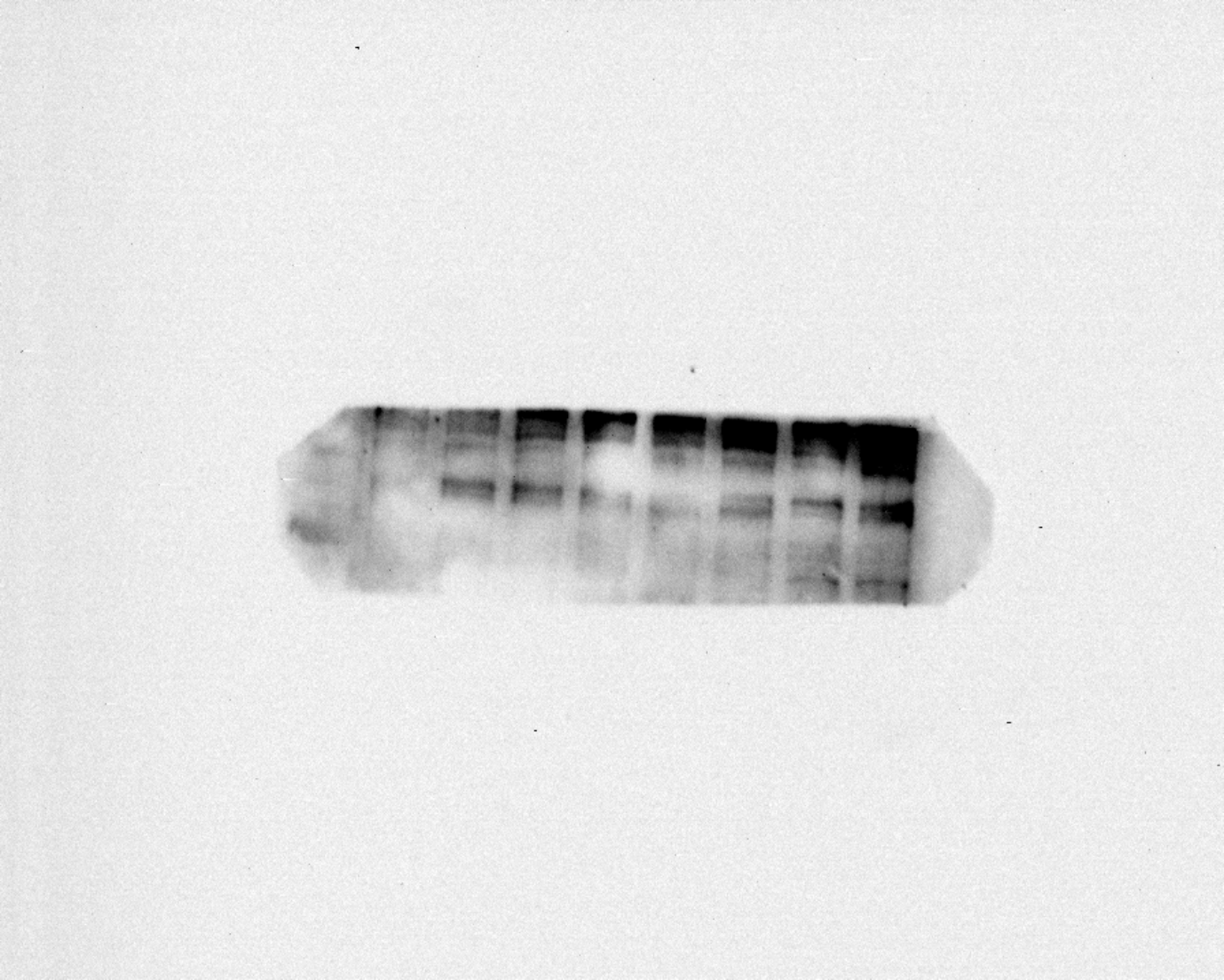

Supplement: Figure 5—source data 1. — Including uncropped Western blot images and raw statistics. [file elife-76436-fig5-data1.zip › Figure 5-Source Data 1/Figure 5A full raw unedited/IB-CED-1.tif]

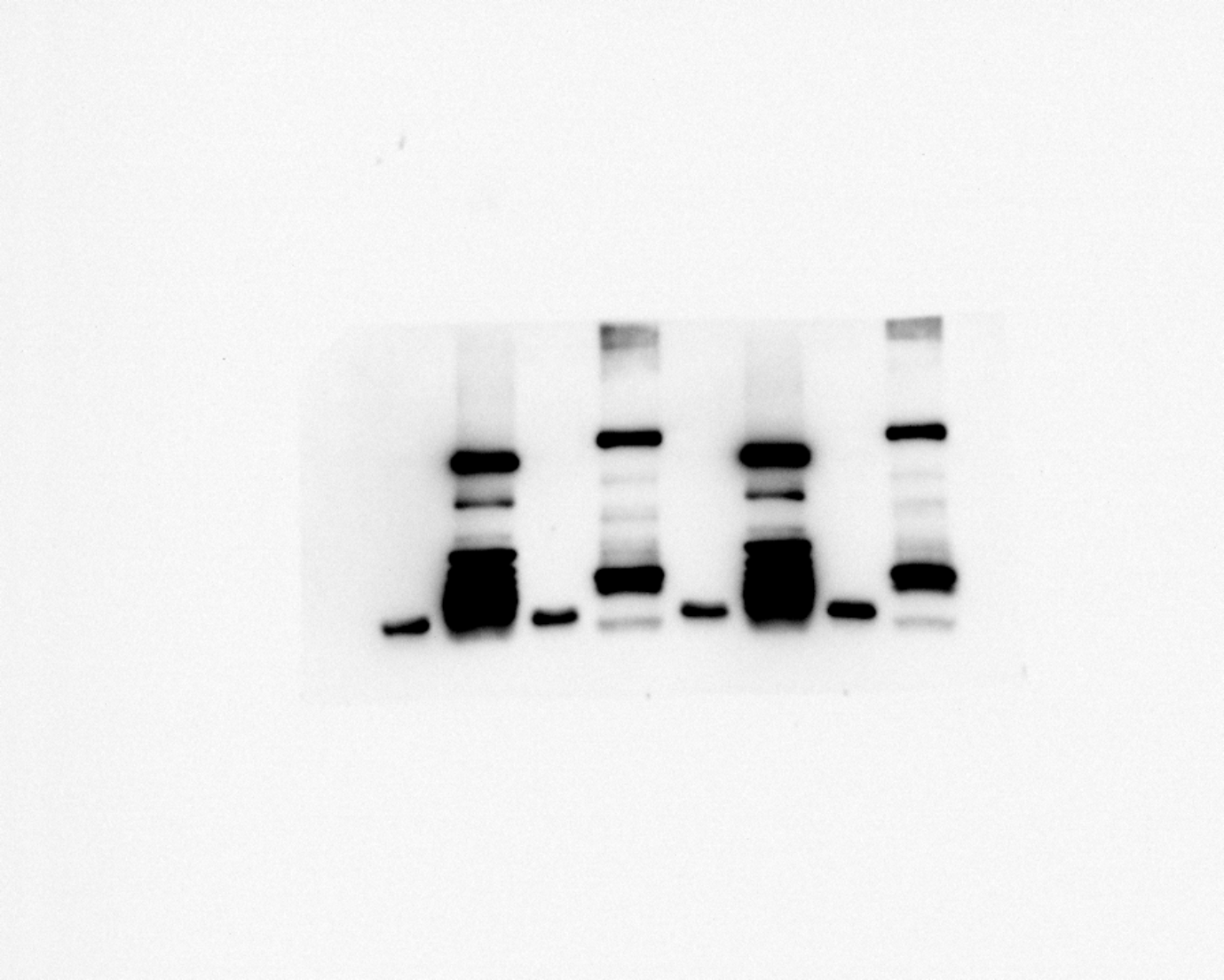

Supplement: Figure 5—source data 1. — Including uncropped Western blot images and raw statistics. [file elife-76436-fig5-data1.zip › Figure 5-Source Data 1/Figure 5C full raw unedited/IB-GST.tif]

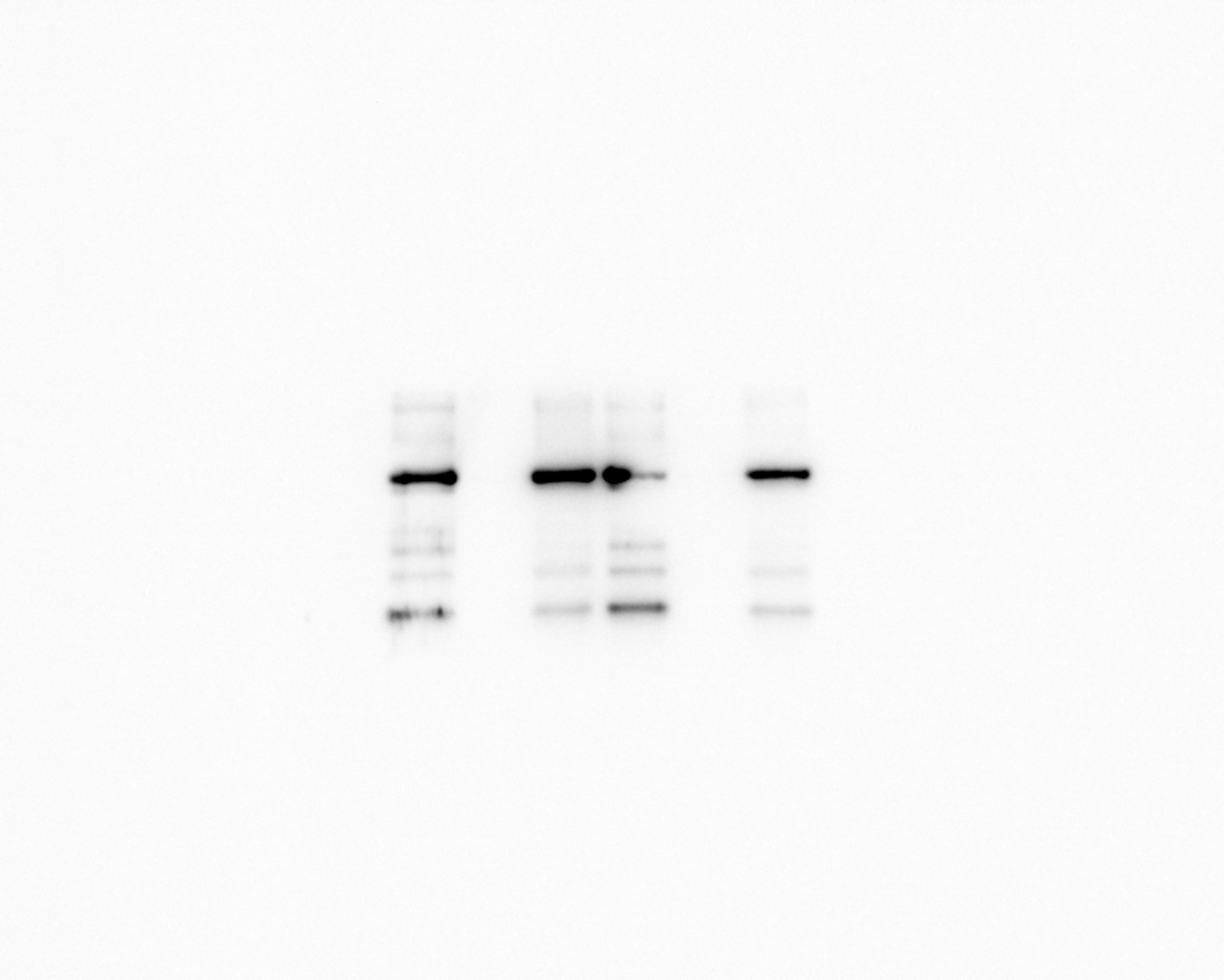

Supplement: Figure 5—source data 1. — Including uncropped Western blot images and raw statistics. [file elife-76436-fig5-data1.zip › Figure 5-Source Data 1/Figure 5C full raw unedited/IB-HA.tif]

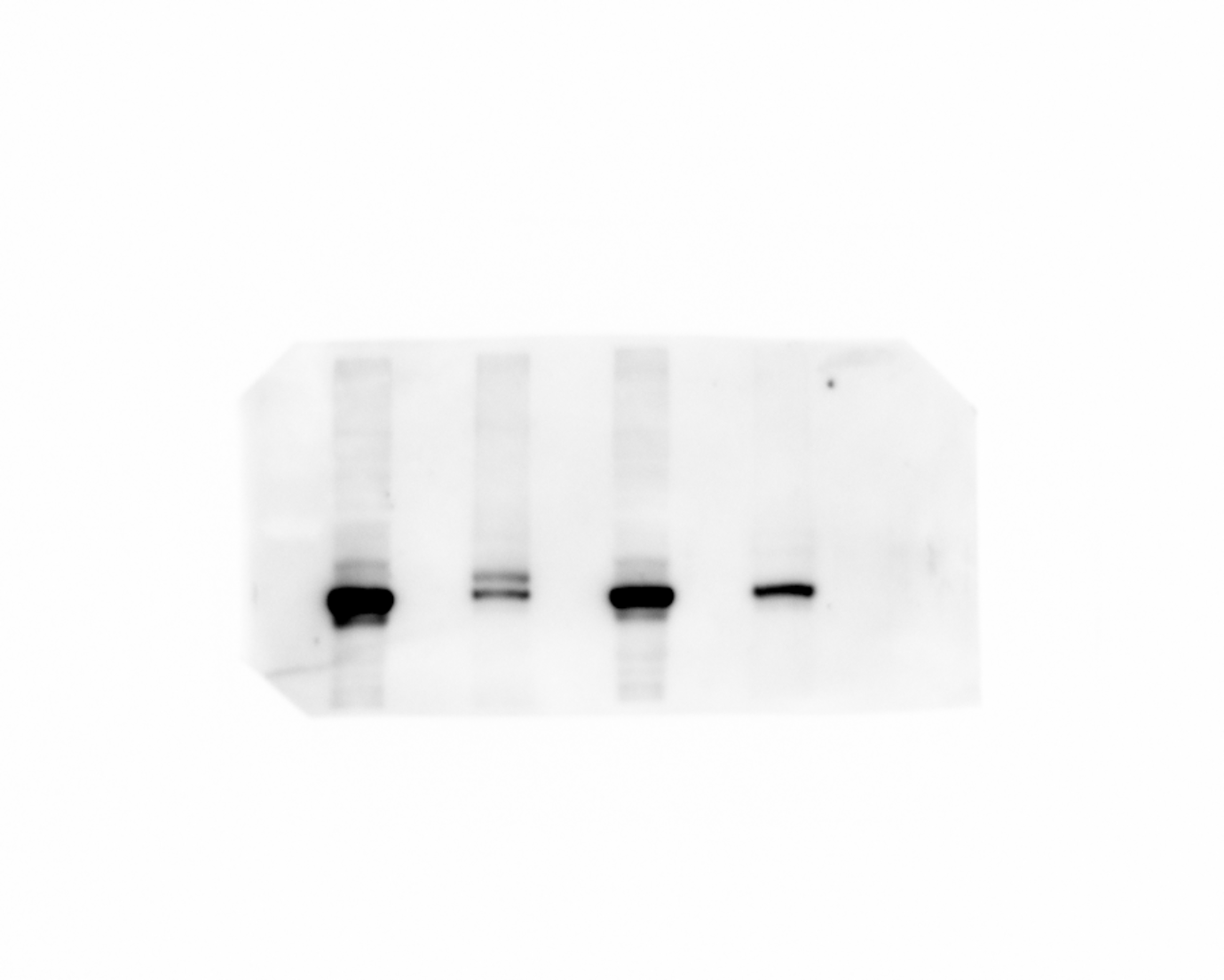

Supplement: Figure 5—source data 1. — Including uncropped Western blot images and raw statistics. [file elife-76436-fig5-data1.zip › Figure 5-Source Data 1/Figure 5D full raw unedited/IB-HA.tif]

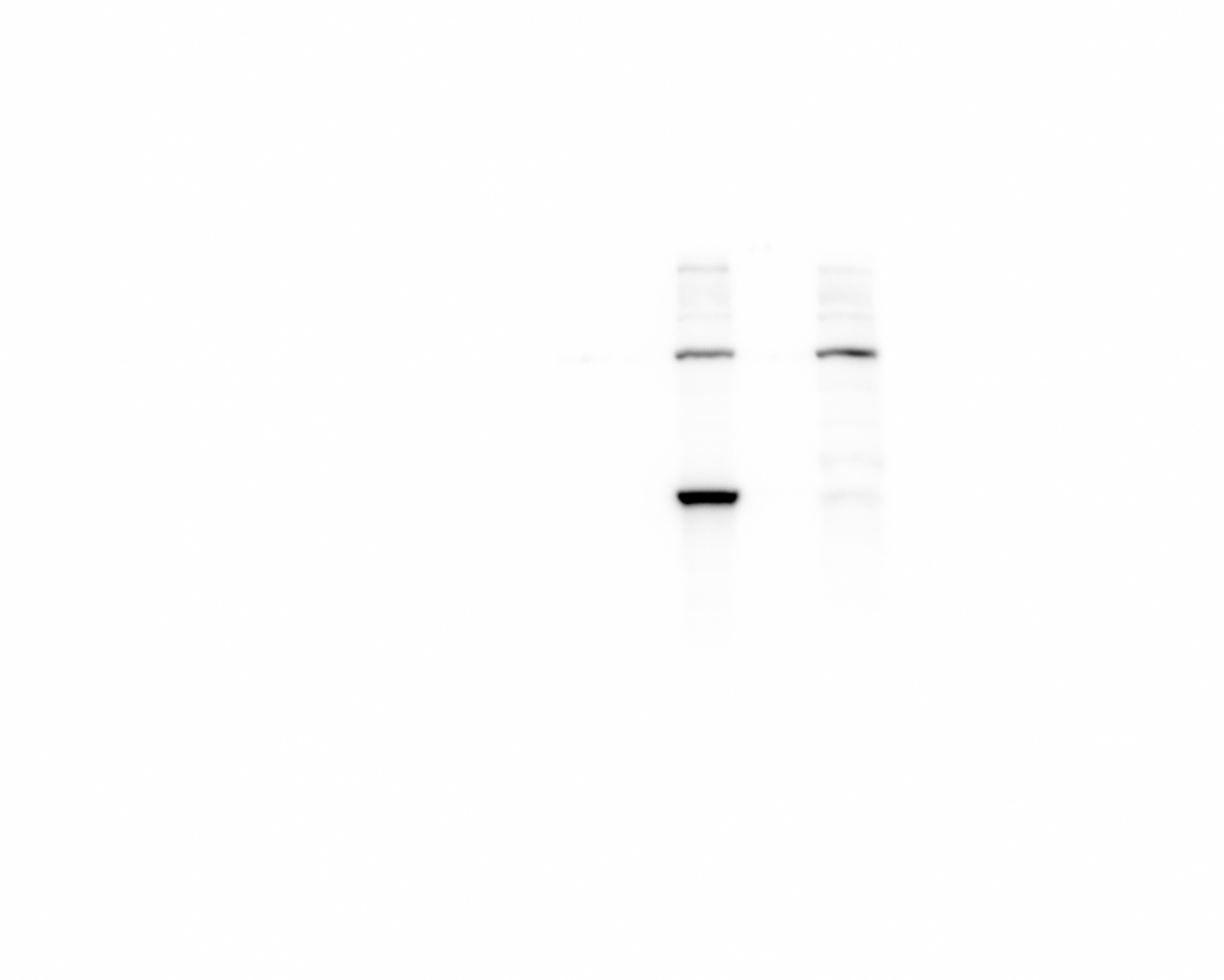

Supplement: Figure 5—source data 1. — Including uncropped Western blot images and raw statistics. [file elife-76436-fig5-data1.zip › Figure 5-Source Data 1/Figure 5D full raw unedited/IB-MYC.tif]

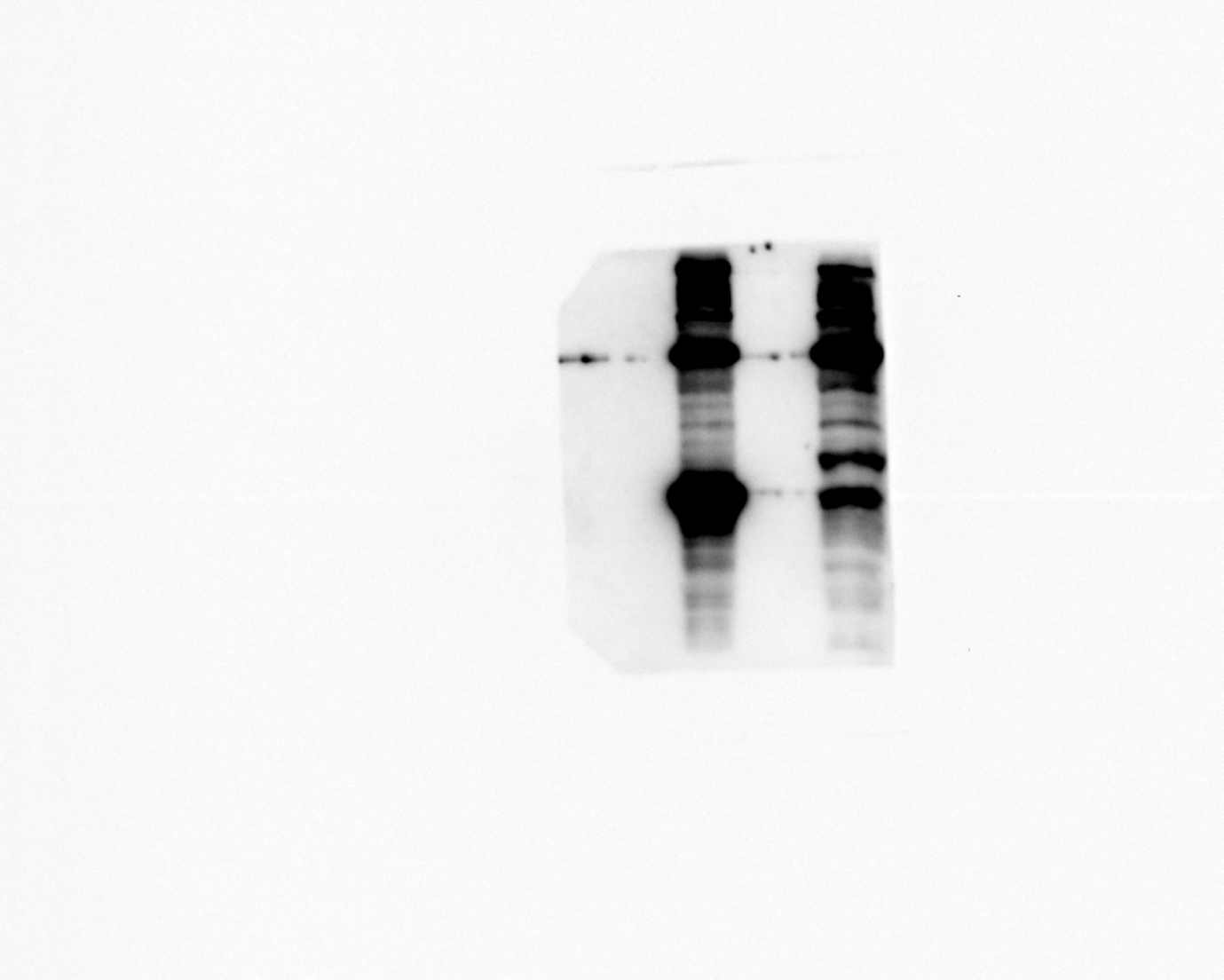

Supplement: Figure 5—source data 1. — Including uncropped Western blot images and raw statistics. [file elife-76436-fig5-data1.zip › Figure 5-Source Data 1/Figure 5E full raw unedited/IB-MYC.tif]

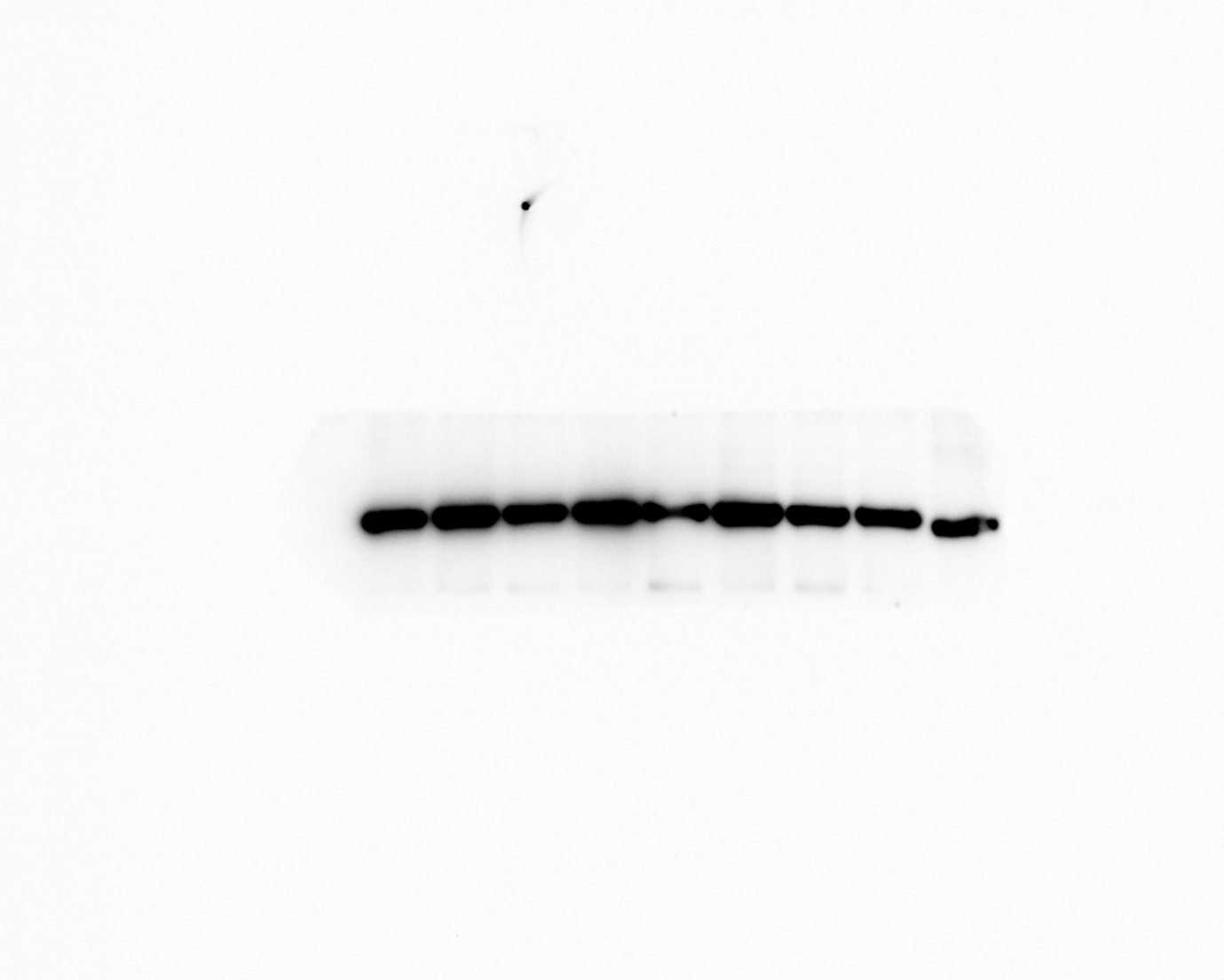

Supplement: Figure 5—source data 1. — Including uncropped Western blot images and raw statistics. [file elife-76436-fig5-data1.zip › Figure 5-Source Data 1/Figure 5F full raw unedited/IB-Actin.tif]

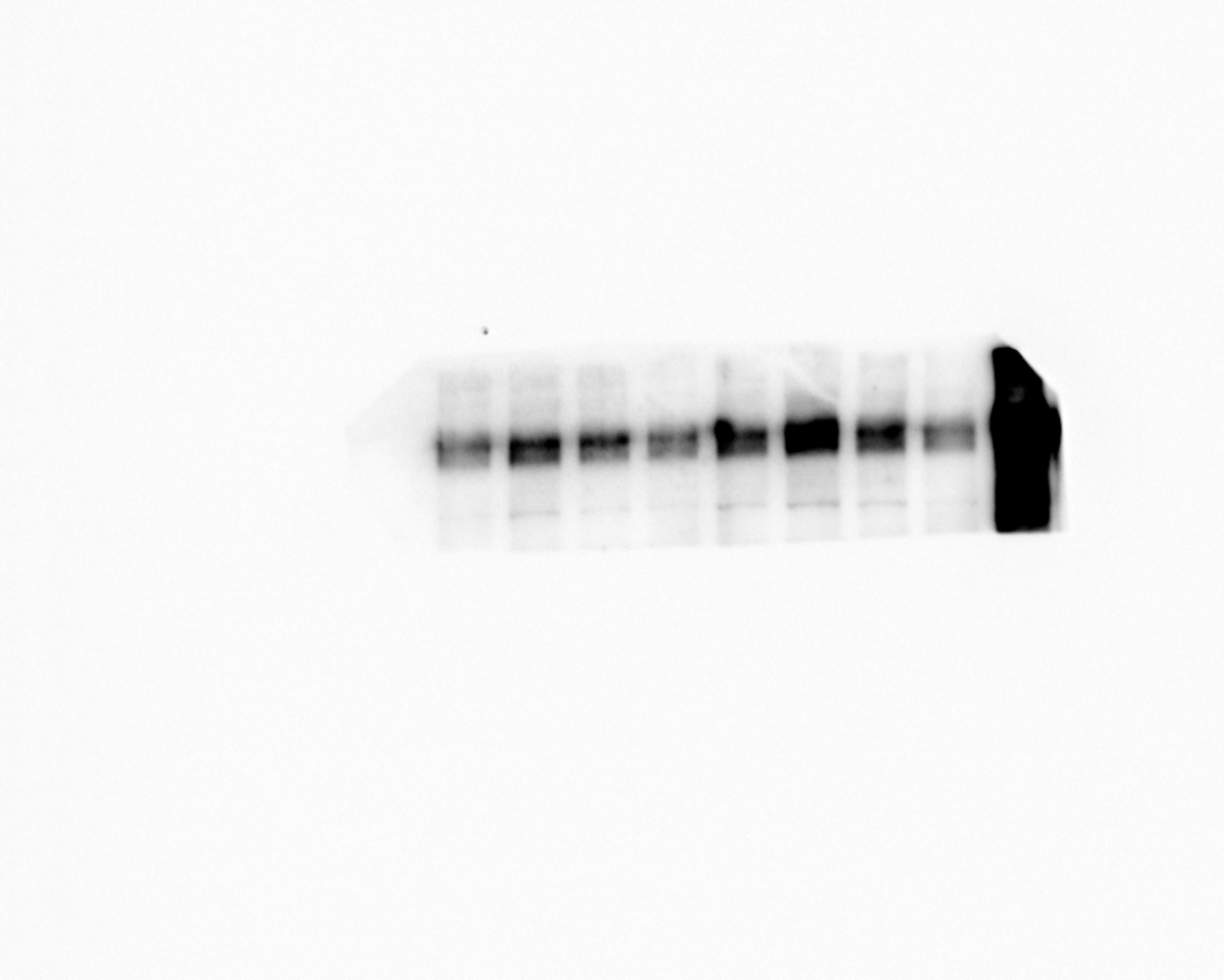

Supplement: Figure 5—source data 1. — Including uncropped Western blot images and raw statistics. [file elife-76436-fig5-data1.zip › Figure 5-Source Data 1/Figure 5F full raw unedited/IB-CED-1.tif]

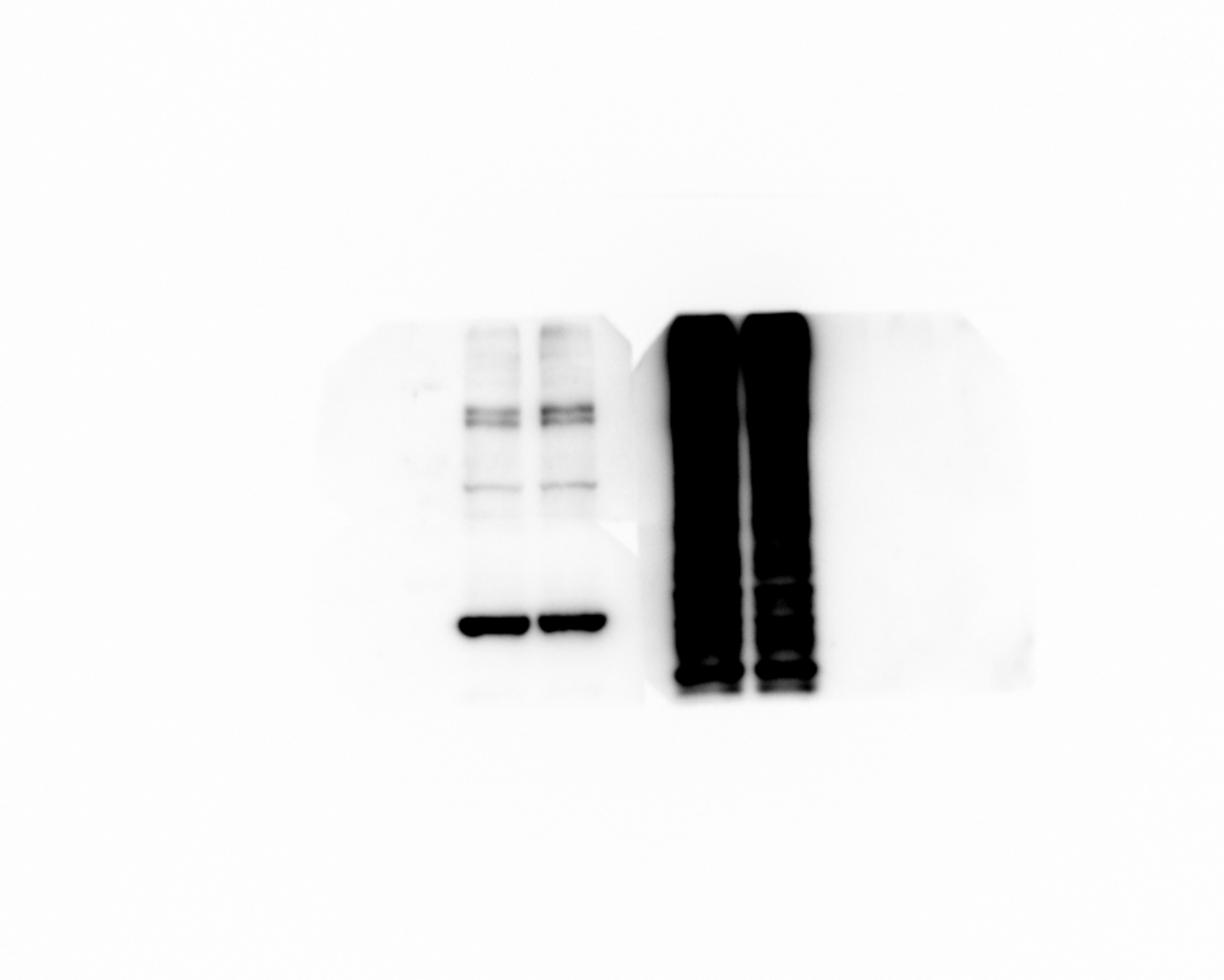

Supplement: Figure 5—source data 1. — Including uncropped Western blot images and raw statistics. [file elife-76436-fig5-data1.zip › Figure 5-Source Data 1/Figure 5G full raw unedited/Input-IB-Actin.tif]

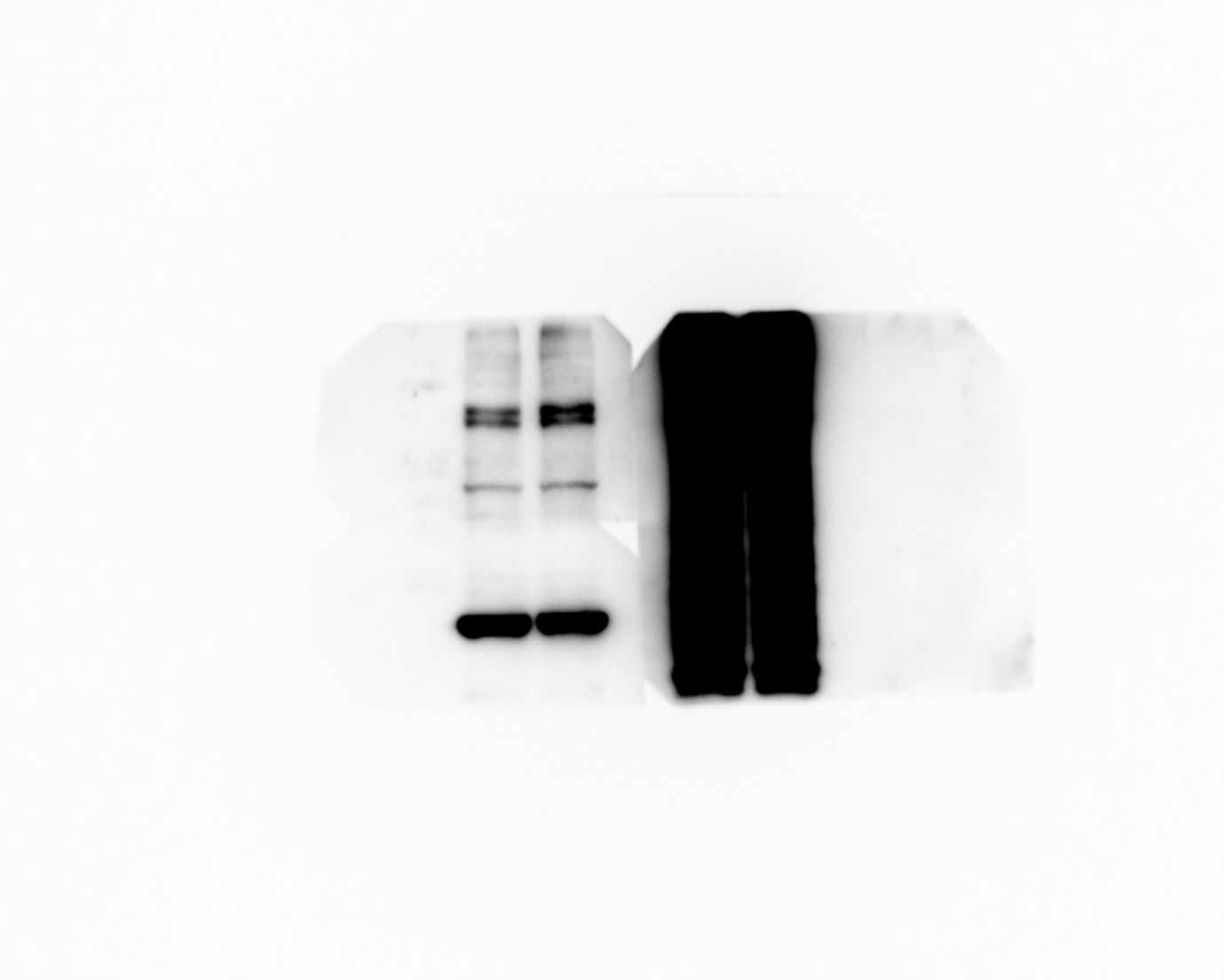

Supplement: Figure 5—source data 1. — Including uncropped Western blot images and raw statistics. [file elife-76436-fig5-data1.zip › Figure 5-Source Data 1/Figure 5G full raw unedited/Input-IB-FLAG.tif]

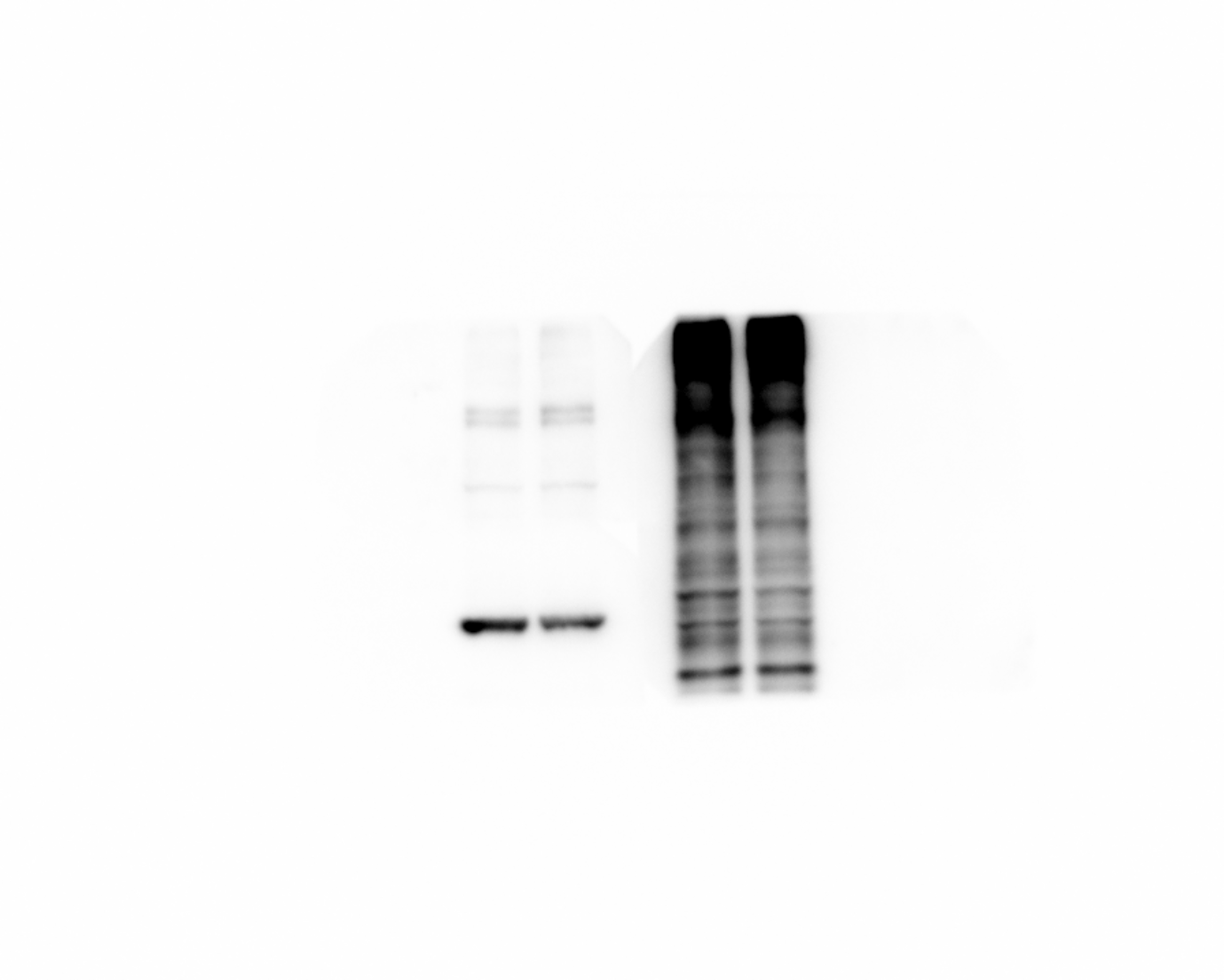

Supplement: Figure 5—source data 1. — Including uncropped Western blot images and raw statistics. [file elife-76436-fig5-data1.zip › Figure 5-Source Data 1/Figure 5G full raw unedited/Input-IB-HA.tif]

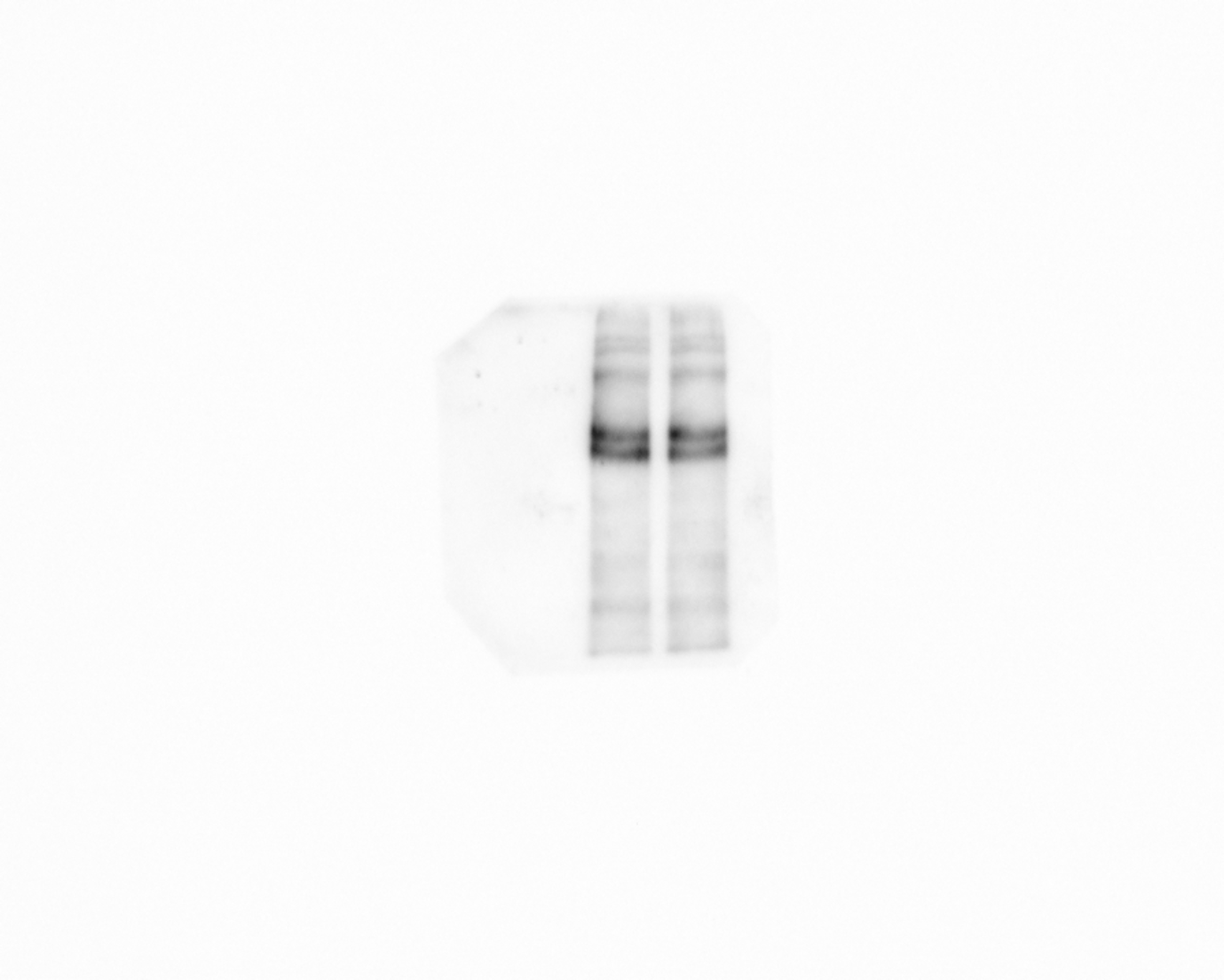

Supplement: Figure 5—source data 1. — Including uncropped Western blot images and raw statistics. [file elife-76436-fig5-data1.zip › Figure 5-Source Data 1/Figure 5G full raw unedited/IP-IB-FLAG.tif]

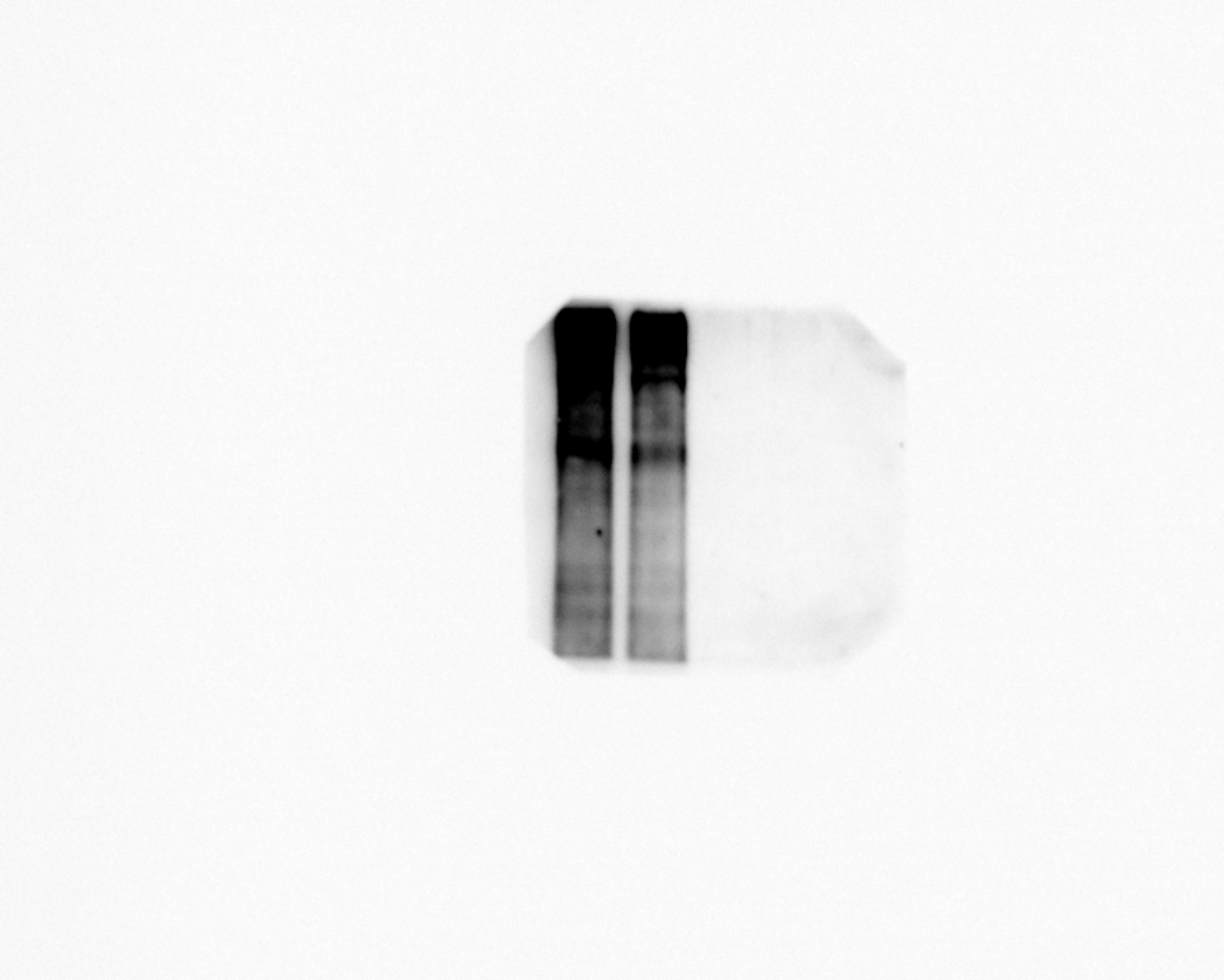

Supplement: Figure 5—source data 1. — Including uncropped Western blot images and raw statistics. [file elife-76436-fig5-data1.zip › Figure 5-Source Data 1/Figure 5G full raw unedited/IP-IB-HA.tif]

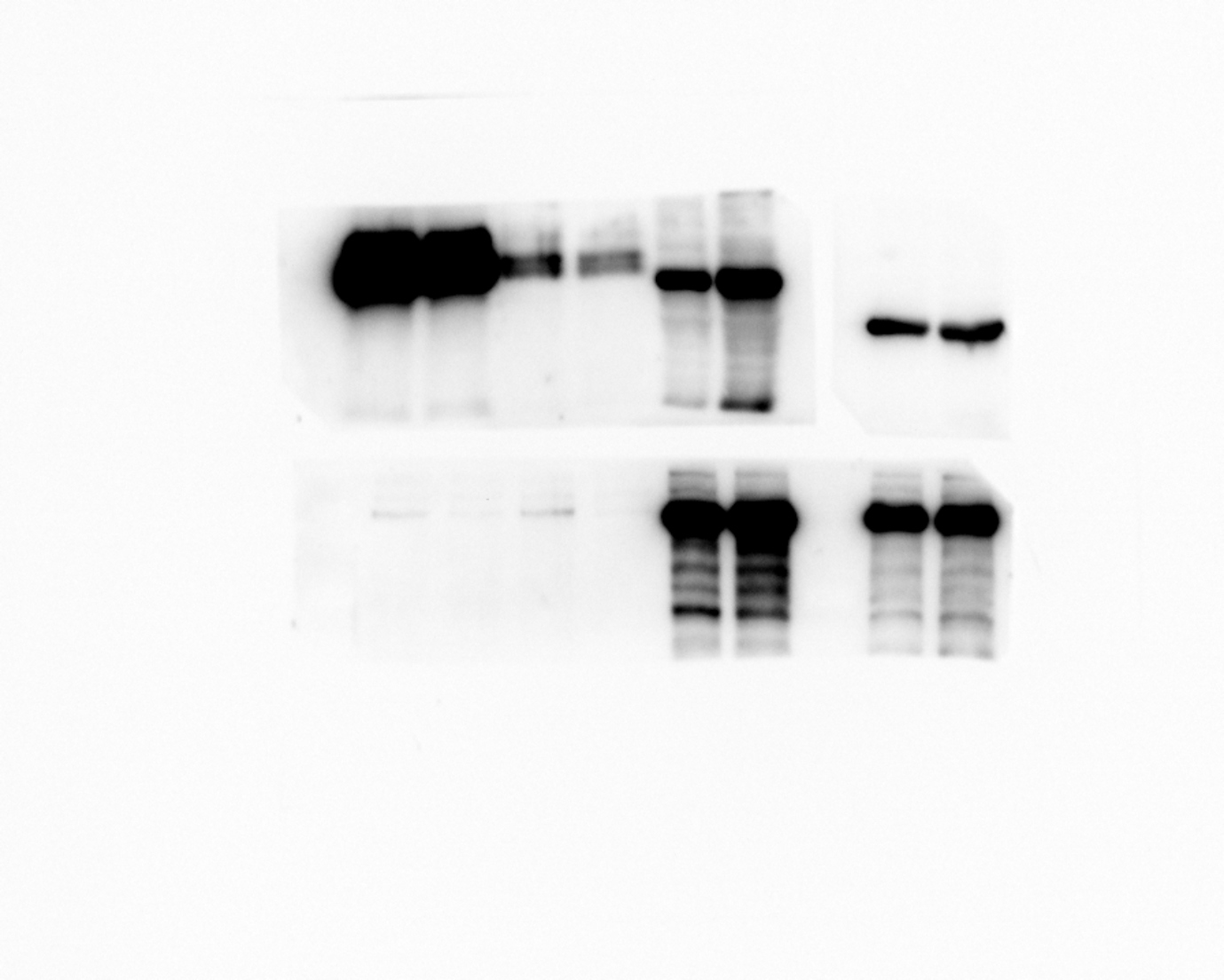

Supplement: Figure 5—source data 1. — Including uncropped Western blot images and raw statistics. [file elife-76436-fig5-data1.zip › Figure 5-Source Data 1/Figure 5K full raw unedited/Input-IB-Actin.tif]

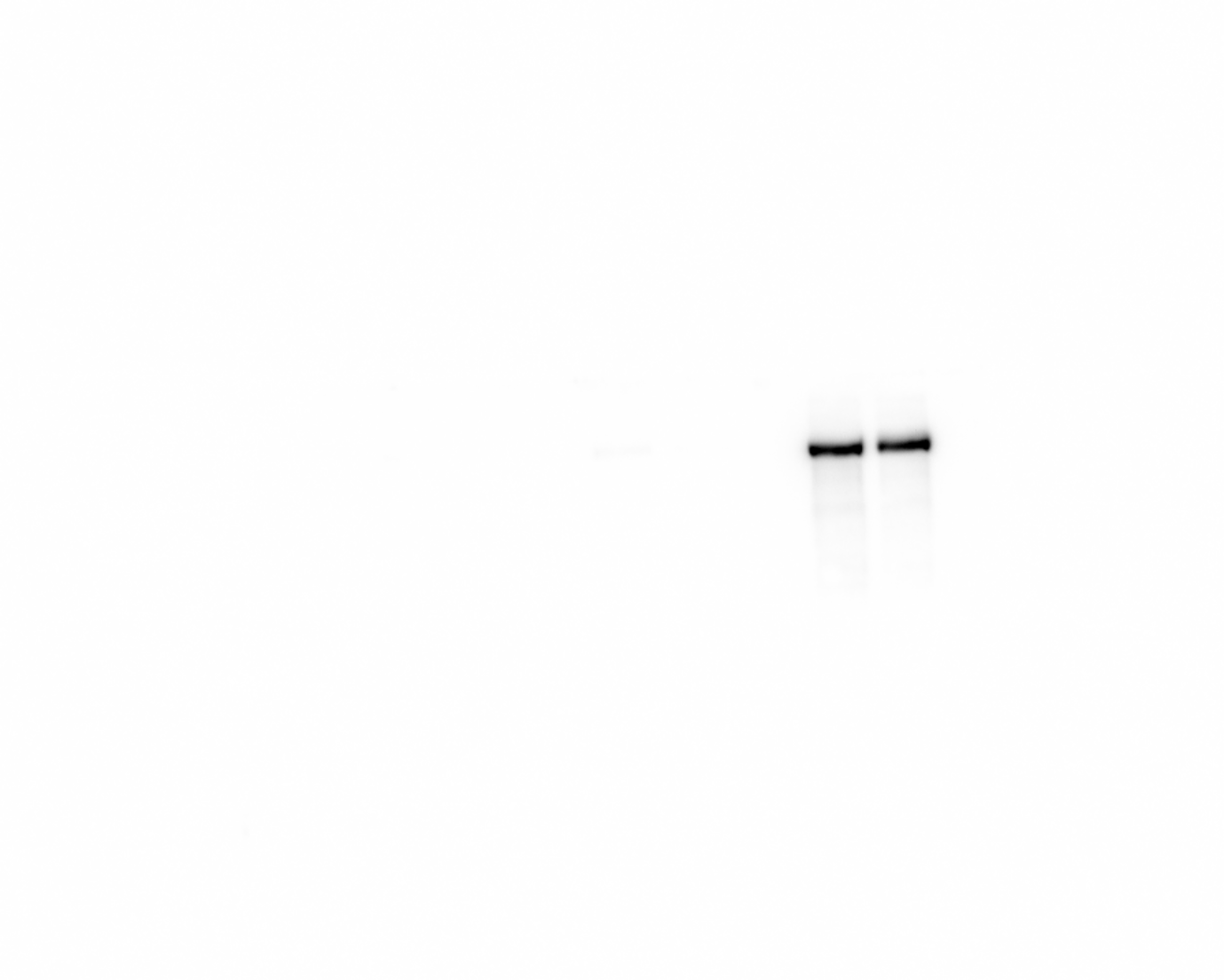

Supplement: Figure 5—source data 1. — Including uncropped Western blot images and raw statistics. [file elife-76436-fig5-data1.zip › Figure 5-Source Data 1/Figure 5K full raw unedited/Input-IB-GFP.tif]

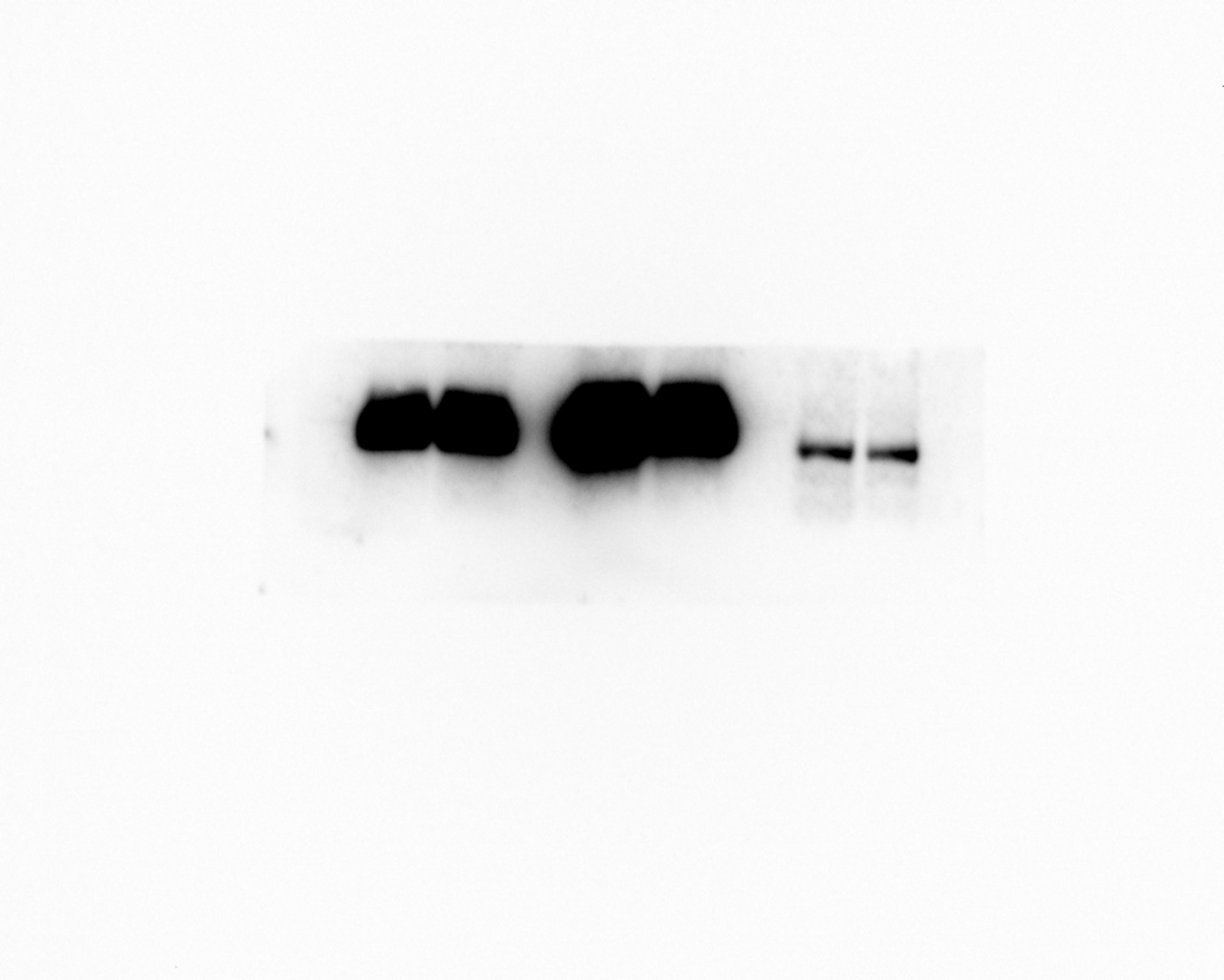

Supplement: Figure 5—source data 1. — Including uncropped Western blot images and raw statistics. [file elife-76436-fig5-data1.zip › Figure 5-Source Data 1/Figure 5K full raw unedited/Input-IB-NCK-1.tif]

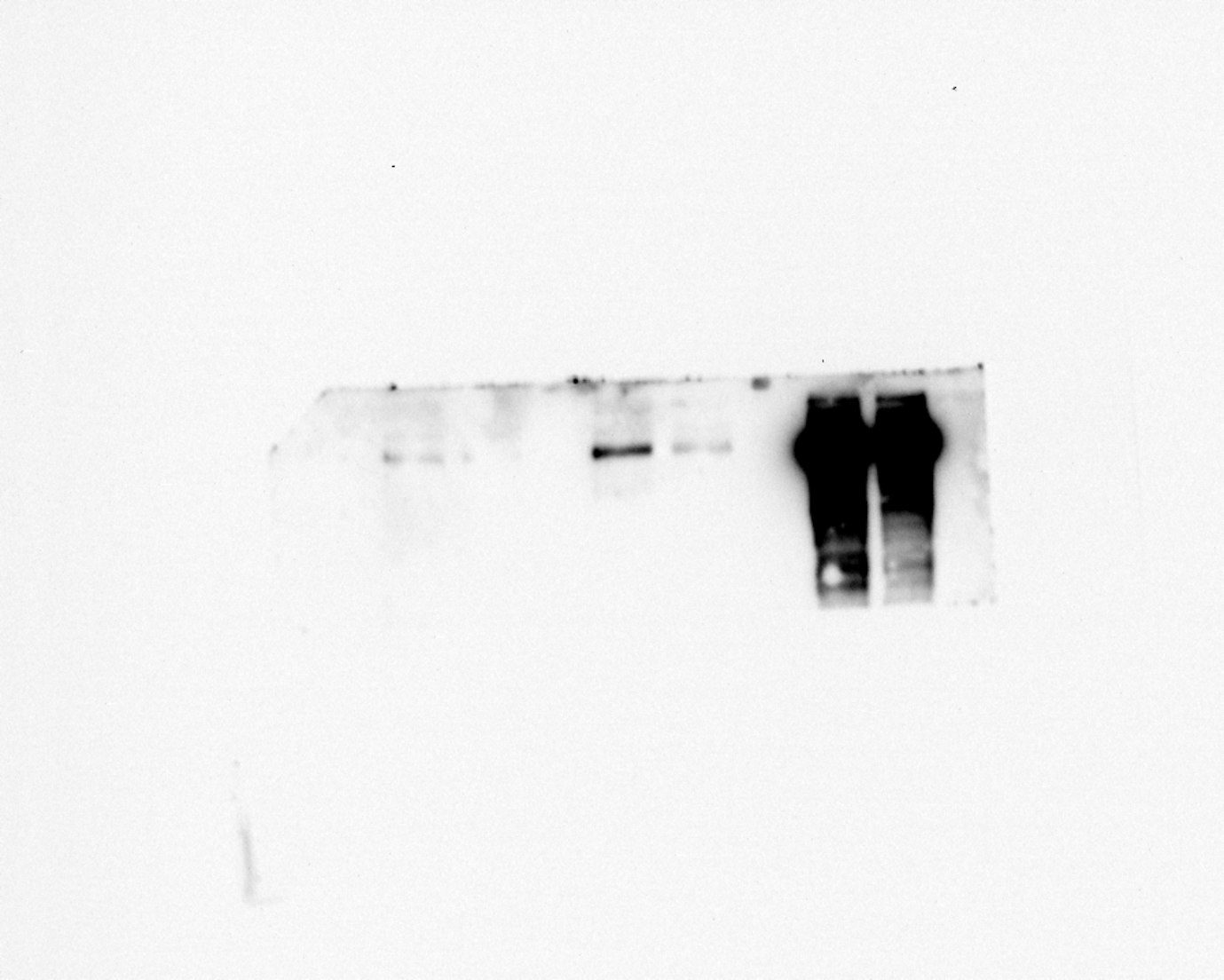

Supplement: Figure 5—source data 1. — Including uncropped Western blot images and raw statistics. [file elife-76436-fig5-data1.zip › Figure 5-Source Data 1/Figure 5K full raw unedited/IP-IB-GFP.tif]

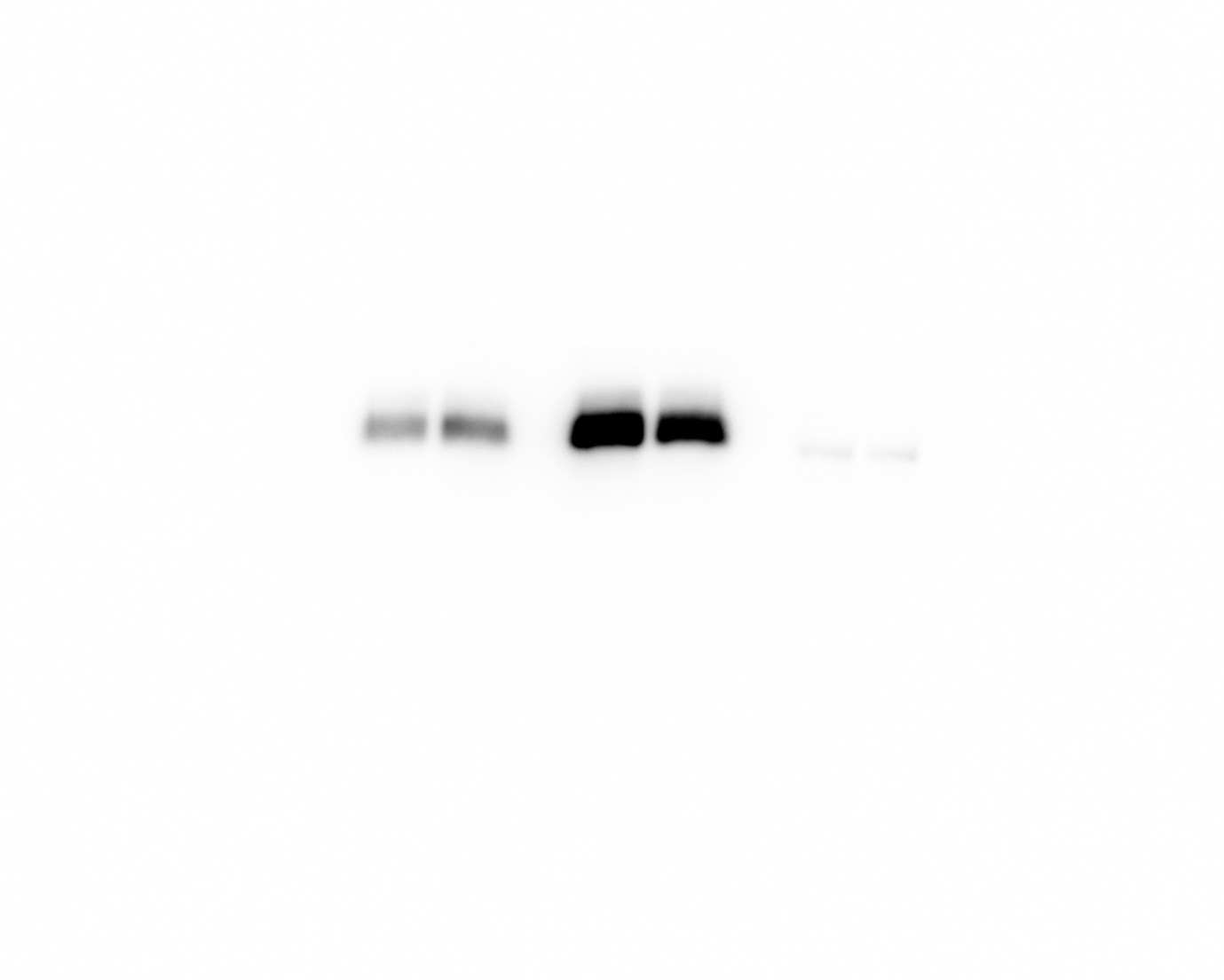

Supplement: Figure 5—source data 1. — Including uncropped Western blot images and raw statistics. [file elife-76436-fig5-data1.zip › Figure 5-Source Data 1/Figure 5K full raw unedited/IP-IB-NCK-1.tif]

Figure 5-figure supplement 1B


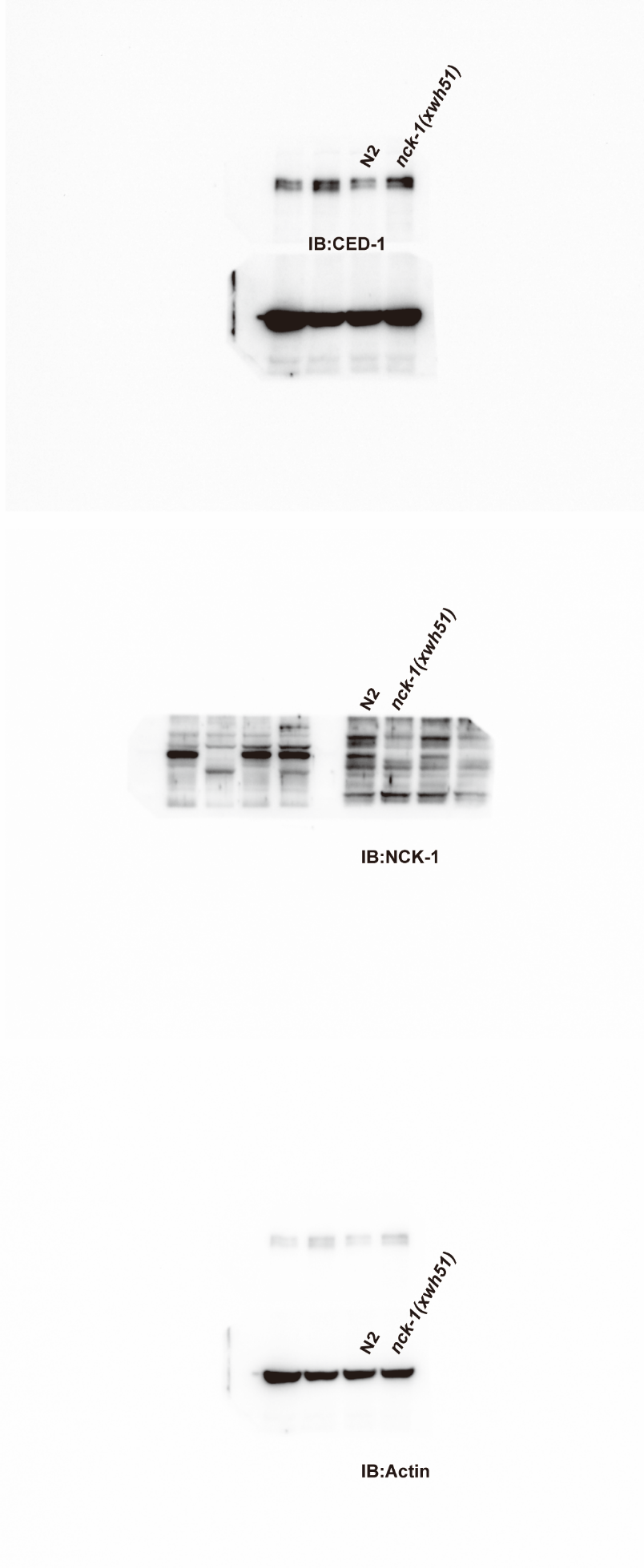


Figure 5-figure supplement 1D


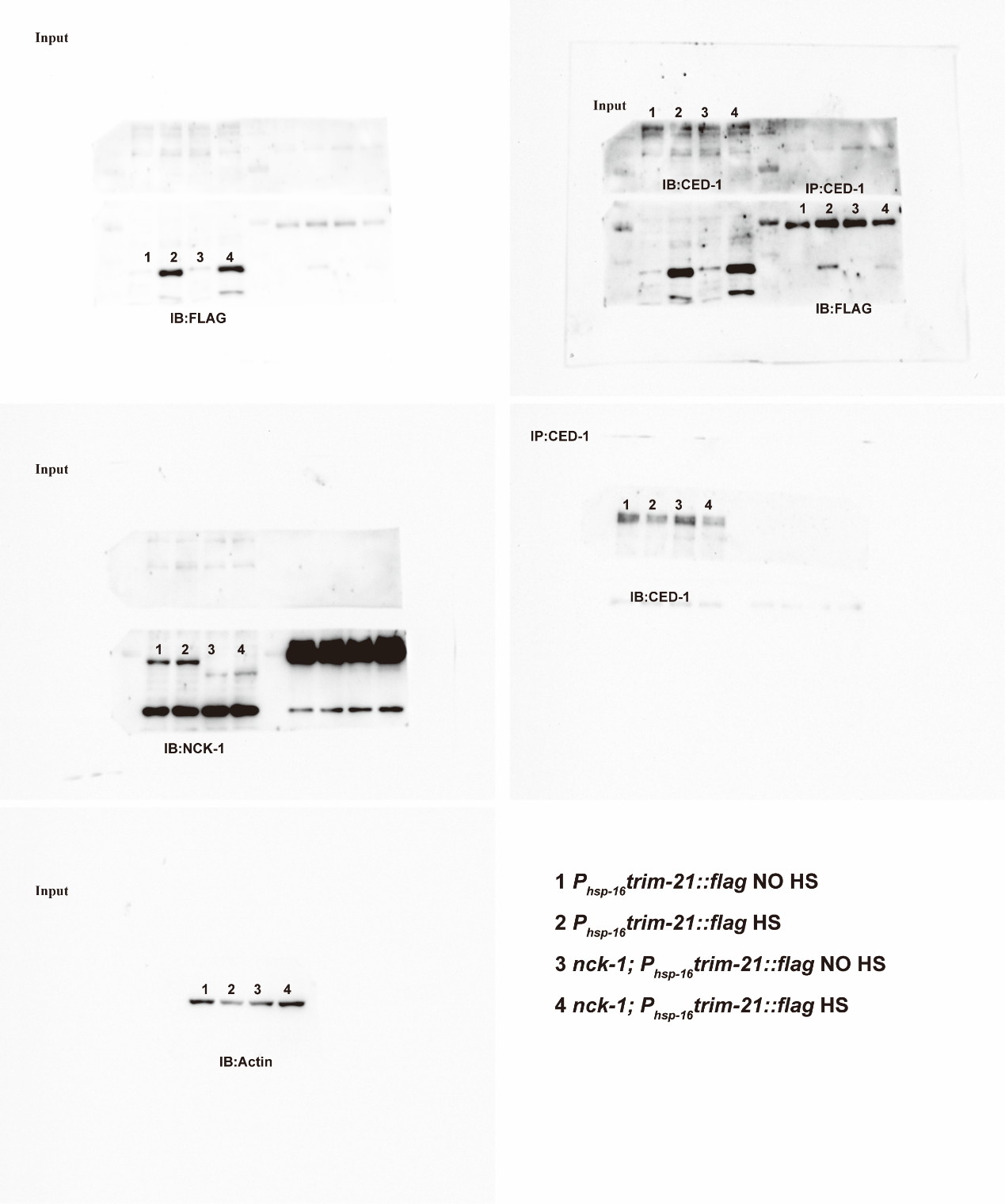


Figure 5-figure supplement 1F


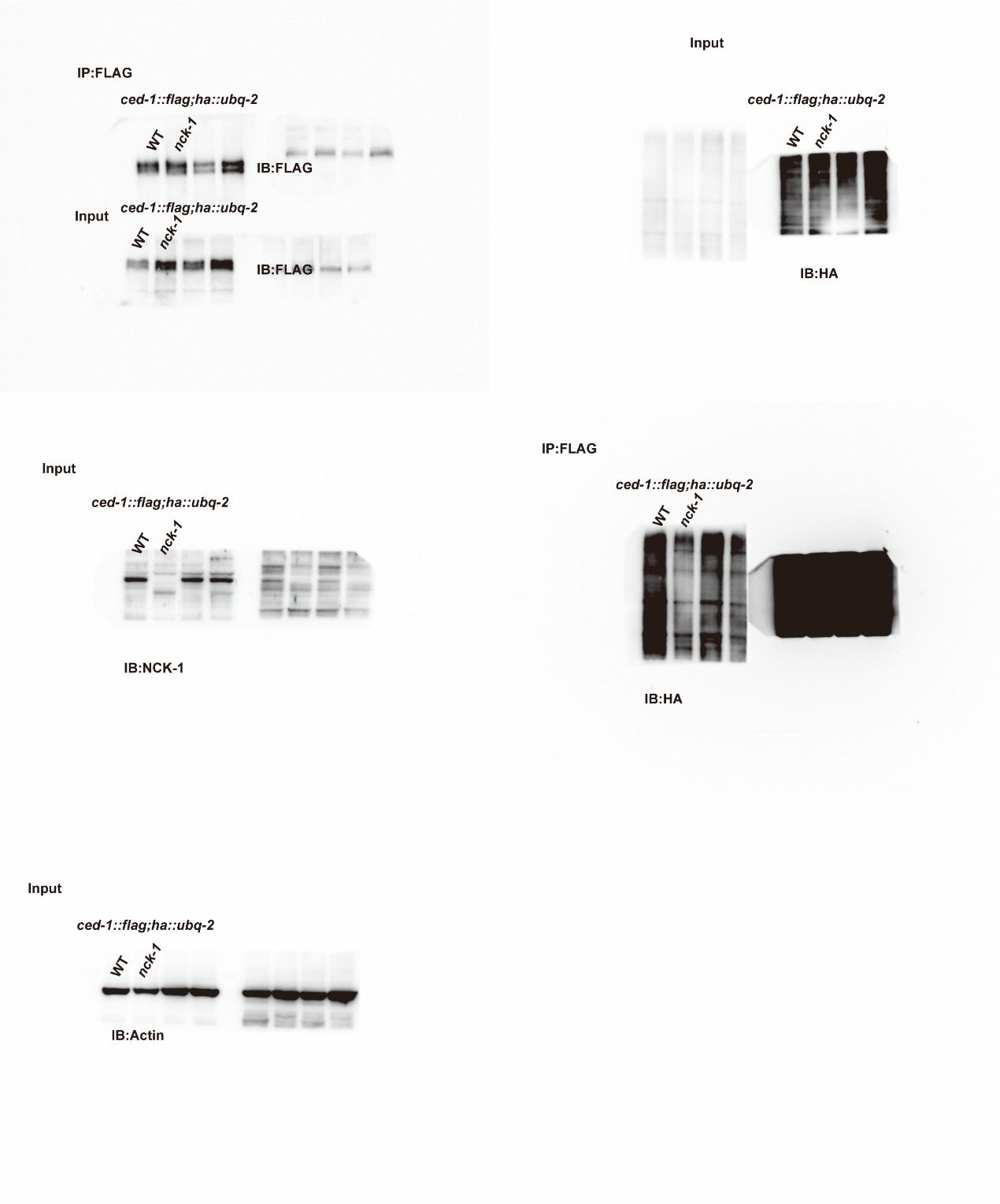

Supplement: Figure 5—figure supplement 1—source data 1. — Including uncropped Western blot images and raw statistics. [file elife-76436-fig5-figsupp1-data1.zip › Figure 5-figure supplement 1-Source Data 1/Figure 5-figure supplement 1 uncroppped blot with relevant bands.docx]

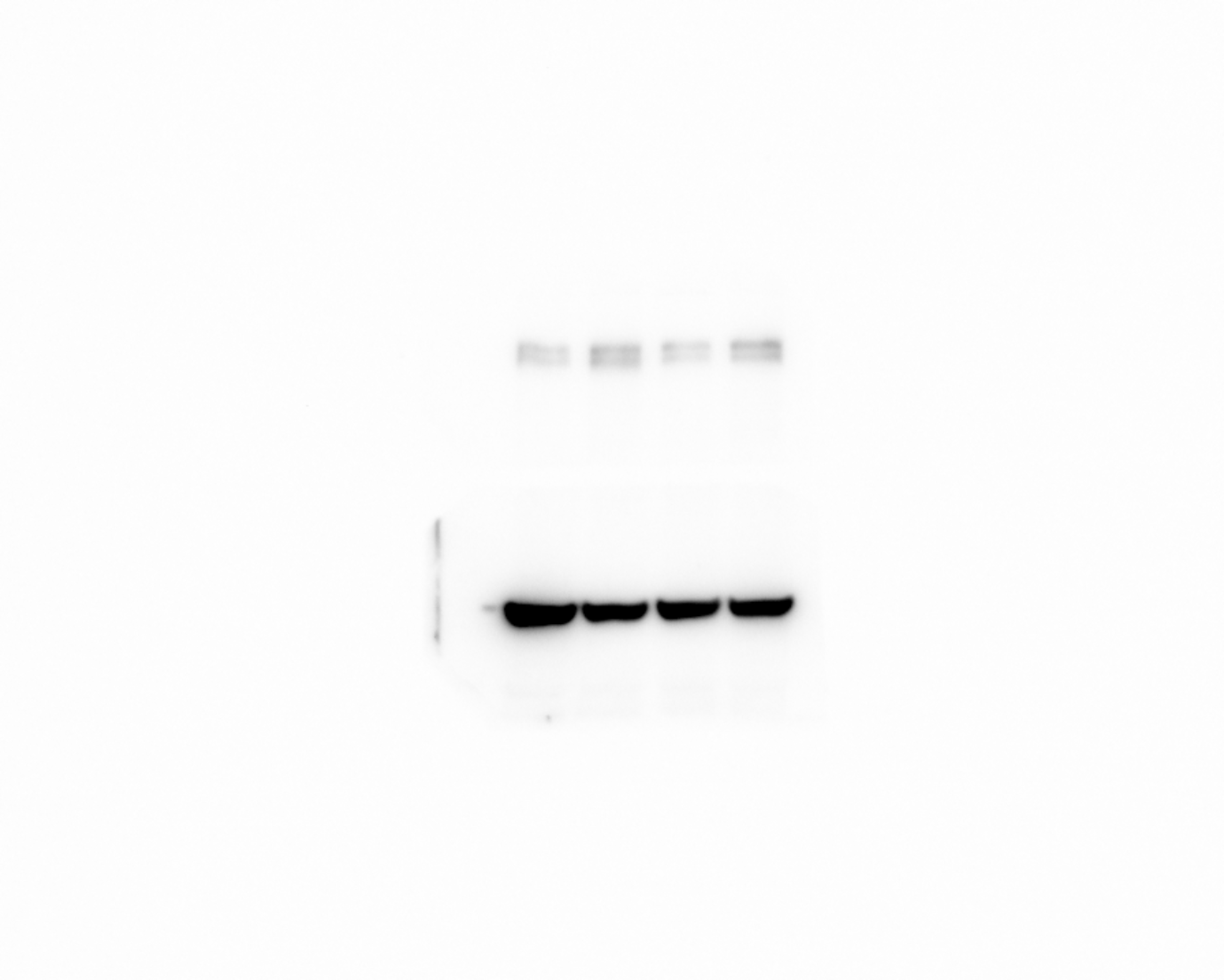

Supplement: Figure 5—figure supplement 1—source data 1. — Including uncropped Western blot images and raw statistics. [file elife-76436-fig5-figsupp1-data1.zip › Figure 5-figure supplement 1-Source Data 1/Figure 5-figure supplement 1B full raw unedited/IB-Actin.tif]

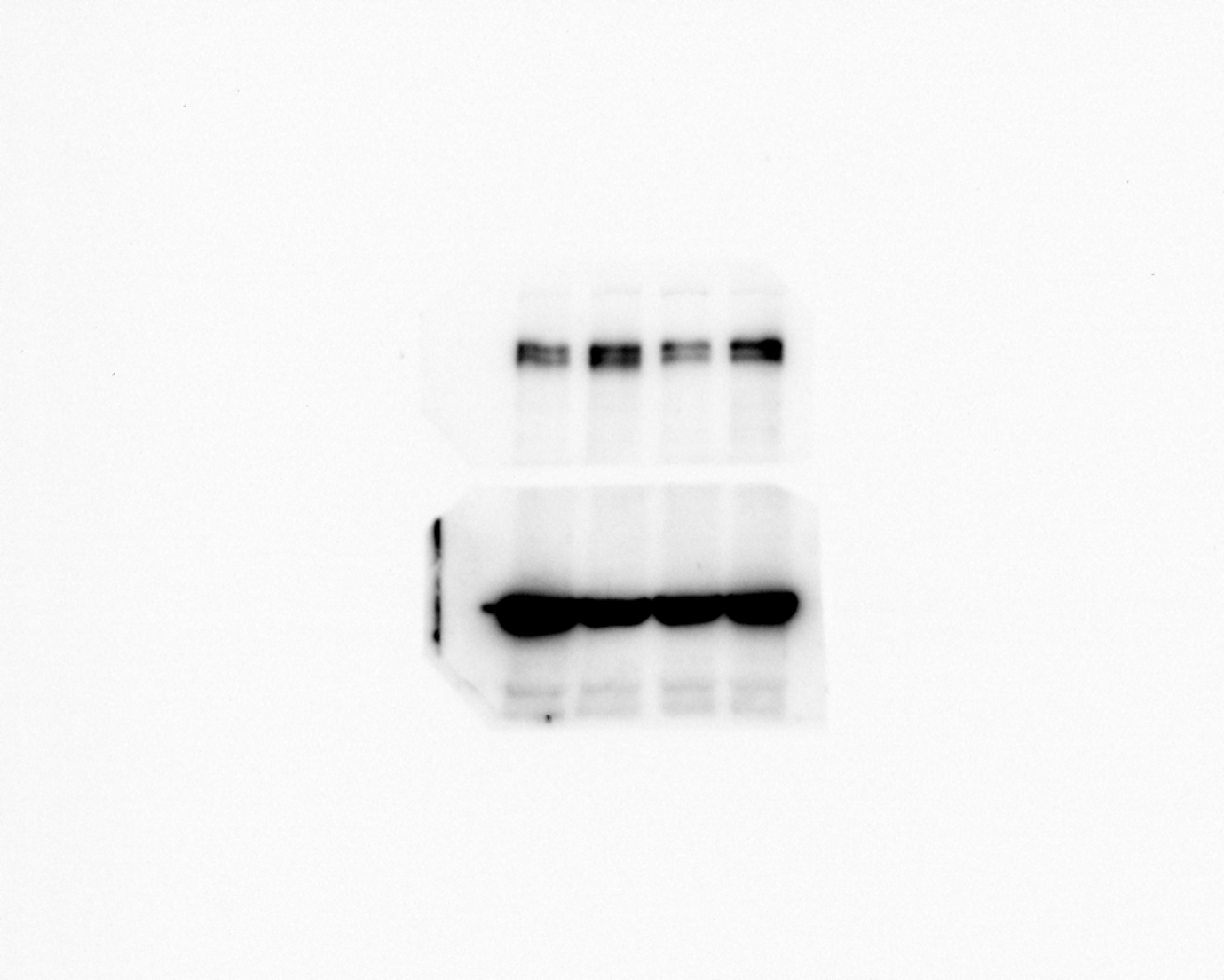

Supplement: Figure 5—figure supplement 1—source data 1. — Including uncropped Western blot images and raw statistics. [file elife-76436-fig5-figsupp1-data1.zip › Figure 5-figure supplement 1-Source Data 1/Figure 5-figure supplement 1B full raw unedited/IB-CED-1.tif]

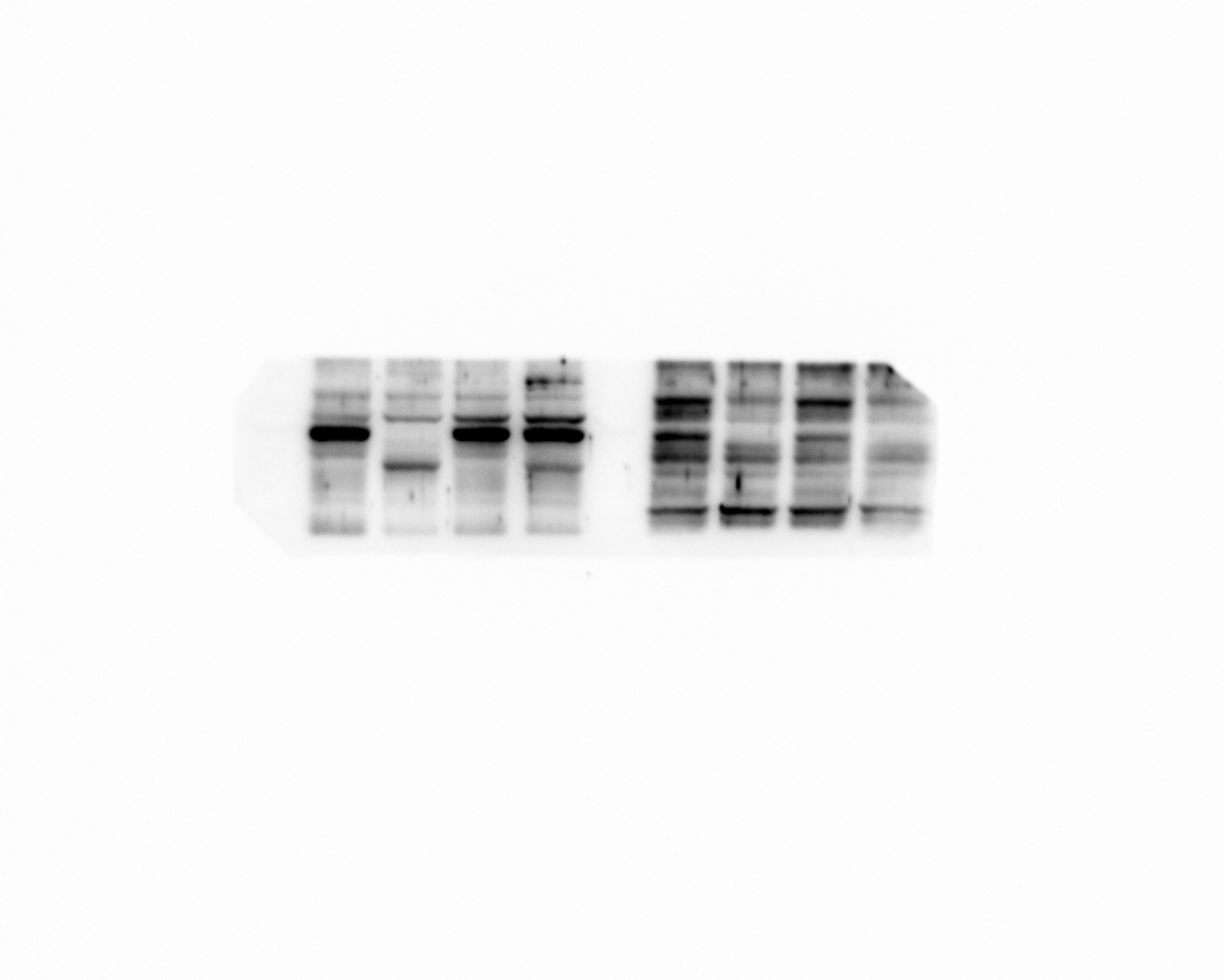

Supplement: Figure 5—figure supplement 1—source data 1. — Including uncropped Western blot images and raw statistics. [file elife-76436-fig5-figsupp1-data1.zip › Figure 5-figure supplement 1-Source Data 1/Figure 5-figure supplement 1B full raw unedited/IB-NCK-1.tif]

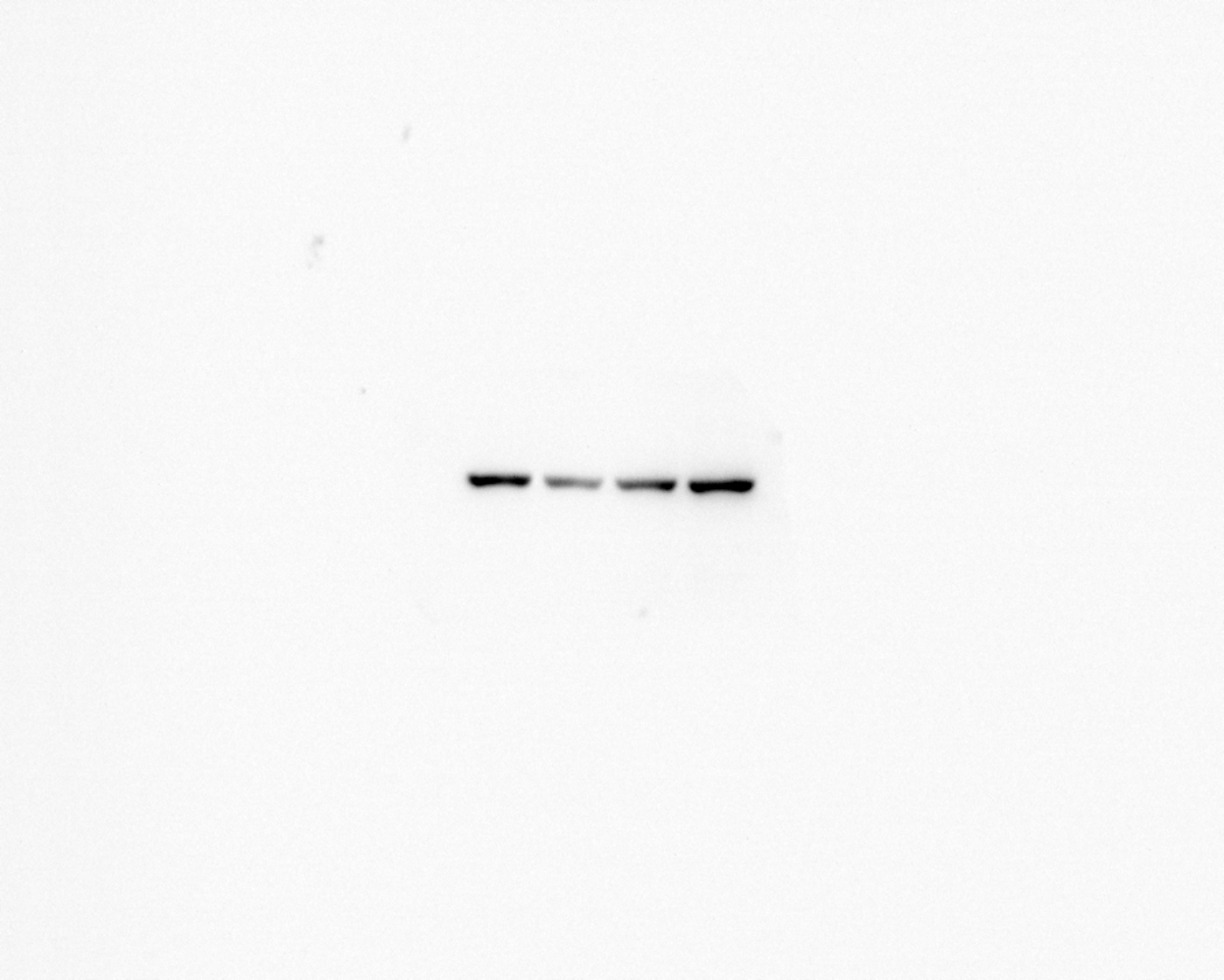

Supplement: Figure 5—figure supplement 1—source data 1. — Including uncropped Western blot images and raw statistics. [file elife-76436-fig5-figsupp1-data1.zip › Figure 5-figure supplement 1-Source Data 1/Figure 5-figure supplement 1D full raw unedited/Input-IB-Actin.tif]

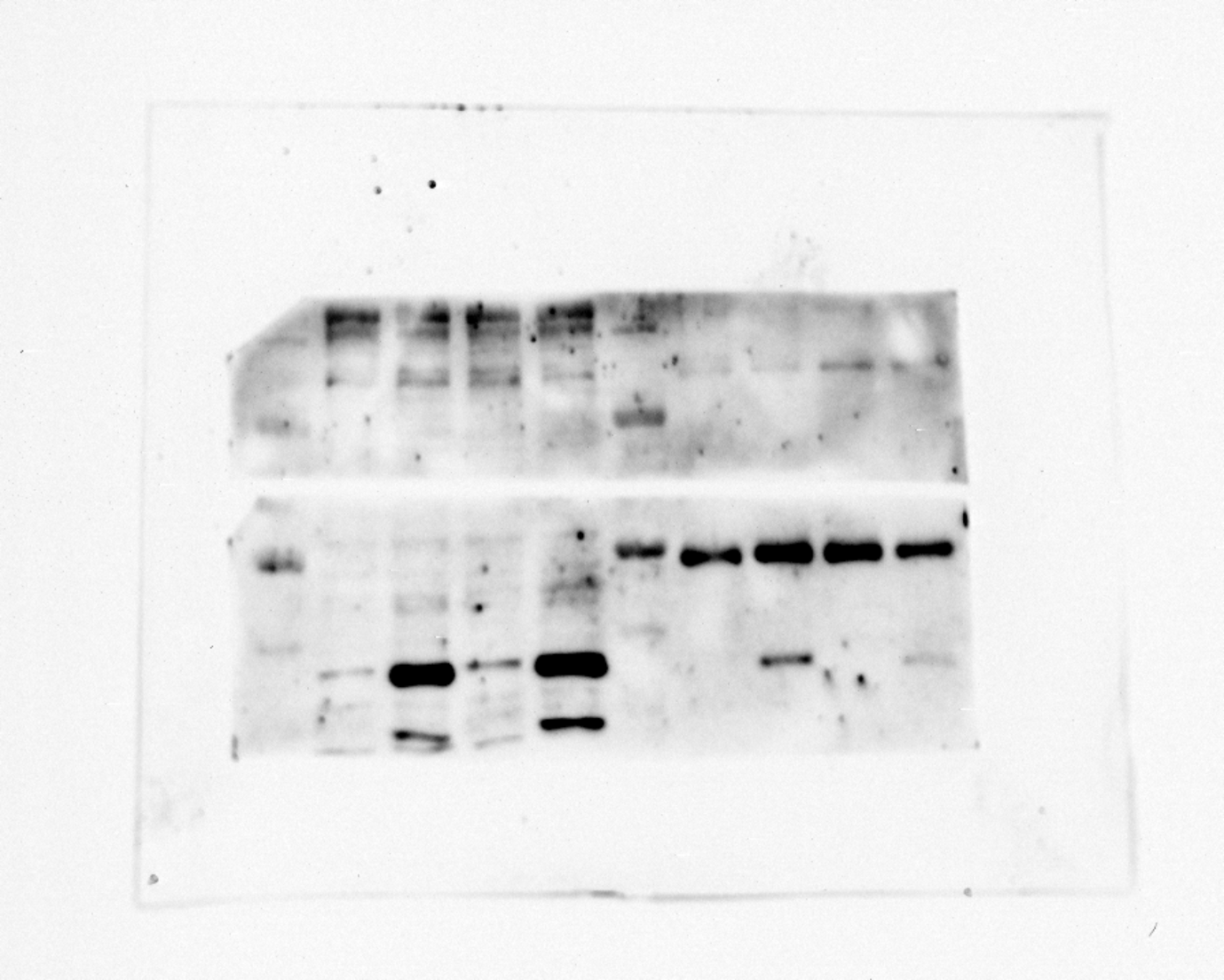

Supplement: Figure 5—figure supplement 1—source data 1. — Including uncropped Western blot images and raw statistics. [file elife-76436-fig5-figsupp1-data1.zip › Figure 5-figure supplement 1-Source Data 1/Figure 5-figure supplement 1D full raw unedited/Input-IB-CED-1-IP-IB-FLAG.tif]

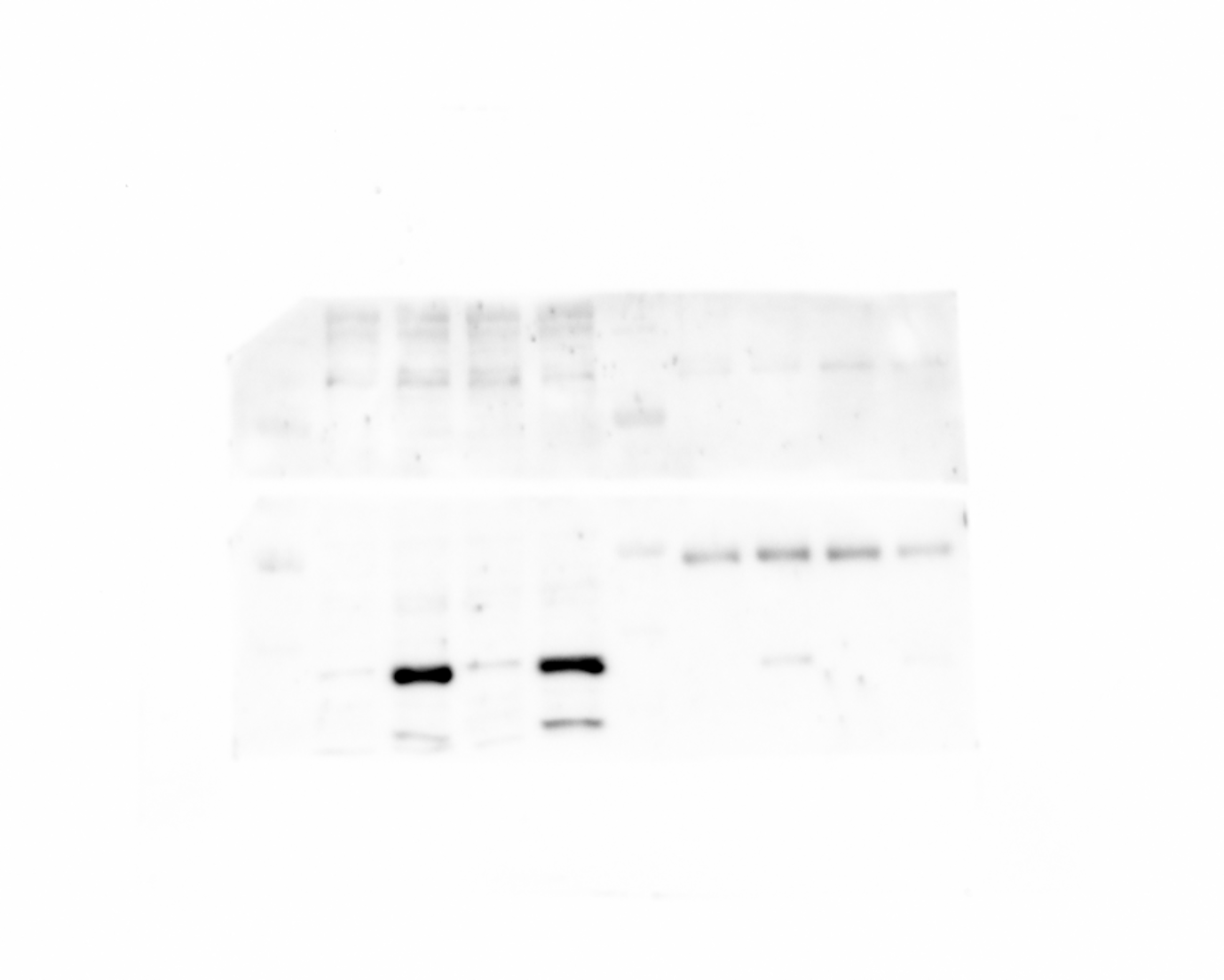

Supplement: Figure 5—figure supplement 1—source data 1. — Including uncropped Western blot images and raw statistics. [file elife-76436-fig5-figsupp1-data1.zip › Figure 5-figure supplement 1-Source Data 1/Figure 5-figure supplement 1D full raw unedited/Input-IB-FLAG.tif]

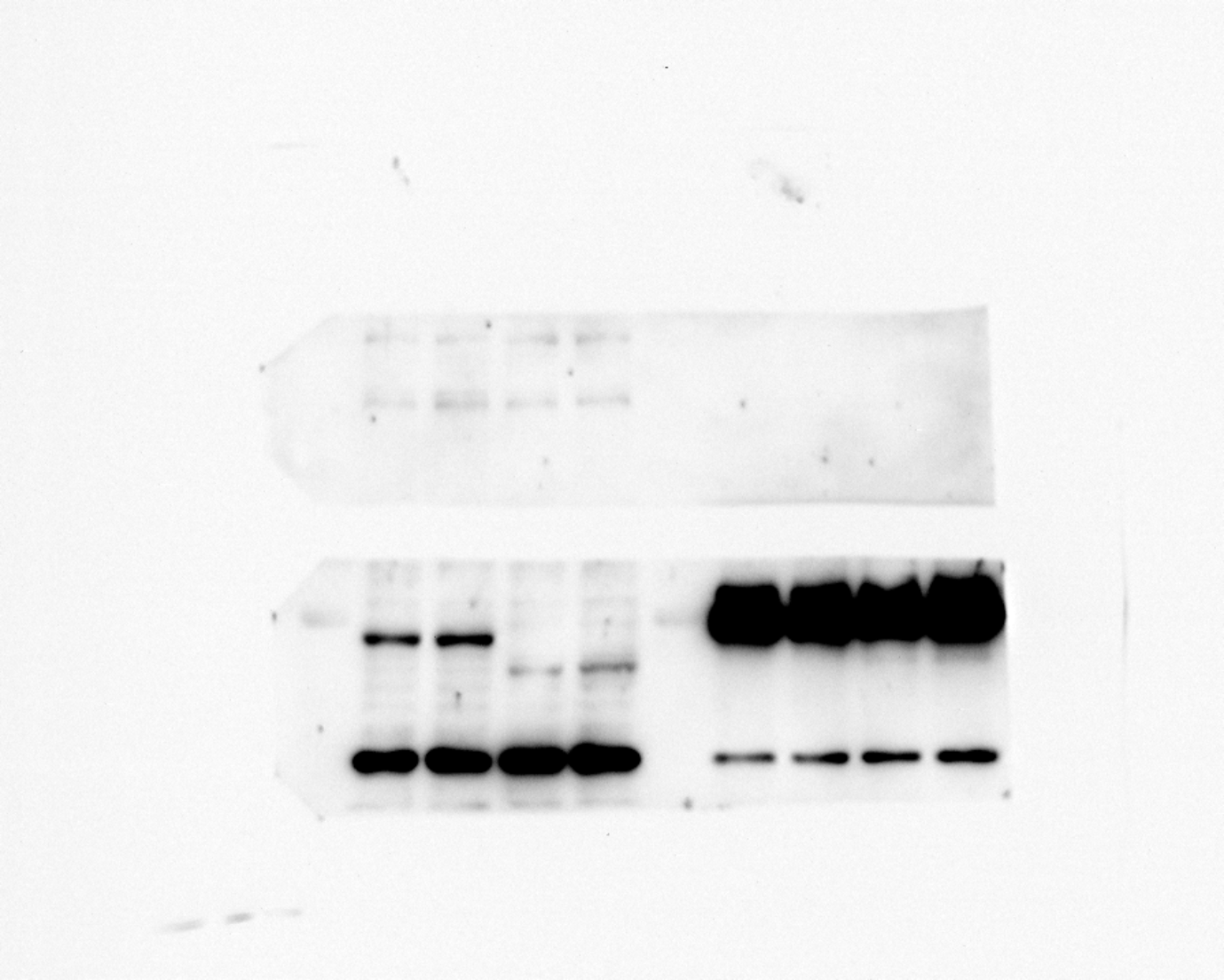

Supplement: Figure 5—figure supplement 1—source data 1. — Including uncropped Western blot images and raw statistics. [file elife-76436-fig5-figsupp1-data1.zip › Figure 5-figure supplement 1-Source Data 1/Figure 5-figure supplement 1D full raw unedited/Input-IB-NCK-1.tif]

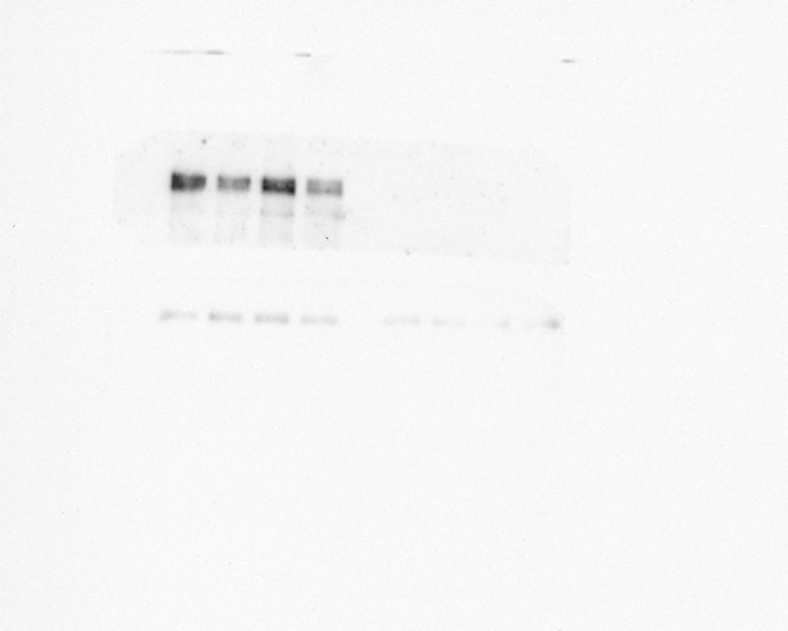

Supplement: Figure 5—figure supplement 1—source data 1. — Including uncropped Western blot images and raw statistics. [file elife-76436-fig5-figsupp1-data1.zip › Figure 5-figure supplement 1-Source Data 1/Figure 5-figure supplement 1D full raw unedited/IP-IB-CED-1.tif]

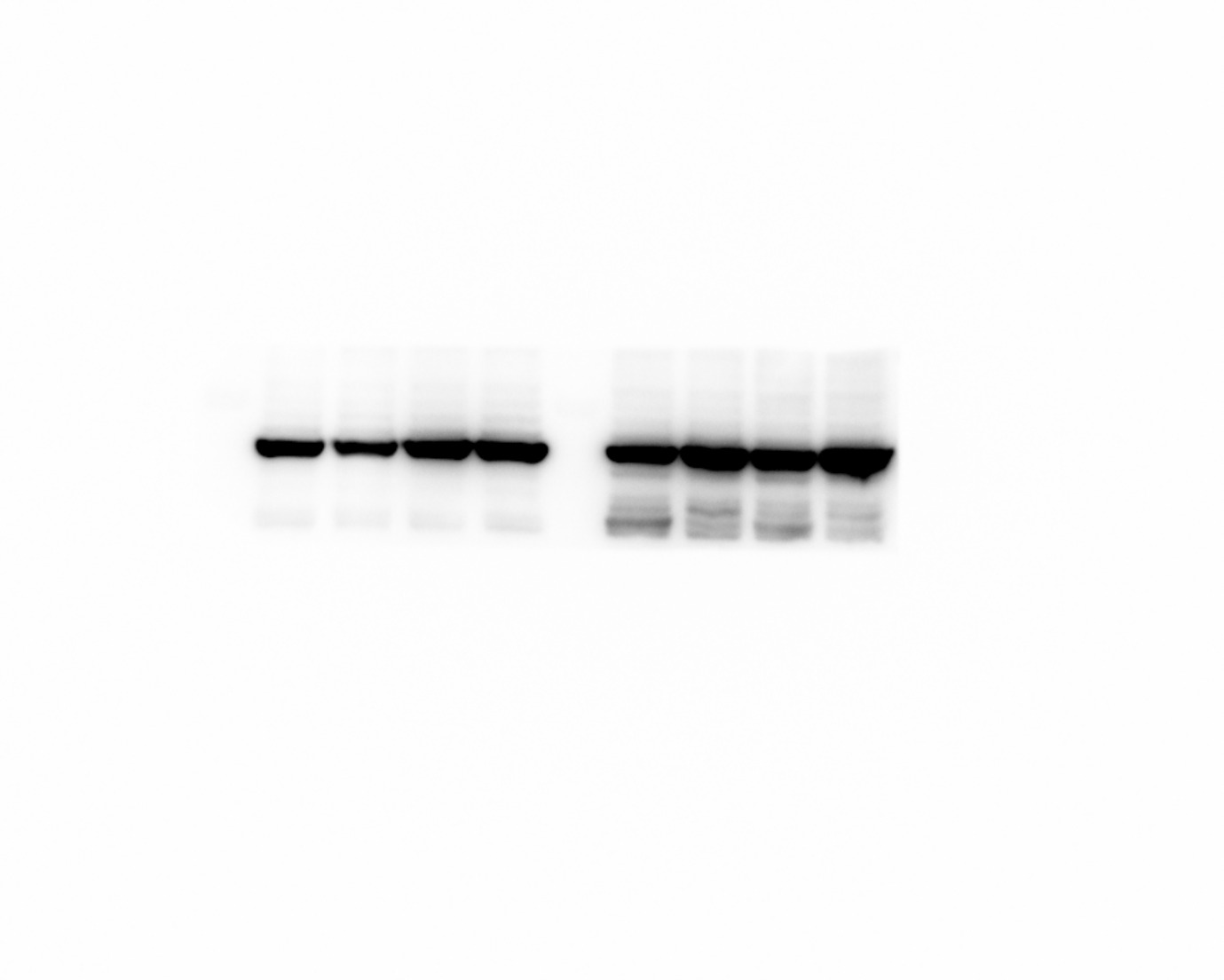

Supplement: Figure 5—figure supplement 1—source data 1. — Including uncropped Western blot images and raw statistics. [file elife-76436-fig5-figsupp1-data1.zip › Figure 5-figure supplement 1-Source Data 1/Figure 5-figure supplement 1F full raw unedited/Input-IB-Actin.tif]

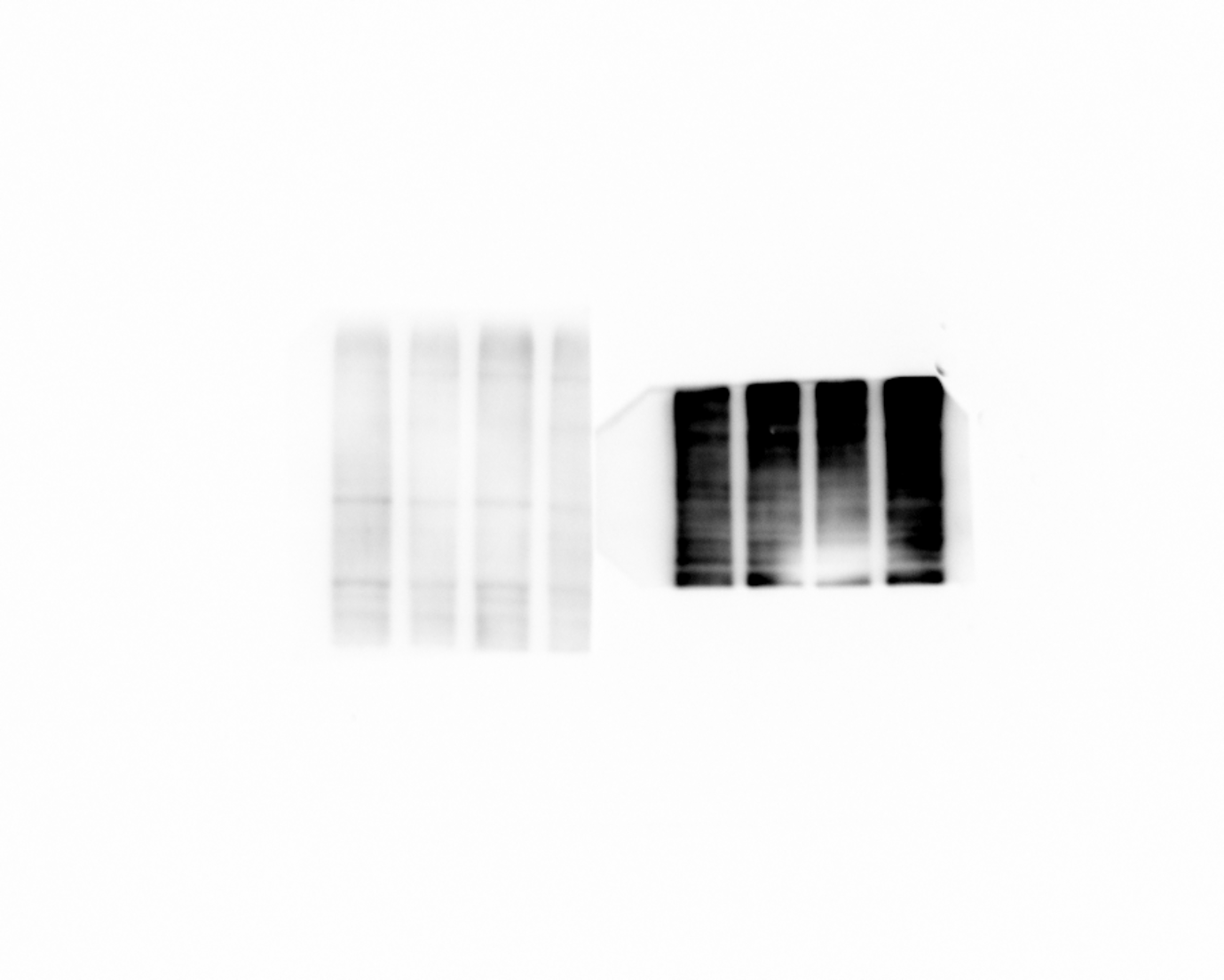

Supplement: Figure 5—figure supplement 1—source data 1. — Including uncropped Western blot images and raw statistics. [file elife-76436-fig5-figsupp1-data1.zip › Figure 5-figure supplement 1-Source Data 1/Figure 5-figure supplement 1F full raw unedited/Input-IB-HA.tif]

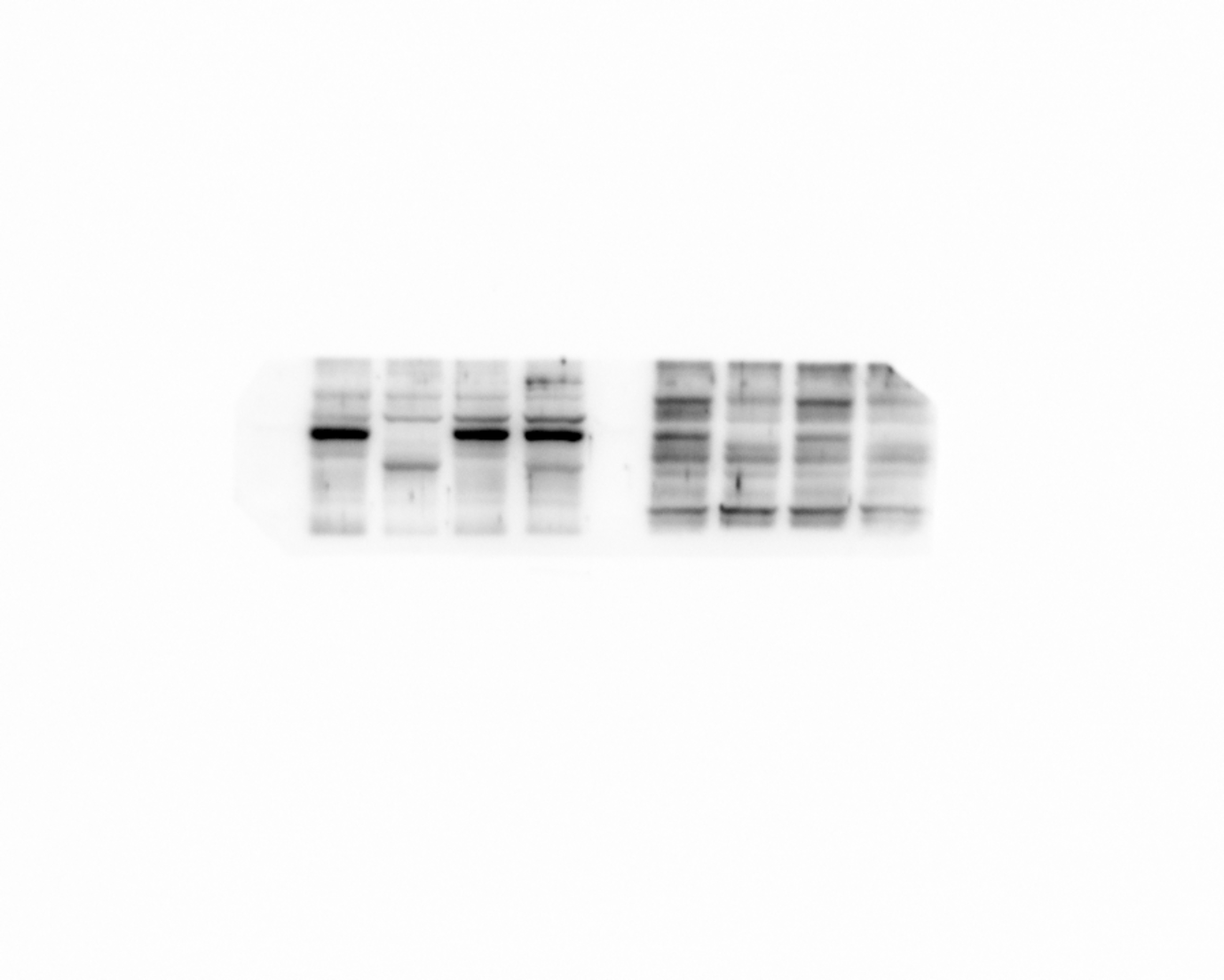

Supplement: Figure 5—figure supplement 1—source data 1. — Including uncropped Western blot images and raw statistics. [file elife-76436-fig5-figsupp1-data1.zip › Figure 5-figure supplement 1-Source Data 1/Figure 5-figure supplement 1F full raw unedited/Input-IB-NCK-1.tif]

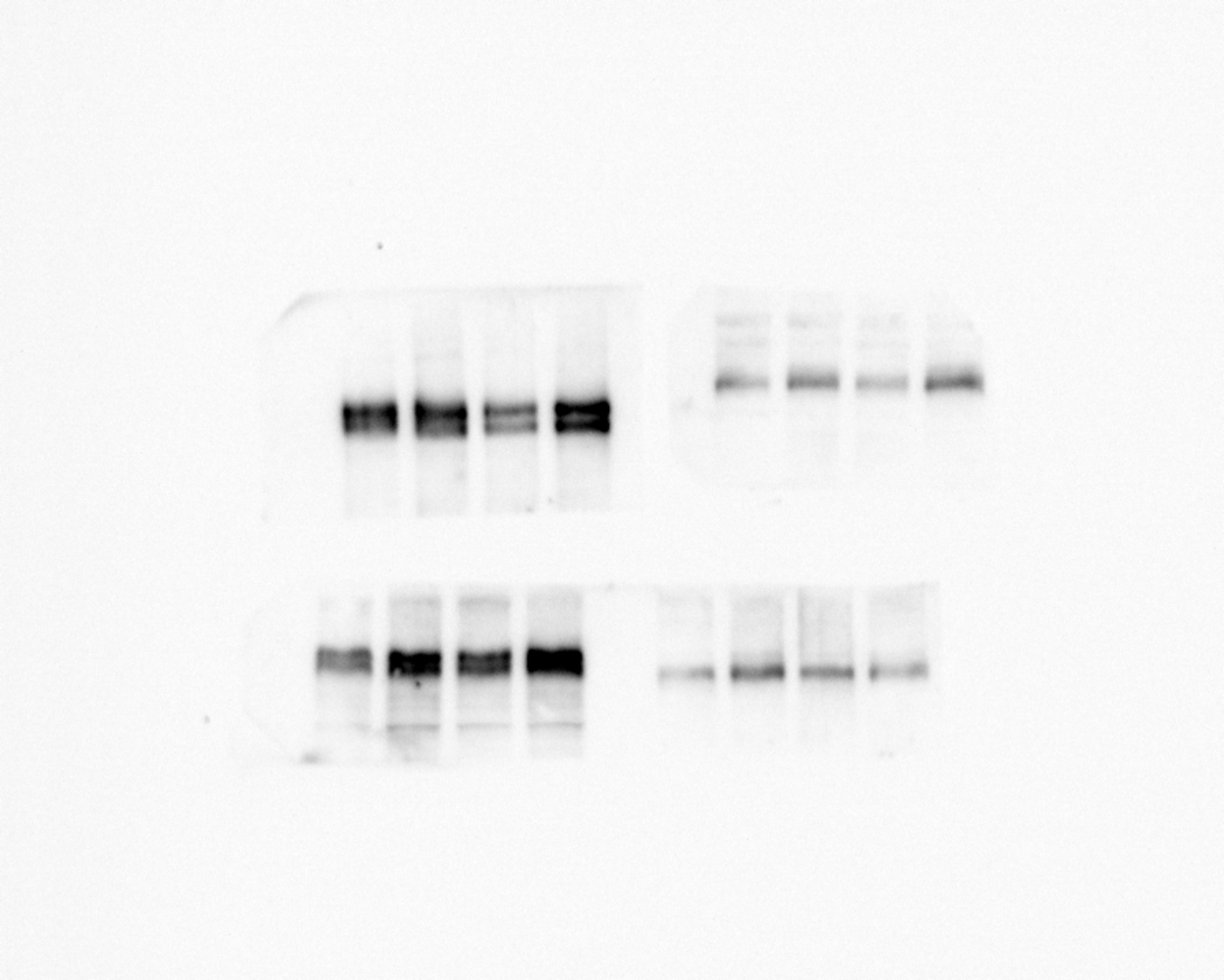

Supplement: Figure 5—figure supplement 1—source data 1. — Including uncropped Western blot images and raw statistics. [file elife-76436-fig5-figsupp1-data1.zip › Figure 5-figure supplement 1-Source Data 1/Figure 5-figure supplement 1F full raw unedited/Input-IP-IB-FLAG.tif]

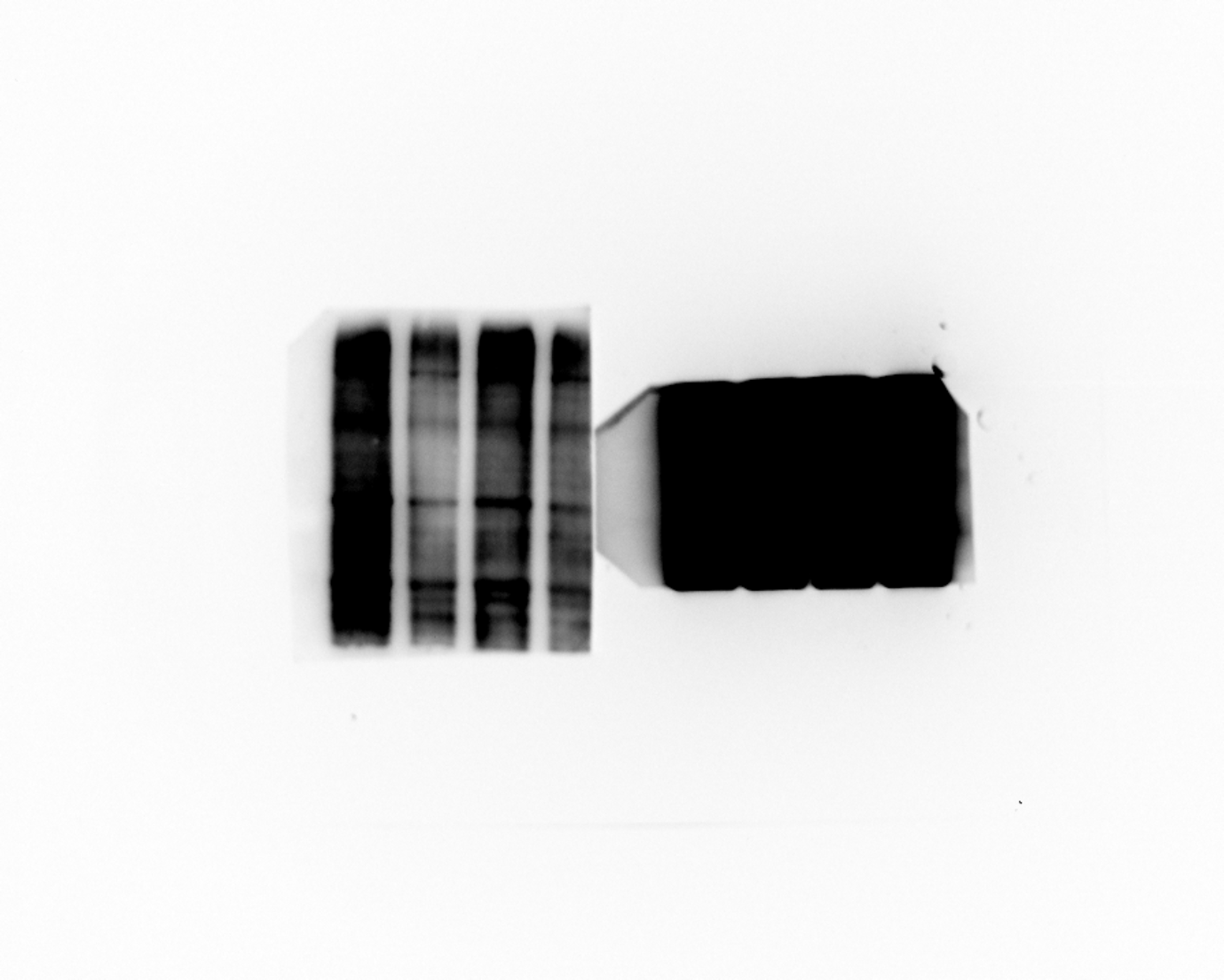

Supplement: Figure 5—figure supplement 1—source data 1. — Including uncropped Western blot images and raw statistics. [file elife-76436-fig5-figsupp1-data1.zip › Figure 5-figure supplement 1-Source Data 1/Figure 5-figure supplement 1F full raw unedited/IP-IB-HA.tif]

Figure 7A


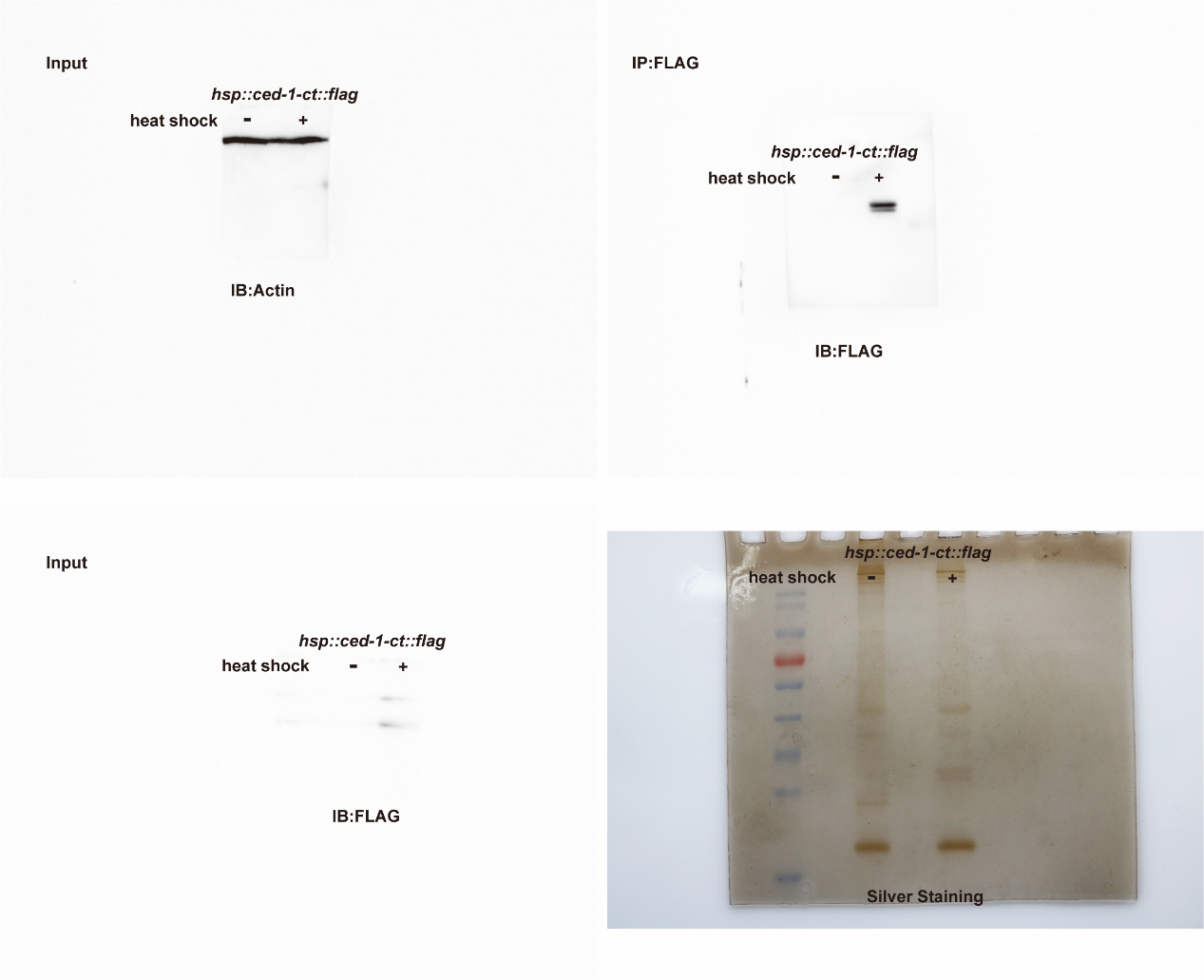


Figure 7C


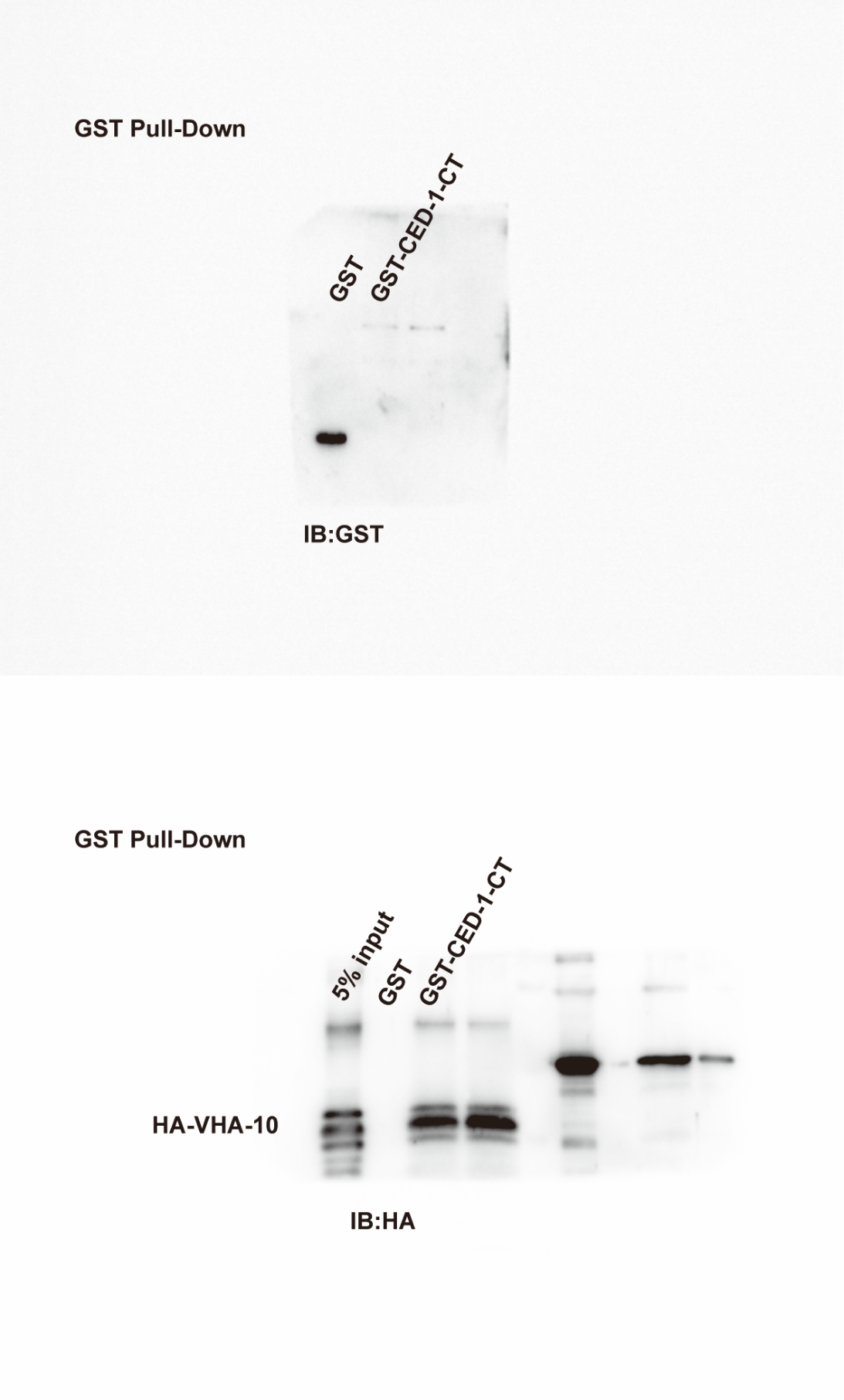


Figure 7D


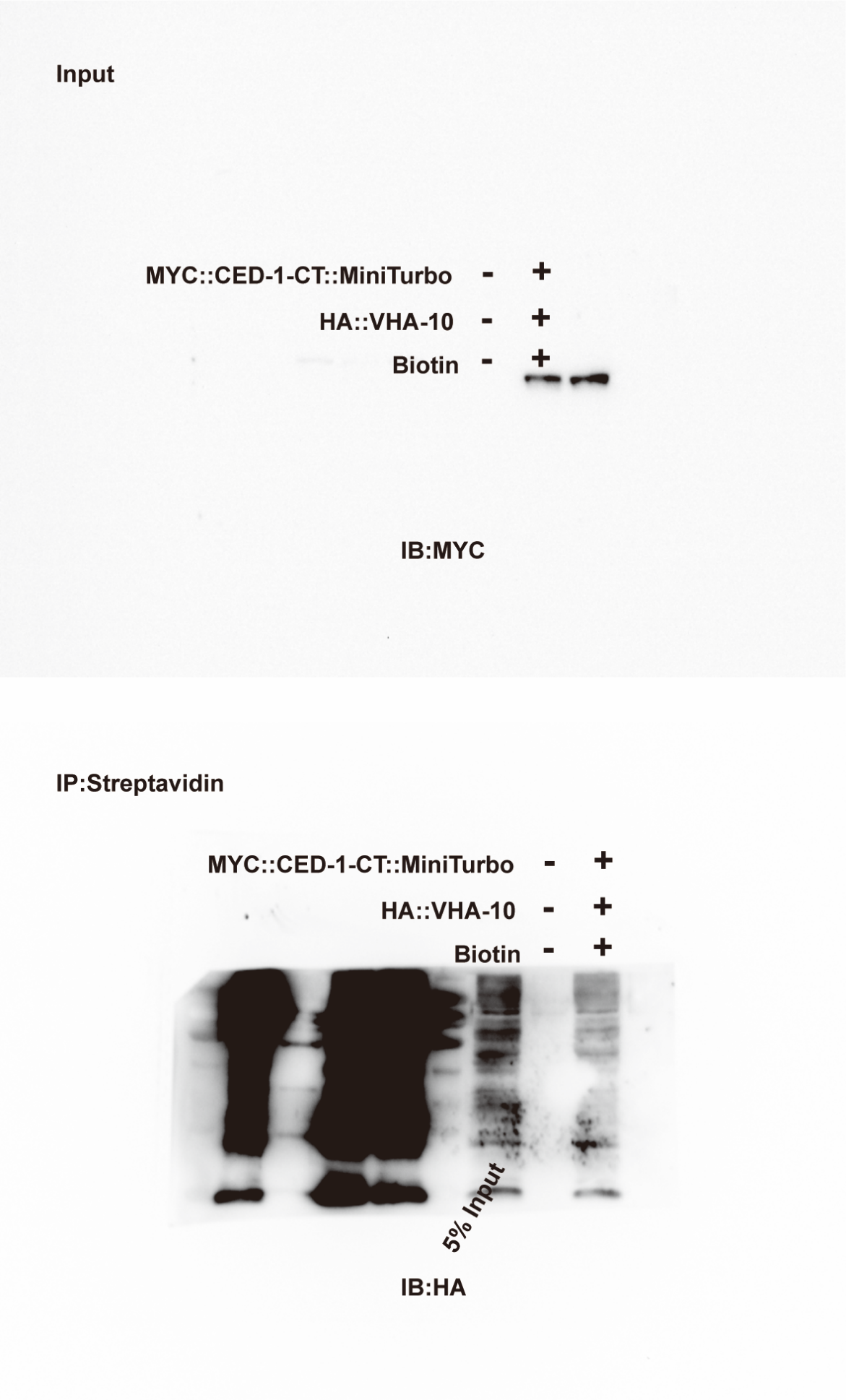

Supplement: Figure 7—source data 1. — Including uncropped Western blot images and raw statistics. [file elife-76436-fig7-data1.zip › Figure 7-Source Data 1/Figure 7 uncroppped blot with relevant bands.docx]

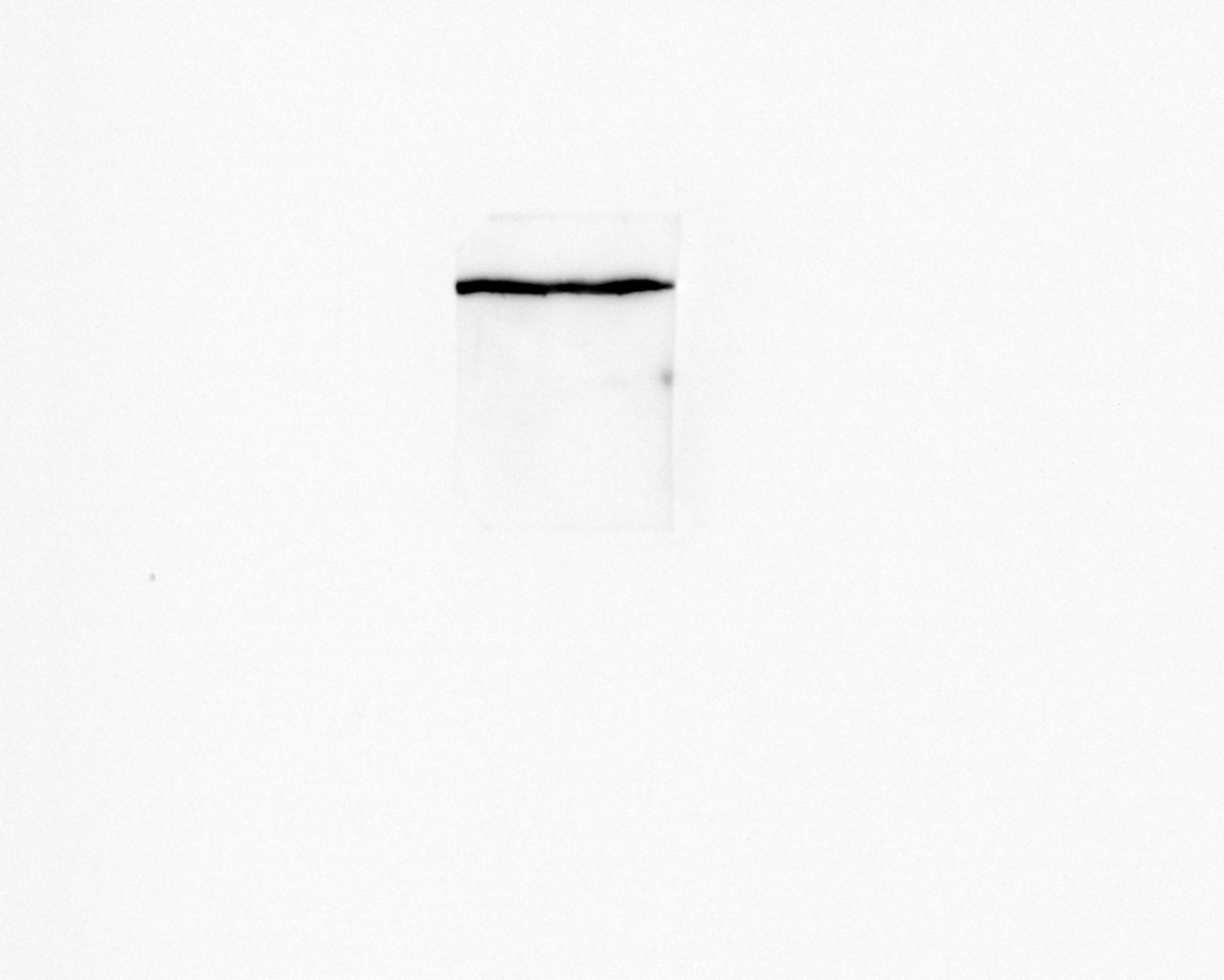

Supplement: Figure 7—source data 1. — Including uncropped Western blot images and raw statistics. [file elife-76436-fig7-data1.zip › Figure 7-Source Data 1/Figure 7A full raw unedited/Input-IB-Actin.tif]

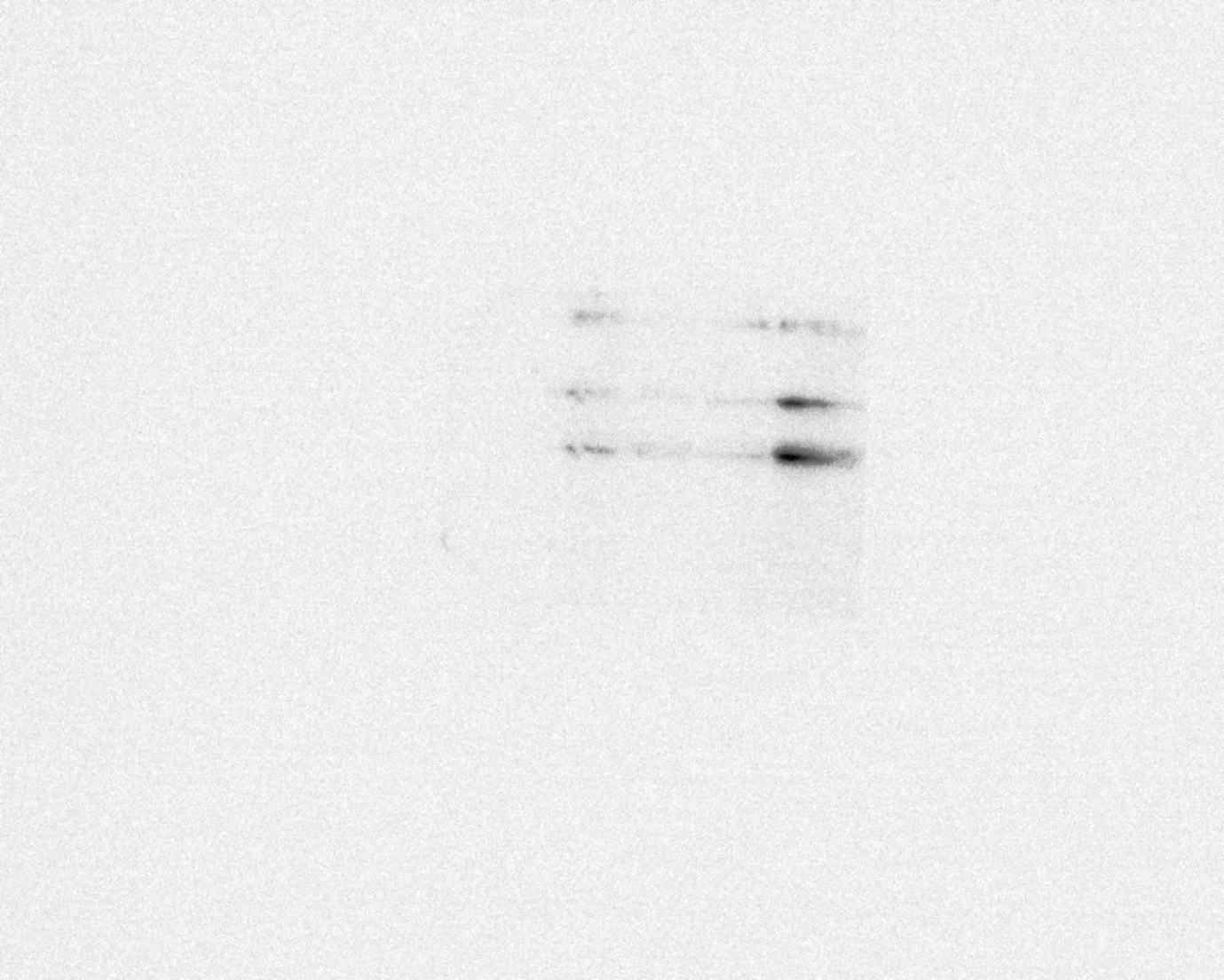

Supplement: Figure 7—source data 1. — Including uncropped Western blot images and raw statistics. [file elife-76436-fig7-data1.zip › Figure 7-Source Data 1/Figure 7A full raw unedited/Input-IB-FLAG.tif]

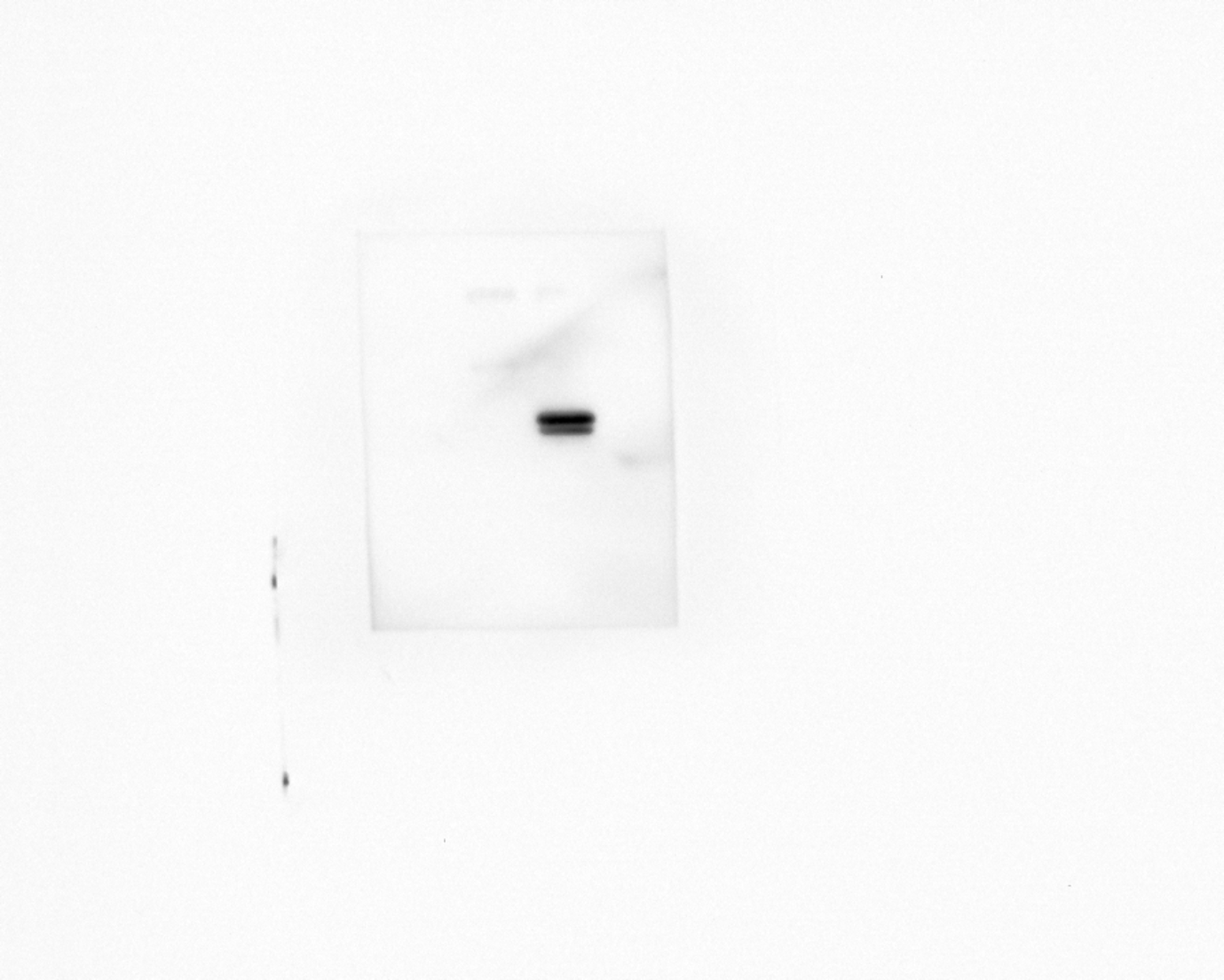

Supplement: Figure 7—source data 1. — Including uncropped Western blot images and raw statistics. [file elife-76436-fig7-data1.zip › Figure 7-Source Data 1/Figure 7A full raw unedited/IP-IB-FLAG.tif]

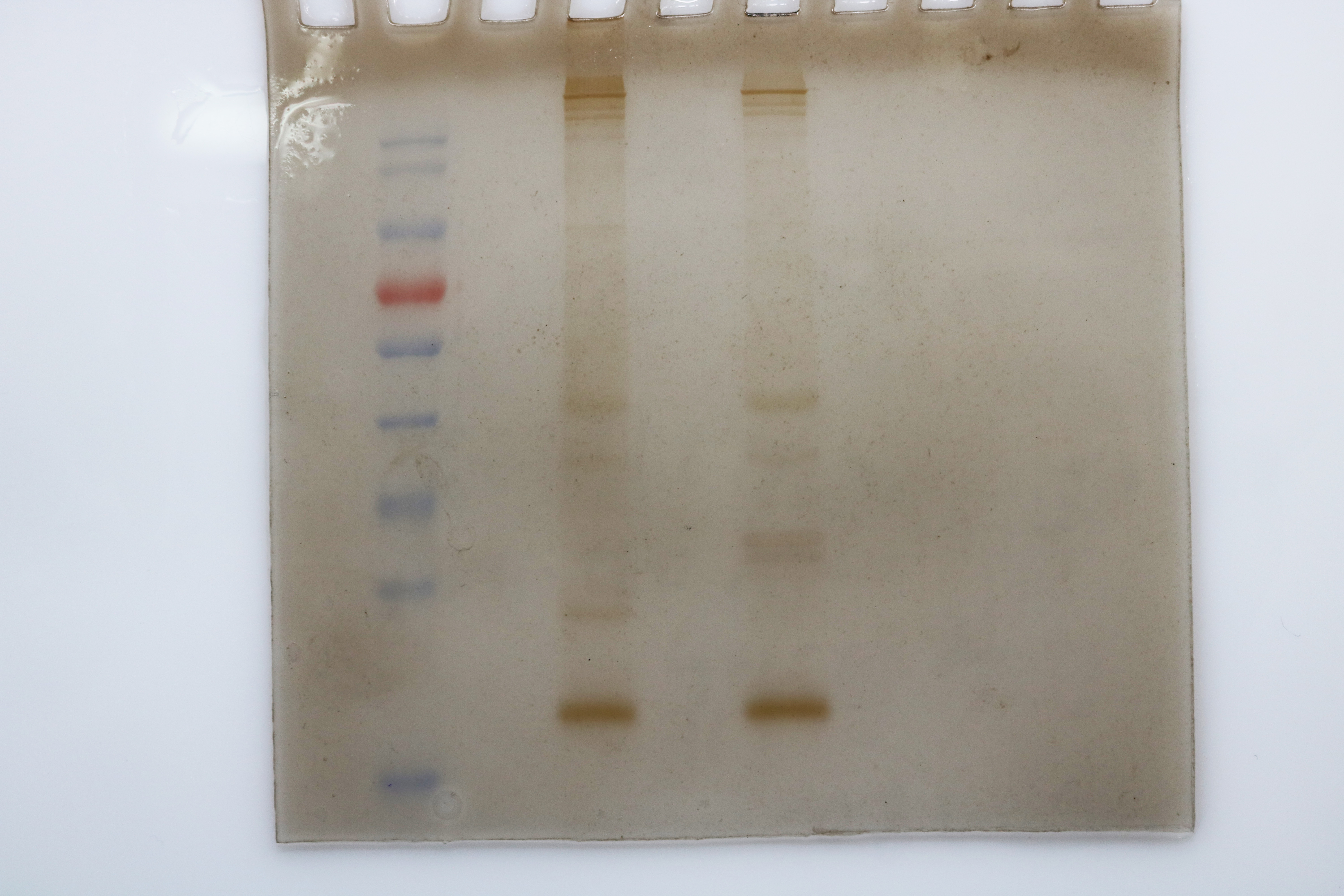

Supplement: Figure 7—source data 1. — Including uncropped Western blot images and raw statistics. [file elife-76436-fig7-data1.zip › Figure 7-Source Data 1/Figure 7A full raw unedited/Silver Staining.JPG]

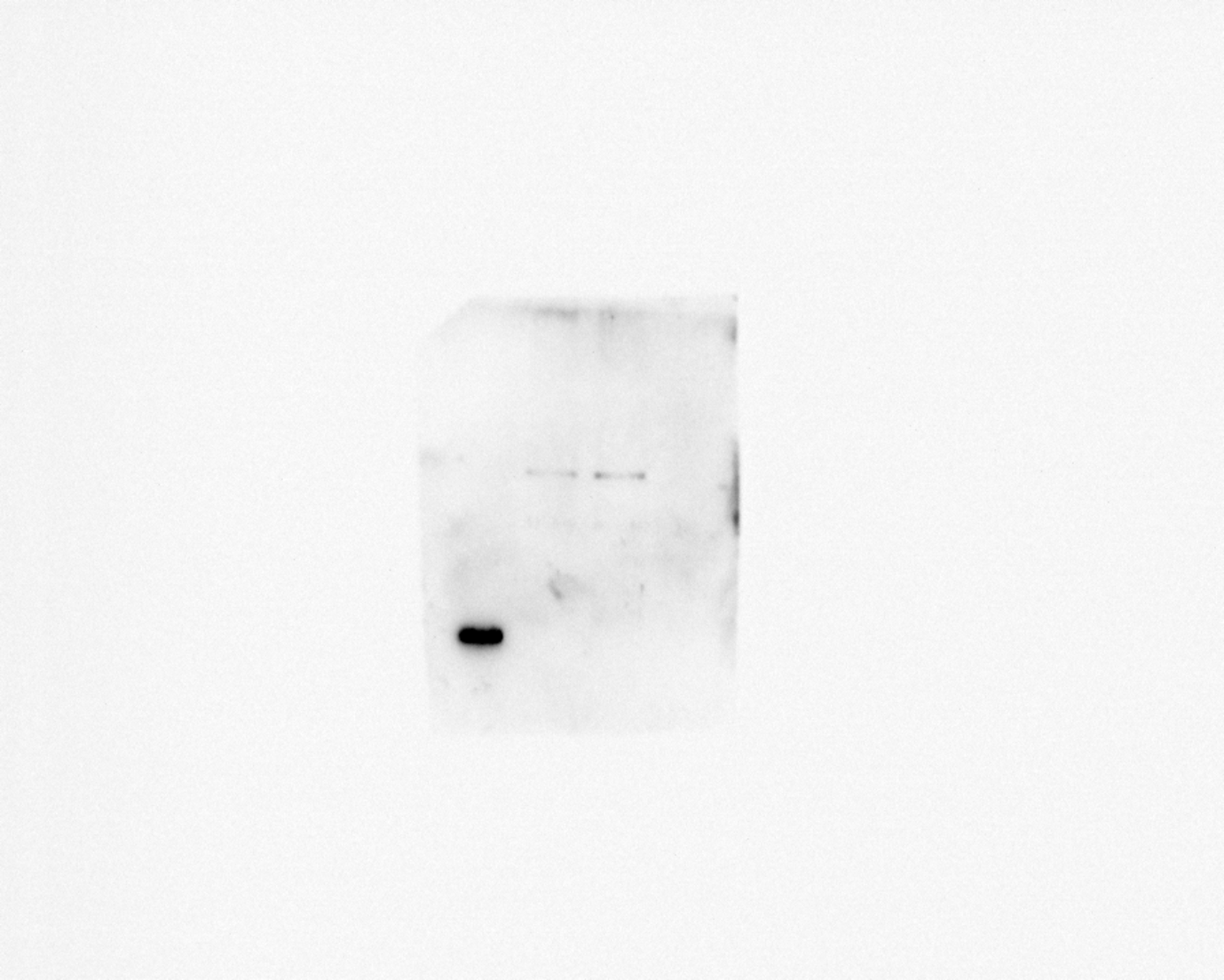

Supplement: Figure 7—source data 1. — Including uncropped Western blot images and raw statistics. [file elife-76436-fig7-data1.zip › Figure 7-Source Data 1/Figure 7C full raw unedited/IB-GST.tif]

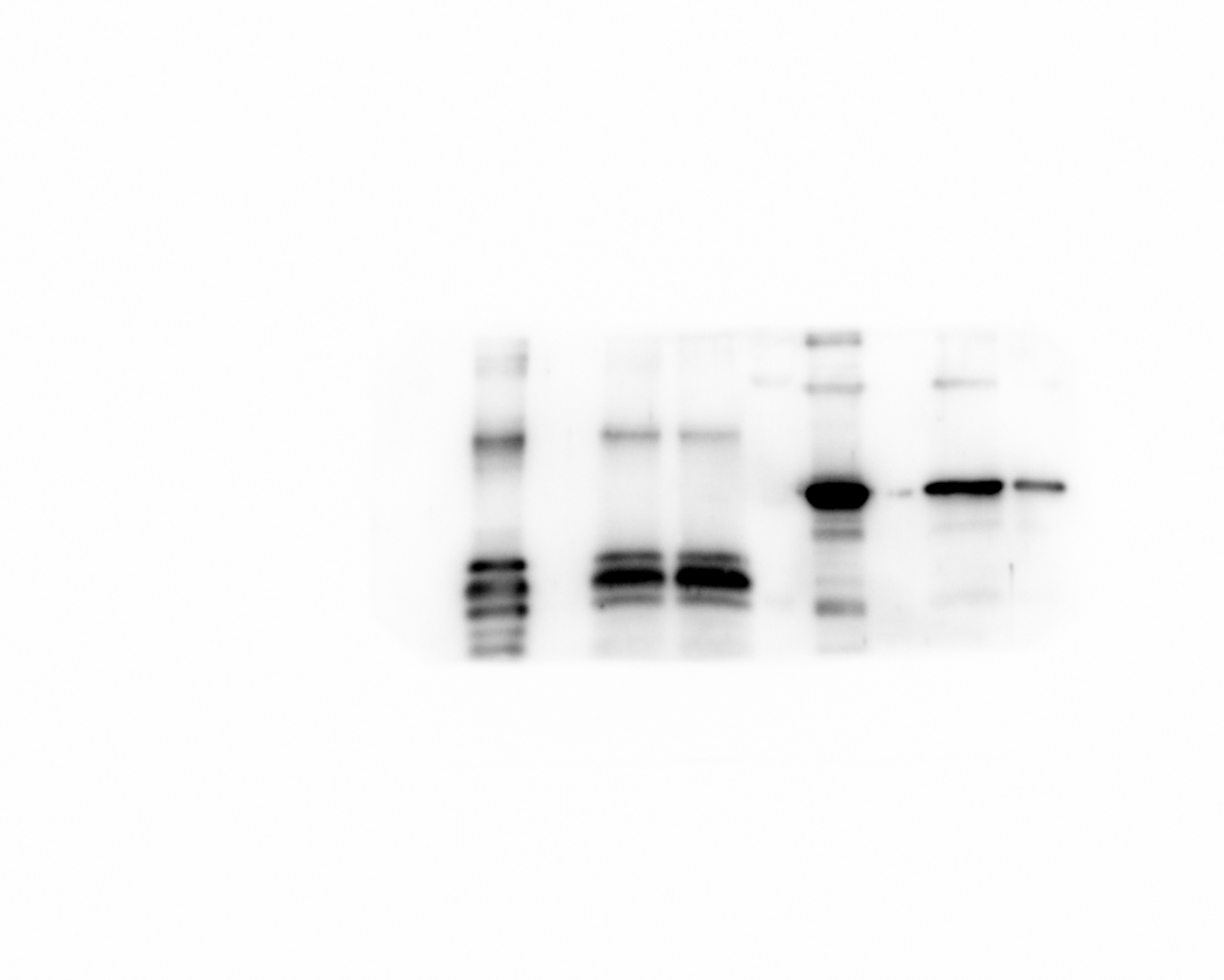

Supplement: Figure 7—source data 1. — Including uncropped Western blot images and raw statistics. [file elife-76436-fig7-data1.zip › Figure 7-Source Data 1/Figure 7C full raw unedited/IB-HA.tif]

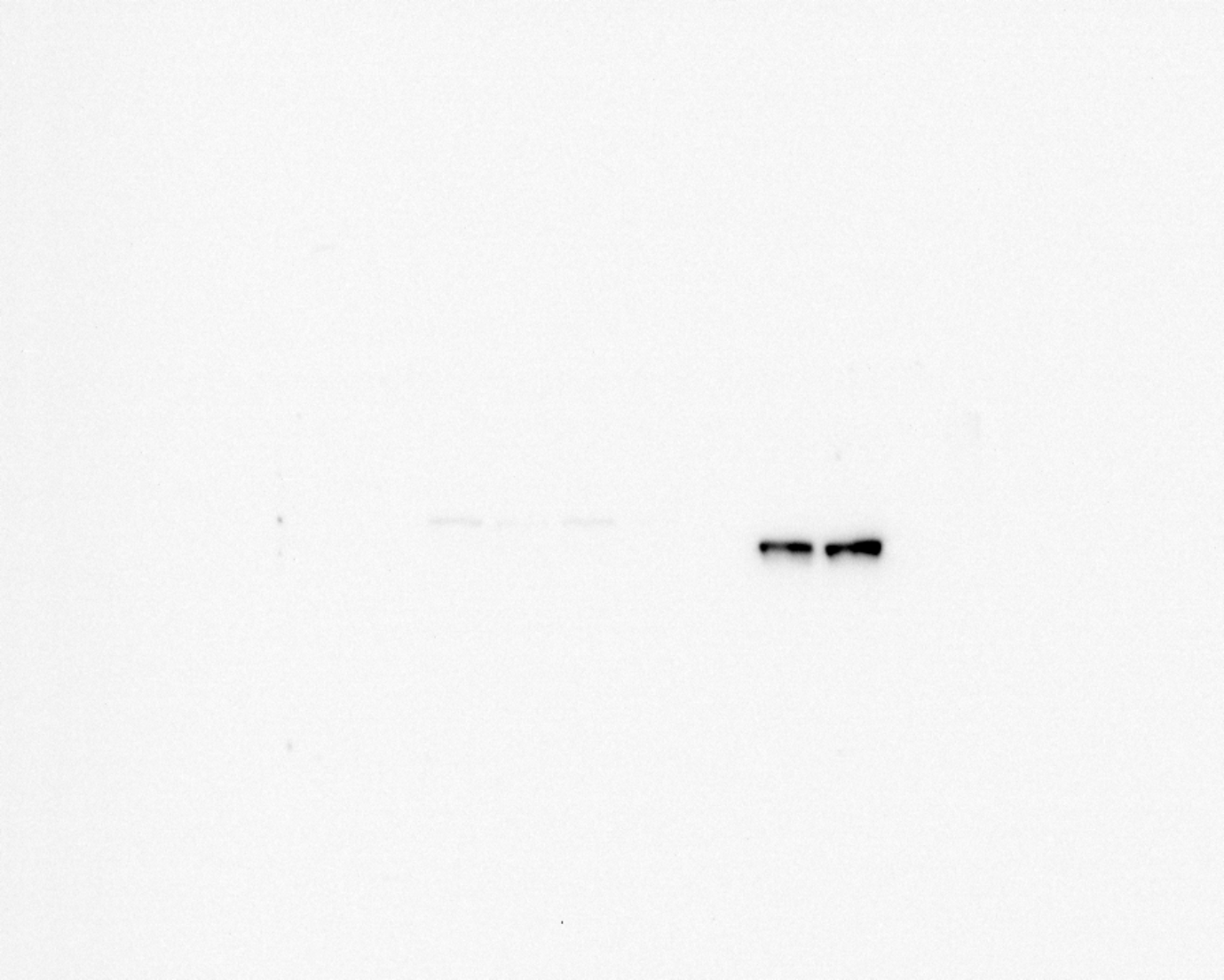

Supplement: Figure 7—source data 1. — Including uncropped Western blot images and raw statistics. [file elife-76436-fig7-data1.zip › Figure 7-Source Data 1/Figure 7D full raw unedited/Input-IB-MYC.tif]

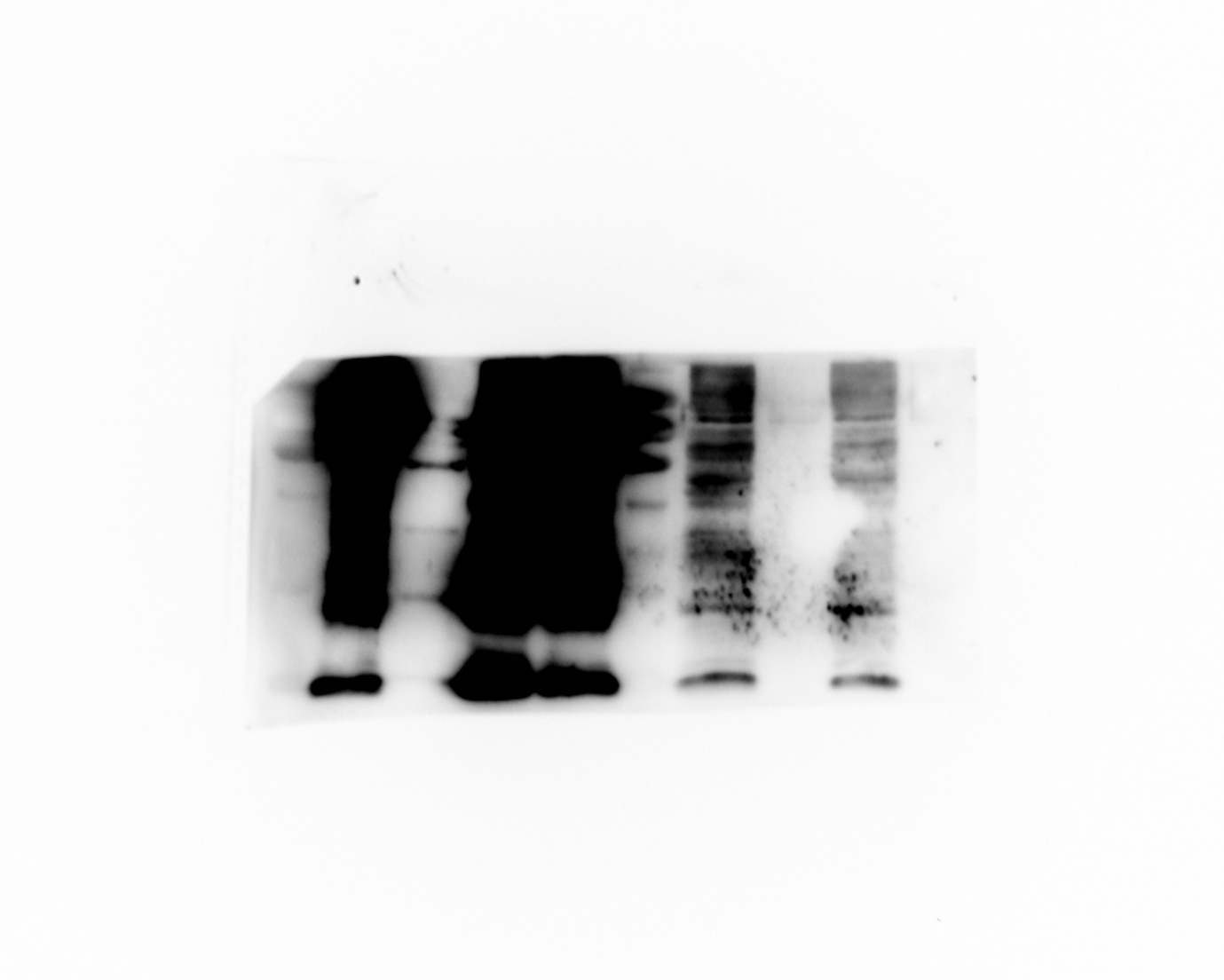

Supplement: Figure 7—source data 1. — Including uncropped Western blot images and raw statistics. [file elife-76436-fig7-data1.zip › Figure 7-Source Data 1/Figure 7D full raw unedited/IP-IB-HA.tif]

Figure 7-figure supplement 2E


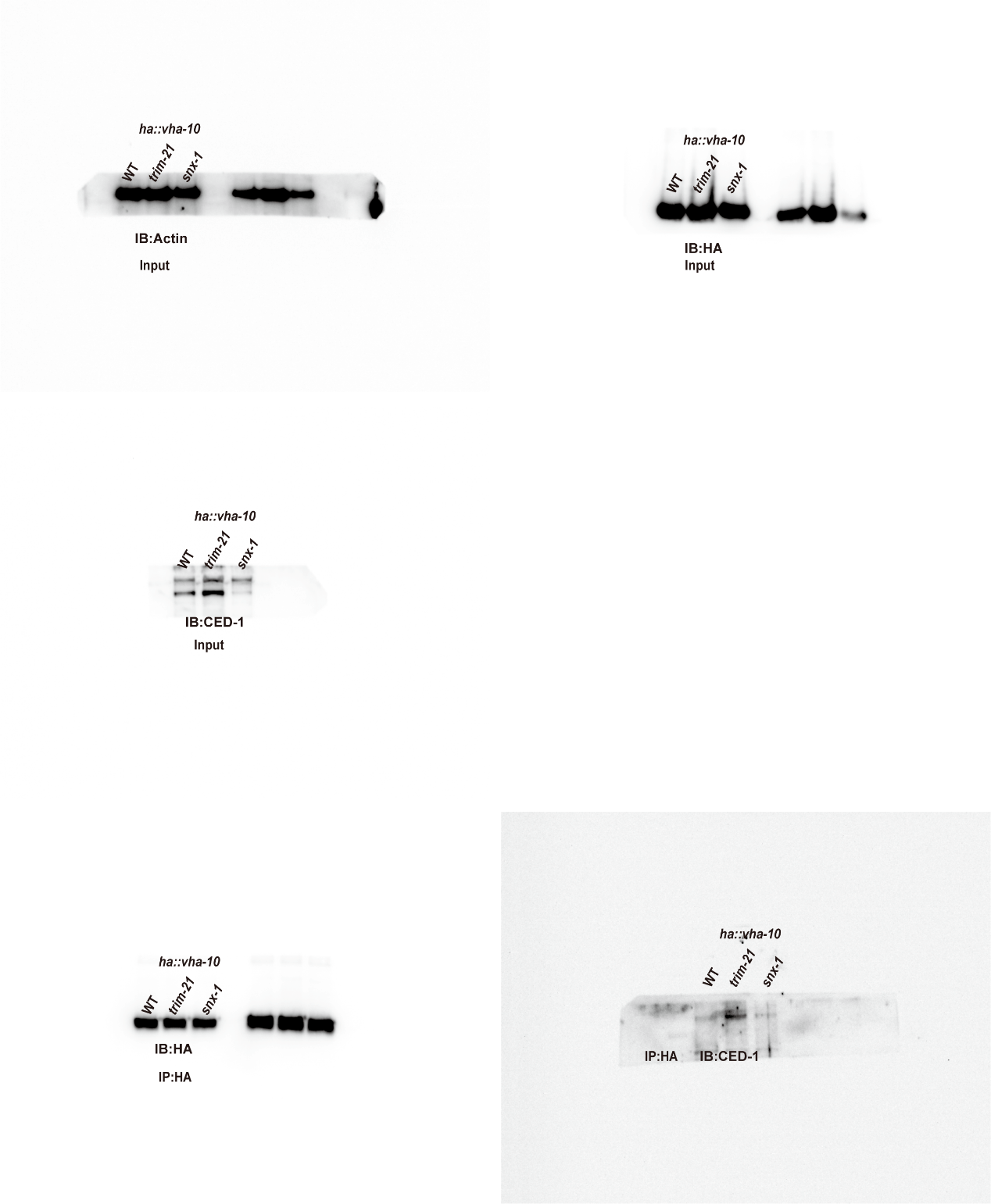

Supplement: Figure 7—figure supplement 2—source data 1. — Including uncropped Western blot images and raw statistics. [file elife-76436-fig7-figsupp2-data1.zip › Figure 7-figure supplement 2 uncroppped blot with relevant bands.docx]

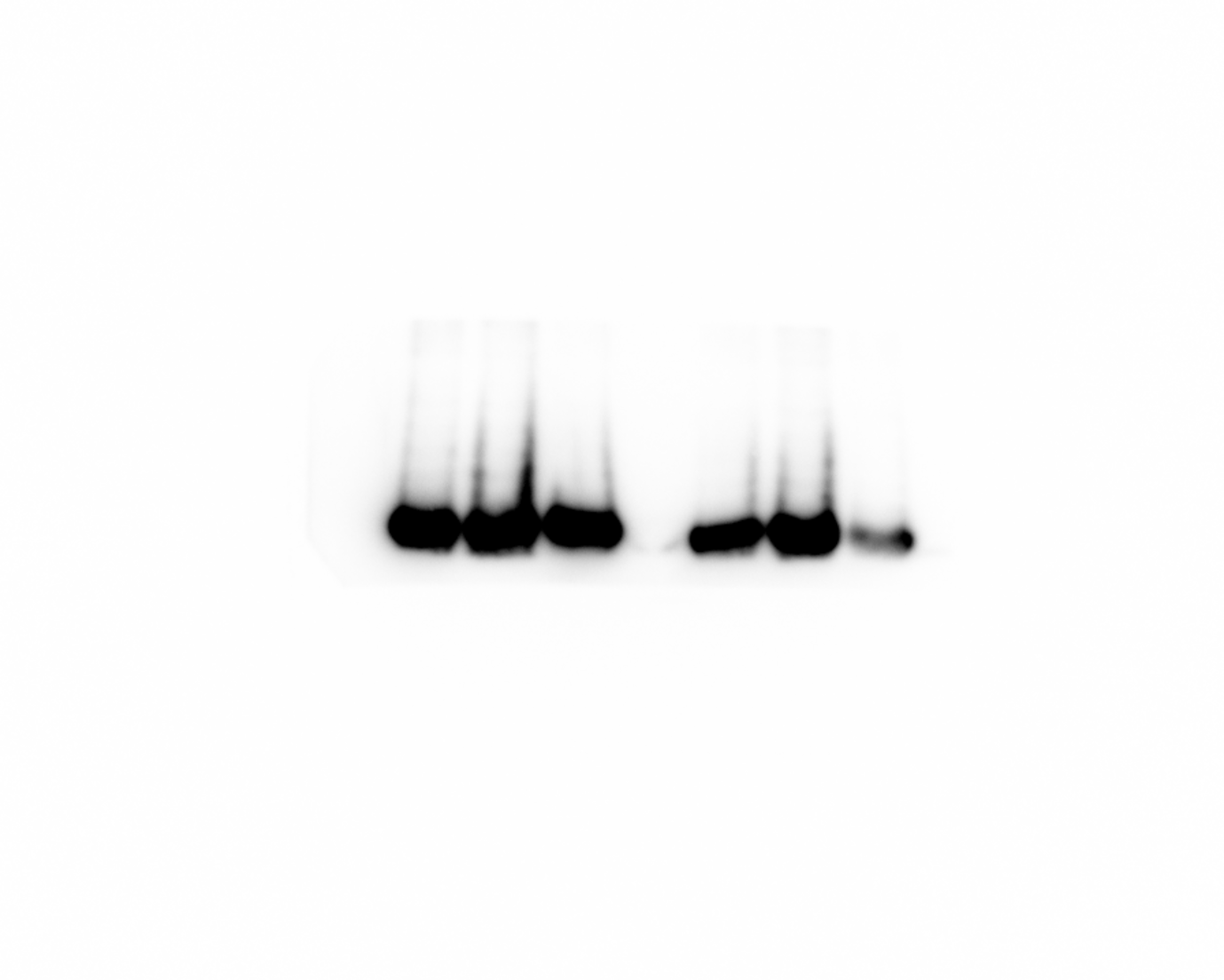

Supplement: Figure 7—figure supplement 2—source data 1. — Including uncropped Western blot images and raw statistics. [file elife-76436-fig7-figsupp2-data1.zip › Figure 7-figure supplement 2E full raw unedited/Input-IB-HA.tif]

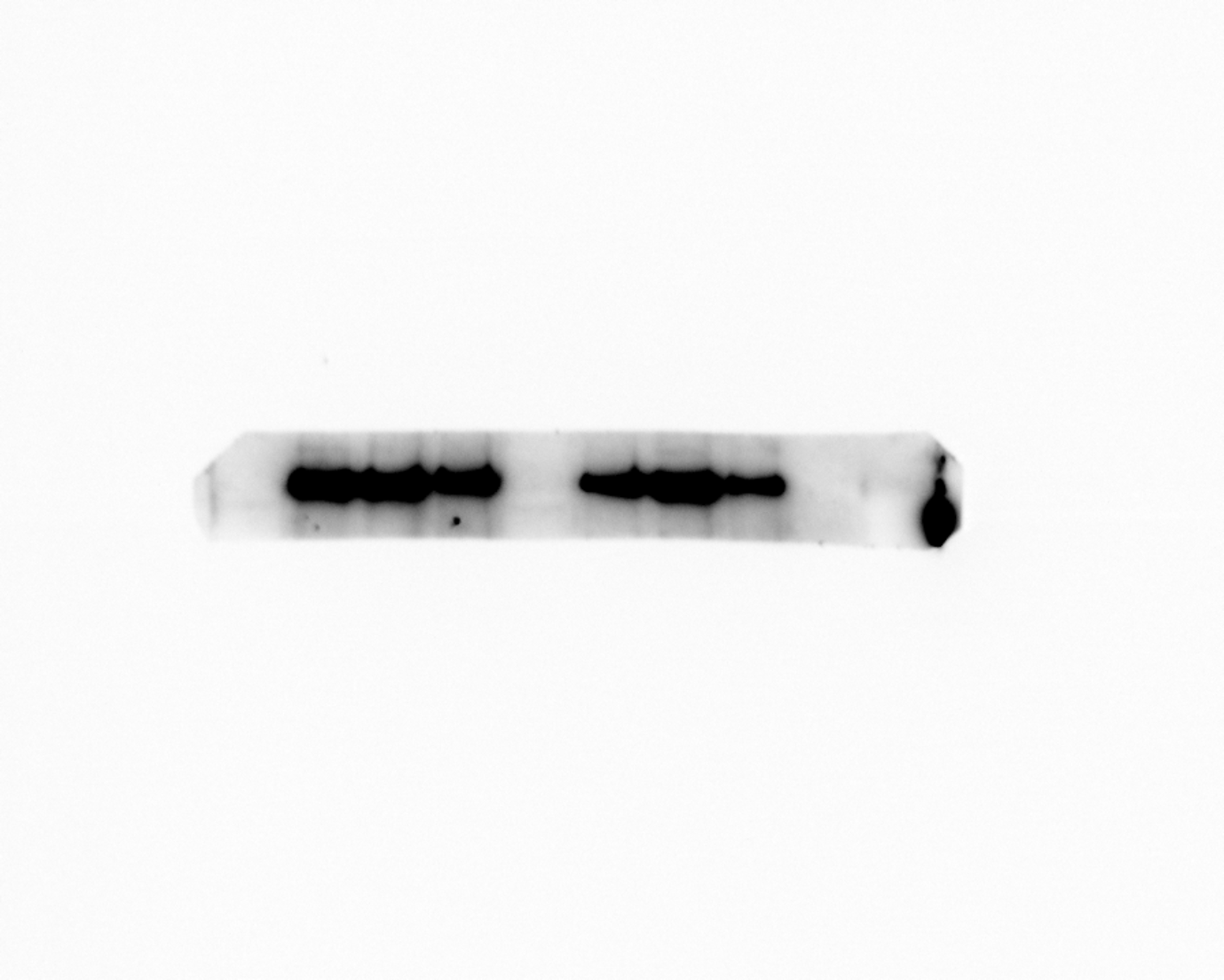

Supplement: Figure 7—figure supplement 2—source data 1. — Including uncropped Western blot images and raw statistics. [file elife-76436-fig7-figsupp2-data1.zip › Figure 7-figure supplement 2E full raw unedited/Input-IB-Actin.tif]

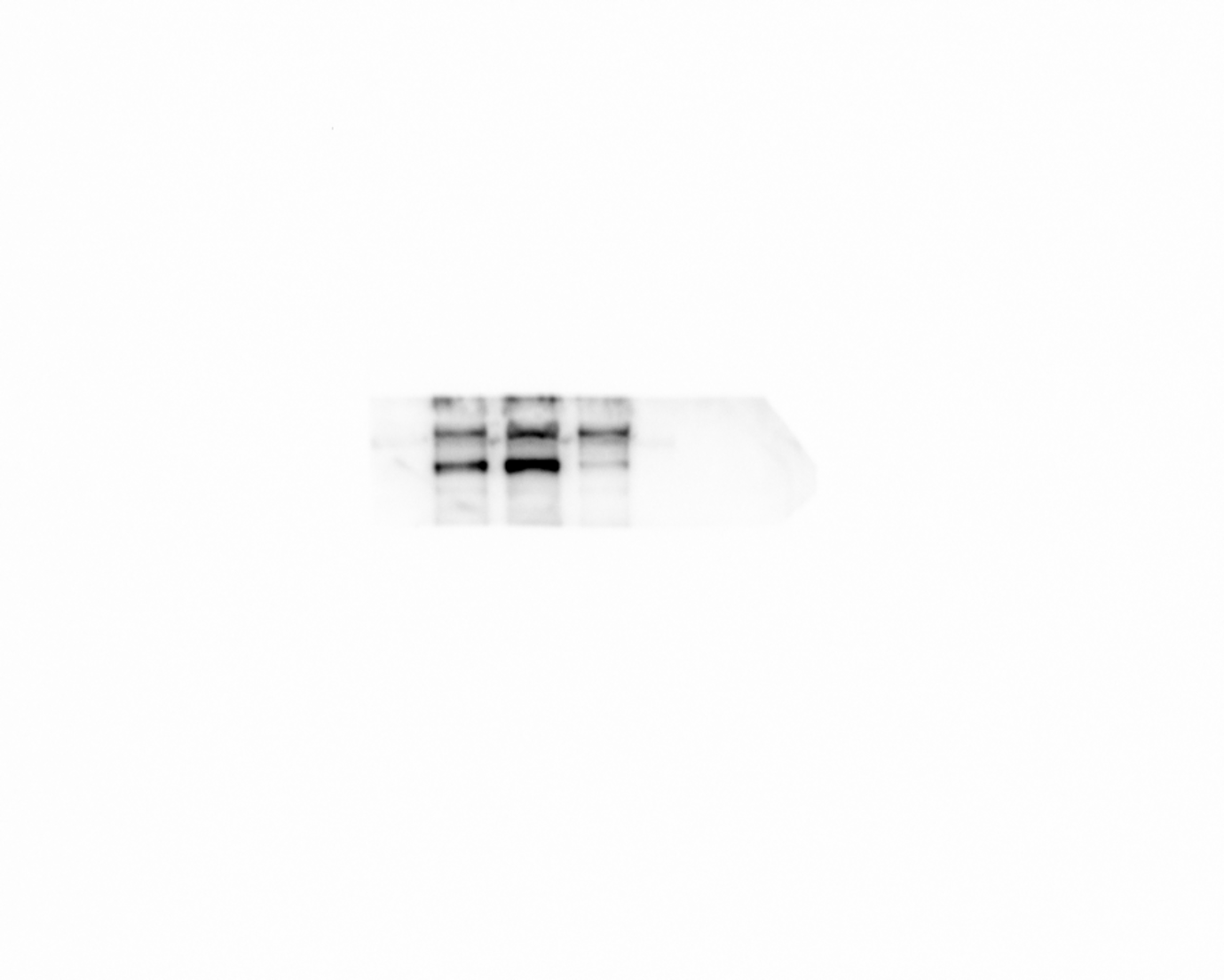

Supplement: Figure 7—figure supplement 2—source data 1. — Including uncropped Western blot images and raw statistics. [file elife-76436-fig7-figsupp2-data1.zip › Figure 7-figure supplement 2E full raw unedited/Input-IB-CED-1.tif]

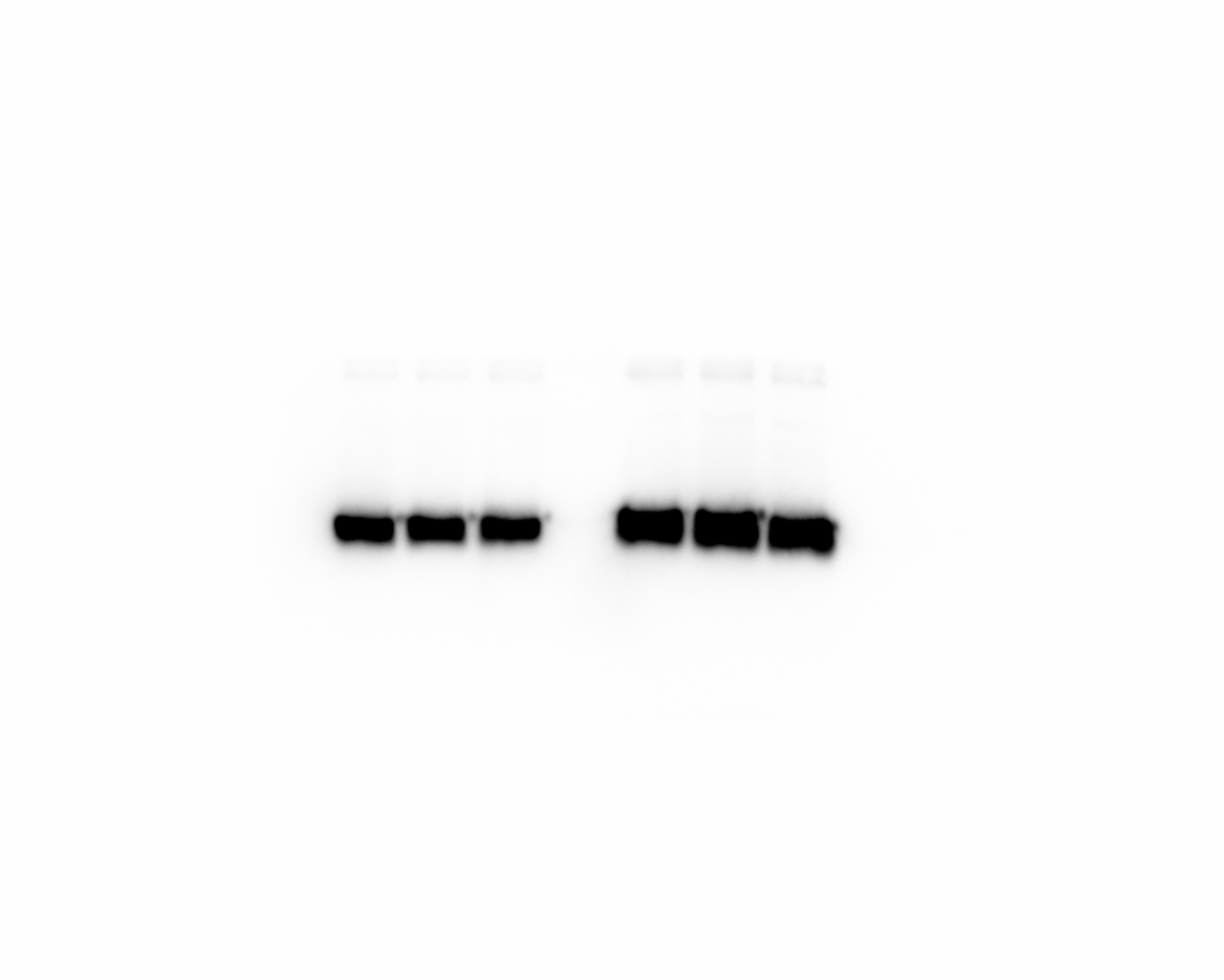

Supplement: Figure 7—figure supplement 2—source data 1. — Including uncropped Western blot images and raw statistics. [file elife-76436-fig7-figsupp2-data1.zip › Figure 7-figure supplement 2E full raw unedited/IP-IB-HA.tif]

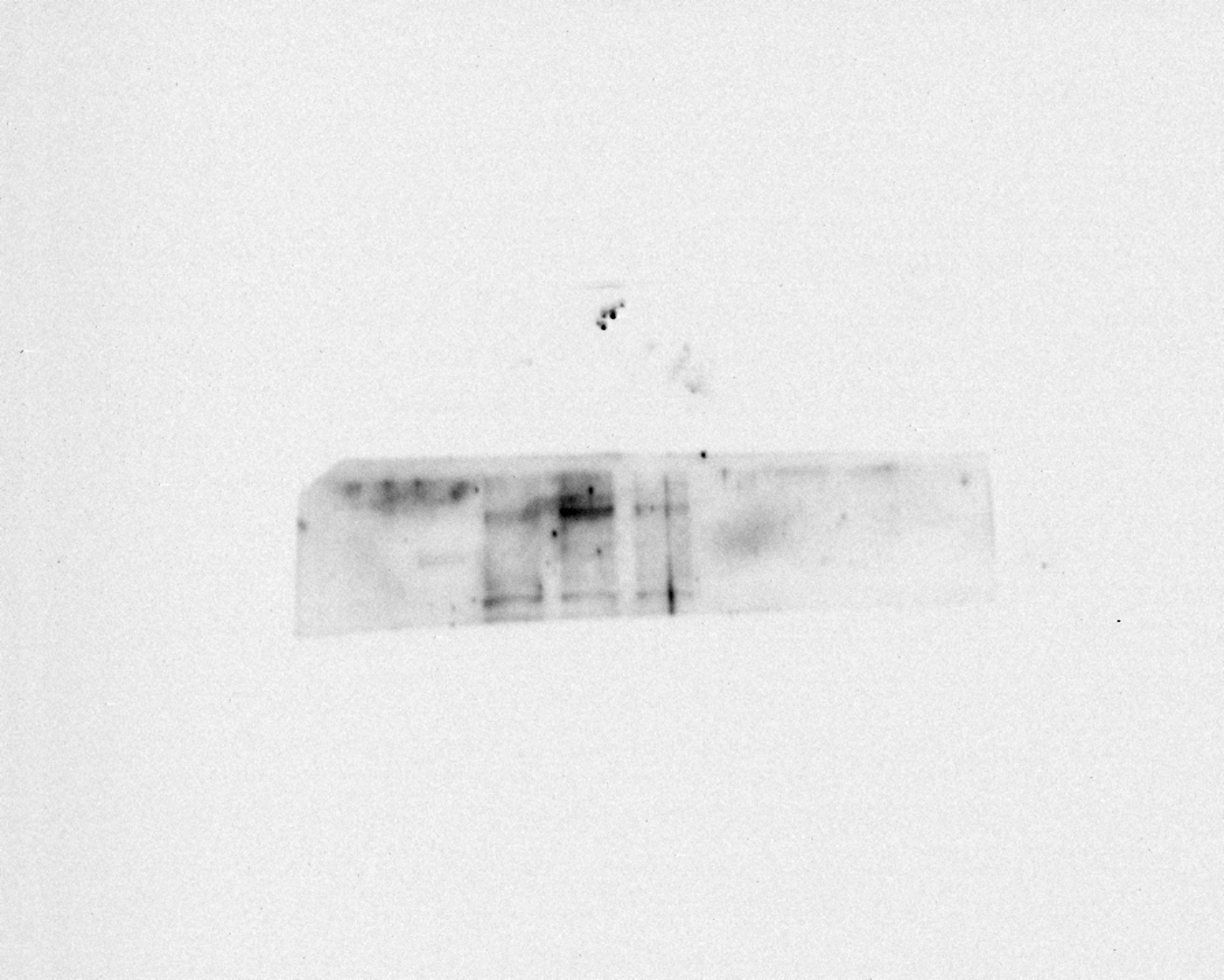

Supplement: Figure 7—figure supplement 2—source data 1. — Including uncropped Western blot images and raw statistics. [file elife-76436-fig7-figsupp2-data1.zip › Figure 7-figure supplement 2E full raw unedited/IP-IB-CED-1.tif]
