# Supplementary material for: trim-21 promotes proteasomal degradation of CED-1 for apoptotic cell clearance in C. elegans
Source: eLife. 2022 Aug 5;11:e76436. doi: 10.7554/eLife.76436 (PMC9388098; doi:10.7554/eLife.76436)

Figure 4-figure supplement 1B


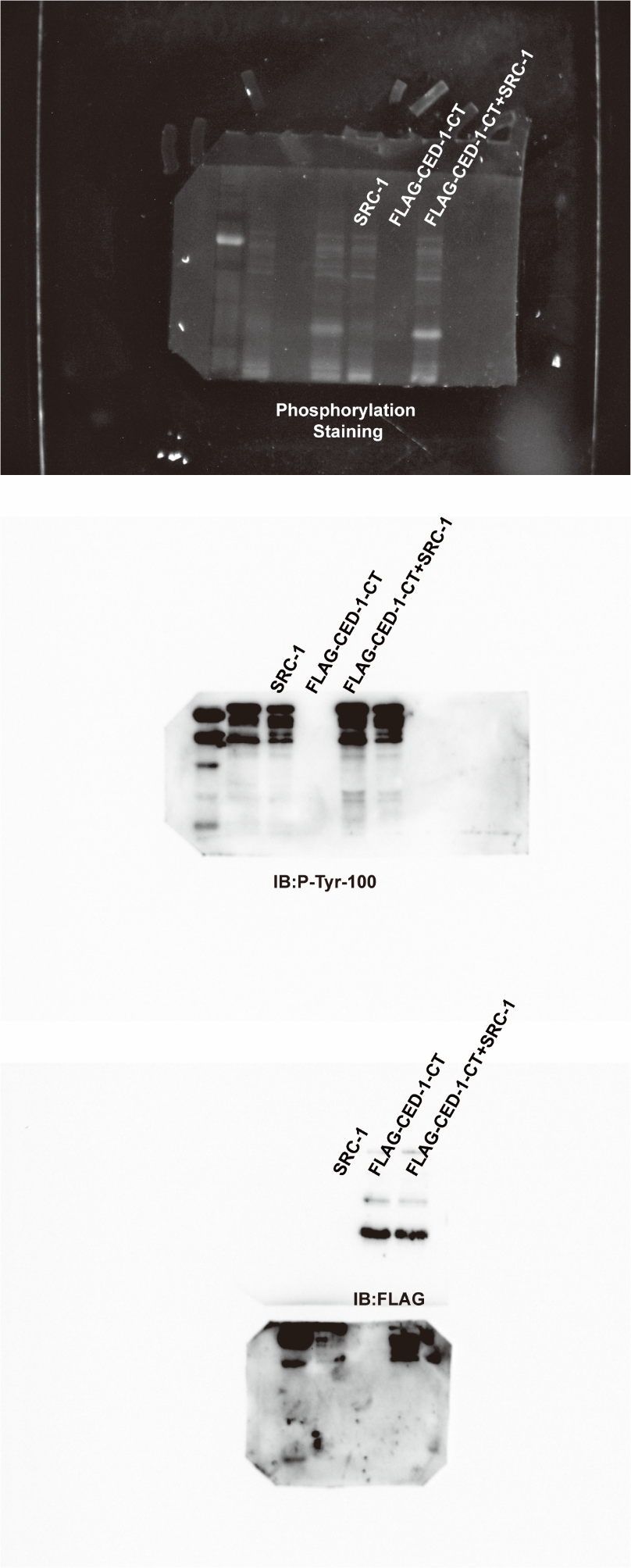


Figure 4-figure supplement 1D


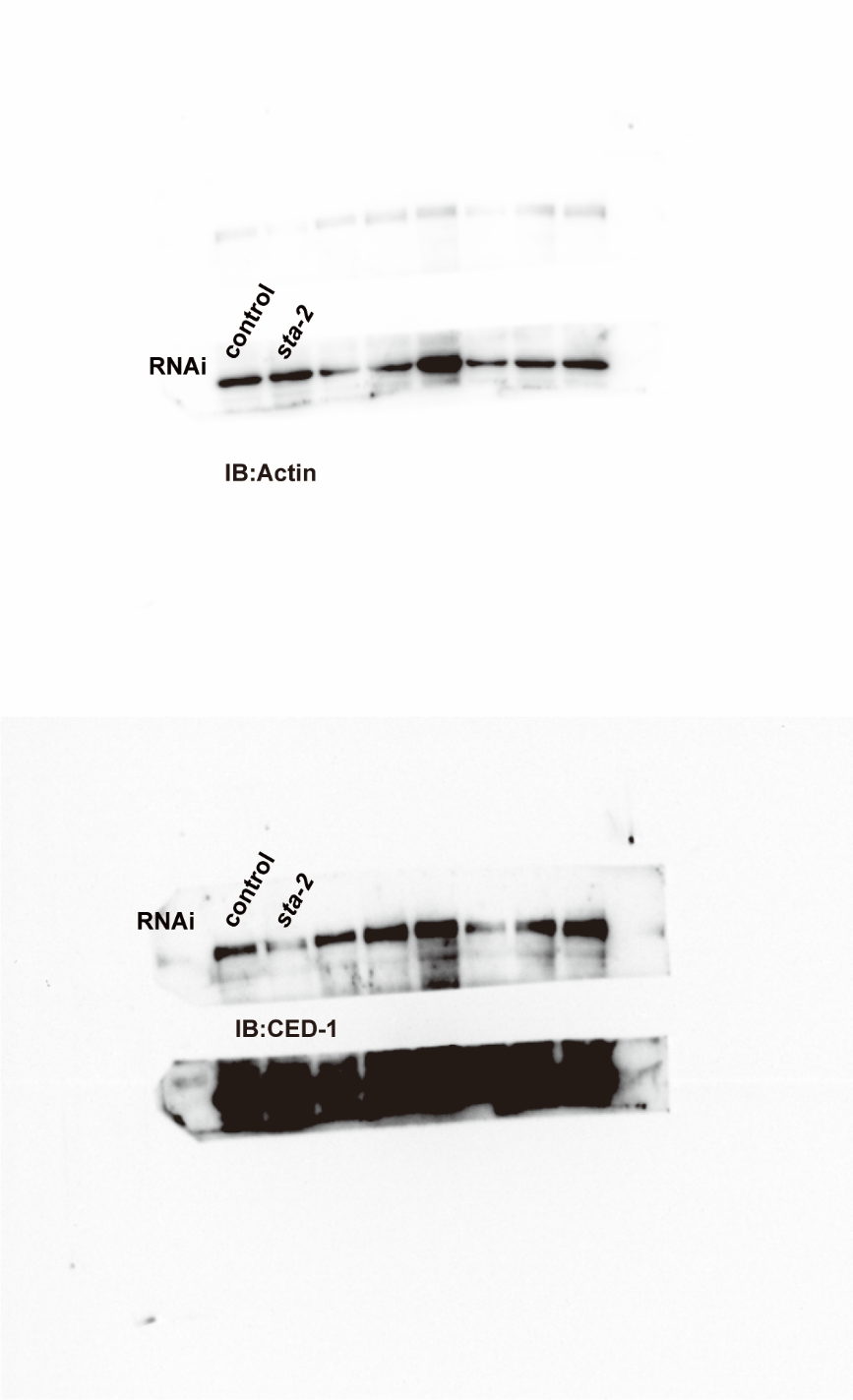


Figure 4-figure supplement 1I


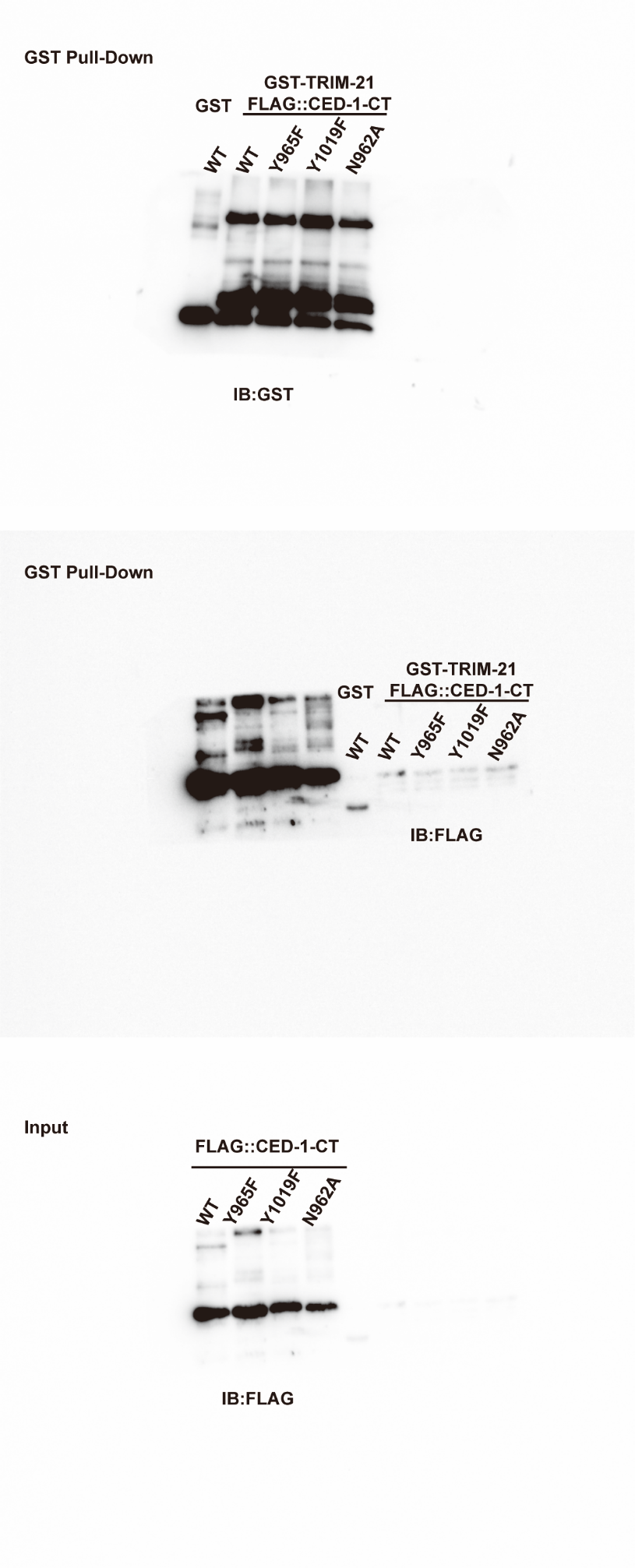


Figure 4-figure supplement 1J


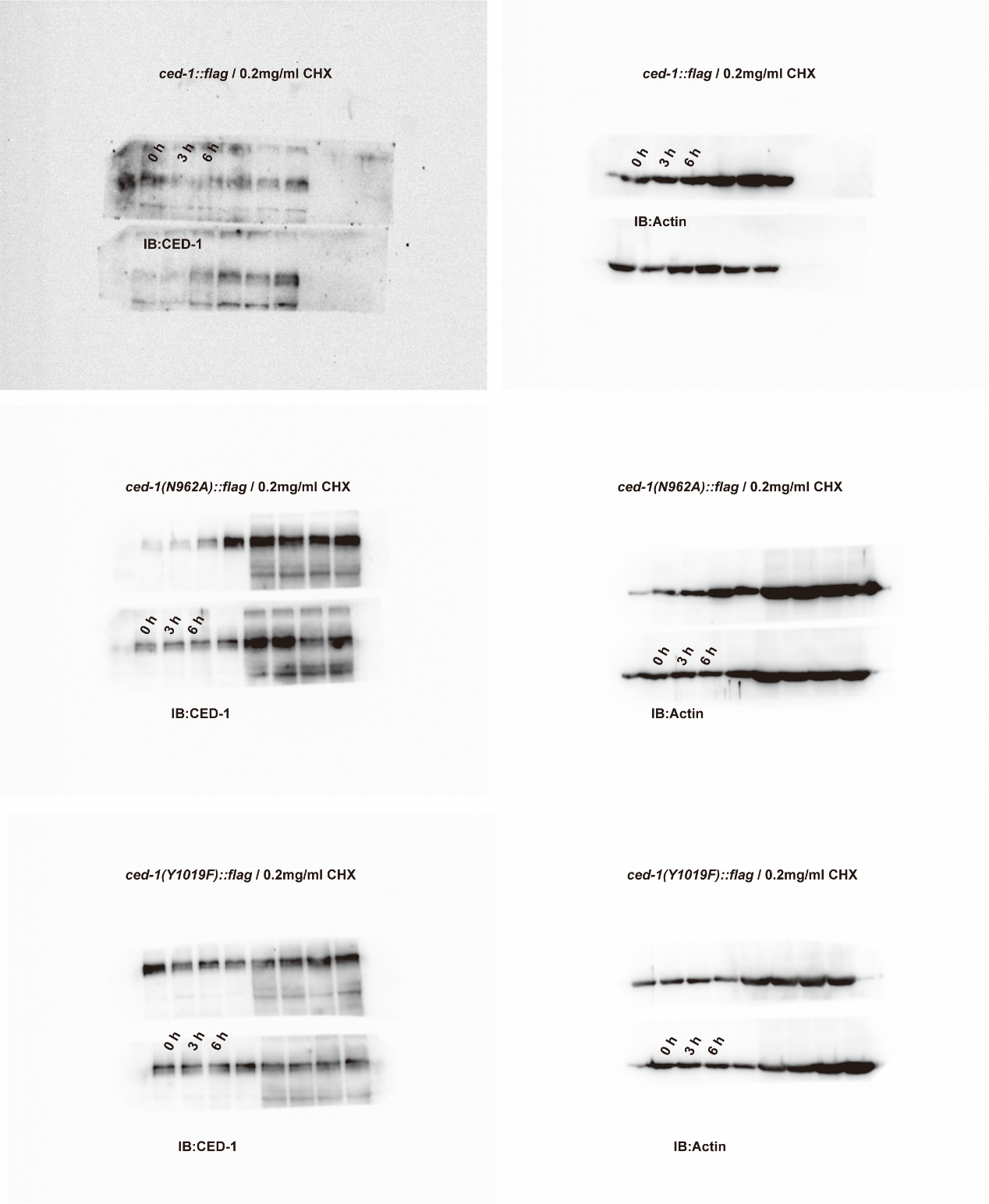


Figure 4-figure supplement 1L


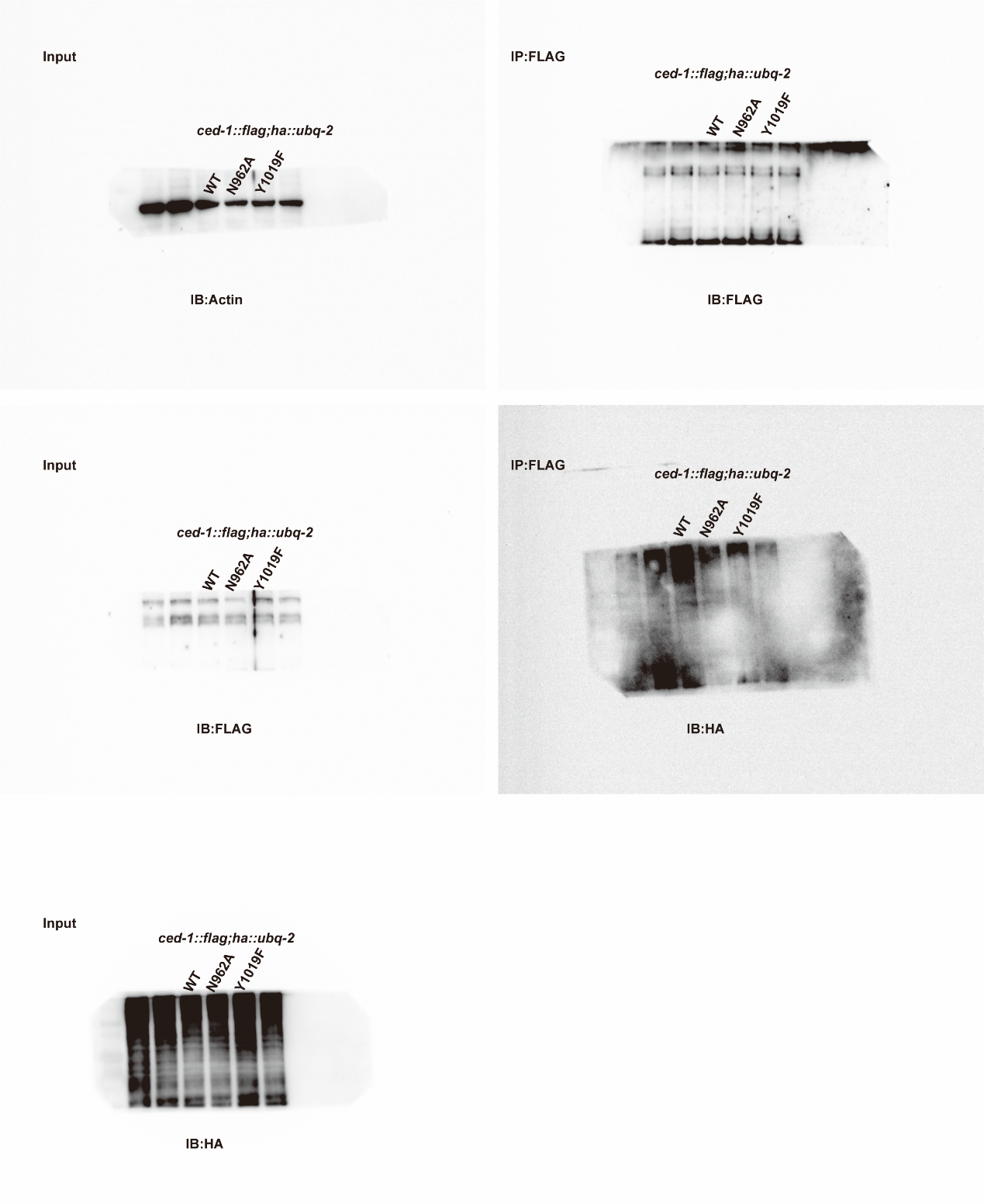

Supplement: Figure 4—figure supplement 1—source data 1. — Including uncropped Western blot images and raw statistics. [file elife-76436-fig4-figsupp1-data1.zip › Figure 4-figure supplement 1-Source Data 1/Figure 4-figure supplement 1 uncroppped blot with relevant bands.docx]
